# Supplementary material for: Genetic diversity of pomegranate germplasm collection from Spain determined by fruit, seed, leaf and flower characteristics
Source: PeerJ. 2016 Jul 19;4:e2214. doi: 10.7717/peerj.2214 (PMC4957998; doi:10.7717/peerj.2214)
Supplement: Data S1 — List of data measured in fruits, aryl, seeds, leaves and flowers. [file peerj-04-2214-s001.doc]

**Raw Data**

**Data from fruit characters of pomegranate accessions**

| Period | Variety | FW | FD1 | FD2 | FL1 | FL2 | FL3 | Nc | PcMc | Ec | Rs |
| --- | --- | --- | --- | --- | --- | --- | --- | --- | --- | --- | --- |
| 1 | ME1 | 242,25 | 82,44 | 13,95 | 68,74 | 82,91 | 14,17 | 6,00 | 94,02 | 1,26 | 61,19 |
| 1 | ME1 | 386,41 | 88,49 | 23,57 | 74,79 | 97,86 | 23,07 | 7,00 | 160,26 | 2,68 | 58,53 |
| 1 | ME1 | 304,68 | 85,58 | 18,45 | 74,80 | 92,67 | 17,87 | 7,00 | 124,41 | 2,52 | 59,17 |
| 1 | ME1 | 299,65 | 83,62 | 18,58 | 74,46 | 88,74 | 14,28 | 6,00 | 118,65 | 2,28 | 60,40 |
| 1 | ME1 | 336,85 | 89,06 | 29,93 | 72,40 | 95,54 | 23,14 | 7,00 | 149,18 | 4,42 | 55,71 |
| 1 | ME1 | 294,69 | 84,57 | 17,34 | 72,80 | 91,82 | 19,02 | 6,00 | 130,14 | 2,75 | 55,84 |
| 1 | ME1 | 315,20 | 83,97 | 20,03 | 73,23 | 92,93 | 19,70 | 8,00 | 135,88 | 2,12 | 56,89 |
| 1 | ME1 | 402,22 | 92,00 | 18,74 | 78,79 | 98,22 | 19,43 | 7,00 | 178,56 | 2,63 | 55,61 |
| 1 | ME1 | 347,86 | 88,01 | 18,70 | 75,54 | 91,97 | 16,43 | 8,00 | 152,46 | 3,97 | 56,17 |
| 1 | ME1 | 358,94 | 89,09 | 19,32 | 78,67 | 92,10 | 13,43 | 7,00 | 159,08 | 2,64 | 55,68 |
| 1 | ME2 | 222,89 | 74,09 | 19,25 | 64,90 | 83,40 | 18,50 | 6,00 | 96,56 | 3,97 | 56,68 |
| 1 | ME2 | 351,20 | 84,90 | 22,30 | 76,79 | 93,22 | 16,43 | 6,00 | 189,38 | 5,93 | 46,08 |
| 1 | ME2 | 252,93 | 78,97 | 19,10 | 67,91 | 86,82 | 18,91 | 5,00 | 129,86 | 3,47 | 48,66 |
| 1 | ME2 | 381,28 | 91,44 | 25,92 | 75,63 | 98,75 | 23,12 | 6,00 | 168,42 | 3,39 | 55,83 |
| 1 | ME2 | 370,08 | 91,38 | 19,91 | 80,40 | 95,36 | 14,96 | 7,00 | 181,42 | 4,45 | 50,98 |
| 1 | ME2 | 258,79 | 82,34 | 15,50 | 74,97 | 84,73 | 9,76 | 6,00 | 102,52 | 3,36 | 60,38 |
| 1 | ME2 | 272,32 | 77,54 | 23,74 | 68,30 | 88,39 | 20,09 | 6,00 | 158,35 | 5,00 | 41,85 |
| 1 | ME2 | 247,27 | 82,29 | 18,86 | 69,42 | 89,57 | 20,15 | 6,00 | 128,01 | 2,97 | 48,23 |
| 1 | ME2 | 302,50 | 87,54 | 20,80 | 71,81 | 91,17 | 19,36 | 6,00 | 136,15 | 3,14 | 54,99 |
| 1 | ME2 | 312,79 | 84,06 | 27,27 | 76,89 | 89,52 | 12,63 | 6,00 | 153,11 | 4,94 | 51,05 |
| 1 | ME3 | 252,01 | 77,98 | 17,22 | 70,40 | 89,05 | 18,65 | 6,00 | 100,48 | 4,18 | 60,13 |
| 1 | ME3 | 306,29 | 86,71 | 17,61 | 71,53 | 90,61 | 19,08 | 6,00 | 161,00 | 5,26 | 47,44 |
| 1 | ME3 | 330,70 | 87,07 | 21,91 | 76,86 | 96,78 | 19,92 | 6,00 | 156,85 | 7,73 | 52,57 |
| 1 | ME3 | 268,70 | 77,21 | 22,01 | 71,51 | 91,51 | 20,00 | 6,00 | 106,10 | 3,42 | 60,51 |
| 1 | ME3 | 272,02 | 84,39 | 19,98 | 73,35 | 82,10 | 8,75 | 6,00 | 109,40 | 3,97 | 59,78 |
| 1 | ME3 | 257,30 | 83,38 | 27,67 | 74,82 | 92,97 | 18,15 | 6,00 | 119,68 | 4,09 | 53,49 |
| 1 | ME3 | 319,70 | 80,04 | 15,64 | 70,16 | 78,43 | 8,27 | 7,00 | 105,50 | 3,24 | 67,00 |
| 1 | ME3 | 332,16 | 84,75 | 18,69 | 76,96 | 86,31 | 9,35 | 6,00 | 127,00 | 3,13 | 61,77 |
| 1 | ME3 | 245,49 | 76,05 | 18,07 | 64,62 | 79,95 | 15,33 | 7,00 | 89,04 | 3,01 | 63,73 |
| 1 | ME3 | 315,06 | 84,01 | 25,26 | 74,44 | 85,81 | 11,37 | 6,00 | 145,41 | 2,89 | 53,85 |
| 1 | ME31 | 249,37 | 78,76 | 17,45 | 70,61 | 88,24 | 17,63 | 7,00 | 116,10 | 3,02 | 53,44 |
| 1 | ME31 | 270,92 | 81,03 | 19,14 | 74,36 | 92,34 | 17,98 | 8,00 | 120,52 | 2,61 | 55,51 |
| 1 | ME31 | 268,28 | 83,22 | 18,54 | 72,60 | 88,94 | 16,34 | 8,00 | 109,37 | 2,34 | 59,23 |
| 1 | ME31 | 237,97 | 79,36 | 18,02 | 67,20 | 85,35 | 18,15 | 6,00 | 105,98 | 4,03 | 55,46 |
| 1 | ME31 | 345,53 | 89,55 | 22,40 | 76,42 | 95,78 | 19,36 | 6,00 | 174,97 | 4,28 | 49,36 |
| 1 | ME31 | 271,49 | 83,80 | 18,41 | 71,40 | 82,52 | 11,12 | 6,00 | 118,54 | 2,45 | 56,34 |
| 1 | ME31 | 191,09 | 73,13 | 16,64 | 60,50 | 77,57 | 17,07 | 7,00 | 90,87 | 2,40 | 52,45 |
| 1 | ME31 | 461,97 | 98,28 | 19,45 | 82,82 | 97,06 | 14,24 | 6,00 | 172,26 | 4,62 | 62,71 |
| 1 | ME31 | 289,17 | 82,11 | 16,29 | 76,38 | 90,19 | 13,81 | 8,00 | 155,53 | 2,86 | 46,22 |
| 1 | ME31 | 351,35 | 88,52 | 19,29 | 74,10 | 97,88 | 23,78 | 7,00 | 115,53 | 3,64 | 67,12 |
| 1 | ME4 | 351,43 | 89,01 | 18,54 | 80,21 | 98,12 | 17,91 | 9,00 | 170,75 | 2,76 | 51,41 |
| 1 | ME4 | 201,62 | 74,66 | 16,67 | 64,79 | 80,06 | 15,27 | 7,00 | 95,65 | 3,37 | 52,56 |
| 1 | ME4 | 307,53 | 87,93 | 31,97 | 70,50 | 89,60 | 19,10 | 8,00 | 182,76 | 3,71 | 40,57 |
| 1 | ME4 | 227,14 | 80,98 | 18,10 | 64,15 | 84,00 | 19,85 | 8,00 | 105,40 | 3,38 | 53,60 |
| 1 | ME4 | 231,22 | 76,53 | 21,86 | 64,02 | 80,17 | 16,15 | 7,00 | 129,87 | 4,87 | 43,83 |
| 1 | ME4 | 421,53 | 99,54 | 20,74 | 85,59 | 101,66 | 16,07 | 9,00 | 217,07 | 6,07 | 48,50 |
| 1 | ME4 | 163,49 | 71,32 | 17,22 | 56,43 | 76,54 | 20,11 | 6,00 | 84,80 | 3,94 | 48,13 |
| 1 | ME4 | 253,03 | 80,23 | 21,56 | 68,76 | 83,71 | 14,95 | 8,00 | 117,83 | 4,81 | 53,43 |
| 1 | ME4 | 316,19 | 89,02 | 18,75 | 70,50 | 91,14 | 20,64 | 8,00 | 148,41 | 5,49 | 53,06 |
| 1 | ME4 | 419,38 | 94,69 | 19,83 | 82,49 | 101,47 | 18,98 | 7,00 | 177,58 | 3,97 | 57,66 |
| 1 | ME5 | 424,11 | 89,37 | 20,16 | 85,19 | 101,51 | 16,32 | 6,00 | 213,93 | 3,73 | 49,56 |
| 1 | ME5 | 361,21 | 92,24 | 25,21 | 77,80 | 100,23 | 22,43 | 7,00 | 214,88 | 4,42 | 40,51 |
| 1 | ME5 | 210,44 | 78,61 | 18,97 | 66,12 | 82,69 | 16,57 | 8,00 | 106,45 | 3,65 | 49,42 |
| 1 | ME5 | 389,11 | 92,57 | 24,28 | 79,66 | 89,50 | 9,84 | 8,00 | 202,42 | 4,45 | 47,98 |
| 1 | ME5 | 241,98 | 83,23 | 24,32 | 68,25 | 81,27 | 13,02 | 6,00 | 121,25 | 4,49 | 49,89 |
| 1 | ME5 | 230,07 | 77,07 | 21,59 | 66,92 | 83,55 | 16,63 | 7,00 | 100,36 | 2,95 | 56,38 |
| 1 | ME5 | 173,82 | 69,08 | 17,00 | 61,49 | 78,20 | 16,71 | 7,00 | 70,40 | 3,10 | 59,50 |
| 1 | ME5 | 213,88 | 76,71 | 17,15 | 62,65 | 82,83 | 20,18 | 6,00 | 73,95 | 4,34 | 65,42 |
| 1 | ME5 | 436,90 | 99,38 | 24,22 | 79,03 | 99,00 | 19,97 | 8,00 | 225,65 | 5,99 | 48,35 |
| 1 | ME5 | 240,26 | 78,30 | 19,18 | 66,24 | 85,01 | 18,77 | 6,00 | 133,56 | 4,51 | 44,41 |
| 1 | ME6 | 432,70 | 97,80 | 24,78 | 80,98 | 103,29 | 22,31 | 7,00 | 180,72 | 3,91 | 58,23 |
| 1 | ME6 | 569,21 | 107,31 | 23,94 | 87,70 | 101,51 | 13,81 | 9,00 | 240,23 | 7,06 | 57,80 |
| 1 | ME6 | 374,36 | 90,20 | 18,33 | 81,01 | 93,51 | 12,50 | 6,00 | 156,62 | 5,08 | 58,16 |
| 1 | ME6 | 238,03 | 88,60 | 22,79 | 79,39 | 96,79 | 17,40 | 7,00 | 110,91 | 6,57 | 53,41 |
| 1 | ME6 | 299,60 | 85,78 | 19,88 | 74,74 | 86,59 | 11,85 | 6,00 | 175,88 | 4,78 | 41,30 |
| 1 | ME6 | 263,82 | 81,88 | 22,82 | 67,29 | 78,89 | 11,60 | 7,00 | 117,98 | 4,18 | 55,28 |
| 1 | ME6 | 375,39 | 91,72 | 19,90 | 80,96 | 92,35 | 11,39 | 7,00 | 156,60 | 3,79 | 58,28 |
| 1 | ME6 | 216,72 | 77,08 | 18,88 | 65,25 | 78,84 | 13,59 | 7,00 | 105,20 | 4,36 | 51,46 |
| 1 | ME6 | 272,03 | 80,30 | 18,51 | 77,01 | 91,05 | 14,04 | 6,00 | 121,40 | 4,60 | 55,37 |
| 1 | ME6 | 305,20 | 84,07 | 18,29 | 82,17 | 93,78 | 11,61 | 8,00 | 115,79 | 3,06 | 62,06 |
| 1 | ME7 | 431,43 | 93,87 | 17,39 | 86,96 | 101,82 | 14,86 | 6,00 | 185,34 | 3,83 | 57,04 |
| 1 | ME7 | 173,04 | 71,46 | 18,78 | 60,48 | 79,42 | 18,94 | 5,00 | 74,84 | 3,79 | 56,75 |
| 1 | ME7 | 210,54 | 76,67 | 19,21 | 65,68 | 77,76 | 12,08 | 7,00 | 101,71 | 4,35 | 51,69 |
| 1 | ME7 | 176,43 | 72,92 | 20,58 | 58,69 | 81,19 | 22,50 | 6,00 | 85,72 | 3,98 | 51,41 |
| 1 | ME7 | 389,96 | 94,31 | 20,68 | 77,29 | 97,68 | 20,39 | 6,00 | 203,74 | 5,32 | 47,75 |
| 1 | ME7 | 277,85 | 82,52 | 19,30 | 72,30 | 82,51 | 10,21 | 7,00 | 113,43 | 5,07 | 59,18 |
| 1 | ME7 | 230,82 | 76,43 | 20,53 | 68,14 | 79,85 | 11,71 | 6,00 | 140,34 | 4,41 | 39,20 |
| 1 | ME7 | 213,72 | 75,59 | 26,70 | 64,89 | 82,28 | 17,39 | 7,00 | 98,95 | 4,66 | 53,70 |
| 1 | ME7 | 438,47 | 64,02 | 15,88 | 59,32 | 78,47 | 19,15 | 6,00 | 160,77 | 4,86 | 63,33 |
| 1 | ME7 | 251,00 | 80,66 | 22,84 | 68,44 | 83,50 | 15,06 | 7,00 | 110,76 | 4,22 | 55,87 |
| 1 | ME8 | 243,70 | 80,87 | 20,11 | 67,73 | 86,20 | 18,47 | 7,00 | 122,85 | 4,12 | 49,59 |
| 1 | ME8 | 165,90 | 69,62 | 20,14 | 59,87 | 74,74 | 14,87 | 7,00 | 77,45 | 4,20 | 53,32 |
| 1 | ME8 | 288,86 | 80,02 | 23,72 | 69,84 | 91,78 | 21,94 | 7,00 | 121,94 | 3,98 | 57,79 |
| 1 | ME8 | 205,36 | 74,25 | 15,43 | 63,93 | 81,81 | 17,88 | 6,00 | 91,55 | 2,98 | 55,42 |
| 1 | ME8 | 171,05 | 72,94 | 17,43 | 57,06 | 75,56 | 18,50 | 6,00 | 79,35 | 3,21 | 53,61 |
| 1 | ME8 | 203,86 | 76,42 | 16,34 | 64,97 | 82,05 | 17,08 | 6,00 | 91,18 | 2,93 | 55,27 |
| 1 | ME8 | 328,44 | 80,41 | 21,89 | 77,82 | 99,54 | 21,72 | 5,00 | 170,32 | 4,52 | 48,14 |
| 1 | ME8 | 166,65 | 69,39 | 20,95 | 61,96 | 75,00 | 13,04 | 6,00 | 91,10 | 4,21 | 45,33 |
| 1 | ME8 | 211,45 | 76,88 | 14,06 | 63,74 | 75,90 | 12,16 | 6,00 | 100,04 | 2,82 | 52,69 |
| 1 | ME8 | 325,58 | 89,92 | 27,30 | 70,06 | 89,45 | 19,39 | 8,00 | 181,81 | 5,68 | 44,16 |
| 1 | ME9 | 269,25 | 84,01 | 20,03 | 69,89 | 91,29 | 21,40 | 6,00 | 126,67 | 3,52 | 52,95 |
| 1 | ME9 | 246,56 | 82,65 | 17,50 | 70,18 | 87,57 | 17,39 | 5,00 | 110,76 | 3,21 | 55,08 |
| 1 | ME9 | 339,96 | 85,86 | 19,21 | 76,74 | 91,93 | 15,19 | 6,00 | 164,06 | 4,18 | 51,74 |
| 1 | ME9 | 333,14 | 88,67 | 23,89 | 72,05 | 88,72 | 16,67 | 7,00 | 167,92 | 5,02 | 49,59 |
| 1 | ME9 | 259,24 | 83,49 | 21,27 | 69,06 | 89,83 | 20,77 | 6,00 | 127,36 | 3,65 | 50,87 |
| 1 | ME9 | 297,11 | 83,34 | 23,86 | 76,77 | 95,21 | 18,44 | 6,00 | 157,90 | 4,30 | 46,85 |
| 1 | ME9 | 423,60 | 97,20 | 22,16 | 86,77 | 97,77 | 11,00 | 7,00 | 217,69 | 4,69 | 48,61 |
| 1 | ME9 | 253,72 | 81,44 | 21,68 | 71,34 | 85,66 | 14,32 | 7,00 | 146,24 | 4,21 | 42,36 |
| 1 | ME9 | 365,06 | 88,35 | 20,23 | 77,25 | 94,38 | 17,13 | 7,00 | 170,74 | 4,87 | 53,23 |
| 1 | ME9 | 250,35 | 82,88 | 19,31 | 77,24 | 85,73 | 8,49 | 8,00 | 118,63 | 3,40 | 52,61 |
| 1 | ME10 | 167,49 | 70,98 | 15,56 | 61,13 | 72,23 | 11,10 | 6,00 | 70,70 | 3,60 | 57,79 |
| 1 | ME10 | 266,64 | 81,60 | 27,27 | 70,85 | 92,12 | 21,27 | 6,00 | 151,82 | 3,47 | 43,06 |
| 1 | ME10 | 191,46 | 72,64 | 18,50 | 64,93 | 81,47 | 16,54 | 6,00 | 89,43 | 3,06 | 53,29 |
| 1 | ME10 | 384,92 | 91,30 | 20,79 | 74,43 | 89,71 | 15,28 | 7,00 | 184,25 | 4,59 | 52,13 |
| 1 | ME10 | 241,10 | 77,35 | 20,52 | 69,02 | 82,81 | 13,79 | 6,00 | 120,20 | 4,03 | 50,15 |
| 1 | ME10 | 382,61 | 91,97 | 25,15 | 78,02 | 97,22 | 19,20 | 6,00 | 199,70 | 5,96 | 47,81 |
| 1 | ME10 | 317,42 | 87,70 | 21,25 | 75,45 | 95,10 | 19,65 | 6,00 | 176,00 | 4,43 | 44,55 |
| 1 | ME10 | 288,05 | 87,66 | 23,39 | 72,38 | 85,96 | 13,58 | 6,00 | 137,19 | 4,76 | 52,37 |
| 1 | ME10 | 234,97 | 80,06 | 19,72 | 63,11 | 84,47 | 21,36 | 7,00 | 120,20 | 2,77 | 48,84 |
| 1 | ME10 | 222,40 | 78,24 | 25,35 | 75,08 | 83,02 | 7,94 | 7,00 | 104,18 | 3,31 | 53,16 |
| 1 | ME11 | 261,58 | 83,29 | 18,61 | 70,12 | 89,90 | 19,78 | 8,00 | 124,57 | 3,99 | 52,38 |
| 1 | ME11 | 211,47 | 79,47 | 18,23 | 67,48 | 77,97 | 10,49 | 8,00 | 102,67 | 3,72 | 51,45 |
| 1 | ME11 | 168,37 | 72,51 | 18,65 | 61,50 | 81,07 | 19,57 | 8,00 | 92,72 | 2,58 | 44,93 |
| 1 | ME11 | 316,22 | 87,11 | 20,27 | 72,64 | 92,56 | 19,92 | 6,00 | 160,98 | 3,89 | 49,09 |
| 1 | ME11 | 186,52 | 76,45 | 18,28 | 61,61 | 78,55 | 16,94 | 7,00 | 98,68 | 4,05 | 47,09 |
| 1 | ME11 | 236,42 | 76,93 | 18,06 | 70,18 | 83,07 | 12,89 | 6,00 | 118,06 | 4,30 | 50,06 |
| 1 | ME11 | 155,64 | 66,37 | 21,69 | 61,00 | 79,72 | 18,72 | 7,00 | 90,73 | 4,18 | 41,71 |
| 1 | ME11 | 239,99 | 78,75 | 20,06 | 59,74 | 83,59 | 23,85 | 5,00 | 106,19 | 4,52 | 55,75 |
| 1 | ME11 | 256,75 | 79,70 | 22,30 | 70,71 | 88,27 | 17,56 | 7,00 | 118,90 | 3,70 | 53,69 |
| 1 | ME11 | 256,61 | 81,71 | 26,20 | 65,75 | 86,80 | 21,05 | 7,00 | 117,19 | 3,58 | 54,33 |
| 1 | ME12 | 279,35 | 81,30 | 17,26 | 67,85 | 82,89 | 15,04 | 9,00 | 108,19 | 2,61 | 61,27 |
| 1 | ME12 | 299,69 | 85,89 | 17,69 | 71,10 | 88,56 | 17,46 | 6,00 | 140,18 | 4,04 | 53,22 |
| 1 | ME12 | 320,44 | 87,06 | 18,23 | 74,99 | 88,26 | 13,27 | 9,00 | 123,71 | 2,59 | 61,39 |
| 1 | ME12 | 271,35 | 87,54 | 18,39 | 77,21 | 83,55 | 6,34 | 7,00 | 130,99 | 4,63 | 51,73 |
| 1 | ME12 | 138,67 | 77,47 | 18,48 | 55,91 | 70,02 | 14,11 | 7,00 | 77,70 | 3,57 | 43,97 |
| 1 | ME12 | 224,63 | 78,54 | 22,31 | 68,26 | 80,60 | 12,34 | 7,00 | 119,87 | 4,39 | 46,64 |
| 1 | ME12 | 495,84 | 103,45 | 21,29 | 82,55 | 101,47 | 18,92 | 9,00 | 188,32 | 3,61 | 62,02 |
| 1 | ME12 | 243,35 | 76,52 | 22,15 | 67,75 | 86,27 | 18,52 | 7,00 | 108,97 | 3,58 | 55,22 |
| 1 | ME12 | 485,30 | 100,32 | 18,24 | 88,53 | 99,72 | 11,19 | 9,00 | 209,48 | 3,82 | 56,83 |
| 1 | ME12 | 155,23 | 68,10 | 17,61 | 57,61 | 75,20 | 17,59 | 7,00 | 78,08 | 3,45 | 49,70 |
| 1 | ME13 | 334,57 | 88,69 | 19,92 | 76,17 | 94,87 | 18,70 | 6,00 | 152,08 | 4,87 | 54,54 |
| 1 | ME13 | 262,40 | 78,75 | 16,21 | 68,55 | 85,82 | 17,27 | 6,00 | 125,96 | 5,64 | 52,00 |
| 1 | ME13 | 342,15 | 90,54 | 21,29 | 75,89 | 90,21 | 14,32 | 6,00 | 158,32 | 4,37 | 53,73 |
| 1 | ME13 | 213,07 | 73,35 | 22,96 | 62,88 | 79,01 | 16,13 | 6,00 | 100,98 | 4,70 | 52,61 |
| 1 | ME13 | 406,85 | 91,45 | 21,90 | 77,90 | 97,58 | 19,68 | 7,00 | 187,51 | 6,32 | 53,91 |
| 1 | ME13 | 436,36 | 95,75 | 17,97 | 83,61 | 93,19 | 9,58 | 7,00 | 207,40 | 7,34 | 52,47 |
| 1 | ME13 | 279,80 | 82,39 | 19,06 | 69,15 | 93,33 | 24,18 | 6,00 | 118,07 | 5,28 | 57,80 |
| 1 | ME13 | 233,06 | 74,76 | 19,93 | 68,23 | 83,94 | 15,71 | 6,00 | 101,43 | 3,16 | 56,48 |
| 1 | ME13 | 293,79 | 79,28 | 20,14 | 72,66 | 83,40 | 10,74 | 6,00 | 141,26 | 4,77 | 51,92 |
| 1 | ME13 | 401,55 | 94,83 | 18,40 | 80,90 | 97,37 | 16,47 | 6,00 | 155,82 | 4,72 | 61,20 |
| 1 | ME14 | 401,22 | 92,59 | 23,41 | 80,44 | 98,05 | 17,61 | 7,00 | 179,47 | 4,87 | 55,27 |
| 1 | ME14 | 425,12 | 96,39 | 25,36 | 83,89 | 95,91 | 12,02 | 6,00 | 178,92 | 6,34 | 57,91 |
| 1 | ME14 | 325,68 | 84,62 | 25,29 | 76,20 | 90,27 | 14,07 | 6,00 | 154,75 | 5,58 | 52,48 |
| 1 | ME14 | 296,33 | 84,89 | 21,50 | 72,58 | 87,11 | 14,53 | 6,00 | 152,00 | 6,79 | 48,71 |
| 1 | ME14 | 399,00 | 94,88 | 23,97 | 77,08 | 88,43 | 11,35 | 6,00 | 189,85 | 6,12 | 52,42 |
| 1 | ME14 | 282,52 | 86,37 | 25,68 | 72,13 | 88,27 | 16,14 | 6,00 | 154,08 | 6,79 | 45,46 |
| 1 | ME14 | 393,75 | 101,80 | 27,91 | 83,79 | 99,12 | 15,33 | 8,00 | 162,79 | 4,95 | 58,66 |
| 1 | ME14 | 304,44 | 82,24 | 26,20 | 75,02 | 91,70 | 16,68 | 6,00 | 132,00 | 5,78 | 56,64 |
| 1 | ME14 | 418,99 | 90,83 | 30,97 | 81,01 | 97,59 | 16,58 | 7,00 | 205,34 | 6,39 | 50,99 |
| 1 | ME14 | 331,02 | 87,58 | 29,11 | 74,74 | 87,06 | 12,32 | 6,00 | 176,40 | 5,84 | 46,71 |
| 1 | ME16 | 392,49 | 92,89 | 23,43 | 84,73 | 106,96 | 22,23 | 5,00 | 147,39 | 4,52 | 62,45 |
| 1 | ME16 | 276,13 | 82,14 | 27,98 | 70,41 | 90,28 | 19,87 | 6,00 | 129,66 | 5,19 | 53,04 |
| 1 | ME16 | 392,61 | 93,47 | 20,53 | 86,27 | 103,18 | 16,91 | 6,00 | 166,36 | 5,55 | 57,63 |
| 1 | ME16 | 386,63 | 89,69 | 24,42 | 86,06 | 94,29 | 8,23 | 6,00 | 179,41 | 4,56 | 53,60 |
| 1 | ME16 | 324,57 | 87,27 | 16,99 | 76,88 | 84,35 | 7,47 | 6,00 | 122,99 | 3,85 | 62,11 |
| 1 | ME16 | 331,25 | 84,53 | 19,17 | 80,69 | 97,49 | 16,80 | 6,00 | 137,69 | 4,03 | 58,43 |
| 1 | ME16 | 470,52 | 96,23 | 23,25 | 95,02 | 104,66 | 9,64 | 6,00 | 181,04 | 4,54 | 61,52 |
| 1 | ME16 | 237,67 | 78,48 | 24,95 | 63,12 | 86,57 | 23,45 | 5,00 | 123,45 | 4,98 | 48,06 |
| 1 | ME16 | 311,63 | 86,56 | 18,65 | 77,11 | 85,36 | 8,25 | 6,00 | 130,18 | 4,16 | 58,23 |
| 1 | ME16 | 374,44 | 90,21 | 19,50 | 84,45 | 101,45 | 17,00 | 6,00 | 162,10 | 4,58 | 56,71 |
| 1 | ME17 | 319,06 | 84,17 | 27,43 | 76,53 | 94,10 | 17,57 | 6,00 | 161,17 | 5,61 | 49,49 |
| 1 | ME17 | 200,60 | 74,50 | 27,82 | 63,34 | 80,12 | 16,78 | 5,00 | 94,18 | 4,76 | 53,05 |
| 1 | ME17 | 388,40 | 94,38 | 29,41 | 79,59 | 96,81 | 17,22 | 7,00 | 218,45 | 6,98 | 43,76 |
| 1 | ME17 | 367,70 | 91,39 | 22,38 | 77,05 | 92,78 | 15,73 | 7,00 | 192,49 | 5,36 | 47,65 |
| 1 | ME17 | 349,40 | 87,79 | 20,06 | 80,48 | 95,74 | 15,26 | 7,00 | 143,98 | 3,87 | 58,79 |
| 1 | ME17 | 292,24 | 86,13 | 31,13 | 72,85 | 79,11 | 6,26 | 5,00 | 183,09 | 6,73 | 37,35 |
| 1 | ME17 | 365,84 | 91,27 | 23,23 | 80,21 | 97,87 | 17,66 | 6,00 | 187,43 | 4,74 | 48,77 |
| 1 | ME17 | 319,00 | 88,97 | 23,38 | 76,54 | 90,66 | 14,12 | 6,00 | 168,46 | 7,37 | 47,19 |
| 1 | ME17 | 234,70 | 75,86 | 27,23 | 68,11 | 85,47 | 17,36 | 5,00 | 134,30 | 4,40 | 42,78 |
| 1 | ME17 | 216,24 | 74,68 | 26,19 | 66,38 | 85,45 | 19,07 | 6,00 | 129,93 | 7,54 | 39,91 |
| 1 | ME18 | 213,31 | 74,38 | 22,47 | 69,47 | 86,50 | 17,03 | 6,00 | 114,44 | 5,55 | 46,35 |
| 1 | ME18 | 231,35 | 75,71 | 19,73 | 69,17 | 81,34 | 12,17 | 6,00 | 111,92 | 5,36 | 51,62 |
| 1 | ME18 | 310,59 | 86,97 | 25,66 | 76,67 | 95,56 | 18,89 | 6,00 | 128,66 | 3,99 | 58,58 |
| 1 | ME18 | 272,72 | 82,49 | 18,25 | 70,27 | 83,72 | 13,45 | 6,00 | 110,66 | 3,72 | 59,42 |
| 1 | ME18 | 198,33 | 75,10 | 18,15 | 74,14 | 80,30 | 6,16 | 6,00 | 103,90 | 4,86 | 47,61 |
| 1 | ME18 | 237,27 | 72,37 | 20,72 | 70,95 | 89,70 | 18,75 | 6,00 | 117,85 | 4,70 | 50,33 |
| 1 | ME18 | 231,03 | 77,37 | 21,40 | 77,04 | 88,01 | 10,97 | 7,00 | 113,58 | 5,68 | 50,84 |
| 1 | ME18 | 497,87 | 74,09 | 28,45 | 89,86 | 100,17 | 10,31 | 8,00 | 251,88 | 5,09 | 49,41 |
| 1 | ME18 | 355,40 | 88,05 | 19,16 | 78,73 | 90,67 | 11,94 | 7,00 | 147,21 | 4,63 | 58,58 |
| 1 | ME18 | 270,09 | 84,88 | 22,00 | 76,77 | 86,65 | 9,88 | 8,00 | 144,83 | 5,68 | 46,38 |
| 1 | ME19 | 204,45 | 72,24 | 17,78 | 67,68 | 80,29 | 12,61 | 5,00 | 106,04 | 5,17 | 48,13 |
| 1 | ME19 | 197,17 | 72,43 | 18,89 | 61,83 | 75,45 | 13,62 | 6,00 | 83,90 | 2,35 | 57,45 |
| 1 | ME19 | 280,74 | 82,35 | 25,70 | 78,11 | 90,88 | 12,77 | 7,00 | 126,95 | 3,21 | 54,78 |
| 1 | ME19 | 331,78 | 88,30 | 16,98 | 75,80 | 89,43 | 13,63 | 7,00 | 137,15 | 5,13 | 58,66 |
| 1 | ME19 | 495,65 | 102,59 | 25,24 | 87,69 | 106,62 | 18,93 | 7,00 | 195,73 | 4,49 | 60,51 |
| 1 | ME19 | 331,65 | 90,11 | 23,88 | 77,77 | 89,56 | 11,79 | 7,00 | 172,43 | 5,76 | 48,01 |
| 1 | ME19 | 307,83 | 84,06 | 17,58 | 77,08 | 89,46 | 12,38 | 6,00 | 136,59 | 2,69 | 55,63 |
| 1 | ME19 | 308,82 | 89,86 | 20,46 | 80,70 | 96,70 | 16,00 | 5,00 | 165,16 | 4,86 | 46,52 |
| 1 | ME19 | 365,86 | 80,68 | 20,36 | 72,48 | 90,04 | 17,56 | 6,00 | 166,94 | 5,48 | 54,37 |
| 1 | ME19 | 281,78 | 85,69 | 18,79 | 73,91 | 88,14 | 14,23 | 6,00 | 132,55 | 4,50 | 52,96 |
| 1 | ME20 | 351,69 | 90,10 | 21,89 | 81,12 | 98,08 | 16,96 | 7,00 | 152,96 | 4,77 | 56,51 |
| 1 | ME20 | 477,10 | 85,89 | 19,80 | 84,02 | 96,69 | 12,67 | 8,00 | 210,83 | 4,64 | 55,81 |
| 1 | ME20 | 317,43 | 88,58 | 21,38 | 76,14 | 89,05 | 12,91 | 6,00 | 127,32 | 4,55 | 59,89 |
| 1 | ME20 | 259,14 | 95,99 | 24,42 | 81,81 | 98,68 | 16,87 | 7,00 | 146,33 | 4,82 | 43,53 |
| 1 | ME20 | 405,17 | 103,85 | 24,08 | 69,89 | 86,38 | 16,49 | 6,00 | 115,10 | 4,25 | 71,59 |
| 1 | ME20 | 395,98 | 93,84 | 24,12 | 83,90 | 102,59 | 18,69 | 7,00 | 183,17 | 5,19 | 53,74 |
| 1 | ME20 | 345,38 | 84,48 | 21,06 | 76,16 | 88,09 | 11,93 | 7,00 | 152,13 | 4,81 | 55,95 |
| 1 | ME20 | 501,60 | 85,07 | 28,85 | 84,50 | 87,51 | 3,01 | 7,00 | 196,71 | 3,83 | 60,78 |
| 1 | ME20 | 258,91 | 86,39 | 20,06 | 63,06 | 88,65 | 25,59 | 7,00 | 121,83 | 4,22 | 52,95 |
| 1 | ME20 | 257,28 | 84,12 | 20,24 | 73,41 | 81,12 | 7,71 | 7,00 | 115,89 | 4,50 | 54,96 |
| 1 | ME21 | 283,15 | 78,39 | 21,48 | 62,06 | 84,02 | 21,96 | 7,00 | 138,82 | 4,69 | 50,97 |
| 1 | ME21 | 245,02 | 80,64 | 20,08 | 73,03 | 83,87 | 10,84 | 7,00 | 130,81 | 5,64 | 46,61 |
| 1 | ME21 | 273,42 | 83,63 | 20,19 | 77,64 | 92,37 | 14,73 | 7,00 | 124,08 | 5,45 | 54,62 |
| 1 | ME21 | 206,59 | 74,58 | 28,69 | 77,65 | 81,24 | 3,59 | 7,00 | 98,68 | 4,86 | 52,23 |
| 1 | ME21 | 386,27 | 80,99 | 11,94 | 72,94 | 92,87 | 19,93 | 7,00 | 196,18 | 5,44 | 49,21 |
| 1 | ME21 | 237,31 | 68,87 | 10,92 | 61,07 | 81,93 | 20,86 | 6,00 | 116,81 | 5,10 | 50,78 |
| 1 | ME21 | 231,48 | 66,14 | 10,54 | 59,48 | 75,24 | 15,76 | 7,00 | 104,31 | 3,83 | 54,94 |
| 1 | ME21 | 249,04 | 67,45 | 10,03 | 59,42 | 69,56 | 10,14 | 6,00 | 127,86 | 4,47 | 48,66 |
| 1 | ME21 | 207,54 | 73,18 | 10,92 | 53,48 | 73,08 | 19,60 | 6,00 | 102,70 | 4,52 | 50,52 |
| 1 | ME21 | 307,28 | 73,98 | 9,09 | 66,93 | 84,02 | 17,09 | 6,00 | 154,14 | 4,53 | 49,84 |
| 1 | MA1 | 276,93 | 84,02 | 17,94 | 70,37 | 79,27 | 8,90 | 6,00 | 125,22 | 4,77 | 54,78 |
| 1 | MA1 | 401,47 | 93,50 | 19,75 | 82,02 | 96,93 | 14,91 | 6,00 | 196,70 | 4,86 | 51,01 |
| 1 | MA1 | 333,56 | 88,26 | 23,15 | 74,01 | 93,11 | 19,10 | 7,00 | 154,25 | 5,11 | 53,76 |
| 1 | MA1 | 306,28 | 84,50 | 21,43 | 71,96 | 87,96 | 16,00 | 6,00 | 145,89 | 5,26 | 52,37 |
| 1 | MA1 | 175,05 | 70,73 | 17,21 | 61,88 | 73,49 | 11,61 | 6,00 | 82,13 | 4,08 | 53,08 |
| 1 | MA1 | 179,32 | 68,91 | 16,34 | 60,40 | 72,23 | 11,83 | 5,00 | 82,15 | 4,62 | 54,19 |
| 1 | MA1 | 365,66 | 90,77 | 22,38 | 73,14 | 89,90 | 16,76 | 7,00 | 182,02 | 5,05 | 50,22 |
| 1 | MA1 | 502,19 | 101,02 | 20,43 | 86,26 | 96,30 | 10,04 | 6,00 | 210,60 | 4,84 | 58,06 |
| 1 | MA1 | 171,53 | 72,07 | 22,49 | 59,03 | 67,61 | 8,58 | 7,00 | 94,49 | 4,50 | 44,91 |
| 1 | MA1 | 326,78 | 87,63 | 17,11 | 76,59 | 91,48 | 14,89 | 7,00 | 138,17 | 3,41 | 57,72 |
| 1 | MA2 | 205,10 | 76,52 | 21,34 | 64,14 | 84,99 | 20,85 | 6,00 | 95,50 | 3,63 | 53,44 |
| 1 | MA2 | 388,65 | 91,18 | 17,73 | 85,08 | 96,09 | 11,01 | 7,00 | 167,01 | 4,33 | 57,03 |
| 1 | MA2 | 247,67 | 80,18 | 17,59 | 70,02 | 87,20 | 17,18 | 6,00 | 118,67 | 3,55 | 52,09 |
| 1 | MA2 | 583,75 | 107,58 | 23,37 | 92,43 | 109,61 | 17,18 | 7,00 | 258,10 | 5,36 | 55,79 |
| 1 | MA2 | 388,21 | 91,32 | 22,14 | 83,28 | 95,37 | 12,09 | 6,00 | 170,14 | 3,60 | 56,17 |
| 1 | MA2 | 249,13 | 76,51 | 22,03 | 71,24 | 82,84 | 11,60 | 5,00 | 119,89 | 4,03 | 51,88 |
| 1 | MA2 | 329,11 | 85,96 | 21,65 | 77,31 | 94,81 | 17,50 | 7,00 | 173,37 | 5,21 | 47,32 |
| 1 | MA2 | 231,35 | 75,99 | 21,40 | 69,06 | 78,05 | 8,99 | 5,00 | 92,11 | 2,68 | 60,19 |
| 1 | MA2 | 563,90 | 108,61 | 21,71 | 99,88 | 103,67 | 3,79 | 6,00 | 265,39 | 5,30 | 52,94 |
| 1 | MA2 | 269,36 | 83,74 | 21,60 | 71,14 | 90,37 | 19,23 | 6,00 | 112,12 | 3,38 | 58,38 |
| 1 | MA3 | 306,58 | 88,03 | 23,41 | 69,78 | 88,50 | 18,72 | 7,00 | 159,70 | 4,91 | 47,91 |
| 1 | MA3 | 350,72 | 88,54 | 20,16 | 76,50 | 94,65 | 18,15 | 8,00 | 145,75 | 3,50 | 58,44 |
| 1 | MA3 | 304,59 | 85,61 | 20,85 | 71,58 | 88,65 | 17,07 | 7,00 | 140,51 | 5,28 | 53,87 |
| 1 | MA3 | 370,32 | 93,72 | 25,49 | 79,38 | 94,31 | 14,93 | 7,00 | 196,07 | 5,20 | 47,05 |
| 1 | MA3 | 224,66 | 77,19 | 18,26 | 66,22 | 80,83 | 14,61 | 7,00 | 104,29 | 4,02 | 53,58 |
| 1 | MA3 | 297,12 | 87,18 | 23,96 | 73,41 | 87,93 | 14,52 | 7,00 | 132,99 | 4,85 | 55,24 |
| 1 | MA3 | 235,23 | 82,34 | 19,10 | 69,75 | 90,66 | 20,91 | 6,00 | 107,03 | 3,23 | 54,50 |
| 1 | MA3 | 341,65 | 89,40 | 20,08 | 70,92 | 89,34 | 18,42 | 8,00 | 171,80 | 5,97 | 49,71 |
| 1 | MA3 | 446,59 | 97,91 | 22,54 | 83,74 | 97,68 | 13,94 | 6,00 | 189,15 | 4,56 | 57,65 |
| 1 | MA3 | 248,75 | 78,14 | 17,30 | 79,77 | 95,40 | 15,63 | 7,00 | 108,50 | 3,45 | 56,38 |
| 1 | MA4 | 413,86 | 96,80 | 18,09 | 79,91 | 98,11 | 18,20 | 8,00 | 193,22 | 5,50 | 53,31 |
| 1 | MA4 | 371,85 | 88,92 | 17,41 | 78,24 | 87,96 | 9,72 | 8,00 | 155,16 | 4,99 | 58,27 |
| 1 | MA4 | 257,35 | 81,52 | 17,08 | 70,63 | 84,56 | 13,93 | 7,00 | 124,30 | 4,83 | 51,70 |
| 1 | MA4 | 230,31 | 76,88 | 18,50 | 66,24 | 82,86 | 16,62 | 8,00 | 116,42 | 4,56 | 49,45 |
| 1 | MA4 | 316,27 | 84,40 | 22,26 | 73,35 | 89,87 | 16,52 | 5,00 | 160,60 | 5,39 | 49,22 |
| 1 | MA4 | 363,81 | 88,79 | 19,86 | 80,36 | 100,16 | 19,80 | 7,00 | 170,61 | 4,49 | 53,10 |
| 1 | MA4 | 172,53 | 69,45 | 17,66 | 59,73 | 74,09 | 14,36 | 6,00 | 80,34 | 3,39 | 53,43 |
| 1 | MA4 | 430,37 | 95,87 | 21,95 | 79,44 | 96,48 | 17,04 | 7,00 | 211,07 | 6,06 | 50,96 |
| 1 | MA4 | 383,34 | 87,42 | 22,82 | 81,05 | 98,29 | 17,24 | 6,00 | 155,53 | 3,24 | 59,43 |
| 1 | MA4 | 232,87 | 78,05 | 16,62 | 69,58 | 81,90 | 12,32 | 7,00 | 101,27 | 4,18 | 56,51 |
| 1 | MA5 | 375,63 | 88,22 | 23,77 | 81,89 | 93,34 | 11,45 | 7,00 | 159,10 | 4,45 | 57,64 |
| 1 | MA5 | 207,75 | 71,81 | 21,17 | 61,32 | 81,63 | 20,31 | 5,00 | 115,56 | 6,18 | 44,38 |
| 1 | MA5 | 339,63 | 89,93 | 24,97 | 76,13 | 93,90 | 17,77 | 6,00 | 159,14 | 5,41 | 53,14 |
| 1 | MA5 | 252,01 | 79,24 | 20,23 | 65,65 | 76,36 | 10,71 | 6,00 | 119,57 | 2,95 | 52,55 |
| 1 | MA5 | 310,24 | 82,80 | 21,49 | 69,15 | 82,75 | 13,60 | 6,00 | 161,09 | 5,15 | 48,08 |
| 1 | MA5 | 496,79 | 97,26 | 19,66 | 84,22 | 95,81 | 11,59 | 7,00 | 129,82 | 6,22 | 73,87 |
| 1 | MA5 | 276,50 | 81,89 | 23,67 | 68,69 | 83,95 | 15,26 | 7,00 | 125,05 | 4,38 | 54,77 |
| 1 | MA5 | 280,24 | 78,88 | 18,69 | 70,55 | 77,83 | 7,28 | 7,00 | 109,31 | 4,57 | 60,99 |
| 1 | MA5 | 281,30 | 81,72 | 17,49 | 72,23 | 91,07 | 18,84 | 7,00 | 130,55 | 4,07 | 53,59 |
| 1 | MA5 | 286,35 | 81,66 | 17,29 | 72,45 | 89,08 | 16,63 | 7,00 | 127,57 | 4,09 | 55,45 |
| 1 | BA1 | 468,40 | 101,85 | 24,10 | 79,97 | 96,87 | 16,90 | 9,00 | 112,20 | 3,89 | 76,05 |
| 1 | BA1 | 272,02 | 83,05 | 23,24 | 69,19 | 89,03 | 19,84 | 6,00 | 130,71 | 2,68 | 51,95 |
| 1 | BA1 | 227,06 | 77,69 | 22,97 | 65,95 | 87,34 | 21,39 | 5,00 | 115,78 | 3,94 | 49,01 |
| 1 | BA1 | 426,92 | 97,44 | 25,38 | 80,42 | 99,97 | 19,55 | 6,00 | 224,53 | 5,48 | 47,41 |
| 1 | BA1 | 370,58 | 90,84 | 24,22 | 80,87 | 93,02 | 12,15 | 6,00 | 185,92 | 3,62 | 49,83 |
| 1 | BA1 | 241,94 | 82,50 | 27,19 | 67,11 | 87,71 | 20,60 | 5,00 | 126,90 | 3,62 | 47,55 |
| 1 | BA1 | 350,20 | 91,86 | 23,41 | 77,08 | 95,59 | 18,51 | 7,00 | 202,99 | 5,68 | 42,04 |
| 1 | BA1 | 252,70 | 79,47 | 26,69 | 67,08 | 87,08 | 20,00 | 7,00 | 115,65 | 3,61 | 54,23 |
| 1 | BA1 | 461,33 | 96,32 | 20,39 | 84,92 | 100,53 | 15,61 | 6,00 | 213,78 | 3,52 | 53,66 |
| 1 | BA1 | 487,48 | 101,93 | 20,77 | 88,34 | 105,28 | 16,94 | 7,00 | 221,77 | 4,01 | 54,51 |
| 1 | VA1 | 268,05 | 81,82 | 24,79 | 71,89 | 91,40 | 19,51 | 5,00 | 153,45 | 5,38 | 42,75 |
| 1 | VA1 | 362,59 | 91,11 | 19,94 | 78,76 | 88,70 | 9,94 | 6,00 | 193,28 | 5,31 | 46,69 |
| 1 | VA1 | 326,51 | 85,35 | 21,92 | 74,27 | 93,34 | 19,07 | 6,00 | 184,17 | 6,59 | 43,59 |
| 1 | VA1 | 303,40 | 86,58 | 21,68 | 73,12 | 85,74 | 12,62 | 6,00 | 142,20 | 3,91 | 53,13 |
| 1 | VA1 | 391,05 | 92,09 | 23,82 | 82,18 | 97,51 | 15,33 | 6,00 | 222,30 | 4,42 | 43,15 |
| 1 | VA1 | 471,90 | 89,96 | 20,24 | 79,29 | 92,18 | 12,89 | 7,00 | 233,31 | 5,12 | 50,56 |
| 1 | VA1 | 232,47 | 75,61 | 23,16 | 67,28 | 82,79 | 15,51 | 5,00 | 139,05 | 4,79 | 40,19 |
| 1 | VA1 | 231,65 | 78,12 | 24,10 | 69,22 | 84,18 | 14,96 | 6,00 | 116,60 | 2,56 | 49,67 |
| 1 | VA1 | 308,80 | 82,30 | 25,76 | 72,65 | 91,92 | 19,27 | 6,00 | 181,22 | 3,67 | 41,31 |
| 1 | VA1 | 470,29 | 96,77 | 24,80 | 88,32 | 102,10 | 13,78 | 7,00 | 227,62 | 5,00 | 51,60 |
| 1 | MC1 | 266,22 | 78,48 | 25,64 | 72,43 | 90,63 | 18,20 | 4,00 | 143,01 | 2,46 | 46,28 |
| 1 | MC1 | 278,82 | 83,40 | 21,44 | 73,08 | 92,74 | 19,66 | 6,00 | 141,86 | 3,80 | 49,12 |
| 1 | MC1 | 275,61 | 80,11 | 22,54 | 74,95 | 91,94 | 16,99 | 6,00 | 134,05 | 5,74 | 51,36 |
| 1 | MC1 | 451,83 | 94,73 | 29,51 | 82,97 | 100,73 | 17,76 | 6,00 | 249,90 | 6,21 | 44,69 |
| 1 | MC1 | 630,50 | 109,63 | 30,80 | 93,32 | 111,14 | 17,82 | 6,00 | 346,99 | 6,06 | 44,97 |
| 1 | MC1 | 321,56 | 92,50 | 25,45 | 79,56 | 98,12 | 18,56 | 6,00 | 156,80 | 5,98 | 51,24 |
| 1 | MC1 | 298,36 | 85,56 | 23,96 | 77,78 | 95,44 | 17,66 | 6,00 | 149,32 | 4,89 | 49,95 |
| 1 | MC1 | 278,14 | 84,15 | 22,16 | 75,48 | 95,06 | 19,58 | 6,00 | 139,48 | 4,15 | 49,85 |
| 1 | MC1 | 304,82 | 88,87 | 23,89 | 78,96 | 97,54 | 18,58 | 6,00 | 147,36 | 5,63 | 51,66 |
| 1 | MC1 | 409,35 | 91,45 | 24,86 | 80,47 | 100,52 | 20,05 | 6,00 | 189,60 | 6,05 | 53,68 |
| 1 | MO2 | 459,92 | 98,18 | 19,66 | 87,96 | 104,33 | 16,37 | 6,00 | 191,05 | 4,71 | 58,46 |
| 1 | MO2 | 233,65 | 80,03 | 21,90 | 68,98 | 78,52 | 9,54 | 6,00 | 113,40 | 4,68 | 51,47 |
| 1 | MO2 | 249,00 | 80,82 | 19,43 | 69,17 | 78,36 | 9,19 | 5,00 | 140,14 | 4,38 | 43,72 |
| 1 | MO2 | 255,32 | 79,73 | 24,12 | 67,91 | 85,09 | 17,18 | 7,00 | 122,90 | 4,81 | 51,86 |
| 1 | MO2 | 174,12 | 69,42 | 22,53 | 61,02 | 76,58 | 15,56 | 6,00 | 85,43 | 4,39 | 50,94 |
| 1 | MO2 | 159,17 | 65,96 | 19,36 | 64,89 | 80,12 | 15,23 | 5,00 | 92,25 | 4,01 | 42,04 |
| 1 | MO2 | 146,80 | 66,22 | 15,66 | 58,63 | 65,92 | 7,29 | 6,00 | 67,53 | 4,71 | 54,00 |
| 1 | MO2 | 243,00 | 74,26 | 20,45 | 73,18 | 90,12 | 16,94 | 6,00 | 152,00 | 4,23 | 37,45 |
| 1 | MO2 | 306,04 | 81,43 | 19,19 | 74,50 | 90,57 | 16,07 | 6,00 | 137,42 | 4,45 | 55,10 |
| 1 | MO2 | 269,80 | 81,08 | 20,10 | 71,72 | 87,47 | 15,75 | 6,00 | 127,02 | 5,23 | 52,92 |
| 1 | MO3 | 272,91 | 102,94 | 25,98 | 88,92 | 104,52 | 15,60 | 5,00 | 141,00 | 5,57 | 48,33 |
| 1 | MO3 | 344,89 | 88,59 | 20,12 | 74,57 | 88,62 | 14,05 | 6,00 | 142,23 | 3,39 | 58,76 |
| 1 | MO3 | 432,35 | 99,50 | 20,56 | 84,56 | 90,06 | 5,50 | 8,00 | 197,57 | 5,40 | 54,30 |
| 1 | MO3 | 369,33 | 89,62 | 23,39 | 77,88 | 92,14 | 14,26 | 6,00 | 198,81 | 5,09 | 46,17 |
| 1 | MO3 | 260,61 | 75,17 | 23,60 | 68,92 | 82,61 | 13,69 | 7,00 | 115,44 | 3,93 | 55,70 |
| 1 | MO3 | 432,34 | 90,88 | 21,92 | 83,85 | 98,59 | 14,74 | 6,00 | 136,25 | 5,26 | 68,49 |
| 1 | MO3 | 361,33 | 90,66 | 22,99 | 80,78 | 93,66 | 12,88 | 6,00 | 170,03 | 4,98 | 52,94 |
| 1 | MO3 | 273,63 | 81,78 | 21,89 | 72,24 | 87,01 | 14,77 | 6,00 | 135,07 | 4,43 | 50,64 |
| 1 | MO3 | 335,81 | 88,85 | 17,99 | 76,87 | 90,13 | 13,26 | 6,00 | 144,39 | 4,94 | 57,00 |
| 1 | MO3 | 270,37 | 83,90 | 17,45 | 71,30 | 90,43 | 19,13 | 6,00 | 132,00 | 2,89 | 51,18 |
| 1 | MO4 | 225,37 | 78,29 | 22,78 | 62,53 | 82,78 | 20,25 | 6,00 | 100,18 | 3,86 | 55,55 |
| 1 | MO4 | 324,35 | 87,00 | 20,48 | 73,99 | 94,41 | 20,42 | 6,00 | 103,68 | 3,92 | 68,03 |
| 1 | MO4 | 381,18 | 90,54 | 22,54 | 78,68 | 96,17 | 17,49 | 5,00 | 91,52 | 4,44 | 75,99 |
| 1 | MO4 | 477,44 | 95,01 | 20,63 | 85,84 | 101,70 | 15,86 | 7,00 | 170,94 | 3,83 | 64,20 |
| 1 | MO4 | 465,18 | 92,19 | 27,41 | 85,05 | 104,61 | 19,56 | 7,00 | 171,60 | 5,25 | 63,11 |
| 1 | MO4 | 357,09 | 86,89 | 22,06 | 79,01 | 97,88 | 18,87 | 6,00 | 212,35 | 6,25 | 40,53 |
| 1 | MO4 | 233,44 | 76,33 | 22,09 | 65,44 | 82,58 | 17,14 | 8,00 | 97,87 | 3,75 | 58,07 |
| 1 | MO4 | 283,52 | 81,74 | 22,39 | 72,97 | 91,37 | 18,40 | 6,00 | 140,70 | 3,64 | 50,37 |
| 1 | MO4 | 204,02 | 76,35 | 23,98 | 63,22 | 84,01 | 20,79 | 6,00 | 119,82 | 3,54 | 41,27 |
| 1 | MO4 | 161,18 | 64,93 | 20,93 | 56,95 | 78,83 | 21,88 | 5,00 | 96,43 | 3,68 | 40,17 |
| 1 | MO5 | 312,63 | 88,20 | 17,76 | 73,77 | 89,50 | 15,73 | 7,00 | 156,98 | 4,29 | 49,79 |
| 1 | MO5 | 486,58 | 99,75 | 31,88 | 84,49 | 95,89 | 11,40 | 7,00 | 223,07 | 4,13 | 54,16 |
| 1 | MO5 | 354,79 | 88,61 | 22,92 | 77,18 | 91,47 | 14,29 | 8,00 | 142,81 | 2,93 | 59,75 |
| 1 | MO5 | 300,34 | 82,32 | 19,14 | 71,42 | 93,32 | 21,90 | 7,00 | 123,77 | 3,30 | 58,79 |
| 1 | MO5 | 451,74 | 98,37 | 23,57 | 79,32 | 93,06 | 13,74 | 8,00 | 197,60 | 4,24 | 56,26 |
| 1 | MO5 | 309,28 | 83,16 | 19,25 | 71,56 | 85,95 | 14,39 | 6,00 | 146,71 | 3,32 | 52,56 |
| 1 | MO5 | 488,07 | 101,04 | 20,31 | 82,12 | 93,92 | 11,80 | 8,00 | 210,45 | 3,93 | 56,88 |
| 1 | MO5 | 356,36 | 90,24 | 20,06 | 75,36 | 91,94 | 16,58 | 8,00 | 141,98 | 4,97 | 60,16 |
| 1 | MO5 | 369,27 | 90,25 | 22,82 | 77,35 | 88,17 | 10,82 | 7,00 | 163,98 | 3,88 | 55,59 |
| 1 | MO5 | 405,43 | 96,26 | 29,15 | 81,21 | 95,89 | 14,68 | 6,00 | 208,60 | 4,71 | 48,55 |
| 1 | MO6 | 196,87 | 75,81 | 21,82 | 64,09 | 83,95 | 19,86 | 6,00 | 87,98 | 2,53 | 55,31 |
| 1 | MO6 | 263,60 | 80,07 | 18,93 | 68,97 | 86,98 | 18,01 | 7,00 | 108,98 | 3,44 | 58,66 |
| 1 | MO6 | 467,72 | 105,46 | 21,30 | 77,54 | 94,19 | 16,65 | 9,00 | 221,73 | 4,29 | 52,59 |
| 1 | MO6 | 489,49 | 104,47 | 23,56 | 80,88 | 99,34 | 18,46 | 7,00 | 247,11 | 4,62 | 49,52 |
| 1 | MO6 | 241,05 | 77,00 | 20,92 | 69,01 | 83,95 | 14,94 | 6,00 | 113,25 | 3,35 | 53,02 |
| 1 | MO6 | 255,19 | 80,49 | 21,86 | 64,47 | 81,68 | 17,21 | 6,00 | 127,02 | 4,18 | 50,23 |
| 1 | MO6 | 293,85 | 82,06 | 23,01 | 72,52 | 94,14 | 21,62 | 6,00 | 138,98 | 2,94 | 52,70 |
| 1 | MO6 | 186,63 | 71,62 | 17,37 | 61,33 | 81,67 | 20,34 | 6,00 | 77,08 | 2,84 | 58,70 |
| 1 | MO6 | 324,51 | 89,36 | 22,25 | 72,25 | 89,88 | 17,63 | 6,00 | 168,79 | 5,35 | 47,99 |
| 1 | MO6 | 197,76 | 73,59 | 18,45 | 64,82 | 77,79 | 12,97 | 6,00 | 101,30 | 3,00 | 48,78 |
| 1 | AB1 | 301,15 | 81,22 | 21,19 | 69,16 | 90,69 | 21,53 | 6,00 | 152,97 | 2,56 | 49,20 |
| 1 | AB1 | 352,78 | 95,03 | 20,63 | 68,28 | 97,72 | 29,44 | 6,00 | 145,12 | 4,93 | 58,86 |
| 1 | AB1 | 235,73 | 77,20 | 21,23 | 66,32 | 88,96 | 22,64 | 7,00 | 147,53 | 5,75 | 37,42 |
| 1 | AB1 | 399,80 | 91,86 | 17,22 | 81,44 | 100,33 | 18,89 | 7,00 | 194,14 | 4,39 | 51,44 |
| 1 | AB1 | 207,65 | 77,70 | 17,60 | 60,79 | 81,82 | 21,03 | 6,00 | 118,45 | 5,41 | 42,96 |
| 1 | AB1 | 485,24 | 100,54 | 21,75 | 80,51 | 97,72 | 17,21 | 6,00 | 185,18 | 4,05 | 61,84 |
| 1 | AB1 | 252,12 | 77,66 | 16,18 | 68,68 | 91,40 | 22,72 | 5,00 | 136,18 | 4,45 | 45,99 |
| 1 | AB1 | 298,64 | 88,93 | 19,98 | 70,70 | 89,72 | 19,02 | 8,00 | 180,57 | 6,31 | 39,54 |
| 1 | AB1 | 360,32 | 89,43 | 17,53 | 74,14 | 93,76 | 19,62 | 7,00 | 190,40 | 4,98 | 47,16 |
| 1 | AB1 | 427,80 | 96,33 | 24,54 | 80,71 | 96,58 | 15,87 | 7,00 | 224,85 | 3,77 | 47,44 |
| 1 | PB1 | 395,59 | 88,78 | 22,38 | 81,11 | 99,04 | 17,93 | 7,00 | 182,06 | 4,45 | 53,98 |
| 1 | PB1 | 199,86 | 76,76 | 17,85 | 61,94 | 79,22 | 17,28 | 6,00 | 90,89 | 5,02 | 54,52 |
| 1 | PB1 | 258,33 | 86,85 | 28,94 | 70,51 | 91,04 | 20,53 | 8,00 | 121,40 | 4,68 | 53,01 |
| 1 | PB1 | 312,09 | 84,39 | 19,76 | 73,27 | 88,56 | 15,29 | 7,00 | 144,91 | 5,54 | 53,57 |
| 1 | PB1 | 372,20 | 95,08 | 20,11 | 73,24 | 92,30 | 19,06 | 7,00 | 144,59 | 3,58 | 61,15 |
| 1 | PB1 | 244,75 | 79,89 | 20,21 | 67,40 | 84,51 | 17,11 | 7,00 | 107,48 | 4,77 | 56,09 |
| 1 | PTB1 | 375,24 | 91,75 | 13,91 | 84,07 | 92,25 | 8,18 | 7,00 | 148,38 | 4,25 | 60,46 |
| 1 | PTB1 | 603,10 | 107,81 | 23,27 | 102,07 | 125,18 | 23,11 | 7,00 | 294,96 | 4,43 | 51,09 |
| 1 | PTB1 | 354,61 | 92,95 | 18,57 | 81,80 | 103,72 | 21,92 | 6,00 | 172,38 | 4,11 | 51,39 |
| 1 | PTB1 | 487,85 | 102,67 | 16,67 | 95,16 | 106,98 | 11,82 | 7,00 | 213,28 | 4,32 | 56,28 |
| 1 | PTB1 | 405,72 | 95,95 | 16,19 | 85,42 | 97,29 | 11,87 | 7,00 | 186,95 | 3,77 | 53,92 |
| 1 | PTB1 | 456,50 | 99,26 | 19,58 | 87,72 | 109,12 | 21,40 | 6,00 | 227,34 | 4,08 | 50,20 |
| 1 | PTB1 | 399,00 | 92,05 | 13,79 | 83,90 | 97,00 | 13,10 | 7,00 | 179,49 | 2,90 | 55,02 |
| 1 | PTB1 | 453,55 | 99,22 | 14,39 | 90,83 | 103,65 | 12,82 | 7,00 | 181,83 | 3,16 | 59,91 |
| 1 | PTB1 | 355,47 | 89,65 | 17,17 | 83,60 | 104,52 | 20,92 | 7,00 | 155,62 | 3,27 | 56,22 |
| 1 | PTB1 | 514,97 | 104,52 | 16,35 | 90,57 | 101,50 | 10,93 | 7,00 | 198,86 | 3,48 | 61,38 |
| 1 | SFB1 | 505,03 | 97,91 | 24,73 | 88,91 | 106,91 | 18,00 | 6,00 | 194,26 | 4,45 | 61,53 |
| 1 | SFB1 | 313,78 | 87,46 | 20,54 | 75,74 | 95,49 | 19,75 | 6,00 | 134,62 | 5,56 | 57,10 |
| 1 | SFB1 | 345,16 | 87,74 | 17,79 | 80,05 | 90,74 | 10,69 | 6,00 | 137,91 | 3,64 | 60,04 |
| 1 | SFB1 | 281,42 | 78,26 | 20,25 | 77,25 | 92,09 | 14,84 | 5,00 | 128,92 | 4,16 | 54,19 |
| 1 | SFB1 | 228,08 | 70,41 | 21,97 | 71,14 | 92,94 | 21,80 | 5,00 | 110,32 | 3,36 | 51,63 |
| 1 | SFB1 | 285,18 | 85,83 | 17,85 | 70,71 | 92,70 | 21,99 | 6,00 | 107,78 | 3,17 | 62,21 |
| 1 | SFB1 | 301,92 | 89,42 | 18,80 | 73,75 | 86,76 | 13,01 | 7,00 | 138,50 | 4,98 | 54,13 |
| 1 | SFB1 | 475,75 | 103,12 | 18,95 | 90,46 | 106,88 | 16,42 | 7,00 | 167,32 | 2,73 | 64,83 |
| 1 | SFB1 | 594,01 | 106,39 | 22,04 | 97,91 | 116,22 | 18,31 | 7,00 | 220,30 | 3,20 | 62,91 |
| 1 | SFB1 | 295,81 | 87,15 | 18,53 | 74,90 | 89,88 | 14,98 | 6,00 | 135,11 | 3,87 | 54,33 |
| 1 | PTO2 | 558,23 | 106,85 | 20,11 | 89,95 | 105,70 | 15,75 | 7,00 | 218,10 | 4,99 | 60,93 |
| 1 | PTO2 | 294,53 | 85,54 | 19,71 | 75,05 | 82,43 | 7,38 | 6,00 | 111,38 | 3,31 | 62,18 |
| 1 | PTO2 | 373,99 | 88,97 | 22,88 | 80,37 | 95,40 | 15,03 | 7,00 | 138,58 | 5,38 | 62,95 |
| 1 | PTO2 | 470,03 | 96,23 | 19,91 | 90,05 | 110,51 | 20,46 | 7,00 | 177,78 | 4,63 | 62,18 |
| 1 | PTO2 | 419,05 | 94,38 | 20,90 | 87,05 | 109,09 | 22,04 | 7,00 | 183,34 | 3,50 | 56,25 |
| 1 | PTO2 | 320,00 | 86,40 | 18,59 | 77,67 | 92,71 | 15,04 | 6,00 | 109,20 | 3,58 | 65,88 |
| 1 | PTO2 | 282,94 | 90,37 | 20,64 | 71,65 | 94,46 | 22,81 | 6,00 | 124,41 | 3,49 | 56,03 |
| 1 | PTO2 | 460,07 | 98,50 | 22,80 | 84,83 | 108,40 | 23,57 | 5,00 | 183,19 | 3,70 | 60,18 |
| 1 | PTO2 | 306,97 | 84,68 | 18,90 | 73,51 | 95,70 | 22,19 | 7,00 | 105,62 | 5,39 | 65,59 |
| 1 | PTO2 | 628,58 | 120,29 | 19,93 | 98,33 | 114,09 | 15,76 | 7,00 | 247,30 | 4,70 | 60,66 |
| 1 | PTO3 | 368,10 | 91,57 | 20,79 | 77,44 | 94,14 | 16,70 | 6,00 | 126,16 | 4,11 | 65,73 |
| 1 | PTO3 | 616,10 | 109,72 | 20,17 | 91,03 | 113,12 | 22,09 | 7,00 | 248,76 | 4,76 | 59,62 |
| 1 | PTO3 | 339,39 | 88,87 | 27,45 | 78,32 | 93,97 | 15,65 | 6,00 | 143,24 | 3,70 | 57,79 |
| 1 | PTO3 | 358,58 | 91,56 | 18,40 | 79,76 | 89,08 | 9,32 | 8,00 | 112,23 | 2,48 | 68,70 |
| 1 | PTO3 | 326,90 | 87,36 | 28,64 | 74,89 | 91,18 | 16,29 | 6,00 | 151,22 | 5,24 | 53,74 |
| 1 | PTO3 | 358,14 | 88,97 | 17,92 | 79,36 | 91,01 | 11,65 | 6,00 | 113,76 | 3,18 | 68,24 |
| 1 | PTO3 | 315,32 | 86,57 | 18,37 | 75,33 | 99,27 | 23,94 | 5,00 | 129,20 | 3,86 | 59,03 |
| 1 | PTO3 | 471,01 | 99,81 | 21,63 | 86,15 | 105,65 | 19,50 | 7,00 | 147,22 | 3,41 | 68,74 |
| 1 | PTO3 | 226,28 | 82,47 | 20,80 | 70,54 | 91,95 | 21,41 | 7,00 | 115,66 | 4,08 | 48,89 |
| 1 | PTO3 | 381,99 | 87,45 | 17,78 | 77,13 | 95,99 | 18,86 | 8,00 | 135,50 | 3,17 | 64,53 |
| 1 | PTO4 | 539,75 | 100,59 | 18,57 | 86,06 | 110,41 | 24,35 | 5,00 | 160,89 | 0,00 | 70,19 |
| 1 | PTO4 | 424,87 | 91,64 | 18,92 | 74,41 | 95,11 | 20,70 | 6,00 | 133,42 | 0,00 | 68,60 |
| 1 | PTO4 | 579,99 | 103,97 | 27,57 | 89,54 | 110,04 | 20,50 | 7,00 | 243,24 | 0,00 | 58,06 |
| 1 | PTO4 | 508,91 | 100,76 | 20,48 | 85,21 | 99,53 | 14,32 | 7,00 | 136,61 | 0,00 | 73,16 |
| 1 | PTO4 | 381,14 | 95,93 | 21,91 | 80,85 | 92,17 | 11,32 | 5,00 | 141,10 | 0,00 | 62,98 |
| 1 | PTO4 | 374,73 | 90,12 | 17,19 | 79,53 | 93,68 | 14,15 | 6,00 | 118,59 | 0,00 | 68,35 |
| 1 | PTO4 | 265,23 | 83,85 | 17,92 | 66,03 | 82,17 | 16,14 | 8,00 | 64,53 | 0,00 | 75,67 |
| 1 | PTO4 | 468,32 | 98,30 | 20,25 | 84,65 | 96,76 | 12,11 | 7,00 | 164,31 | 0,00 | 64,92 |
| 1 | PTO4 | 396,44 | 88,64 | 20,82 | 81,53 | 97,31 | 15,78 | 6,00 | 135,35 | 0,00 | 65,86 |
| 1 | PTO4 | 276,26 | 80,72 | 20,49 | 69,82 | 78,16 | 8,34 | 6,00 | 89,50 | 0,00 | 67,60 |
| 1 | PTO5 | 194,04 | 70,36 | 17,46 | 61,41 | 80,07 | 18,66 | 6,00 | 77,56 | 2,71 | 60,03 |
| 1 | PTO5 | 190,71 | 73,01 | 15,51 | 63,79 | 77,15 | 13,36 | 6,00 | 84,12 | 3,37 | 55,89 |
| 1 | PTO5 | 239,01 | 79,44 | 16,80 | 72,13 | 87,01 | 14,88 | 6,00 | 102,60 | 3,40 | 57,07 |
| 1 | PTO5 | 201,53 | 74,20 | 19,25 | 66,41 | 73,84 | 7,43 | 6,00 | 89,00 | 3,94 | 55,84 |
| 1 | PTO6 | 550,18 | 101,95 | 22,84 | 91,44 | 98,89 | 7,45 | 7,00 | 180,39 | 0,00 | 67,21 |
| 1 | PTO6 | 359,78 | 90,10 | 19,64 | 72,93 | 97,51 | 24,58 | 6,00 | 124,66 | 0,00 | 65,35 |
| 1 | PTO6 | 413,64 | 91,68 | 19,70 | 79,88 | 101,59 | 21,71 | 6,00 | 142,76 | 0,00 | 65,49 |
| 1 | PTO6 | 268,60 | 78,91 | 17,65 | 66,84 | 77,79 | 10,95 | 6,00 | 103,37 | 0,00 | 61,52 |
| 1 | PTO6 | 291,90 | 85,37 | 19,17 | 71,57 | 83,69 | 12,12 | 6,00 | 103,03 | 0,00 | 64,70 |
| 1 | PTO6 | 311,72 | 87,01 | 20,31 | 76,21 | 86,10 | 9,89 | 6,00 | 108,13 | 0,00 | 65,31 |
| 1 | PTO6 | 391,99 | 91,64 | 18,21 | 76,19 | 87,83 | 11,64 | 6,00 | 128,12 | 0,00 | 67,32 |
| 1 | PTO6 | 500,38 | 104,96 | 20,05 | 89,46 | 93,76 | 4,30 | 7,00 | 164,02 | 0,00 | 67,22 |
| 1 | PTO6 | 604,20 | 105,55 | 22,36 | 91,27 | 104,01 | 12,74 | 7,00 | 192,16 | 0,00 | 68,20 |
| 1 | PTO6 | 443,29 | 94,60 | 18,82 | 82,87 | 98,93 | 16,06 | 6,00 | 144,18 | 0,00 | 67,48 |
| 1 | PTO7 | 291,17 | 82,24 | 19,07 | 72,64 | 91,30 | 18,66 | 6,00 | 118,22 | 3,56 | 59,40 |
| 1 | PTO7 | 435,74 | 91,30 | 19,35 | 82,72 | 103,72 | 21,00 | 6,00 | 138,44 | 4,83 | 68,23 |
| 1 | PTO7 | 306,63 | 84,60 | 20,37 | 76,71 | 90,53 | 13,82 | 6,00 | 112,96 | 4,04 | 63,16 |
| 1 | PTO7 | 513,88 | 103,66 | 22,99 | 94,87 | 113,39 | 18,52 | 6,00 | 172,50 | 2,60 | 66,43 |
| 1 | PTO7 | 405,10 | 90,64 | 20,00 | 83,15 | 103,50 | 20,35 | 6,00 | 145,22 | 3,38 | 64,15 |
| 1 | PTO7 | 328,40 | 87,25 | 20,25 | 77,48 | 98,00 | 20,52 | 5,00 | 129,97 | 5,16 | 60,42 |
| 1 | PTO7 | 461,34 | 96,23 | 24,31 | 83,64 | 93,64 | 10,00 | 7,00 | 167,11 | 3,94 | 63,78 |
| 1 | PTO7 | 503,86 | 98,68 | 17,03 | 85,98 | 98,09 | 12,11 | 8,00 | 161,58 | 4,37 | 67,93 |
| 1 | PTO7 | 361,07 | 87,63 | 17,02 | 77,02 | 91,39 | 14,37 | 6,00 | 106,16 | 4,94 | 70,60 |
| 1 | PTO7 | 219,09 | 73,03 | 23,83 | 63,88 | 85,50 | 21,62 | 6,00 | 102,97 | 4,15 | 53,00 |
| 1 | PTO8 | 399,64 | 90,72 | 18,29 | 79,34 | 95,72 | 16,38 | 6,00 | 116,38 | 2,73 | 70,88 |
| 1 | PTO8 | 534,93 | 98,11 | 21,05 | 83,80 | 105,77 | 21,97 | 8,00 | 212,61 | 3,31 | 60,25 |
| 1 | PTO8 | 322,91 | 84,24 | 15,76 | 68,77 | 88,47 | 19,70 | 6,00 | 122,44 | 2,84 | 62,08 |
| 1 | PTO8 | 309,52 | 81,99 | 15,02 | 69,72 | 89,42 | 19,70 | 6,00 | 98,27 | 2,74 | 68,25 |
| 1 | PTO8 | 333,94 | 83,97 | 16,51 | 73,93 | 89,82 | 15,89 | 6,00 | 119,96 | 2,82 | 64,08 |
| 1 | PTO8 | 377,66 | 88,51 | 16,90 | 75,40 | 94,11 | 18,71 | 5,00 | 151,82 | 2,63 | 59,80 |
| 1 | PTO8 | 574,44 | 101,56 | 17,47 | 84,93 | 104,53 | 19,60 | 6,00 | 188,99 | 3,62 | 67,10 |
| 1 | PTO8 | 240,23 | 72,35 | 11,38 | 61,51 | 81,51 | 20,00 | 6,00 | 87,06 | 2,30 | 63,76 |
| 1 | PTO8 | 386,37 | 72,21 | 18,91 | 82,05 | 102,82 | 20,77 | 6,00 | 102,62 | 3,36 | 73,44 |
| 1 | PTO8 | 235,21 | 77,97 | 18,15 | 67,60 | 85,10 | 17,50 | 6,00 | 105,51 | 2,56 | 55,14 |
| 1 | PTO9 | 332,59 | 86,82 | 24,46 | 72,62 | 97,30 | 24,68 | 6,00 | 162,24 | 5,15 | 51,22 |
| 1 | PTO9 | 277,61 | 85,07 | 17,59 | 70,10 | 84,34 | 14,24 | 6,00 | 110,98 | 4,93 | 60,02 |
| 1 | PTO9 | 219,35 | 77,07 | 12,99 | 65,46 | 74,10 | 8,64 | 6,00 | 119,38 | 5,23 | 45,58 |
| 1 | PTO9 | 371,59 | 91,04 | 26,46 | 77,82 | 98,29 | 20,47 | 6,00 | 190,10 | 4,86 | 48,84 |
| 1 | PTO9 | 315,05 | 83,66 | 21,68 | 72,21 | 89,27 | 17,06 | 6,00 | 147,49 | 5,47 | 53,19 |
| 1 | PTO9 | 207,01 | 76,92 | 16,71 | 65,04 | 76,33 | 11,29 | 6,00 | 72,93 | 2,67 | 64,77 |
| 1 | PTO9 | 198,56 | 70,81 | 17,02 | 64,87 | 85,86 | 20,99 | 6,00 | 101,01 | 3,57 | 49,13 |
| 1 | PTO9 | 281,04 | 81,23 | 18,31 | 76,88 | 94,41 | 17,53 | 5,00 | 136,73 | 5,46 | 51,35 |
| 1 | PTO9 | 257,47 | 81,56 | 27,37 | 71,48 | 90,01 | 18,53 | 6,00 | 147,73 | 5,48 | 42,62 |
| 1 | PTO9 | 346,54 | 86,55 | 17,96 | 80,55 | 99,43 | 18,88 | 6,00 | 136,29 | 3,31 | 60,67 |
| 1 | PDO2 | 516,68 | 104,10 | 20,72 | 86,35 | 102,74 | 16,39 | 7,00 | 189,39 | 3,78 | 63,34 |
| 1 | PDO2 | 521,13 | 103,72 | 19,26 | 87,72 | 105,23 | 17,51 | 7,00 | 218,05 | 3,93 | 58,16 |
| 1 | PDO2 | 449,91 | 102,49 | 19,05 | 80,84 | 89,81 | 8,97 | 7,00 | 157,35 | 4,13 | 65,03 |
| 1 | PDO2 | 441,86 | 91,02 | 20,41 | 89,15 | 99,11 | 9,96 | 6,00 | 181,32 | 4,54 | 58,96 |
| 1 | PDO2 | 435,09 | 98,26 | 18,94 | 79,23 | 98,16 | 18,93 | 7,00 | 192,65 | 4,57 | 55,72 |
| 1 | PDO2 | 437,98 | 99,94 | 20,53 | 78,09 | 94,70 | 16,61 | 7,00 | 231,84 | 4,02 | 47,07 |
| 1 | PDO2 | 258,69 | 81,19 | 14,65 | 69,59 | 77,94 | 8,35 | 7,00 | 103,49 | 2,48 | 59,99 |
| 1 | PDO2 | 325,60 | 92,44 | 20,94 | 78,63 | 99,99 | 21,36 | 7,00 | 124,56 | 3,67 | 61,74 |
| 1 | PDO2 | 324,58 | 87,53 | 17,98 | 73,62 | 89,83 | 16,21 | 7,00 | 122,48 | 3,92 | 62,27 |
| 1 | PDO2 | 458,12 | 98,73 | 26,42 | 80,15 | 100,58 | 20,43 | 6,00 | 195,08 | 4,73 | 57,42 |
| 1 | CRO1 | 514,83 | 105,84 | 19,04 | 85,34 | 105,35 | 20,01 | 6,00 | 162,67 | 3,60 | 68,40 |
| 1 | CRO1 | 391,22 | 93,82 | 23,26 | 87,45 | 107,68 | 20,23 | 6,00 | 145,10 | 3,29 | 62,91 |
| 1 | CRO1 | 398,38 | 95,39 | 20,47 | 90,33 | 109,13 | 18,80 | 6,00 | 159,69 | 2,27 | 59,92 |
| 1 | CRO1 | 644,20 | 110,63 | 19,05 | 99,45 | 113,34 | 13,89 | 8,00 | 271,69 | 5,05 | 57,83 |
| 1 | CRO1 | 219,39 | 74,47 | 18,98 | 68,53 | 86,15 | 17,62 | 6,00 | 115,00 | 3,24 | 47,58 |
| 1 | CRO1 | 324,90 | 87,87 | 20,32 | 75,19 | 96,18 | 20,99 | 7,00 | 124,87 | 3,23 | 61,57 |
| 1 | CRO1 | 460,30 | 104,03 | 18,42 | 88,74 | 104,40 | 15,66 | 6,00 | 155,24 | 3,62 | 66,27 |
| 1 | CRO1 | 594,67 | 112,33 | 22,40 | 53,82 | 74,93 | 21,11 | 7,00 | 202,96 | 3,31 | 65,87 |
| 1 | CRO1 | 288,89 | 84,03 | 17,62 | 80,56 | 97,73 | 17,17 | 6,00 | 123,18 | 3,39 | 57,36 |
| 1 | CRO1 | 376,81 | 94,94 | 20,66 | 82,88 | 111,64 | 28,76 | 6,00 | 151,48 | 2,71 | 59,80 |
| 1 | CRO2 | 416,14 | 98,60 | 20,50 | 87,67 | 105,00 | 17,33 | 6,00 | 169,68 | 3,58 | 59,23 |
| 1 | CRO2 | 448,03 | 99,85 | 22,08 | 90,33 | 108,58 | 18,25 | 6,00 | 149,22 | 3,69 | 66,69 |
| 1 | CRO2 | 587,80 | 110,85 | 20,87 | 90,98 | 113,14 | 22,16 | 6,00 | 207,24 | 3,40 | 64,74 |
| 1 | CRO2 | 259,68 | 80,53 | 21,00 | 69,99 | 91,47 | 21,48 | 6,00 | 82,90 | 3,26 | 68,08 |
| 1 | CRO2 | 333,39 | 89,55 | 17,01 | 78,90 | 96,73 | 17,83 | 7,00 | 136,17 | 3,91 | 59,16 |
| 1 | CRO2 | 264,04 | 86,23 | 20,68 | 72,39 | 87,21 | 14,82 | 6,00 | 122,63 | 3,86 | 53,56 |
| 1 | CRO2 | 305,74 | 88,38 | 20,02 | 70,94 | 83,45 | 12,51 | 7,00 | 109,47 | 3,85 | 64,20 |
| 1 | CRO2 | 347,79 | 94,37 | 19,15 | 80,61 | 101,27 | 20,66 | 7,00 | 163,87 | 4,79 | 52,88 |
| 1 | CRO2 | 452,12 | 104,62 | 24,07 | 82,99 | 106,69 | 23,70 | 7,00 | 178,57 | 3,45 | 60,50 |
| 1 | CRO2 | 232,43 | 81,45 | 18,94 | 72,55 | 98,17 | 25,62 | 5,00 | 113,48 | 3,71 | 51,18 |
| 1 | ADO3 | 268,94 | 82,52 | 25,27 | 68,21 | 88,04 | 19,83 | 6,00 | 110,58 | 4,37 | 58,88 |
| 1 | ADO3 | 256,58 | 78,04 | 20,79 | 69,12 | 86,51 | 17,39 | 6,00 | 104,69 | 3,51 | 59,20 |
| 1 | ADO3 | 304,95 | 85,18 | 17,18 | 72,73 | 96,39 | 23,66 | 6,00 | 130,25 | 3,81 | 57,29 |
| 1 | ADO3 | 446,54 | 93,94 | 23,04 | 85,77 | 103,28 | 17,51 | 6,00 | 150,43 | 5,02 | 66,31 |
| 1 | ADO3 | 275,67 | 79,04 | 20,72 | 67,15 | 89,68 | 22,53 | 5,00 | 115,24 | 4,85 | 58,20 |
| 1 | ADO3 | 377,65 | 93,38 | 20,81 | 79,72 | 102,63 | 22,91 | 6,00 | 158,09 | 5,14 | 58,14 |
| 1 | ADO3 | 332,70 | 85,44 | 20,03 | 77,43 | 94,10 | 16,67 | 6,00 | 126,40 | 3,21 | 62,01 |
| 1 | ADO3 | 479,40 | 98,07 | 15,32 | 87,35 | 108,48 | 21,13 | 7,00 | 163,66 | 4,02 | 65,86 |
| 1 | ADO3 | 478,59 | 98,17 | 21,55 | 85,76 | 96,06 | 10,30 | 6,00 | 158,22 | 2,19 | 66,94 |
| 1 | ADO3 | 551,32 | 101,44 | 24,56 | 91,17 | 109,11 | 17,94 | 8,00 | 202,97 | 3,15 | 63,18 |
| 1 | BO1 | 291,34 | 83,37 | 21,45 | 77,27 | 93,92 | 16,65 | 5,00 | 127,12 | 3,57 | 56,37 |
| 1 | BO1 | 285,76 | 84,59 | 20,16 | 73,01 | 92,49 | 19,48 | 5,00 | 151,69 | 4,85 | 46,92 |
| 1 | BO1 | 406,03 | 89,45 | 20,98 | 81,12 | 102,77 | 21,65 | 6,00 | 143,22 | 2,93 | 64,73 |
| 1 | BO1 | 476,28 | 100,76 | 27,67 | 85,74 | 104,63 | 18,89 | 6,00 | 198,24 | 3,49 | 58,38 |
| 1 | BO1 | 384,80 | 91,95 | 20,76 | 80,22 | 95,92 | 15,70 | 6,00 | 163,33 | 5,48 | 57,55 |
| 1 | BO1 | 501,62 | 102,02 | 30,69 | 90,78 | 107,44 | 16,66 | 6,00 | 236,89 | 5,10 | 52,78 |
| 1 | BO1 | 434,89 | 96,90 | 26,33 | 87,02 | 106,68 | 19,66 | 7,00 | 217,53 | 5,09 | 49,98 |
| 1 | BO1 | 390,84 | 93,63 | 20,41 | 81,00 | 98,19 | 17,19 | 6,00 | 177,77 | 4,65 | 54,52 |
| 1 | BO1 | 309,67 | 82,20 | 29,75 | 75,92 | 93,17 | 17,25 | 5,00 | 148,58 | 4,62 | 52,02 |
| 1 | BO1 | 312,74 | 87,30 | 20,98 | 78,63 | 99,15 | 20,52 | 6,00 | 127,67 | 2,88 | 59,18 |
| 1 | ADO2 | 450,70 | 98,62 | 18,52 | 82,67 | 100,85 | 18,18 | 8,00 | 186,21 | 0,00 | 58,68 |
| 1 | ADO2 | 310,00 | 86,20 | 18,61 | 71,87 | 92,15 | 20,28 | 6,00 | 113,21 | 0,00 | 63,48 |
| 1 | ADO2 | 418,58 | 92,96 | 24,85 | 84,37 | 105,26 | 20,89 | 5,00 | 189,18 | 0,00 | 54,80 |
| 1 | ADO2 | 206,66 | 74,58 | 18,26 | 66,79 | 84,99 | 18,20 | 6,00 | 74,64 | 0,00 | 63,88 |
| 1 | ADO2 | 263,56 | 85,45 | 19,65 | 68,94 | 88,34 | 19,40 | 5,00 | 161,58 | 0,00 | 38,69 |
| 1 | ADO2 | 343,62 | 84,92 | 24,31 | 78,53 | 98,60 | 20,07 | 5,00 | 161,58 | 0,00 | 52,98 |
| 1 | ADO2 | 374,14 | 84,01 | 18,67 | 81,62 | 92,17 | 10,55 | 6,00 | 106,15 | 0,00 | 71,63 |
| 1 | ADO2 | 307,52 | 85,59 | 21,17 | 75,24 | 96,03 | 20,79 | 6,00 | 114,51 | 0,00 | 62,76 |
| 1 | ADO2 | 310,77 | 84,52 | 23,65 | 74,50 | 93,00 | 18,50 | 6,00 | 130,51 | 0,00 | 58,00 |
| 1 | ADO2 | 305,43 | 85,66 | 19,08 | 76,12 | 97,06 | 20,94 | 6,00 | 101,08 | 0,00 | 66,91 |
| 1 | PG | 376,27 | 92,80 | 18,01 | 78,06 | 88,40 | 10,34 | 6,00 | 156,91 | 3,90 | 58,30 |
| 1 | PG | 404,63 | 95,92 | 18,77 | 76,12 | 99,37 | 23,25 | 6,00 | 171,80 | 4,31 | 57,54 |
| 1 | PG | 286,26 | 84,08 | 18,46 | 66,90 | 85,09 | 18,19 | 6,00 | 120,97 | 4,43 | 57,74 |
| 1 | PG | 306,90 | 86,07 | 18,10 | 70,68 | 89,20 | 18,52 | 6,00 | 139,82 | 4,29 | 54,44 |
| 1 | PG | 262,02 | 78,72 | 17,95 | 66,83 | 85,20 | 18,37 | 6,00 | 99,25 | 3,30 | 62,12 |
| 1 | PG | 296,81 | 87,27 | 17,56 | 69,90 | 90,32 | 20,42 | 7,00 | 142,15 | 3,63 | 52,11 |
| 1 | PG | 395,01 | 92,02 | 18,47 | 80,56 | 99,12 | 18,56 | 7,00 | 132,77 | 3,68 | 66,39 |
| 1 | PG | 380,40 | 94,40 | 15,49 | 72,62 | 84,54 | 11,92 | 6,00 | 129,78 | 5,69 | 65,88 |
| 1 | PG | 453,55 | 95,30 | 22,32 | 85,78 | 101,37 | 15,59 | 7,00 | 201,83 | 3,53 | 55,50 |
| 1 | PG | 321,33 | 86,76 | 17,44 | 71,91 | 89,71 | 17,80 | 6,00 | 137,61 | 3,68 | 57,17 |
| 2 | ME1 | 436,20 | 96,19 | 24,17 | 81,01 | 99,14 | 18,13 | 6,00 | 172,71 | 3,58 | 60,41 |
| 2 | ME1 | 311,80 | 84,30 | 18,90 | 76,36 | 89,63 | 13,27 | 6,00 | 108,58 | 3,50 | 65,18 |
| 2 | ME1 | 407,20 | 93,60 | 20,79 | 83,50 | 103,12 | 19,62 | 6,00 | 156,98 | 3,80 | 61,45 |
| 2 | ME1 | 153,20 | 66,56 | 20,83 | 57,62 | 71,09 | 13,47 | 6,00 | 62,20 | 4,81 | 59,40 |
| 2 | ME1 | 295,00 | 81,44 | 19,76 | 67,55 | 89,80 | 22,25 | 6,00 | 128,54 | 3,40 | 56,43 |
| 2 | ME1 | 452,20 | 98,33 | 16,98 | 88,75 | 105,58 | 16,83 | 7,00 | 152,60 | 3,91 | 66,25 |
| 2 | ME1 | 230,80 | 81,05 | 17,40 | 68,47 | 86,78 | 18,31 | 6,00 | 87,20 | 3,52 | 62,22 |
| 2 | ME1 | 243,80 | 82,26 | 23,03 | 73,26 | 84,03 | 10,77 | 6,00 | 97,71 | 3,40 | 59,92 |
| 2 | ME1 | 183,80 | 69,05 | 19,05 | 64,34 | 83,15 | 18,81 | 6,00 | 64,40 | 2,05 | 64,96 |
| 2 | ME1 | 303,50 | 86,44 | 19,91 | 62,65 | 90,89 | 28,24 | 7,00 | 112,49 | 3,34 | 62,94 |
| 2 | ME2 | 283,89 | 84,65 | 19,19 | 73,70 | 92,24 | 18,54 | 6,00 | 158,57 | 3,91 | 44,14 |
| 2 | ME2 | 342,93 | 90,20 | 30,10 | 73,76 | 93,92 | 20,16 | 6,00 | 158,30 | 4,52 | 53,84 |
| 2 | ME2 | 391,72 | 94,89 | 17,56 | 77,08 | 88,90 | 11,82 | 7,00 | 136,23 | 3,19 | 65,22 |
| 2 | ME2 | 209,15 | 74,21 | 18,19 | 62,72 | 74,58 | 11,86 | 7,00 | 81,09 | 3,16 | 61,23 |
| 2 | ME2 | 291,08 | 84,50 | 24,28 | 71,44 | 91,07 | 19,63 | 8,00 | 118,49 | 4,38 | 59,29 |
| 2 | ME2 | 220,75 | 79,80 | 17,15 | 67,66 | 87,66 | 20,00 | 7,00 | 77,01 | 2,43 | 65,11 |
| 2 | ME2 | 187,15 | 72,64 | 17,67 | 62,59 | 79,07 | 16,48 | 7,00 | 60,23 | 1,58 | 67,82 |
| 2 | ME2 | 367,51 | 89,88 | 18,82 | 76,38 | 91,68 | 15,30 | 7,00 | 132,97 | 2,96 | 63,82 |
| 2 | ME2 | 249,50 | 76,07 | 18,61 | 72,24 | 89,53 | 17,29 | 7,00 | 79,46 | 2,31 | 68,15 |
| 2 | ME2 | 290,88 | 85,54 | 19,16 | 70,23 | 89,83 | 19,60 | 7,00 | 108,81 | 2,74 | 62,59 |
| 2 | ME3 | 415,71 | 94,29 | 20,02 | 79,09 | 98,36 | 19,27 | 6,00 | 131,36 | 3,48 | 68,40 |
| 2 | ME3 | 356,71 | 90,51 | 18,20 | 77,27 | 97,47 | 20,20 | 5,00 | 116,68 | 3,46 | 67,29 |
| 2 | ME3 | 499,77 | 102,98 | 19,06 | 82,44 | 99,44 | 17,00 | 7,00 | 139,54 | 2,54 | 72,08 |
| 2 | ME3 | 337,74 | 86,83 | 16,46 | 78,74 | 87,78 | 9,04 | 6,00 | 99,20 | 2,29 | 70,63 |
| 2 | ME3 | 381,82 | 92,95 | 22,02 | 78,94 | 101,17 | 22,23 | 6,00 | 112,75 | 2,48 | 70,47 |
| 2 | ME3 | 282,89 | 79,91 | 18,97 | 78,83 | 92,24 | 13,41 | 6,00 | 82,76 | 2,49 | 70,74 |
| 2 | ME3 | 302,97 | 85,27 | 18,52 | 73,53 | 85,52 | 11,99 | 7,00 | 114,69 | 3,27 | 62,14 |
| 2 | ME3 | 403,48 | 94,90 | 21,12 | 77,72 | 95,76 | 18,04 | 5,00 | 118,25 | 2,15 | 70,69 |
| 2 | ME3 | 305,11 | 88,16 | 19,92 | 76,73 | 98,06 | 21,33 | 5,00 | 116,85 | 2,60 | 61,70 |
| 2 | ME3 | 415,55 | 91,98 | 18,81 | 80,50 | 98,72 | 18,22 | 7,00 | 116,32 | 1,53 | 72,01 |
| 2 | ME31 | 361,61 | 90,24 | 20,06 | 75,80 | 93,45 | 17,65 | 6,00 | 118,85 | 3,67 | 67,13 |
| 2 | ME31 | 282,63 | 83,63 | 18,20 | 70,85 | 89,60 | 18,75 | 6,00 | 100,04 | 4,31 | 64,60 |
| 2 | ME31 | 373,27 | 92,51 | 18,03 | 83,56 | 101,63 | 18,07 | 7,00 | 122,30 | 3,23 | 67,24 |
| 2 | ME31 | 358,61 | 93,62 | 18,70 | 75,48 | 92,80 | 17,32 | 7,00 | 122,89 | 4,06 | 65,73 |
| 2 | ME31 | 417,33 | 92,80 | 20,78 | 79,37 | 100,11 | 20,74 | 7,00 | 146,13 | 2,76 | 64,98 |
| 2 | ME31 | 376,96 | 94,36 | 21,93 | 73,61 | 93,89 | 20,28 | 5,00 | 159,03 | 3,03 | 57,81 |
| 2 | ME31 | 357,19 | 91,66 | 23,03 | 74,15 | 97,02 | 22,87 | 8,00 | 134,31 | 4,16 | 62,40 |
| 2 | ME31 | 347,92 | 86,61 | 19,58 | 77,45 | 97,92 | 20,47 | 7,00 | 111,96 | 3,14 | 67,82 |
| 2 | ME31 | 293,33 | 83,28 | 17,34 | 73,47 | 92,57 | 19,10 | 7,00 | 106,60 | 3,00 | 63,66 |
| 2 | ME31 | 208,61 | 77,56 | 18,89 | 64,92 | 78,65 | 13,73 | 7,00 | 66,82 | 3,15 | 67,97 |
| 2 | ME4 | 292,04 | 85,27 | 17,81 | 75,83 | 94,21 | 18,38 | 8,00 | 101,74 | 3,73 | 65,16 |
| 2 | ME4 | 315,19 | 87,72 | 17,51 | 76,33 | 92,15 | 15,82 | 6,00 | 116,83 | 3,31 | 62,93 |
| 2 | ME4 | 285,73 | 82,06 | 23,95 | 76,06 | 93,62 | 17,56 | 6,00 | 98,89 | 4,85 | 65,39 |
| 2 | ME4 | 312,34 | 85,76 | 18,54 | 76,40 | 95,63 | 19,23 | 5,00 | 90,83 | 4,32 | 70,92 |
| 2 | ME4 | 380,92 | 93,72 | 19,23 | 80,60 | 97,86 | 17,26 | 6,00 | 144,05 | 4,59 | 62,18 |
| 2 | ME4 | 326,46 | 91,55 | 19,10 | 71,29 | 82,15 | 10,86 | 7,00 | 106,70 | 3,37 | 67,32 |
| 2 | ME4 | 251,78 | 79,99 | 19,55 | 66,60 | 85,45 | 18,85 | 6,00 | 86,12 | 3,69 | 65,80 |
| 2 | ME4 | 283,37 | 84,16 | 19,63 | 75,25 | 93,80 | 18,55 | 8,00 | 95,64 | 3,01 | 66,25 |
| 2 | ME4 | 256,89 | 80,73 | 17,62 | 69,32 | 86,25 | 16,93 | 6,00 | 77,30 | 2,68 | 69,91 |
| 2 | ME4 | 187,11 | 74,93 | 16,62 | 64,80 | 78,20 | 13,40 | 6,00 | 76,31 | 3,91 | 59,22 |
| 2 | ME5 | 574,16 | 106,39 | 26,30 | 85,31 | 107,95 | 22,64 | 8,00 | 222,65 | 4,81 | 61,22 |
| 2 | ME5 | 470,38 | 97,03 | 16,72 | 85,39 | 98,72 | 13,33 | 7,00 | 153,94 | 4,44 | 67,27 |
| 2 | ME5 | 325,00 | 87,24 | 21,59 | 76,09 | 94,56 | 18,47 | 7,00 | 176,52 | 4,07 | 45,69 |
| 2 | ME5 | 284,31 | 83,37 | 21,85 | 72,85 | 92,31 | 19,46 | 6,00 | 133,08 | 5,17 | 53,19 |
| 2 | ME5 | 346,39 | 92,28 | 19,15 | 79,47 | 99,51 | 20,04 | 6,00 | 150,47 | 5,30 | 56,56 |
| 2 | ME5 | 183,57 | 74,30 | 21,83 | 64,05 | 81,17 | 17,12 | 6,00 | 94,46 | 5,23 | 48,54 |
| 2 | ME5 | 220,31 | 77,18 | 17,30 | 66,03 | 74,30 | 8,27 | 6,00 | 80,25 | 4,76 | 63,57 |
| 2 | ME5 | 349,14 | 87,87 | 22,77 | 79,47 | 99,29 | 19,82 | 7,00 | 135,47 | 3,03 | 61,20 |
| 2 | ME5 | 319,33 | 89,97 | 20,72 | 72,36 | 91,79 | 19,43 | 7,00 | 111,58 | 3,46 | 65,06 |
| 2 | ME5 | 342,52 | 88,20 | 17,13 | 75,80 | 85,23 | 9,43 | 7,00 | 114,93 | 3,07 | 66,45 |
| 2 | ME6 | 363,65 | 89,56 | 21,66 | 72,36 | 93,03 | 20,67 | 7,00 | 141,84 | 2,80 | 61,00 |
| 2 | ME6 | 444,04 | 60,42 | 21,90 | 81,61 | 99,35 | 17,74 | 7,00 | 151,60 | 4,08 | 65,86 |
| 2 | ME6 | 296,24 | 86,21 | 24,53 | 73,82 | 98,35 | 24,53 | 6,00 | 135,00 | 6,17 | 54,43 |
| 2 | ME6 | 302,86 | 81,82 | 22,88 | 75,22 | 93,77 | 18,55 | 6,00 | 116,89 | 4,53 | 61,40 |
| 2 | ME6 | 356,90 | 85,89 | 22,51 | 80,76 | 93,62 | 12,86 | 6,00 | 151,20 | 5,45 | 57,64 |
| 2 | ME6 | 377,23 | 88,65 | 20,78 | 84,41 | 92,77 | 8,36 | 7,00 | 154,30 | 3,42 | 59,10 |
| 2 | ME6 | 171,23 | 71,61 | 18,50 | 62,65 | 81,71 | 19,06 | 7,00 | 75,27 | 5,23 | 56,04 |
| 2 | ME6 | 288,72 | 82,09 | 21,77 | 73,05 | 84,04 | 10,99 | 7,00 | 111,70 | 4,05 | 61,31 |
| 2 | ME6 | 386,80 | 92,22 | 27,06 | 83,65 | 97,92 | 14,27 | 6,00 | 186,40 | 4,80 | 51,81 |
| 2 | ME6 | 355,56 | 91,81 | 24,18 | 78,72 | 94,78 | 16,06 | 7,00 | 154,25 | 5,54 | 56,62 |
| 2 | ME7 | 317,80 | 83,91 | 19,40 | 73,80 | 87,43 | 13,63 | 6,00 | 137,20 | 4,28 | 56,83 |
| 2 | ME7 | 186,20 | 70,50 | 21,40 | 64,80 | 82,14 | 17,34 | 5,00 | 75,67 | 4,11 | 59,36 |
| 2 | ME7 | 481,60 | 95,14 | 25,82 | 81,24 | 99,73 | 18,49 | 7,00 | 145,06 | 5,14 | 69,88 |
| 2 | ME7 | 433,88 | 96,05 | 28,50 | 81,55 | 101,70 | 20,15 | 7,00 | 150,50 | 6,13 | 65,31 |
| 2 | ME7 | 269,02 | 81,33 | 19,88 | 75,42 | 88,89 | 13,47 | 6,00 | 138,25 | 3,28 | 48,61 |
| 2 | ME7 | 283,25 | 80,19 | 25,20 | 70,15 | 87,06 | 16,91 | 7,00 | 117,08 | 6,21 | 58,67 |
| 2 | ME7 | 349,70 | 86,85 | 20,40 | 79,02 | 93,59 | 14,57 | 7,00 | 135,30 | 3,33 | 61,31 |
| 2 | ME7 | 273,96 | 80,30 | 19,40 | 78,40 | 84,09 | 5,69 | 7,00 | 154,22 | 4,77 | 43,71 |
| 2 | ME7 | 297,05 | 83,41 | 21,59 | 75,80 | 93,89 | 18,09 | 6,00 | 95,26 | 6,06 | 67,93 |
| 2 | ME7 | 300,76 | 84,86 | 25,13 | 75,80 | 90,89 | 15,09 | 6,00 | 127,10 | 4,15 | 57,74 |
| 2 | ME8 | 376,08 | 88,27 | 24,07 | 78,38 | 90,93 | 12,55 | 6,00 | 165,34 | 4,98 | 56,04 |
| 2 | ME8 | 310,57 | 84,91 | 21,83 | 71,47 | 88,20 | 16,73 | 6,00 | 144,90 | 3,95 | 53,34 |
| 2 | ME8 | 289,88 | 81,58 | 20,86 | 73,22 | 82,90 | 9,68 | 7,00 | 131,60 | 3,62 | 54,60 |
| 2 | ME8 | 389,45 | 91,74 | 24,74 | 81,40 | 94,11 | 12,71 | 7,00 | 184,50 | 4,46 | 52,63 |
| 2 | ME8 | 223,37 | 70,26 | 19,27 | 76,81 | 80,46 | 3,65 | 6,00 | 105,80 | 4,15 | 52,63 |
| 2 | ME8 | 330,27 | 83,15 | 19,38 | 75,11 | 96,19 | 21,08 | 7,00 | 165,25 | 4,56 | 49,97 |
| 2 | ME8 | 189,14 | 72,73 | 20,09 | 59,04 | 77,35 | 18,31 | 6,00 | 94,68 | 3,60 | 49,94 |
| 2 | ME8 | 337,21 | 83,92 | 19,22 | 75,92 | 85,15 | 9,23 | 7,00 | 126,60 | 3,09 | 62,46 |
| 2 | ME8 | 294,70 | 83,36 | 22,17 | 71,60 | 81,65 | 10,05 | 7,00 | 128,28 | 4,58 | 56,47 |
| 2 | ME8 | 354,71 | 84,07 | 20,33 | 74,70 | 91,17 | 16,47 | 7,00 | 130,63 | 4,50 | 63,17 |
| 2 | ME9 | 373,93 | 89,91 | 20,24 | 76,42 | 96,13 | 19,71 | 7,00 | 136,27 | 2,59 | 63,56 |
| 2 | ME9 | 302,14 | 85,28 | 26,50 | 72,70 | 85,90 | 13,20 | 7,00 | 136,80 | 4,40 | 54,72 |
| 2 | ME9 | 277,76 | 82,35 | 20,15 | 74,32 | 92,37 | 18,05 | 6,00 | 131,70 | 5,00 | 52,58 |
| 2 | ME9 | 300,50 | 84,83 | 19,60 | 76,20 | 97,13 | 20,93 | 6,00 | 152,20 | 4,71 | 49,35 |
| 2 | ME9 | 219,01 | 74,26 | 25,55 | 65,83 | 85,15 | 19,32 | 6,00 | 125,20 | 5,69 | 42,83 |
| 2 | ME9 | 385,90 | 88,14 | 23,30 | 79,60 | 96,65 | 17,05 | 7,00 | 192,20 | 7,42 | 50,19 |
| 2 | ME9 | 410,03 | 94,50 | 25,40 | 81,00 | 103,40 | 22,40 | 7,00 | 171,60 | 5,17 | 58,15 |
| 2 | ME9 | 418,00 | 93,62 | 22,80 | 83,60 | 100,44 | 16,84 | 7,00 | 178,70 | 4,48 | 57,25 |
| 2 | ME9 | 207,15 | 74,95 | 20,30 | 77,50 | 92,87 | 15,37 | 6,00 | 100,00 | 3,97 | 51,73 |
| 2 | ME9 | 240,35 | 77,95 | 22,82 | 66,50 | 87,18 | 20,68 | 7,00 | 106,06 | 4,60 | 55,87 |
| 2 | ME10 | 298,80 | 83,80 | 24,40 | 66,09 | 81,00 | 14,91 | 6,00 | 135,43 | 5,80 | 54,68 |
| 2 | ME10 | 200,90 | 75,21 | 24,34 | 64,40 | 79,40 | 15,00 | 7,00 | 97,80 | 3,26 | 51,32 |
| 2 | ME10 | 297,40 | 80,89 | 19,28 | 76,11 | 92,09 | 15,98 | 6,00 | 131,60 | 2,34 | 55,75 |
| 2 | ME10 | 187,68 | 73,01 | 22,70 | 59,65 | 79,30 | 19,65 | 6,00 | 93,20 | 3,18 | 50,34 |
| 2 | ME10 | 383,30 | 88,78 | 23,52 | 76,25 | 95,60 | 19,35 | 6,00 | 122,40 | 1,77 | 68,07 |
| 2 | ME10 | 276,99 | 77,38 | 22,30 | 72,30 | 88,03 | 15,73 | 7,00 | 100,20 | 2,33 | 63,83 |
| 2 | ME10 | 297,17 | 80,80 | 25,50 | 75,89 | 92,04 | 16,15 | 7,00 | 162,10 | 4,90 | 45,45 |
| 2 | ME10 | 421,90 | 95,47 | 17,30 | 81,70 | 94,70 | 13,00 | 6,00 | 148,50 | 3,57 | 64,80 |
| 2 | ME10 | 369,02 | 90,20 | 21,68 | 83,00 | 96,30 | 13,30 | 7,00 | 134,50 | 5,24 | 63,55 |
| 2 | ME10 | 353,52 | 90,17 | 20,30 | 77,28 | 93,77 | 16,49 | 7,00 | 148,80 | 4,50 | 57,91 |
| 2 | ME11 | 325,58 | 82,79 | 20,30 | 73,12 | 87,10 | 13,98 | 7,00 | 141,63 | 4,92 | 56,50 |
| 2 | ME11 | 364,45 | 85,38 | 23,93 | 75,70 | 91,50 | 15,80 | 6,00 | 115,80 | 3,68 | 68,23 |
| 2 | ME11 | 292,73 | 81,83 | 23,26 | 72,56 | 92,56 | 20,00 | 6,00 | 116,05 | 3,28 | 60,36 |
| 2 | ME11 | 223,95 | 77,40 | 19,54 | 64,90 | 79,45 | 14,55 | 7,00 | 102,31 | 4,51 | 54,32 |
| 2 | ME11 | 251,20 | 81,30 | 24,50 | 67,70 | 86,06 | 18,36 | 7,00 | 110,47 | 4,67 | 56,02 |
| 2 | ME11 | 255,33 | 80,05 | 22,06 | 71,50 | 84,37 | 12,87 | 7,00 | 126,78 | 1,53 | 50,35 |
| 2 | ME11 | 359,60 | 86,65 | 26,02 | 78,25 | 95,96 | 17,71 | 6,00 | 144,35 | 2,86 | 59,86 |
| 2 | ME11 | 283,23 | 82,02 | 24,12 | 72,80 | 90,40 | 17,60 | 7,00 | 153,60 | 2,75 | 45,77 |
| 2 | ME11 | 270,76 | 82,05 | 27,21 | 76,04 | 77,82 | 1,78 | 7,00 | 148,20 | 5,29 | 45,27 |
| 2 | ME11 | 330,42 | 87,84 | 27,56 | 76,06 | 87,04 | 10,98 | 7,00 | 174,30 | 5,93 | 47,25 |
| 2 | ME12 | 319,86 | 86,99 | 22,41 | 71,70 | 92,24 | 20,54 | 7,00 | 148,01 | 2,82 | 53,73 |
| 2 | ME12 | 331,20 | 87,75 | 21,56 | 74,12 | 91,07 | 16,95 | 7,00 | 129,88 | 2,40 | 60,79 |
| 2 | ME12 | 315,51 | 86,83 | 17,41 | 74,20 | 86,67 | 12,47 | 7,00 | 107,96 | 2,84 | 65,78 |
| 2 | ME12 | 366,97 | 93,56 | 21,68 | 75,47 | 95,09 | 19,62 | 7,00 | 149,70 | 2,64 | 59,21 |
| 2 | ME12 | 477,64 | 95,01 | 16,85 | 88,87 | 101,63 | 12,76 | 6,00 | 168,56 | 2,22 | 64,71 |
| 2 | ME12 | 459,68 | 98,25 | 21,70 | 76,82 | 96,48 | 19,66 | 7,00 | 190,55 | 2,94 | 58,55 |
| 2 | ME12 | 441,20 | 92,27 | 20,57 | 78,01 | 97,13 | 19,12 | 9,00 | 148,40 | 2,58 | 66,36 |
| 2 | ME12 | 481,27 | 81,50 | 19,52 | 68,85 | 82,20 | 13,35 | 8,00 | 151,54 | 2,18 | 68,51 |
| 2 | ME12 | 274,32 | 80,35 | 22,52 | 68,31 | 88,53 | 20,22 | 6,00 | 104,93 | 2,14 | 61,75 |
| 2 | ME12 | 271,03 | 79,98 | 21,14 | 65,76 | 80,89 | 15,13 | 8,00 | 98,81 | 1,70 | 63,54 |
| 2 | ME13 | 219,86 | 76,61 | 20,95 | 58,72 | 79,71 | 20,99 | 6,00 | 88,31 | 1,92 | 59,83 |
| 2 | ME13 | 265,20 | 76,03 | 18,83 | 65,19 | 88,58 | 23,39 | 5,00 | 87,04 | 1,88 | 67,18 |
| 2 | ME13 | 305,51 | 81,62 | 18,11 | 69,82 | 84,25 | 14,43 | 6,00 | 99,85 | 2,12 | 67,32 |
| 2 | ME13 | 316,97 | 83,14 | 19,76 | 67,89 | 86,66 | 18,77 | 7,00 | 100,86 | 1,60 | 68,18 |
| 2 | ME13 | 257,64 | 79,34 | 15,90 | 67,50 | 86,12 | 18,62 | 6,00 | 83,77 | 2,13 | 67,49 |
| 2 | ME13 | 329,68 | 80,39 | 16,66 | 70,41 | 89,02 | 18,61 | 6,00 | 100,16 | 1,61 | 69,62 |
| 2 | ME13 | 341,20 | 81,82 | 16,71 | 72,27 | 85,45 | 13,18 | 7,00 | 106,80 | 4,59 | 68,70 |
| 2 | ME13 | 281,27 | 83,29 | 16,31 | 70,64 | 88,29 | 17,65 | 6,00 | 94,97 | 2,33 | 66,24 |
| 2 | ME13 | 274,32 | 75,69 | 15,73 | 64,64 | 82,76 | 18,12 | 6,00 | 86,41 | 1,74 | 68,50 |
| 2 | ME13 | 271,03 | 92,23 | 17,38 | 76,43 | 95,90 | 19,47 | 7,00 | 131,02 | 1,67 | 51,66 |
| 2 | ME14 | 368,15 | 92,68 | 20,47 | 73,38 | 89,94 | 16,56 | 7,00 | 151,57 | 3,77 | 58,83 |
| 2 | ME14 | 271,70 | 87,17 | 21,59 | 65,12 | 87,46 | 22,34 | 7,00 | 119,95 | 2,64 | 55,85 |
| 2 | ME14 | 280,50 | 82,05 | 27,28 | 66,40 | 87,36 | 20,96 | 6,00 | 149,60 | 3,89 | 46,67 |
| 2 | ME14 | 367,50 | 91,05 | 21,14 | 79,29 | 96,94 | 17,65 | 6,00 | 152,20 | 3,58 | 58,59 |
| 2 | ME14 | 184,00 | 68,97 | 26,08 | 62,51 | 82,40 | 19,89 | 5,00 | 93,80 | 2,97 | 49,02 |
| 2 | ME14 | 236,76 | 76,21 | 30,84 | 69,68 | 82,54 | 12,86 | 7,00 | 115,27 | 3,25 | 51,31 |
| 2 | ME14 | 308,46 | 81,98 | 23,08 | 71,92 | 93,81 | 21,89 | 6,00 | 136,78 | 3,45 | 55,66 |
| 2 | ME14 | 375,48 | 89,62 | 20,61 | 77,98 | 93,13 | 15,15 | 7,00 | 135,50 | 2,75 | 63,91 |
| 2 | ME14 | 298,32 | 84,43 | 27,31 | 61,56 | 88,24 | 26,68 | 7,00 | 121,85 | 3,22 | 59,15 |
| 2 | ME14 | 204,70 | 76,52 | 23,70 | 66,55 | 82,34 | 15,79 | 6,00 | 97,40 | 3,03 | 52,42 |
| 2 | ME16 | 415,35 | 92,58 | 23,46 | 80,11 | 96,53 | 16,42 | 8,00 | 161,27 | 1,97 | 61,17 |
| 2 | ME16 | 308,58 | 82,73 | 18,16 | 75,79 | 95,89 | 20,10 | 6,00 | 124,98 | 2,06 | 59,50 |
| 2 | ME16 | 319,76 | 84,67 | 18,60 | 75,80 | 94,21 | 18,41 | 6,00 | 115,88 | 1,36 | 63,76 |
| 2 | ME16 | 446,08 | 98,04 | 19,59 | 82,71 | 99,17 | 16,46 | 7,00 | 153,03 | 2,41 | 65,69 |
| 2 | ME16 | 441,06 | 97,09 | 21,89 | 84,45 | 102,47 | 18,02 | 6,00 | 172,86 | 3,17 | 60,81 |
| 2 | ME16 | 386,28 | 93,27 | 20,60 | 75,72 | 91,25 | 15,53 | 7,00 | 134,53 | 3,10 | 65,17 |
| 2 | ME16 | 264,88 | 79,13 | 19,22 | 70,93 | 89,15 | 18,22 | 6,00 | 97,50 | 1,84 | 63,19 |
| 2 | ME16 | 273,71 | 80,42 | 16,53 | 69,12 | 85,02 | 15,90 | 5,00 | 97,23 | 1,73 | 64,48 |
| 2 | ME16 | 375,32 | 92,10 | 20,34 | 78,23 | 97,98 | 19,75 | 7,00 | 141,74 | 2,02 | 62,23 |
| 2 | ME16 | 413,13 | 92,86 | 20,71 | 81,40 | 101,52 | 20,12 | 6,00 | 166,22 | 1,95 | 59,77 |
| 2 | ME17 | 403,35 | 92,58 | 21,87 | 80,29 | 99,99 | 19,70 | 7,00 | 154,28 | 3,26 | 61,75 |
| 2 | ME17 | 248,90 | 78,00 | 20,67 | 66,62 | 84,75 | 18,13 | 8,00 | 108,20 | 3,60 | 56,53 |
| 2 | ME17 | 309,11 | 88,51 | 26,98 | 66,81 | 85,06 | 18,25 | 7,00 | 103,09 | 3,20 | 66,65 |
| 2 | ME17 | 317,12 | 103,89 | 21,90 | 82,00 | 101,10 | 19,10 | 8,00 | 102,67 | 3,23 | 67,62 |
| 2 | ME17 | 400,29 | 92,55 | 21,68 | 80,00 | 97,94 | 17,94 | 7,00 | 173,57 | 3,08 | 56,64 |
| 2 | ME17 | 385,93 | 90,52 | 21,20 | 77,11 | 93,56 | 16,45 | 8,00 | 143,97 | 3,93 | 62,70 |
| 2 | ME17 | 382,15 | 86,82 | 22,24 | 78,32 | 98,06 | 19,74 | 6,00 | 143,80 | 3,75 | 62,37 |
| 2 | ME17 | 377,54 | 91,13 | 25,36 | 78,84 | 91,39 | 12,55 | 8,00 | 159,06 | 3,45 | 57,87 |
| 2 | ME17 | 431,11 | 98,17 | 22,97 | 76,37 | 91,49 | 15,12 | 8,00 | 173,23 | 2,92 | 59,82 |
| 2 | ME17 | 592,00 | 111,25 | 28,93 | 88,34 | 102,24 | 13,90 | 7,00 | 257,59 | 6,97 | 56,49 |
| 2 | ME18 | 313,74 | 84,92 | 20,77 | 72,02 | 88,68 | 16,66 | 7,00 | 105,42 | 2,41 | 66,40 |
| 2 | ME18 | 417,58 | 93,08 | 25,39 | 84,25 | 95,75 | 11,50 | 7,00 | 138,59 | 3,25 | 66,81 |
| 2 | ME18 | 348,92 | 87,34 | 23,50 | 88,96 | 96,84 | 7,88 | 6,00 | 109,64 | 4,48 | 68,58 |
| 2 | ME18 | 283,95 | 86,32 | 20,88 | 67,36 | 80,68 | 13,32 | 7,00 | 105,83 | 3,17 | 62,73 |
| 2 | ME18 | 241,02 | 79,55 | 26,00 | 67,16 | 81,11 | 13,95 | 6,00 | 120,41 | 3,52 | 50,04 |
| 2 | ME18 | 324,43 | 85,59 | 23,04 | 76,71 | 93,61 | 16,90 | 6,00 | 130,02 | 3,94 | 59,92 |
| 2 | ME18 | 268,57 | 79,76 | 18,35 | 73,42 | 90,96 | 17,54 | 6,00 | 86,15 | 2,64 | 67,92 |
| 2 | ME18 | 449,70 | 95,11 | 18,10 | 87,24 | 104,53 | 17,29 | 7,00 | 142,37 | 3,60 | 68,34 |
| 2 | ME18 | 327,26 | 83,05 | 20,09 | 79,13 | 91,14 | 12,01 | 7,00 | 162,10 | 3,09 | 50,47 |
| 2 | ME18 | 270,16 | 80,03 | 21,60 | 71,97 | 90,32 | 18,35 | 6,00 | 109,70 | 3,40 | 59,39 |
| 2 | ME19 | 272,49 | 81,63 | 22,00 | 72,17 | 91,94 | 19,77 | 7,00 | 91,89 | 2,67 | 66,28 |
| 2 | ME19 | 488,85 | 98,53 | 18,80 | 87,27 | 102,37 | 15,10 | 7,00 | 163,19 | 3,63 | 66,62 |
| 2 | ME19 | 504,30 | 95,81 | 20,77 | 86,83 | 100,24 | 13,41 | 7,00 | 150,19 | 2,70 | 70,22 |
| 2 | ME19 | 444,93 | 98,50 | 21,34 | 82,71 | 97,48 | 14,77 | 7,00 | 163,02 | 2,77 | 63,36 |
| 2 | ME19 | 477,75 | 97,75 | 25,21 | 83,23 | 96,58 | 13,35 | 7,00 | 149,03 | 2,99 | 68,81 |
| 2 | ME19 | 346,81 | 86,86 | 20,43 | 74,30 | 94,63 | 20,33 | 7,00 | 118,17 | 3,04 | 65,93 |
| 2 | ME19 | 300,70 | 84,00 | 21,68 | 72,40 | 87,44 | 15,04 | 7,00 | 98,22 | 2,76 | 67,34 |
| 2 | ME19 | 285,65 | 86,72 | 20,10 | 73,70 | 80,39 | 6,69 | 7,00 | 99,56 | 3,32 | 65,15 |
| 2 | ME19 | 416,68 | 95,70 | 19,45 | 82,16 | 98,32 | 16,16 | 7,00 | 139,87 | 3,46 | 66,43 |
| 2 | ME19 | 300,35 | 85,61 | 24,84 | 70,62 | 90,28 | 19,66 | 7,00 | 118,48 | 3,85 | 60,55 |
| 2 | ME20 | 348,38 | 88,21 | 18,61 | 72,61 | 88,08 | 15,47 | 8,00 | 114,34 | 1,28 | 67,18 |
| 2 | ME20 | 443,61 | 100,18 | 20,42 | 76,99 | 98,47 | 21,48 | 9,00 | 153,45 | 1,03 | 65,41 |
| 2 | ME20 | 438,65 | 98,31 | 18,26 | 81,30 | 98,77 | 17,47 | 7,00 | 151,63 | 1,48 | 65,43 |
| 2 | ME20 | 329,70 | 88,04 | 19,66 | 72,51 | 90,24 | 17,73 | 8,00 | 113,54 | 1,01 | 65,56 |
| 2 | ME20 | 367,18 | 93,14 | 17,21 | 74,42 | 91,36 | 16,94 | 7,00 | 126,04 | 1,05 | 65,67 |
| 2 | ME20 | 317,28 | 85,45 | 22,77 | 70,77 | 87,27 | 16,50 | 8,00 | 114,94 | 1,52 | 63,77 |
| 2 | ME20 | 310,72 | 85,39 | 21,77 | 75,38 | 93,53 | 18,15 | 5,00 | 121,33 | 1,54 | 60,95 |
| 2 | ME20 | 490,94 | 96,81 | 23,01 | 84,25 | 96,30 | 12,05 | 9,00 | 164,88 | 1,90 | 66,42 |
| 2 | ME20 | 346,97 | 89,42 | 19,47 | 77,26 | 94,80 | 17,54 | 7,00 | 131,77 | 2,24 | 62,02 |
| 2 | ME20 | 415,39 | 99,38 | 23,25 | 76,07 | 88,47 | 12,40 | 9,00 | 174,52 | 2,96 | 57,99 |
| 2 | ME21 | 497,83 | 97,63 | 20,93 | 87,30 | 104,68 | 17,38 | 6,00 | 215,75 | 2,49 | 56,66 |
| 2 | ME21 | 388,43 | 92,51 | 21,23 | 79,46 | 97,17 | 17,71 | 8,00 | 151,48 | 2,07 | 61,00 |
| 2 | ME21 | 331,63 | 87,40 | 18,30 | 77,11 | 98,04 | 20,93 | 7,00 | 125,64 | 1,73 | 62,11 |
| 2 | ME21 | 205,95 | 74,58 | 18,72 | 62,04 | 82,52 | 20,48 | 6,00 | 95,44 | 2,07 | 53,66 |
| 2 | ME21 | 388,80 | 92,29 | 20,06 | 77,39 | 94,52 | 17,13 | 7,00 | 145,72 | 1,99 | 62,52 |
| 2 | ME21 | 336,60 | 87,23 | 18,72 | 76,12 | 91,98 | 15,86 | 8,00 | 121,20 | 1,10 | 63,99 |
| 2 | ME21 | 296,69 | 82,92 | 19,68 | 71,04 | 90,23 | 19,19 | 6,00 | 130,23 | 2,27 | 56,11 |
| 2 | ME21 | 383,40 | 89,23 | 20,92 | 78,58 | 96,78 | 18,20 | 7,00 | 161,15 | 2,12 | 57,97 |
| 2 | ME21 | 212,13 | 74,73 | 20,72 | 64,18 | 81,97 | 17,79 | 6,00 | 100,94 | 2,72 | 52,42 |
| 2 | ME21 | 331,62 | 83,04 | 18,62 | 83,25 | 94,43 | 11,18 | 6,00 | 141,82 | 2,31 | 57,23 |
| 2 | MA1 | 336,48 | 89,34 | 15,27 | 77,74 | 91,04 | 13,30 | 5,00 | 155,40 | 4,80 | 53,82 |
| 2 | MA1 | 390,00 | 95,84 | 17,11 | 83,96 | 94,44 | 10,48 | 7,00 | 194,80 | 3,74 | 50,05 |
| 2 | MA1 | 410,04 | 81,44 | 20,72 | 73,22 | 91,70 | 18,48 | 6,00 | 180,80 | 6,07 | 55,91 |
| 2 | MA1 | 399,98 | 92,33 | 17,80 | 82,04 | 96,29 | 14,25 | 7,00 | 182,60 | 2,93 | 54,35 |
| 2 | MA1 | 279,95 | 94,23 | 20,88 | 84,85 | 90,17 | 5,32 | 8,00 | 156,60 | 3,00 | 44,06 |
| 2 | MA1 | 318,40 | 100,41 | 19,28 | 89,31 | 101,84 | 12,53 | 7,00 | 190,30 | 5,41 | 40,23 |
| 2 | MA1 | 189,63 | 89,03 | 18,02 | 70,16 | 88,03 | 17,87 | 6,00 | 110,20 | 3,67 | 41,89 |
| 2 | MA1 | 311,22 | 96,59 | 20,23 | 86,07 | 93,03 | 6,96 | 7,00 | 160,20 | 4,60 | 48,53 |
| 2 | MA1 | 451,41 | 73,22 | 19,18 | 60,84 | 72,84 | 12,00 | 6,00 | 136,30 | 3,67 | 69,81 |
| 2 | MA1 | 302,00 | 81,69 | 17,81 | 79,92 | 81,93 | 2,01 | 6,00 | 102,80 | 4,30 | 65,96 |
| 2 | MA2 | 198,96 | 74,60 | 20,97 | 64,56 | 85,39 | 20,83 | 5,00 | 98,05 | 4,63 | 50,72 |
| 2 | MA2 | 281,70 | 83,13 | 18,58 | 76,68 | 99,35 | 22,67 | 7,00 | 125,40 | 3,11 | 55,48 |
| 2 | MA2 | 345,46 | 87,65 | 19,24 | 81,05 | 90,40 | 9,35 | 7,00 | 129,80 | 2,70 | 62,43 |
| 2 | MA2 | 275,70 | 82,39 | 23,73 | 72,84 | 91,08 | 18,24 | 5,00 | 125,90 | 3,34 | 54,33 |
| 2 | MA2 | 442,70 | 98,04 | 19,18 | 87,26 | 101,08 | 13,82 | 7,00 | 137,50 | 4,58 | 68,94 |
| 2 | MA2 | 234,70 | 76,69 | 23,90 | 68,25 | 86,78 | 18,53 | 6,00 | 135,60 | 2,98 | 42,22 |
| 2 | MA2 | 308,60 | 88,23 | 22,24 | 72,40 | 93,72 | 21,32 | 7,00 | 108,80 | 2,47 | 64,74 |
| 2 | MA2 | 222,59 | 77,16 | 19,05 | 68,70 | 88,60 | 19,90 | 6,00 | 105,20 | 2,70 | 52,74 |
| 2 | MA2 | 554,90 | 102,98 | 23,20 | 88,32 | 104,03 | 15,71 | 8,00 | 240,70 | 3,25 | 56,62 |
| 2 | MA2 | 198,96 | 79,68 | 20,63 | 68,30 | 99,82 | 31,52 | 6,00 | 80,60 | 3,66 | 59,49 |
| 2 | MA3 | 376,23 | 87,93 | 20,34 | 76,52 | 94,52 | 18,00 | 6,00 | 150,10 | 3,93 | 60,10 |
| 2 | MA3 | 462,45 | 93,87 | 20,77 | 79,76 | 92,68 | 12,92 | 6,00 | 141,32 | 2,76 | 69,44 |
| 2 | MA3 | 285,26 | 96,30 | 24,64 | 80,75 | 94,01 | 13,26 | 8,00 | 149,94 | 4,07 | 47,44 |
| 2 | MA3 | 406,94 | 94,87 | 20,53 | 79,14 | 97,68 | 18,54 | 7,00 | 132,43 | 3,58 | 67,46 |
| 2 | MA3 | 413,50 | 83,25 | 21,44 | 66,62 | 85,62 | 19,00 | 7,00 | 126,75 | 3,98 | 69,35 |
| 2 | MA3 | 542,08 | 88,50 | 17,53 | 76,99 | 84,11 | 7,12 | 7,00 | 168,96 | 3,43 | 68,83 |
| 2 | MA3 | 287,90 | 67,98 | 20,53 | 64,08 | 78,36 | 14,28 | 6,00 | 95,39 | 4,15 | 66,87 |
| 2 | MA3 | 452,30 | 86,16 | 18,34 | 74,02 | 84,51 | 10,49 | 6,00 | 149,38 | 3,10 | 66,97 |
| 2 | MA3 | 187,29 | 95,56 | 23,79 | 82,15 | 94,62 | 12,47 | 6,00 | 82,46 | 5,77 | 55,97 |
| 2 | MA3 | 302,80 | 81,82 | 19,02 | 73,03 | 87,22 | 14,19 | 8,00 | 113,52 | 2,35 | 62,51 |
| 2 | MA4 | 419,47 | 98,30 | 17,83 | 80,98 | 94,96 | 13,98 | 8,00 | 156,84 | 3,44 | 62,61 |
| 2 | MA4 | 449,81 | 97,30 | 21,69 | 82,28 | 99,68 | 17,40 | 7,00 | 163,57 | 3,69 | 63,64 |
| 2 | MA4 | 209,28 | 76,39 | 17,84 | 64,85 | 82,15 | 17,30 | 6,00 | 80,33 | 2,72 | 61,62 |
| 2 | MA4 | 303,51 | 85,00 | 18,54 | 75,93 | 96,63 | 20,70 | 6,00 | 125,00 | 2,78 | 58,82 |
| 2 | MA4 | 419,25 | 92,87 | 18,23 | 84,74 | 102,53 | 17,79 | 7,00 | 134,24 | 2,33 | 67,98 |
| 2 | MA4 | 461,39 | 100,90 | 20,11 | 81,38 | 94,09 | 12,71 | 8,00 | 179,04 | 4,92 | 61,20 |
| 2 | MA4 | 244,65 | 82,10 | 16,06 | 70,12 | 85,23 | 15,11 | 6,00 | 93,10 | 2,67 | 61,95 |
| 2 | MA4 | 459,69 | 98,41 | 18,95 | 81,66 | 93,04 | 11,38 | 7,00 | 139,01 | 2,41 | 69,76 |
| 2 | MA4 | 271,37 | 81,80 | 20,48 | 72,22 | 79,98 | 7,76 | 7,00 | 92,80 | 2,93 | 65,80 |
| 2 | MA4 | 508,76 | 100,75 | 20,50 | 85,29 | 102,45 | 17,16 | 7,00 | 179,42 | 4,07 | 64,73 |
| 2 | MA5 | 445,00 | 95,67 | 26,56 | 82,82 | 98,20 | 15,38 | 6,00 | 173,29 | 4,83 | 61,06 |
| 2 | MA5 | 338,00 | 88,87 | 22,68 | 77,07 | 93,04 | 15,97 | 8,00 | 102,79 | 2,51 | 69,59 |
| 2 | MA5 | 381,60 | 92,02 | 25,56 | 82,10 | 94,66 | 12,56 | 6,00 | 159,30 | 5,63 | 58,25 |
| 2 | MA5 | 281,90 | 83,82 | 25,56 | 78,00 | 82,22 | 4,22 | 5,00 | 106,80 | 3,07 | 62,11 |
| 2 | MA5 | 399,30 | 88,66 | 17,73 | 78,17 | 92,40 | 14,23 | 6,00 | 119,60 | 4,00 | 70,05 |
| 2 | MA5 | 243,20 | 80,69 | 19,36 | 68,80 | 78,54 | 9,74 | 5,00 | 80,10 | 3,11 | 67,06 |
| 2 | MA5 | 309,90 | 81,30 | 19,32 | 71,56 | 87,21 | 15,65 | 6,00 | 99,50 | 2,89 | 67,89 |
| 2 | MA5 | 479,04 | 100,19 | 18,79 | 89,20 | 101,56 | 12,36 | 6,00 | 176,03 | 3,66 | 63,25 |
| 2 | MA5 | 397,90 | 92,70 | 20,19 | 78,89 | 93,02 | 14,13 | 6,00 | 131,40 | 2,86 | 66,98 |
| 2 | MA5 | 358,60 | 89,36 | 22,66 | 79,97 | 92,98 | 13,01 | 6,00 | 133,35 | 3,83 | 62,81 |
| 2 | BA1 | 382,70 | 90,42 | 20,27 | 77,81 | 96,64 | 18,83 | 7,00 | 154,60 | 2,57 | 59,60 |
| 2 | BA1 | 360,90 | 84,22 | 19,20 | 81,40 | 97,54 | 16,14 | 6,00 | 164,60 | 2,00 | 54,39 |
| 2 | BA1 | 316,60 | 82,40 | 19,42 | 79,70 | 89,12 | 9,42 | 6,00 | 160,80 | 2,66 | 49,21 |
| 2 | BA1 | 332,70 | 91,24 | 14,68 | 79,63 | 90,63 | 11,00 | 6,00 | 180,60 | 6,09 | 45,72 |
| 2 | BA1 | 455,40 | 93,93 | 25,37 | 78,34 | 95,36 | 17,02 | 7,00 | 188,90 | 5,29 | 58,52 |
| 2 | BA1 | 407,70 | 92,94 | 21,98 | 80,11 | 94,31 | 14,20 | 6,00 | 160,20 | 4,53 | 60,71 |
| 2 | BA1 | 421,80 | 94,68 | 27,93 | 85,25 | 98,05 | 12,80 | 7,00 | 174,78 | 3,22 | 58,56 |
| 2 | BA1 | 365,80 | 91,83 | 26,60 | 79,64 | 93,82 | 14,18 | 6,00 | 216,60 | 4,21 | 40,79 |
| 2 | BA1 | 365,31 | 93,51 | 23,49 | 63,36 | 89,37 | 26,01 | 6,00 | 200,40 | 2,82 | 45,14 |
| 2 | BA1 | 286,20 | 75,53 | 16,44 | 70,92 | 86,67 | 15,75 | 6,00 | 130,90 | 4,00 | 54,26 |
| 2 | VA1 | 442,05 | 94,12 | 22,46 | 83,25 | 97,04 | 13,79 | 5,00 | 198,71 | 4,26 | 55,05 |
| 2 | VA1 | 346,43 | 90,20 | 23,95 | 76,91 | 95,63 | 18,72 | 7,00 | 151,64 | 4,39 | 56,23 |
| 2 | VA1 | 330,60 | 87,44 | 20,57 | 79,18 | 87,91 | 8,73 | 7,00 | 120,10 | 2,94 | 63,67 |
| 2 | VA1 | 335,09 | 89,17 | 18,07 | 72,67 | 85,00 | 12,33 | 5,00 | 151,17 | 3,70 | 54,89 |
| 2 | VA1 | 363,36 | 90,38 | 19,00 | 77,70 | 94,60 | 16,90 | 7,00 | 124,02 | 3,23 | 65,87 |
| 2 | VA1 | 239,57 | 72,98 | 20,73 | 66,55 | 84,55 | 18,00 | 6,00 | 106,72 | 3,67 | 55,45 |
| 2 | VA1 | 321,58 | 83,38 | 19,60 | 73,20 | 92,18 | 18,98 | 7,00 | 114,97 | 2,97 | 64,25 |
| 2 | VA1 | 376,11 | 90,79 | 16,93 | 79,08 | 94,64 | 15,56 | 7,00 | 118,90 | 2,82 | 68,39 |
| 2 | VA1 | 319,26 | 86,58 | 18,21 | 70,39 | 85,63 | 15,24 | 6,00 | 108,36 | 2,33 | 66,06 |
| 2 | VA1 | 269,83 | 81,13 | 24,61 | 74,01 | 91,19 | 17,18 | 7,00 | 117,66 | 3,03 | 56,39 |
| 2 | MC1 | 473,50 | 97,09 | 21,48 | 89,00 | 102,11 | 13,11 | 7,00 | 236,97 | 3,72 | 49,95 |
| 2 | MC1 | 368,28 | 92,90 | 16,35 | 80,67 | 97,68 | 17,01 | 7,00 | 167,64 | 3,53 | 54,48 |
| 2 | MC1 | 277,00 | 80,23 | 18,68 | 73,94 | 88,27 | 14,33 | 6,00 | 118,23 | 2,54 | 57,32 |
| 2 | MC1 | 329,00 | 87,92 | 22,32 | 79,01 | 91,18 | 12,17 | 5,00 | 185,46 | 2,67 | 43,63 |
| 2 | MC1 | 360,17 | 89,09 | 26,01 | 80,00 | 94,92 | 14,92 | 6,00 | 136,78 | 4,12 | 62,02 |
| 2 | MC1 | 368,15 | 88,82 | 17,40 | 84,69 | 97,14 | 12,45 | 6,00 | 171,80 | 3,36 | 53,33 |
| 2 | MC1 | 343,86 | 86,66 | 20,05 | 76,42 | 91,48 | 15,06 | 7,00 | 174,20 | 2,84 | 49,34 |
| 2 | MC1 | 321,52 | 86,00 | 21,90 | 80,66 | 92,81 | 12,15 | 6,00 | 172,40 | 4,22 | 46,38 |
| 2 | MC1 | 484,55 | 100,32 | 20,02 | 86,51 | 95,46 | 8,95 | 8,00 | 156,70 | 3,08 | 67,66 |
| 2 | MC1 | 313,03 | 85,97 | 17,07 | 75,44 | 91,56 | 16,12 | 7,00 | 152,60 | 2,93 | 51,25 |
| 2 | MO2 | 453,44 | 92,39 | 16,41 | 81,01 | 98,82 | 17,81 | 7,00 | 144,47 | 2,91 | 68,14 |
| 2 | MO2 | 338,71 | 86,69 | 16,68 | 73,87 | 95,91 | 22,04 | 8,00 | 109,77 | 2,67 | 67,59 |
| 2 | MO2 | 368,83 | 91,50 | 26,27 | 71,98 | 92,82 | 20,84 | 7,00 | 146,60 | 3,34 | 60,25 |
| 2 | MO2 | 387,82 | 91,28 | 18,19 | 76,78 | 90,38 | 13,60 | 8,00 | 121,70 | 2,79 | 68,62 |
| 2 | MO2 | 426,50 | 95,30 | 23,15 | 79,70 | 96,40 | 16,70 | 8,00 | 152,07 | 3,38 | 64,34 |
| 2 | MO2 | 336,70 | 83,11 | 19,19 | 69,20 | 84,34 | 15,14 | 7,00 | 127,06 | 2,80 | 62,26 |
| 2 | MO2 | 481,14 | 98,34 | 19,75 | 86,18 | 102,03 | 15,85 | 7,00 | 190,18 | 2,50 | 60,47 |
| 2 | MO2 | 400,00 | 91,09 | 17,98 | 82,43 | 95,43 | 13,00 | 7,00 | 128,85 | 2,81 | 67,79 |
| 2 | MO2 | 477,80 | 101,90 | 19,60 | 82,81 | 96,14 | 13,33 | 7,00 | 172,23 | 4,55 | 63,95 |
| 2 | MO2 | 472,25 | 97,57 | 22,41 | 83,15 | 101,13 | 17,98 | 7,00 | 178,32 | 2,86 | 62,24 |
| 2 | MO3 | 332,20 | 87,21 | 21,19 | 74,54 | 89,25 | 14,71 | 6,00 | 119,90 | 1,10 | 63,91 |
| 2 | MO3 | 472,30 | 100,06 | 21,82 | 88,09 | 104,65 | 16,56 | 7,00 | 150,65 | 0,82 | 68,10 |
| 2 | MO3 | 447,10 | 95,89 | 19,12 | 83,62 | 105,58 | 21,96 | 8,00 | 167,92 | 2,64 | 62,44 |
| 2 | MO3 | 414,20 | 93,41 | 19,13 | 82,19 | 101,51 | 19,32 | 8,00 | 130,46 | 1,97 | 68,50 |
| 2 | MO3 | 459,70 | 99,58 | 21,35 | 79,65 | 95,93 | 16,28 | 8,00 | 185,75 | 2,93 | 59,59 |
| 2 | MO3 | 414,60 | 93,16 | 19,16 | 80,70 | 94,08 | 13,38 | 8,00 | 187,30 | 3,40 | 54,82 |
| 2 | MO3 | 404,30 | 93,28 | 22,59 | 77,25 | 90,80 | 13,55 | 8,00 | 147,40 | 2,73 | 63,54 |
| 2 | MO3 | 384,50 | 94,45 | 23,37 | 77,93 | 91,93 | 14,00 | 6,00 | 140,47 | 1,86 | 63,47 |
| 2 | MO3 | 450,20 | 95,45 | 22,50 | 86,02 | 100,21 | 14,19 | 7,00 | 179,60 | 2,59 | 60,11 |
| 2 | MO3 | 454,20 | 93,37 | 20,20 | 81,51 | 95,80 | 14,29 | 7,00 | 168,45 | 2,98 | 62,91 |
| 2 | MO4 | 456,30 | 94,23 | 18,25 | 76,95 | 90,99 | 14,04 | 7,00 | 173,90 | 1,24 | 61,89 |
| 2 | MO4 | 346,60 | 83,99 | 17,65 | 77,65 | 83,81 | 6,16 | 6,00 | 116,13 | 1,80 | 66,49 |
| 2 | MO4 | 396,10 | 94,04 | 18,05 | 78,67 | 97,59 | 18,92 | 6,00 | 161,41 | 2,32 | 59,25 |
| 2 | MO4 | 432,10 | 95,52 | 17,35 | 80,32 | 104,59 | 24,27 | 7,00 | 149,73 | 2,84 | 65,35 |
| 2 | MO4 | 448,20 | 98,42 | 24,47 | 80,73 | 91,73 | 11,00 | 7,00 | 162,73 | 2,61 | 63,69 |
| 2 | MO4 | 396,70 | 91,12 | 22,47 | 75,62 | 93,89 | 18,27 | 6,00 | 151,87 | 3,29 | 61,72 |
| 2 | MO4 | 378,10 | 95,08 | 26,00 | 76,24 | 94,78 | 18,54 | 8,00 | 144,96 | 2,06 | 61,66 |
| 2 | MO4 | 430,10 | 90,41 | 23,11 | 82,88 | 96,30 | 13,42 | 7,00 | 147,62 | 4,11 | 65,68 |
| 2 | MO4 | 296,80 | 84,04 | 19,75 | 72,95 | 84,54 | 11,59 | 7,00 | 103,34 | 0,95 | 65,18 |
| 2 | MO4 | 314,00 | 83,39 | 19,47 | 68,50 | 77,28 | 8,78 | 6,00 | 122,20 | 1,44 | 61,08 |
| 2 | MO5 | 412,90 | 89,42 | 29,91 | 73,28 | 92,90 | 19,62 | 7,00 | 153,54 | 1,61 | 62,81 |
| 2 | MO5 | 377,60 | 89,38 | 24,30 | 74,89 | 84,31 | 9,42 | 6,00 | 139,40 | 0,97 | 63,08 |
| 2 | MO5 | 418,20 | 94,53 | 22,19 | 79,68 | 94,44 | 14,76 | 8,00 | 156,27 | 1,66 | 62,63 |
| 2 | MO5 | 358,50 | 86,26 | 29,71 | 72,39 | 82,44 | 10,05 | 7,00 | 117,31 | 1,60 | 67,28 |
| 2 | MO5 | 345,10 | 89,08 | 18,17 | 73,57 | 90,44 | 16,87 | 7,00 | 116,70 | 2,06 | 66,18 |
| 2 | MO5 | 399,30 | 88,43 | 18,18 | 79,30 | 90,23 | 10,93 | 7,00 | 132,29 | 1,63 | 66,87 |
| 2 | MO5 | 428,90 | 96,37 | 19,08 | 79,31 | 95,77 | 16,46 | 8,00 | 140,25 | 2,50 | 67,30 |
| 2 | MO5 | 287,10 | 81,16 | 19,32 | 72,87 | 85,40 | 12,53 | 7,00 | 93,94 | 1,29 | 67,28 |
| 2 | MO5 | 378,20 | 90,11 | 17,60 | 77,48 | 89,80 | 12,32 | 7,00 | 111,06 | 1,20 | 70,63 |
| 2 | MO5 | 358,60 | 88,58 | 20,43 | 75,61 | 87,52 | 11,91 | 6,00 | 110,95 | 1,44 | 69,06 |
| 2 | MO6 | 455,20 | 95,93 | 17,44 | 89,20 | 104,06 | 14,86 | 7,00 | 155,34 | 2,76 | 65,87 |
| 2 | MO6 | 263,03 | 81,75 | 18,10 | 69,84 | 83,63 | 13,79 | 7,00 | 98,49 | 3,50 | 62,56 |
| 2 | MO6 | 453,54 | 97,85 | 26,03 | 81,60 | 99,04 | 17,44 | 7,00 | 162,35 | 3,80 | 64,20 |
| 2 | MO6 | 421,66 | 94,26 | 23,76 | 75,82 | 95,10 | 19,28 | 6,00 | 148,87 | 3,71 | 64,69 |
| 2 | MO6 | 315,71 | 84,24 | 21,72 | 67,80 | 84,70 | 16,90 | 7,00 | 123,17 | 4,80 | 60,99 |
| 2 | MO6 | 499,18 | 98,88 | 20,21 | 88,75 | 103,23 | 14,48 | 7,00 | 174,48 | 4,55 | 65,05 |
| 2 | MO6 | 406,25 | 93,53 | 18,39 | 79,00 | 95,06 | 16,06 | 6,00 | 159,31 | 4,28 | 60,79 |
| 2 | MO6 | 489,95 | 102,14 | 27,94 | 81,78 | 95,18 | 13,40 | 7,00 | 196,46 | 5,97 | 59,90 |
| 2 | MO6 | 385,11 | 94,23 | 22,80 | 75,27 | 90,55 | 15,28 | 6,00 | 132,18 | 3,44 | 65,68 |
| 2 | MO6 | 362,46 | 90,80 | 19,68 | 78,38 | 96,21 | 17,83 | 7,00 | 128,89 | 2,76 | 64,44 |
| 2 | AB1 | 459,84 | 96,59 | 16,28 | 85,79 | 96,10 | 10,31 | 7,00 | 179,45 | 2,20 | 60,98 |
| 2 | AB1 | 364,04 | 88,20 | 18,63 | 78,93 | 96,24 | 17,31 | 7,00 | 148,08 | 1,79 | 59,32 |
| 2 | AB1 | 324,51 | 88,89 | 17,36 | 75,36 | 96,53 | 21,17 | 7,00 | 147,36 | 2,53 | 54,59 |
| 2 | AB1 | 419,20 | 94,75 | 16,51 | 85,00 | 99,90 | 14,90 | 6,00 | 152,61 | 2,32 | 63,59 |
| 2 | AB1 | 385,57 | 91,45 | 17,34 | 85,03 | 99,76 | 14,73 | 8,00 | 164,30 | 2,29 | 57,39 |
| 2 | AB1 | 266,94 | 78,68 | 18,51 | 69,67 | 89,66 | 19,99 | 6,00 | 118,04 | 2,50 | 55,78 |
| 2 | AB1 | 322,18 | 89,48 | 18,78 | 71,95 | 88,98 | 17,03 | 6,00 | 147,40 | 2,46 | 54,25 |
| 2 | AB1 | 464,11 | 99,08 | 16,66 | 86,64 | 105,69 | 19,05 | 8,00 | 177,46 | 1,83 | 61,76 |
| 2 | AB1 | 241,52 | 80,01 | 17,13 | 66,06 | 88,20 | 22,14 | 7,00 | 90,11 | 2,07 | 62,69 |
| 2 | AB1 | 454,18 | 98,52 | 20,72 | 79,81 | 97,00 | 17,19 | 8,00 | 167,27 | 1,70 | 63,17 |
| 2 | PB1 | 420,90 | 95,76 | 19,30 | 75,04 | 94,40 | 19,36 | 7,00 | 185,61 | 1,29 | 55,90 |
| 2 | PB1 | 361,30 | 88,47 | 19,30 | 75,05 | 93,85 | 18,80 | 7,00 | 143,20 | 2,79 | 60,37 |
| 2 | PB1 | 429,20 | 93,53 | 21,20 | 81,07 | 101,53 | 20,46 | 7,00 | 191,90 | 1,59 | 55,29 |
| 2 | PB1 | 315,97 | 84,27 | 18,03 | 68,10 | 88,79 | 20,69 | 7,00 | 160,98 | 1,92 | 49,05 |
| 2 | PB1 | 390,59 | 92,50 | 18,27 | 77,80 | 94,80 | 17,00 | 6,00 | 178,25 | 2,05 | 54,36 |
| 2 | PB1 | 384,89 | 96,26 | 19,82 | 70,61 | 89,90 | 19,29 | 6,00 | 224,76 | 2,93 | 41,60 |
| 2 | PTB1 | 456,91 | 93,71 | 22,93 | 85,05 | 106,65 | 21,60 | 6,00 | 216,40 | 2,68 | 52,64 |
| 2 | PTB1 | 494,01 | 103,27 | 18,20 | 83,45 | 103,45 | 20,00 | 8,00 | 207,72 | 2,18 | 57,95 |
| 2 | PTB1 | 360,73 | 94,80 | 18,25 | 79,01 | 96,85 | 17,84 | 7,00 | 165,34 | 3,93 | 54,17 |
| 2 | PTB1 | 336,23 | 88,14 | 19,01 | 74,82 | 93,85 | 19,03 | 7,00 | 181,67 | 2,84 | 45,97 |
| 2 | PTB1 | 356,84 | 87,83 | 17,60 | 79,26 | 99,89 | 20,63 | 6,00 | 127,18 | 1,51 | 64,36 |
| 2 | PTB1 | 355,38 | 88,10 | 14,83 | 81,23 | 103,03 | 21,80 | 6,00 | 173,40 | 1,67 | 51,21 |
| 2 | PTB1 | 526,95 | 102,85 | 18,67 | 90,20 | 104,60 | 14,40 | 6,00 | 184,49 | 1,76 | 64,99 |
| 2 | PTB1 | 464,89 | 99,17 | 16,46 | 87,98 | 105,14 | 17,16 | 7,00 | 174,26 | 1,08 | 62,52 |
| 2 | PTB1 | 385,97 | 93,28 | 19,00 | 77,90 | 95,51 | 17,61 | 6,00 | 182,37 | 2,06 | 52,75 |
| 2 | PTB1 | 390,78 | 90,88 | 17,16 | 85,21 | 103,56 | 18,35 | 6,00 | 158,56 | 1,69 | 59,42 |
| 2 | PTB1 | 353,90 | 90,13 | 16,03 | 81,37 | 95,95 | 14,58 | 7,00 | 134,12 | 2,45 | 62,10 |
| 2 | PTB1 | 267,79 | 81,53 | 16,25 | 75,03 | 92,60 | 17,57 | 6,00 | 118,80 | 2,15 | 55,64 |
| 2 | PTB1 | 563,82 | 106,03 | 18,67 | 92,98 | 111,47 | 18,49 | 7,00 | 227,95 | 2,19 | 59,57 |
| 2 | PTB1 | 375,38 | 93,50 | 16,11 | 81,62 | 100,40 | 18,78 | 7,00 | 143,08 | 1,49 | 61,88 |
| 2 | SFB1 | 483,45 | 106,53 | 18,06 | 81,14 | 102,89 | 21,75 | 7,00 | 165,00 | 1,90 | 65,87 |
| 2 | SFB1 | 315,37 | 84,08 | 18,80 | 72,76 | 88,78 | 16,02 | 6,00 | 99,34 | 0,82 | 68,50 |
| 2 | SFB1 | 456,83 | 100,71 | 21,38 | 81,91 | 102,50 | 20,59 | 7,00 | 154,09 | 1,99 | 66,27 |
| 2 | SFB1 | 417,60 | 93,95 | 20,52 | 79,52 | 99,22 | 19,70 | 6,00 | 126,82 | 2,11 | 69,63 |
| 2 | SFB1 | 471,60 | 98,35 | 16,22 | 91,28 | 106,21 | 14,93 | 6,00 | 151,45 | 1,13 | 67,89 |
| 2 | SFB1 | 415,92 | 98,51 | 16,63 | 79,60 | 100,20 | 20,60 | 6,00 | 129,86 | 1,29 | 68,78 |
| 2 | SFB1 | 262,71 | 79,69 | 18,62 | 69,26 | 92,00 | 22,74 | 5,00 | 99,22 | 0,97 | 62,23 |
| 2 | SFB1 | 228,60 | 76,74 | 15,90 | 61,83 | 85,32 | 23,49 | 6,00 | 92,27 | 1,72 | 59,64 |
| 2 | SFB1 | 252,80 | 75,19 | 20,56 | 69,49 | 90,50 | 21,01 | 5,00 | 115,65 | 2,85 | 54,25 |
| 2 | SFB1 | 303,74 | 84,79 | 21,14 | 71,33 | 90,43 | 19,10 | 6,00 | 115,20 | 2,71 | 62,07 |
| 2 | PTO2 | 312,24 | 93,54 | 19,40 | 80,54 | 95,28 | 14,74 | 5,00 | 140,04 | 4,56 | 55,15 |
| 2 | PTO2 | 346,48 | 100,51 | 19,87 | 88,20 | 110,72 | 22,52 | 7,00 | 159,01 | 5,20 | 54,11 |
| 2 | PTO2 | 545,19 | 85,91 | 21,06 | 72,91 | 94,64 | 21,73 | 6,00 | 166,60 | 6,16 | 69,44 |
| 2 | PTO2 | 423,04 | 81,62 | 18,32 | 77,56 | 93,14 | 15,58 | 6,00 | 131,14 | 4,33 | 69,00 |
| 2 | PTO2 | 446,67 | 106,69 | 18,42 | 88,65 | 103,68 | 15,03 | 7,00 | 202,59 | 2,93 | 54,64 |
| 2 | PTO2 | 610,40 | 93,68 | 17,88 | 83,07 | 100,66 | 17,59 | 6,00 | 192,42 | 2,30 | 68,48 |
| 2 | PTO2 | 301,80 | 97,15 | 20,27 | 87,69 | 106,64 | 18,95 | 7,00 | 157,60 | 1,89 | 47,78 |
| 2 | PTO2 | 653,40 | 105,49 | 18,59 | 96,02 | 103,95 | 7,93 | 7,00 | 239,67 | 3,78 | 63,32 |
| 2 | PTO2 | 253,56 | 87,67 | 16,15 | 72,76 | 99,16 | 26,40 | 5,00 | 109,59 | 2,17 | 56,78 |
| 2 | PTO2 | 360,80 | 111,53 | 17,14 | 96,33 | 112,65 | 16,32 | 8,00 | 173,50 | 2,70 | 51,91 |
| 2 | PTO3 | 450,60 | 114,13 | 17,63 | 93,16 | 104,51 | 11,35 | 6,00 | 227,69 | 3,60 | 49,47 |
| 2 | PTO3 | 589,60 | 90,16 | 17,24 | 73,93 | 93,94 | 20,01 | 7,00 | 191,16 | 2,66 | 67,58 |
| 2 | PTO3 | 528,50 | 99,25 | 19,76 | 89,11 | 107,17 | 18,06 | 5,00 | 169,85 | 3,47 | 67,86 |
| 2 | PTO3 | 223,70 | 103,75 | 22,62 | 88,67 | 105,84 | 17,17 | 6,00 | 102,60 | 4,77 | 54,14 |
| 2 | PTO3 | 522,40 | 89,82 | 14,00 | 73,28 | 85,23 | 11,95 | 6,00 | 254,60 | 2,50 | 51,26 |
| 2 | PTO3 | 529,10 | 99,42 | 16,00 | 89,23 | 96,14 | 6,91 | 5,00 | 160,70 | 3,45 | 69,63 |
| 2 | PTO3 | 275,10 | 92,52 | 17,57 | 89,89 | 106,45 | 16,56 | 7,00 | 133,50 | 3,33 | 51,47 |
| 2 | PTO3 | 354,54 | 83,51 | 14,20 | 71,01 | 82,28 | 11,27 | 6,00 | 113,90 | 2,71 | 67,87 |
| 2 | PTO3 | 324,60 | 109,25 | 15,30 | 98,07 | 108,96 | 10,89 | 6,00 | 135,30 | 4,88 | 58,32 |
| 2 | PTO3 | 678,90 | 81,91 | 22,48 | 66,19 | 84,25 | 18,06 | 6,00 | 272,10 | 2,74 | 59,92 |
| 2 | PTO4 | 413,80 | 98,41 | 19,06 | 75,49 | 89,99 | 14,50 | 5,00 | 153,70 | 3,06 | 62,86 |
| 2 | PTO4 | 587,20 | 106,83 | 16,11 | 87,05 | 101,41 | 14,36 | 7,00 | 170,49 | 1,92 | 70,97 |
| 2 | PTO4 | 532,10 | 111,91 | 19,82 | 87,77 | 108,85 | 21,08 | 6,00 | 197,58 | 3,45 | 62,87 |
| 2 | PTO4 | 435,40 | 97,40 | 15,81 | 87,74 | 102,66 | 14,92 | 6,00 | 158,90 | 3,18 | 63,50 |
| 2 | PTO4 | 393,70 | 95,17 | 18,09 | 79,96 | 88,06 | 8,10 | 6,00 | 136,20 | 2,26 | 65,41 |
| 2 | PTO4 | 419,30 | 98,51 | 19,68 | 85,76 | 96,07 | 10,31 | 6,00 | 137,50 | 2,33 | 67,21 |
| 2 | PTO4 | 277,90 | 82,69 | 19,37 | 71,12 | 87,50 | 16,38 | 5,00 | 106,20 | 2,20 | 61,78 |
| 2 | PTO4 | 403,11 | 100,50 | 20,69 | 75,45 | 96,70 | 21,25 | 6,00 | 120,20 | 2,02 | 70,18 |
| 2 | PTO4 | 509,70 | 100,20 | 18,75 | 88,50 | 107,24 | 18,74 | 7,00 | 154,30 | 4,23 | 69,73 |
| 2 | PTO4 | 319,20 | 88,89 | 19,30 | 77,68 | 81,77 | 4,09 | 6,00 | 133,80 | 2,30 | 58,08 |
| 2 | PTO5 | 319,20 | 88,02 | 17,55 | 89,39 | 93,61 | 4,22 | 5,00 | 125,15 | 2,91 | 60,79 |
| 2 | PTO5 | 388,30 | 90,28 | 18,03 | 78,00 | 91,25 | 13,25 | 7,00 | 143,26 | 4,37 | 63,11 |
| 2 | PTO5 | 456,80 | 88,27 | 22,02 | 86,65 | 96,81 | 10,16 | 6,00 | 167,23 | 5,26 | 63,39 |
| 2 | PTO5 | 366,50 | 86,16 | 19,41 | 68,76 | 88,43 | 19,67 | 7,00 | 145,42 | 3,58 | 60,32 |
| 2 | PTO5 | 294,60 | 96,20 | 19,00 | 82,13 | 94,74 | 12,61 | 6,00 | 137,20 | 3,25 | 53,43 |
| 2 | PTO5 | 435,70 | 83,66 | 21,61 | 76,82 | 87,53 | 10,71 | 6,00 | 196,75 | 6,14 | 54,84 |
| 2 | PTO5 | 497,80 | 97,97 | 19,97 | 81,69 | 92,21 | 10,52 | 6,00 | 186,79 | 5,98 | 62,48 |
| 2 | PTO5 | 379,10 | 100,11 | 15,55 | 85,81 | 97,10 | 11,29 | 6,00 | 167,21 | 3,58 | 55,89 |
| 2 | PTO5 | 400,00 | 88,29 | 16,90 | 80,94 | 97,63 | 16,69 | 6,00 | 173,72 | 4,73 | 56,57 |
| 2 | PTO5 | 388,70 | 92,31 | 15,78 | 81,07 | 102,60 | 21,53 | 6,00 | 169,30 | 4,16 | 56,44 |
| 2 | PTO6 | 381,95 | 92,81 | 16,66 | 78,66 | 101,28 | 22,62 | 7,00 | 125,59 | 2,58 | 67,12 |
| 2 | PTO6 | 536,45 | 100,87 | 18,63 | 88,49 | 100,07 | 11,58 | 7,00 | 170,71 | 1,79 | 68,18 |
| 2 | PTO6 | 300,80 | 90,23 | 23,54 | 67,64 | 88,73 | 21,09 | 6,00 | 109,42 | 4,28 | 63,62 |
| 2 | PTO6 | 443,31 | 98,82 | 20,64 | 84,34 | 103,39 | 19,05 | 6,00 | 137,18 | 2,95 | 69,06 |
| 2 | PTO6 | 422,93 | 100,76 | 20,85 | 80,72 | 102,73 | 22,01 | 7,00 | 138,92 | 3,37 | 67,15 |
| 2 | PTO6 | 427,43 | 95,52 | 18,66 | 84,03 | 93,63 | 9,60 | 7,00 | 141,30 | 3,90 | 66,94 |
| 2 | PTO6 | 406,23 | 95,56 | 20,83 | 82,50 | 103,58 | 21,08 | 6,00 | 126,39 | 3,52 | 68,89 |
| 2 | PTO6 | 321,16 | 89,53 | 21,01 | 72,10 | 87,42 | 15,32 | 7,00 | 98,83 | 2,69 | 69,23 |
| 2 | PTO6 | 563,75 | 108,97 | 18,81 | 87,98 | 108,25 | 20,27 | 6,00 | 184,38 | 3,08 | 67,29 |
| 2 | PTO6 | 341,70 | 90,46 | 18,33 | 77,11 | 95,09 | 17,98 | 6,00 | 102,54 | 2,56 | 69,99 |
| 2 | PTO7 | 544,74 | 103,49 | 15,82 | 86,58 | 97,97 | 11,39 | 8,00 | 167,32 | 0,78 | 69,28 |
| 2 | PTO7 | 635,30 | 107,52 | 20,04 | 91,92 | 107,94 | 16,02 | 6,00 | 180,24 | 2,07 | 71,63 |
| 2 | PTO7 | 484,60 | 100,09 | 15,57 | 77,09 | 97,56 | 20,47 | 7,00 | 144,26 | 0,75 | 70,23 |
| 2 | PTO7 | 752,60 | 114,35 | 16,68 | 99,31 | 109,30 | 9,99 | 6,00 | 224,19 | 1,24 | 70,21 |
| 2 | PTO7 | 545,36 | 107,37 | 17,89 | 89,22 | 101,45 | 12,23 | 6,00 | 172,57 | 1,30 | 68,36 |
| 2 | PTO7 | 596,14 | 100,09 | 18,27 | 85,54 | 107,01 | 21,47 | 7,00 | 178,34 | 1,64 | 70,08 |
| 2 | PTO7 | 361,61 | 88,46 | 18,76 | 75,08 | 94,73 | 19,65 | 7,00 | 137,13 | 2,31 | 62,08 |
| 2 | PTO7 | 527,17 | 101,97 | 20,09 | 82,23 | 93,91 | 11,68 | 7,00 | 184,21 | 2,35 | 65,06 |
| 2 | PTO7 | 390,34 | 92,13 | 17,31 | 76,24 | 90,47 | 14,23 | 6,00 | 132,40 | 1,53 | 66,08 |
| 2 | PTO7 | 620,80 | 107,96 | 16,65 | 87,56 | 109,34 | 21,78 | 6,00 | 202,62 | 2,45 | 67,36 |
| 2 | PTO8 | 408,13 | 92,10 | 18,09 | 85,28 | 100,79 | 15,51 | 6,00 | 136,41 | 1,32 | 66,58 |
| 2 | PTO8 | 541,30 | 101,76 | 20,31 | 93,29 | 109,09 | 15,80 | 7,00 | 156,37 | 0,77 | 71,11 |
| 2 | PTO8 | 533,20 | 102,23 | 19,55 | 87,45 | 108,94 | 21,49 | 8,00 | 191,08 | 2,50 | 64,16 |
| 2 | PTO8 | 541,28 | 105,18 | 16,60 | 83,32 | 100,55 | 17,23 | 5,00 | 167,71 | 1,96 | 69,02 |
| 2 | PTO8 | 334,83 | 84,29 | 15,57 | 75,05 | 91,05 | 16,00 | 7,00 | 122,19 | 1,13 | 63,51 |
| 2 | PTO8 | 350,32 | 85,84 | 19,56 | 75,74 | 94,94 | 19,20 | 7,00 | 123,88 | 1,02 | 64,64 |
| 2 | PTO8 | 250,60 | 76,94 | 15,29 | 63,28 | 73,71 | 10,43 | 7,00 | 100,27 | 0,85 | 59,99 |
| 2 | PTO8 | 280,70 | 85,73 | 20,35 | 68,86 | 91,13 | 22,27 | 7,00 | 92,77 | 1,40 | 66,95 |
| 2 | PTO8 | 238,90 | 76,10 | 22,50 | 66,58 | 88,20 | 21,62 | 5,00 | 98,07 | 2,43 | 58,95 |
| 2 | PTO8 | 196,40 | 72,16 | 19,17 | 60,34 | 81,78 | 21,44 | 5,00 | 92,23 | 1,82 | 53,04 |
| 2 | PTO9 | 468,03 | 97,47 | 18,53 | 85,56 | 105,04 | 19,48 | 7,00 | 184,44 | 1,36 | 60,59 |
| 2 | PTO9 | 253,76 | 74,40 | 21,62 | 68,79 | 93,35 | 24,56 | 6,00 | 123,60 | 1,87 | 51,29 |
| 2 | PTO9 | 580,32 | 104,05 | 25,90 | 92,31 | 117,04 | 24,73 | 6,00 | 242,76 | 2,10 | 58,17 |
| 2 | PTO9 | 295,44 | 88,17 | 24,62 | 68,83 | 89,57 | 20,74 | 6,00 | 114,88 | 1,06 | 61,12 |
| 2 | PTO9 | 315,16 | 83,54 | 18,94 | 72,35 | 93,12 | 20,77 | 7,00 | 117,16 | 0,86 | 62,83 |
| 2 | PTO9 | 536,15 | 100,67 | 28,06 | 90,94 | 109,55 | 18,61 | 7,00 | 209,03 | 2,24 | 61,01 |
| 2 | PTO9 | 314,83 | 82,91 | 20,16 | 74,24 | 94,30 | 20,06 | 7,00 | 99,62 | 1,26 | 68,36 |
| 2 | PTO9 | 429,96 | 96,06 | 30,17 | 82,41 | 99,22 | 16,81 | 6,00 | 205,48 | 1,55 | 52,21 |
| 2 | PTO9 | 473,24 | 94,57 | 26,16 | 80,90 | 99,91 | 19,01 | 7,00 | 195,80 | 2,47 | 58,63 |
| 2 | PTO9 | 380,79 | 90,20 | 17,20 | 79,09 | 96,93 | 17,84 | 7,00 | 146,05 | 2,21 | 61,65 |
| 2 | PDO2 | 451,40 | 89,59 | 16,34 | 89,21 | 101,83 | 12,62 | 7,00 | 169,20 | 1,86 | 62,52 |
| 2 | PDO2 | 331,50 | 85,68 | 19,68 | 78,30 | 87,53 | 9,23 | 6,00 | 115,36 | 1,85 | 65,20 |
| 2 | PDO2 | 434,60 | 96,30 | 18,80 | 77,13 | 90,90 | 13,77 | 7,00 | 172,25 | 1,65 | 60,37 |
| 2 | PDO2 | 404,77 | 95,40 | 19,00 | 68,00 | 88,90 | 20,90 | 7,00 | 183,10 | 2,74 | 54,76 |
| 2 | PDO2 | 449,46 | 89,90 | 19,40 | 89,66 | 99,46 | 9,80 | 7,00 | 200,50 | 2,83 | 55,39 |
| 2 | PDO2 | 422,60 | 99,12 | 15,03 | 87,00 | 95,67 | 8,67 | 8,00 | 190,30 | 2,24 | 54,97 |
| 2 | PDO2 | 342,07 | 91,01 | 16,46 | 75,20 | 80,86 | 5,66 | 6,00 | 166,80 | 3,95 | 51,24 |
| 2 | PDO2 | 646,00 | 108,60 | 15,50 | 102,60 | 118,60 | 16,00 | 6,00 | 192,80 | 2,38 | 70,15 |
| 2 | PDO2 | 356,30 | 85,60 | 15,30 | 83,24 | 91,43 | 8,19 | 6,00 | 162,20 | 2,88 | 54,48 |
| 2 | PDO2 | 447,30 | 92,80 | 20,36 | 84,51 | 84,77 | 0,26 | 7,00 | 227,60 | 3,31 | 49,12 |
| 2 | CRO1 | 584,90 | 105,42 | 16,34 | 92,76 | 107,23 | 14,47 | 7,00 | 174,62 | 1,53 | 70,15 |
| 2 | CRO1 | 507,60 | 106,06 | 19,54 | 89,55 | 108,59 | 19,04 | 7,00 | 175,10 | 1,94 | 65,50 |
| 2 | CRO1 | 431,40 | 98,93 | 16,46 | 82,34 | 97,42 | 15,08 | 7,00 | 135,55 | 1,29 | 68,58 |
| 2 | CRO1 | 299,60 | 86,69 | 20,34 | 73,43 | 87,89 | 14,46 | 6,00 | 107,77 | 2,50 | 64,03 |
| 2 | CRO1 | 502,60 | 110,81 | 24,84 | 81,74 | 95,47 | 13,73 | 7,00 | 202,35 | 2,90 | 59,74 |
| 2 | CRO1 | 351,80 | 91,47 | 17,00 | 71,70 | 94,24 | 22,54 | 6,00 | 107,63 | 1,00 | 69,41 |
| 2 | CRO1 | 381,70 | 87,61 | 20,08 | 77,32 | 97,99 | 20,67 | 6,00 | 145,38 | 2,62 | 61,91 |
| 2 | CRO1 | 532,10 | 105,59 | 20,15 | 84,98 | 104,39 | 19,41 | 6,00 | 157,54 | 1,27 | 70,39 |
| 2 | CRO1 | 491,60 | 107,09 | 17,96 | 82,12 | 98,09 | 15,97 | 6,00 | 141,00 | 1,15 | 71,32 |
| 2 | CRO1 | 342,90 | 92,57 | 21,52 | 75,08 | 92,12 | 17,04 | 7,00 | 138,52 | 2,21 | 59,60 |
| 2 | CRO2 | 371,80 | 95,87 | 14,95 | 78,14 | 90,05 | 11,91 | 7,00 | 110,80 | 1,42 | 70,20 |
| 2 | CRO2 | 501,10 | 105,79 | 24,86 | 84,41 | 102,40 | 17,99 | 7,00 | 214,60 | 2,93 | 57,17 |
| 2 | CRO2 | 436,40 | 98,09 | 20,54 | 81,75 | 98,94 | 17,19 | 7,00 | 173,21 | 2,52 | 60,31 |
| 2 | CRO2 | 304,90 | 90,94 | 21,36 | 70,62 | 92,03 | 21,41 | 7,00 | 124,99 | 1,66 | 59,01 |
| 2 | CRO2 | 267,50 | 82,65 | 16,90 | 68,77 | 91,06 | 22,29 | 6,00 | 95,05 | 3,24 | 64,47 |
| 2 | CRO2 | 503,40 | 105,82 | 23,53 | 86,89 | 106,69 | 19,80 | 7,00 | 209,24 | 3,25 | 58,43 |
| 2 | CRO2 | 297,17 | 86,40 | 19,08 | 75,81 | 95,14 | 19,33 | 6,00 | 123,90 | 2,73 | 58,31 |
| 2 | CRO2 | 348,80 | 96,41 | 20,46 | 73,39 | 91,25 | 17,86 | 7,00 | 142,85 | 1,64 | 59,05 |
| 2 | CRO2 | 401,70 | 93,23 | 21,36 | 80,36 | 98,81 | 18,45 | 7,00 | 166,43 | 2,36 | 58,57 |
| 2 | CRO2 | 434,10 | 101,65 | 21,11 | 76,58 | 91,11 | 14,53 | 8,00 | 122,42 | 0,81 | 71,80 |
| 2 | ADO3 | 716,60 | 113,69 | 20,21 | 93,68 | 106,63 | 12,95 | 8,00 | 185,54 | 1,38 | 74,11 |
| 2 | ADO3 | 351,39 | 91,41 | 15,65 | 63,44 | 89,72 | 26,28 | 6,00 | 113,65 | 2,55 | 67,66 |
| 2 | ADO3 | 601,40 | 106,43 | 19,47 | 94,44 | 114,97 | 20,53 | 6,00 | 204,25 | 2,58 | 66,04 |
| 2 | ADO3 | 514,20 | 98,87 | 20,47 | 78,76 | 99,30 | 20,54 | 8,00 | 157,10 | 1,83 | 69,45 |
| 2 | ADO3 | 447,97 | 95,32 | 19,64 | 81,15 | 99,12 | 17,97 | 6,00 | 146,12 | 1,98 | 67,38 |
| 2 | ADO3 | 578,74 | 106,25 | 17,39 | 87,26 | 105,71 | 18,45 | 7,00 | 169,63 | 1,26 | 70,69 |
| 2 | ADO3 | 553,40 | 97,08 | 19,72 | 80,47 | 100,96 | 20,49 | 7,00 | 131,43 | 2,30 | 76,25 |
| 2 | ADO3 | 746,70 | 116,25 | 19,83 | 99,28 | 114,14 | 14,86 | 7,00 | 213,75 | 2,49 | 71,37 |
| 2 | ADO3 | 441,16 | 98,40 | 14,93 | 80,50 | 100,01 | 19,51 | 6,00 | 121,61 | 0,82 | 72,43 |
| 2 | ADO3 | 468,67 | 97,72 | 15,83 | 88,22 | 111,78 | 23,56 | 6,00 | 133,29 | 2,66 | 71,56 |
| 2 | BO1 | 418,30 | 94,16 | 25,00 | 81,00 | 103,00 | 22,00 | 6,00 | 192,60 | 3,02 | 53,96 |
| 2 | BO1 | 296,60 | 85,82 | 18,62 | 70,95 | 86,51 | 15,56 | 6,00 | 134,40 | 3,17 | 54,69 |
| 2 | BO1 | 388,02 | 93,52 | 24,79 | 83,42 | 100,21 | 16,79 | 7,00 | 192,40 | 4,85 | 50,41 |
| 2 | BO1 | 492,30 | 97,20 | 26,39 | 87,53 | 103,16 | 15,63 | 6,00 | 290,60 | 5,96 | 40,97 |
| 2 | BO1 | 479,96 | 100,71 | 23,12 | 91,30 | 93,82 | 2,52 | 6,00 | 264,40 | 4,68 | 44,91 |
| 2 | BO1 | 469,25 | 96,17 | 24,66 | 81,02 | 94,80 | 13,78 | 6,00 | 250,50 | 6,01 | 46,62 |
| 2 | BO1 | 429,60 | 93,64 | 24,22 | 81,96 | 94,13 | 12,17 | 7,00 | 242,40 | 4,82 | 43,58 |
| 2 | BO1 | 381,20 | 87,62 | 20,61 | 81,34 | 97,07 | 15,73 | 7,00 | 199,80 | 3,64 | 47,59 |
| 2 | BO1 | 421,40 | 93,34 | 17,90 | 81,01 | 101,20 | 20,19 | 8,00 | 192,70 | 4,10 | 54,27 |
| 2 | BO1 | 190,31 | 72,61 | 19,68 | 60,55 | 82,37 | 21,82 | 7,00 | 112,50 | 6,90 | 40,89 |
| 2 | ADO2 | 469,80 | 103,25 | 19,16 | 82,55 | 105,04 | 22,49 | 7,00 | 161,29 | 2,69 | 65,67 |
| 2 | ADO2 | 466,40 | 100,35 | 18,25 | 79,25 | 93,08 | 13,83 | 6,00 | 141,80 | 2,26 | 69,60 |
| 2 | ADO2 | 549,80 | 103,92 | 20,13 | 84,88 | 105,58 | 20,70 | 7,00 | 189,32 | 3,11 | 65,57 |
| 2 | ADO2 | 339,30 | 91,26 | 20,87 | 70,15 | 85,93 | 15,78 | 6,00 | 112,60 | 2,70 | 66,81 |
| 2 | ADO2 | 358,50 | 92,82 | 20,34 | 73,32 | 91,47 | 18,15 | 6,00 | 114,11 | 3,03 | 68,17 |
| 2 | ADO2 | 353,60 | 88,19 | 19,20 | 73,89 | 92,11 | 18,22 | 7,00 | 139,35 | 2,21 | 60,59 |
| 2 | ADO2 | 279,50 | 80,48 | 19,50 | 66,91 | 84,32 | 17,41 | 6,00 | 115,47 | 2,76 | 58,69 |
| 2 | ADO2 | 337,20 | 93,01 | 22,81 | 70,12 | 85,92 | 15,80 | 7,00 | 134,86 | 2,43 | 60,01 |
| 2 | ADO2 | 232,90 | 78,78 | 16,53 | 66,42 | 88,09 | 21,67 | 6,00 | 106,90 | 2,76 | 54,10 |
| 2 | ADO2 | 369,30 | 94,64 | 16,60 | 77,23 | 97,06 | 19,83 | 6,00 | 118,75 | 2,20 | 67,84 |
| 2 | PG | 345,50 | 91,99 | 18,00 | 95,79 | 98,14 | 2,35 | 6,00 | 146,30 | 2,46 | 57,66 |
| 2 | PG | 202,34 | 72,20 | 16,00 | 62,95 | 73,71 | 10,76 | 5,00 | 92,30 | 3,83 | 54,38 |
| 2 | PG | 438,60 | 95,90 | 15,20 | 80,20 | 91,64 | 11,44 | 7,00 | 188,92 | 1,61 | 56,93 |
| 2 | PG | 362,04 | 92,67 | 19,80 | 82,10 | 82,91 | 0,81 | 7,00 | 148,95 | 3,19 | 58,86 |
| 2 | PG | 312,68 | 85,46 | 13,20 | 92,00 | 92,66 | 0,66 | 6,00 | 170,20 | 1,97 | 45,57 |
| 2 | PG | 388,10 | 93,70 | 17,90 | 76,06 | 93,90 | 17,84 | 8,00 | 198,80 | 4,08 | 48,78 |
| 2 | PG | 335,30 | 87,10 | 15,90 | 73,80 | 91,05 | 17,25 | 7,00 | 192,70 | 2,66 | 42,53 |
| 2 | PG | 300,30 | 84,28 | 18,71 | 71,39 | 82,94 | 11,55 | 6,00 | 161,63 | 3,49 | 46,18 |
| 2 | PG | 293,60 | 82,20 | 16,14 | 73,19 | 88,66 | 15,47 | 7,00 | 133,90 | 2,49 | 54,39 |
| 2 | PG | 393,20 | 94,68 | 19,80 | 77,98 | 96,08 | 18,10 | 7,00 | 187,40 | 3,24 | 52,34 |
| 3 | ME1 | 354,62 | 85,60 | 14,29 | 78,86 | 88,57 | 9,71 | 7,00 | 141,52 | 2,99 | 60,09 |
| 3 | ME1 | 305,16 | 78,86 | 10,06 | 74,37 | 97,70 | 23,33 | 5,00 | 158,81 | 3,76 | 47,96 |
| 3 | ME1 | 281,63 | 86,48 | 18,53 | 68,26 | 89,21 | 20,95 | 6,00 | 137,40 | 3,02 | 51,21 |
| 3 | ME1 | 451,38 | 99,17 | 16,81 | 80,25 | 97,42 | 17,17 | 7,00 | 199,97 | 4,54 | 55,70 |
| 3 | ME1 | 310,10 | 86,45 | 16,88 | 74,42 | 89,80 | 15,38 | 6,00 | 129,80 | 3,40 | 58,14 |
| 3 | ME1 | 311,50 | 88,47 | 19,47 | 72,53 | 92,70 | 20,17 | 8,00 | 158,10 | 3,40 | 49,25 |
| 3 | ME1 | 393,42 | 92,68 | 20,12 | 76,96 | 94,10 | 17,14 | 8,00 | 155,73 | 2,09 | 60,42 |
| 3 | ME1 | 443,94 | 99,21 | 21,55 | 84,72 | 98,73 | 14,01 | 8,00 | 113,60 | 1,45 | 74,41 |
| 3 | ME1 | 278,61 | 81,01 | 19,40 | 70,07 | 89,27 | 19,20 | 8,00 | 113,60 | 1,99 | 59,23 |
| 3 | ME1 | 206,50 | 69,80 | 18,21 | 51,00 | 81,35 | 30,35 | 8,00 | 96,80 | 1,97 | 53,12 |
| 3 | ME2 | 450,56 | 93,91 | 23,21 | 82,37 | 100,29 | 17,92 | 8,00 | 187,92 | 4,69 | 58,29 |
| 3 | ME2 | 250,42 | 78,61 | 18,78 | 69,20 | 89,46 | 20,26 | 7,00 | 107,12 | 3,93 | 57,22 |
| 3 | ME2 | 403,31 | 91,44 | 21,71 | 82,83 | 101,92 | 19,09 | 7,00 | 192,27 | 3,72 | 52,33 |
| 3 | ME2 | 327,40 | 86,97 | 19,90 | 72,59 | 91,57 | 18,98 | 7,00 | 142,46 | 3,62 | 56,49 |
| 3 | ME2 | 340,68 | 87,44 | 20,00 | 78,26 | 97,49 | 19,23 | 7,00 | 161,03 | 4,39 | 52,73 |
| 3 | ME2 | 335,33 | 86,68 | 18,36 | 79,99 | 96,84 | 16,85 | 7,00 | 166,75 | 4,15 | 50,27 |
| 3 | ME2 | 272,42 | 83,77 | 30,44 | 65,64 | 90,00 | 24,36 | 6,00 | 135,15 | 5,14 | 50,39 |
| 3 | ME2 | 298,83 | 87,74 | 20,36 | 77,67 | 91,89 | 14,22 | 7,00 | 137,14 | 3,39 | 54,11 |
| 3 | ME2 | 357,70 | 90,90 | 18,94 | 77,82 | 97,01 | 19,19 | 8,00 | 140,17 | 3,78 | 60,81 |
| 3 | ME2 | 196,62 | 74,57 | 19,33 | 66,21 | 86,54 | 20,33 | 6,00 | 94,94 | 2,46 | 51,71 |
| 3 | ME3 | 331,50 | 86,16 | 20,76 | 74,79 | 93,23 | 18,44 | 5,00 | 174,60 | 5,35 | 47,33 |
| 3 | ME3 | 497,95 | 102,97 | 20,13 | 81,27 | 93,39 | 12,12 | 6,00 | 199,18 | 3,61 | 60,00 |
| 3 | ME3 | 429,27 | 96,98 | 22,23 | 78,64 | 96,52 | 17,88 | 7,00 | 188,13 | 3,85 | 56,17 |
| 3 | ME3 | 404,78 | 90,42 | 23,49 | 79,70 | 98,04 | 18,34 | 6,00 | 201,80 | 3,71 | 50,15 |
| 3 | ME3 | 308,18 | 81,42 | 19,34 | 71,84 | 92,31 | 20,47 | 6,00 | 127,79 | 2,80 | 58,53 |
| 3 | ME3 | 438,18 | 96,36 | 20,31 | 83,64 | 101,14 | 17,50 | 6,00 | 171,06 | 3,51 | 60,96 |
| 3 | ME3 | 467,46 | 96,28 | 22,02 | 84,41 | 102,17 | 17,76 | 7,00 | 208,18 | 4,38 | 55,47 |
| 3 | ME3 | 422,52 | 94,56 | 18,86 | 79,52 | 90,18 | 10,66 | 6,00 | 156,30 | 4,15 | 63,01 |
| 3 | ME3 | 335,70 | 89,37 | 19,11 | 75,42 | 94,08 | 18,66 | 7,00 | 140,36 | 4,58 | 58,19 |
| 3 | ME3 | 445,55 | 97,85 | 23,23 | 78,00 | 97,20 | 19,20 | 7,00 | 181,42 | 3,61 | 59,28 |
| 3 | ME31 | 326,83 | 86,38 | 16,64 | 76,13 | 96,04 | 19,91 | 7,00 | 137,85 | 1,67 | 57,82 |
| 3 | ME31 | 487,79 | 99,82 | 18,81 | 84,68 | 100,38 | 15,70 | 8,00 | 212,81 | 2,75 | 56,37 |
| 3 | ME31 | 361,38 | 87,42 | 22,00 | 75,75 | 96,92 | 21,17 | 6,00 | 172,65 | 2,33 | 52,22 |
| 3 | ME31 | 287,38 | 86,62 | 18,65 | 73,87 | 82,24 | 8,37 | 8,00 | 145,19 | 2,50 | 49,48 |
| 3 | ME31 | 323,53 | 87,12 | 20,99 | 73,93 | 88,90 | 14,97 | 6,00 | 156,23 | 3,00 | 51,71 |
| 3 | ME31 | 300,85 | 86,56 | 17,93 | 76,56 | 95,47 | 18,91 | 6,00 | 139,23 | 1,33 | 53,72 |
| 3 | ME31 | 399,62 | 93,90 | 19,54 | 83,52 | 102,67 | 19,15 | 6,00 | 197,11 | 3,60 | 50,68 |
| 3 | ME31 | 326,05 | 85,56 | 16,80 | 77,62 | 96,05 | 18,43 | 6,00 | 143,79 | 1,70 | 55,90 |
| 3 | ME31 | 375,68 | 97,48 | 23,70 | 78,50 | 98,42 | 19,92 | 8,00 | 192,45 | 4,39 | 48,77 |
| 3 | ME31 | 365,97 | 93,81 | 24,35 | 75,83 | 95,02 | 19,19 | 6,00 | 182,14 | 3,65 | 50,23 |
| 3 | ME4 | 313,68 | 87,46 | 25,19 | 73,01 | 88,88 | 15,87 | 6,00 | 159,46 | 4,77 | 49,16 |
| 3 | ME4 | 124,66 | 73,59 | 22,24 | 67,63 | 85,36 | 17,73 | 6,00 | 61,09 | 3,71 | 50,99 |
| 3 | ME4 | 184,20 | 72,24 | 28,11 | 67,64 | 86,93 | 19,29 | 7,00 | 103,06 | 3,86 | 44,05 |
| 3 | ME4 | 220,53 | 78,65 | 23,39 | 64,69 | 83,54 | 18,85 | 6,00 | 114,18 | 3,92 | 48,22 |
| 3 | ME4 | 336,60 | 88,55 | 25,39 | 76,11 | 87,92 | 11,81 | 7,00 | 145,90 | 4,06 | 56,65 |
| 3 | ME4 | 308,74 | 88,22 | 18,77 | 76,55 | 85,85 | 9,30 | 7,00 | 137,85 | 2,71 | 55,35 |
| 3 | ME4 | 288,00 | 82,67 | 24,83 | 76,76 | 90,52 | 13,76 | 6,00 | 168,90 | 5,15 | 41,35 |
| 3 | ME4 | 312,68 | 82,64 | 29,19 | 74,18 | 88,16 | 13,98 | 7,00 | 150,91 | 3,39 | 51,74 |
| 3 | ME4 | 205,89 | 74,10 | 22,25 | 64,89 | 85,01 | 20,12 | 6,00 | 123,54 | 5,52 | 40,00 |
| 3 | ME4 | 271,90 | 90,89 | 19,47 | 71,79 | 84,66 | 12,87 | 6,00 | 144,31 | 3,06 | 46,93 |
| 3 | ME5 | 219,63 | 75,05 | 22,50 | 65,66 | 84,33 | 18,67 | 6,00 | 104,29 | 4,62 | 52,52 |
| 3 | ME5 | 220,66 | 80,99 | 22,56 | 66,24 | 83,52 | 17,28 | 7,00 | 117,00 | 6,54 | 46,98 |
| 3 | ME5 | 254,17 | 80,95 | 26,00 | 77,81 | 98,47 | 20,66 | 8,00 | 90,25 | 7,53 | 64,49 |
| 3 | ME5 | 302,90 | 88,11 | 24,59 | 72,10 | 93,11 | 21,01 | 7,00 | 163,90 | 5,71 | 45,89 |
| 3 | ME5 | 233,85 | 76,90 | 18,11 | 68,43 | 76,80 | 8,37 | 6,00 | 98,01 | 3,81 | 58,09 |
| 3 | ME5 | 442,73 | 99,15 | 20,61 | 83,89 | 95,94 | 12,05 | 7,00 | 188,64 | 4,78 | 57,39 |
| 3 | ME5 | 329,83 | 90,09 | 21,06 | 72,11 | 92,42 | 20,31 | 7,00 | 165,60 | 4,37 | 49,79 |
| 3 | ME5 | 465,76 | 99,86 | 28,39 | 85,31 | 103,64 | 18,33 | 7,00 | 149,94 | 7,62 | 67,81 |
| 3 | ME5 | 322,76 | 85,03 | 21,31 | 76,04 | 90,67 | 14,63 | 6,00 | 140,60 | 4,41 | 56,44 |
| 3 | ME5 | 240,60 | 80,74 | 20,22 | 68,11 | 88,64 | 20,53 | 6,00 | 127,93 | 3,75 | 46,83 |
| 3 | ME6 | 396,55 | 98,10 | 23,33 | 79,27 | 99,77 | 20,50 | 6,00 | 164,67 | 4,97 | 58,47 |
| 3 | ME6 | 363,30 | 91,10 | 22,25 | 75,65 | 94,35 | 18,70 | 7,00 | 143,13 | 2,18 | 60,60 |
| 3 | ME6 | 423,23 | 97,01 | 20,16 | 80,73 | 97,26 | 16,53 | 6,00 | 169,00 | 4,61 | 60,07 |
| 3 | ME6 | 345,93 | 89,56 | 23,21 | 75,05 | 89,03 | 13,98 | 8,00 | 143,96 | 5,72 | 58,38 |
| 3 | ME6 | 317,02 | 86,04 | 19,17 | 75,59 | 95,18 | 19,59 | 6,00 | 151,03 | 5,22 | 52,36 |
| 3 | ME6 | 301,28 | 88,15 | 22,87 | 71,51 | 86,74 | 15,23 | 7,00 | 151,26 | 3,94 | 49,79 |
| 3 | ME6 | 318,20 | 85,44 | 17,34 | 75,80 | 89,25 | 13,45 | 6,00 | 116,39 | 2,66 | 63,42 |
| 3 | ME6 | 453,50 | 100,19 | 23,08 | 84,59 | 99,75 | 15,16 | 6,00 | 174,14 | 3,05 | 61,60 |
| 3 | ME6 | 377,77 | 93,00 | 22,41 | 79,17 | 96,77 | 17,60 | 6,00 | 162,88 | 3,28 | 56,88 |
| 3 | ME6 | 461,02 | 100,00 | 20,41 | 82,91 | 104,98 | 22,07 | 7,00 | 207,74 | 3,17 | 54,94 |
| 3 | ME7 | 355,16 | 89,38 | 22,27 | 76,76 | 89,05 | 12,29 | 7,00 | 192,38 | 5,17 | 45,83 |
| 3 | ME7 | 366,94 | 90,46 | 15,79 | 78,32 | 95,90 | 17,58 | 6,00 | 178,31 | 1,88 | 51,41 |
| 3 | ME7 | 392,06 | 93,29 | 20,15 | 77,96 | 89,12 | 11,16 | 6,00 | 167,39 | 2,69 | 57,31 |
| 3 | ME7 | 370,95 | 82,87 | 15,83 | 70,07 | 86,61 | 16,54 | 7,00 | 123,50 | 2,70 | 66,71 |
| 3 | ME7 | 315,64 | 85,87 | 16,42 | 78,86 | 89,30 | 10,44 | 7,00 | 141,44 | 2,42 | 55,19 |
| 3 | ME7 | 301,74 | 85,89 | 15,65 | 76,79 | 92,11 | 15,32 | 6,00 | 145,70 | 2,13 | 51,71 |
| 3 | ME7 | 357,07 | 78,34 | 14,81 | 69,46 | 77,84 | 8,38 | 7,00 | 112,28 | 1,89 | 68,56 |
| 3 | ME7 | 346,04 | 91,99 | 17,56 | 77,87 | 95,01 | 17,14 | 7,00 | 177,85 | 3,34 | 48,60 |
| 3 | ME7 | 276,62 | 81,53 | 23,58 | 73,92 | 89,54 | 15,62 | 7,00 | 158,12 | 4,13 | 42,84 |
| 3 | ME7 | 439,88 | 96,80 | 23,75 | 83,96 | 99,50 | 15,54 | 7,00 | 201,60 | 2,96 | 54,17 |
| 3 | ME8 | 334,74 | 84,13 | 19,78 | 80,21 | 94,32 | 14,11 | 7,00 | 152,48 | 4,27 | 54,45 |
| 3 | ME8 | 304,41 | 77,03 | 20,94 | 69,95 | 86,14 | 16,19 | 7,00 | 139,94 | 4,13 | 54,03 |
| 3 | ME8 | 346,92 | 88,13 | 20,68 | 76,48 | 91,47 | 14,99 | 6,00 | 163,40 | 3,38 | 52,90 |
| 3 | ME8 | 321,21 | 89,26 | 17,02 | 71,93 | 92,30 | 20,37 | 7,00 | 170,45 | 4,27 | 46,94 |
| 3 | ME8 | 331,65 | 84,16 | 19,38 | 75,89 | 94,92 | 19,03 | 7,00 | 140,51 | 3,15 | 57,63 |
| 3 | ME8 | 427,60 | 89,78 | 17,43 | 82,53 | 97,77 | 15,24 | 5,00 | 184,73 | 4,29 | 56,80 |
| 3 | ME8 | 408,45 | 97,37 | 18,64 | 83,76 | 100,54 | 16,78 | 6,00 | 203,50 | 6,04 | 50,18 |
| 3 | ME8 | 441,87 | 98,98 | 17,77 | 84,17 | 95,61 | 11,44 | 7,00 | 219,48 | 3,72 | 50,33 |
| 3 | ME8 | 330,56 | 88,06 | 20,05 | 75,13 | 92,53 | 17,40 | 7,00 | 183,33 | 4,12 | 44,54 |
| 3 | ME8 | 363,90 | 93,98 | 23,95 | 74,98 | 93,31 | 18,33 | 7,00 | 193,56 | 5,84 | 46,81 |
| 3 | ME9 | 613,00 | 109,37 | 23,48 | 85,09 | 102,13 | 17,04 | 6,00 | 195,80 | 3,90 | 68,06 |
| 3 | ME9 | 369,60 | 92,88 | 22,31 | 77,33 | 94,37 | 17,04 | 7,00 | 172,03 | 4,45 | 53,46 |
| 3 | ME9 | 249,45 | 87,04 | 17,90 | 79,38 | 95,57 | 16,19 | 6,00 | 152,34 | 3,72 | 38,93 |
| 3 | ME9 | 423,00 | 95,29 | 21,60 | 82,50 | 99,37 | 16,87 | 7,00 | 173,68 | 3,26 | 58,94 |
| 3 | ME9 | 341,74 | 88,77 | 29,84 | 75,36 | 92,44 | 17,08 | 6,00 | 171,68 | 4,24 | 49,76 |
| 3 | ME9 | 360,69 | 88,07 | 23,75 | 70,18 | 91,57 | 21,39 | 6,00 | 153,85 | 5,01 | 57,35 |
| 3 | ME9 | 266,55 | 82,77 | 26,09 | 67,47 | 85,33 | 17,86 | 8,00 | 128,75 | 4,78 | 51,70 |
| 3 | ME9 | 355,76 | 89,79 | 19,87 | 76,93 | 87,97 | 11,04 | 7,00 | 156,10 | 3,99 | 56,12 |
| 3 | ME9 | 423,99 | 95,29 | 24,94 | 85,13 | 96,89 | 11,76 | 6,00 | 179,56 | 4,07 | 57,65 |
| 3 | ME9 | 279,57 | 84,74 | 28,27 | 70,00 | 86,33 | 16,33 | 6,00 | 133,30 | 5,22 | 52,32 |
| 3 | ME10 | 298,07 | 88,24 | 15,72 | 71,73 | 86,44 | 14,71 | 7,00 | 120,70 | 3,18 | 59,51 |
| 3 | ME10 | 375,10 | 90,72 | 14,31 | 88,44 | 98,05 | 9,61 | 6,00 | 167,66 | 3,48 | 55,30 |
| 3 | ME10 | 456,06 | 98,97 | 23,51 | 89,04 | 100,84 | 11,80 | 7,00 | 151,30 | 4,66 | 66,82 |
| 3 | ME10 | 413,45 | 104,91 | 24,98 | 87,49 | 103,50 | 16,01 | 7,00 | 157,43 | 4,57 | 61,92 |
| 3 | ME10 | 410,26 | 92,22 | 15,49 | 81,39 | 100,58 | 19,19 | 7,00 | 187,93 | 3,46 | 54,19 |
| 3 | ME10 | 355,12 | 90,55 | 17,12 | 78,87 | 93,65 | 14,78 | 7,00 | 160,18 | 3,72 | 54,89 |
| 3 | ME10 | 465,50 | 98,22 | 17,58 | 98,72 | 100,02 | 1,30 | 7,00 | 175,95 | 5,79 | 62,20 |
| 3 | ME10 | 315,72 | 85,66 | 24,63 | 70,78 | 92,50 | 21,72 | 7,00 | 164,49 | 4,21 | 47,90 |
| 3 | ME10 | 390,72 | 93,11 | 22,27 | 81,83 | 99,66 | 17,83 | 7,00 | 165,52 | 2,71 | 57,64 |
| 3 | ME10 | 399,80 | 91,19 | 22,57 | 84,90 | 104,06 | 19,16 | 6,00 | 186,86 | 2,63 | 53,26 |
| 3 | ME11 | 275,46 | 82,20 | 18,33 | 73,40 | 87,47 | 14,07 | 8,00 | 113,10 | 4,01 | 58,94 |
| 3 | ME11 | 207,57 | 76,27 | 24,16 | 63,42 | 79,11 | 15,69 | 7,00 | 113,60 | 3,69 | 45,27 |
| 3 | ME11 | 279,50 | 87,14 | 21,74 | 72,52 | 88,55 | 16,03 | 9,00 | 133,20 | 4,03 | 52,34 |
| 3 | ME11 | 308,20 | 87,51 | 19,61 | 76,47 | 84,82 | 8,35 | 7,00 | 153,02 | 4,47 | 50,35 |
| 3 | ME11 | 410,09 | 94,40 | 21,44 | 87,95 | 97,72 | 9,77 | 7,00 | 180,82 | 5,49 | 55,91 |
| 3 | ME11 | 325,64 | 87,10 | 19,46 | 78,32 | 92,92 | 14,60 | 6,00 | 149,52 | 4,10 | 54,08 |
| 3 | ME11 | 250,67 | 80,44 | 23,31 | 67,51 | 89,34 | 21,83 | 6,00 | 142,72 | 4,56 | 43,06 |
| 3 | ME11 | 315,94 | 88,09 | 19,78 | 75,99 | 89,18 | 13,19 | 7,00 | 154,90 | 3,70 | 50,97 |
| 3 | ME11 | 314,26 | 88,09 | 20,72 | 71,50 | 91,30 | 19,80 | 7,00 | 138,33 | 2,50 | 55,98 |
| 3 | ME11 | 342,29 | 88,44 | 21,06 | 75,29 | 87,11 | 11,82 | 8,00 | 164,31 | 3,72 | 52,00 |
| 3 | ME12 | 470,64 | 98,94 | 25,31 | 76,74 | 99,06 | 22,32 | 8,00 | 209,76 | 3,46 | 55,43 |
| 3 | ME12 | 447,23 | 92,52 | 20,65 | 79,75 | 95,99 | 16,24 | 7,00 | 173,75 | 1,88 | 61,15 |
| 3 | ME12 | 217,06 | 77,96 | 20,87 | 61,04 | 82,81 | 21,77 | 7,00 | 106,10 | 2,93 | 51,12 |
| 3 | ME12 | 233,09 | 73,16 | 25,80 | 64,62 | 73,43 | 8,81 | 8,00 | 123,88 | 4,00 | 46,85 |
| 3 | ME12 | 284,89 | 80,44 | 20,16 | 71,07 | 81,59 | 10,52 | 6,00 | 119,62 | 2,92 | 58,01 |
| 3 | ME12 | 430,35 | 95,88 | 19,86 | 78,77 | 98,73 | 19,96 | 7,00 | 171,08 | 3,03 | 60,25 |
| 3 | ME12 | 456,05 | 96,88 | 21,69 | 82,27 | 100,92 | 18,65 | 7,00 | 199,60 | 2,64 | 56,23 |
| 3 | ME12 | 349,19 | 90,04 | 19,54 | 76,25 | 92,40 | 16,15 | 8,00 | 143,05 | 1,56 | 59,03 |
| 3 | ME12 | 257,72 | 86,30 | 19,50 | 75,18 | 92,05 | 16,87 | 5,00 | 117,51 | 1,78 | 54,40 |
| 3 | ME12 | 428,55 | 93,76 | 22,11 | 82,25 | 99,52 | 17,27 | 7,00 | 161,30 | 2,35 | 62,36 |
| 3 | ME13 | 255,31 | 75,03 | 22,40 | 52,62 | 77,06 | 24,44 | 6,00 | 102,27 | 3,60 | 59,94 |
| 3 | ME13 | 443,55 | 84,16 | 17,00 | 86,13 | 97,44 | 11,31 | 7,00 | 235,84 | 6,41 | 46,83 |
| 3 | ME13 | 558,72 | 105,77 | 27,73 | 83,65 | 98,41 | 14,76 | 7,00 | 286,84 | 6,06 | 48,66 |
| 3 | ME13 | 431,33 | 93,30 | 21,04 | 82,70 | 95,88 | 13,18 | 7,00 | 191,20 | 6,59 | 55,67 |
| 3 | ME13 | 349,94 | 90,57 | 21,93 | 73,20 | 89,56 | 16,36 | 6,00 | 153,53 | 4,31 | 56,13 |
| 3 | ME13 | 444,24 | 90,83 | 19,50 | 78,39 | 93,91 | 15,52 | 6,00 | 189,90 | 4,84 | 57,25 |
| 3 | ME13 | 366,64 | 88,75 | 19,50 | 72,87 | 87,81 | 14,94 | 6,00 | 165,44 | 3,35 | 54,88 |
| 3 | ME13 | 457,42 | 96,80 | 21,35 | 77,33 | 91,52 | 14,19 | 5,00 | 182,13 | 4,83 | 60,18 |
| 3 | ME13 | 322,22 | 84,82 | 20,09 | 64,55 | 77,59 | 13,04 | 8,00 | 165,82 | 5,94 | 48,54 |
| 3 | ME13 | 284,16 | 80,23 | 22,79 | 66,59 | 85,24 | 18,65 | 6,00 | 145,39 | 5,54 | 48,84 |
| 3 | ME14 | 280,30 | 89,93 | 25,24 | 67,71 | 88,15 | 20,44 | 7,00 | 147,50 | 4,41 | 47,38 |
| 3 | ME14 | 254,39 | 85,53 | 23,02 | 67,75 | 84,36 | 16,61 | 6,00 | 131,29 | 3,50 | 48,39 |
| 3 | ME14 | 383,44 | 94,08 | 20,18 | 77,77 | 95,13 | 17,36 | 7,00 | 192,28 | 5,70 | 49,85 |
| 3 | ME14 | 324,25 | 86,05 | 18,34 | 73,87 | 88,26 | 14,39 | 6,00 | 161,36 | 3,81 | 50,24 |
| 3 | ME14 | 293,03 | 85,62 | 22,08 | 72,35 | 89,61 | 17,26 | 6,00 | 153,50 | 6,19 | 47,62 |
| 3 | ME14 | 412,97 | 99,07 | 29,35 | 76,68 | 93,44 | 16,76 | 6,00 | 244,65 | 5,57 | 40,76 |
| 3 | ME14 | 502,41 | 100,00 | 21,70 | 89,88 | 106,21 | 16,33 | 7,00 | 241,68 | 4,64 | 51,90 |
| 3 | ME14 | 424,47 | 98,91 | 17,78 | 81,32 | 93,94 | 12,62 | 7,00 | 190,55 | 3,71 | 55,11 |
| 3 | ME14 | 257,03 | 79,49 | 17,73 | 65,45 | 84,37 | 18,92 | 7,00 | 123,27 | 4,17 | 52,04 |
| 3 | ME14 | 399,00 | 98,80 | 17,03 | 77,08 | 90,91 | 13,83 | 8,00 | 159,16 | 3,40 | 60,11 |
| 3 | ME16 | 292,38 | 82,80 | 16,19 | 72,03 | 84,21 | 12,18 | 7,00 | 151,83 | 5,08 | 48,07 |
| 3 | ME16 | 381,47 | 89,83 | 18,63 | 82,00 | 95,22 | 13,22 | 7,00 | 191,56 | 4,97 | 49,78 |
| 3 | ME16 | 480,50 | 94,20 | 25,39 | 87,90 | 96,87 | 8,97 | 7,00 | 260,43 | 6,72 | 45,80 |
| 3 | ME16 | 450,66 | 96,20 | 19,31 | 85,37 | 96,09 | 10,72 | 7,00 | 171,40 | 5,33 | 61,97 |
| 3 | ME16 | 272,23 | 84,62 | 21,65 | 68,72 | 80,87 | 12,15 | 6,00 | 137,32 | 3,92 | 49,56 |
| 3 | ME16 | 335,09 | 88,31 | 18,91 | 76,70 | 89,43 | 12,73 | 6,00 | 178,20 | 5,20 | 46,82 |
| 3 | ME16 | 301,52 | 83,47 | 23,86 | 65,67 | 87,60 | 21,93 | 7,00 | 151,70 | 5,88 | 49,69 |
| 3 | ME16 | 217,51 | 72,95 | 16,54 | 65,49 | 78,27 | 12,78 | 6,00 | 106,80 | 4,82 | 50,90 |
| 3 | ME16 | 298,30 | 83,48 | 20,02 | 75,00 | 81,74 | 6,74 | 6,00 | 133,20 | 4,43 | 55,35 |
| 3 | ME16 | 329,37 | 86,98 | 19,96 | 76,11 | 89,36 | 13,25 | 7,00 | 151,22 | 3,87 | 54,09 |
| 3 | ME17 | 376,07 | 92,42 | 21,64 | 79,15 | 94,91 | 15,76 | 6,00 | 191,17 | 3,54 | 49,17 |
| 3 | ME17 | 408,32 | 94,93 | 23,62 | 73,99 | 90,91 | 16,92 | 8,00 | 236,71 | 4,82 | 42,03 |
| 3 | ME17 | 264,68 | 77,18 | 21,39 | 71,55 | 82,24 | 10,69 | 7,00 | 162,55 | 4,76 | 38,59 |
| 3 | ME17 | 497,00 | 97,15 | 30,32 | 75,82 | 90,55 | 14,73 | 7,00 | 210,75 | 4,69 | 57,60 |
| 3 | ME17 | 359,10 | 91,59 | 24,73 | 76,81 | 89,23 | 12,42 | 6,00 | 206,87 | 4,70 | 42,39 |
| 3 | ME17 | 373,30 | 95,10 | 31,53 | 75,85 | 90,21 | 14,36 | 6,00 | 181,76 | 5,69 | 51,31 |
| 3 | ME17 | 358,89 | 90,69 | 20,55 | 77,36 | 97,23 | 19,87 | 6,00 | 169,79 | 3,80 | 52,69 |
| 3 | ME17 | 436,07 | 94,14 | 26,45 | 84,96 | 96,42 | 11,46 | 6,00 | 220,21 | 4,12 | 49,50 |
| 3 | ME17 | 275,66 | 84,51 | 19,70 | 71,90 | 88,57 | 16,67 | 6,00 | 132,80 | 4,37 | 51,82 |
| 3 | ME17 | 270,99 | 93,17 | 23,21 | 70,23 | 82,81 | 12,58 | 6,00 | 136,82 | 3,49 | 49,51 |
| 3 | ME18 | 350,77 | 84,92 | 18,90 | 76,70 | 95,97 | 19,27 | 6,00 | 162,91 | 3,27 | 53,56 |
| 3 | ME18 | 499,43 | 101,94 | 22,61 | 87,71 | 103,11 | 15,40 | 6,00 | 240,93 | 4,48 | 51,76 |
| 3 | ME18 | 288,42 | 84,27 | 15,94 | 73,45 | 94,91 | 21,46 | 6,00 | 118,44 | 3,63 | 58,93 |
| 3 | ME18 | 468,81 | 96,00 | 15,04 | 85,17 | 111,59 | 26,42 | 7,00 | 210,96 | 3,51 | 55,00 |
| 3 | ME18 | 458,27 | 96,92 | 25,53 | 83,64 | 99,85 | 16,21 | 6,00 | 211,06 | 4,27 | 53,94 |
| 3 | ME18 | 351,99 | 89,48 | 22,96 | 79,42 | 95,44 | 16,02 | 6,00 | 174,86 | 3,20 | 50,32 |
| 3 | ME18 | 405,64 | 92,75 | 19,37 | 82,84 | 99,73 | 16,89 | 6,00 | 188,56 | 6,25 | 53,52 |
| 3 | ME18 | 449,40 | 93,02 | 24,02 | 86,54 | 103,59 | 17,05 | 6,00 | 199,04 | 4,50 | 55,71 |
| 3 | ME18 | 368,70 | 87,34 | 22,82 | 80,93 | 96,45 | 15,52 | 6,00 | 191,18 | 5,64 | 48,15 |
| 3 | ME18 | 347,88 | 87,36 | 21,60 | 77,80 | 95,36 | 17,56 | 7,00 | 189,91 | 3,86 | 45,41 |
| 3 | ME19 | 310,30 | 89,53 | 22,92 | 74,67 | 83,71 | 9,04 | 8,00 | 136,62 | 3,61 | 55,97 |
| 3 | ME19 | 286,10 | 89,51 | 15,25 | 73,18 | 87,74 | 14,56 | 7,00 | 102,55 | 5,50 | 64,16 |
| 3 | ME19 | 560,30 | 108,31 | 25,71 | 87,62 | 102,96 | 15,34 | 7,00 | 289,04 | 4,58 | 48,41 |
| 3 | ME19 | 427,69 | 96,84 | 26,08 | 86,97 | 100,04 | 13,07 | 6,00 | 185,35 | 3,60 | 56,66 |
| 3 | ME19 | 312,13 | 88,04 | 16,47 | 77,32 | 87,48 | 10,16 | 7,00 | 131,92 | 3,58 | 57,74 |
| 3 | ME19 | 299,65 | 88,89 | 21,03 | 71,26 | 90,26 | 19,00 | 5,00 | 125,76 | 3,38 | 58,03 |
| 3 | ME19 | 339,31 | 88,73 | 23,74 | 79,44 | 92,23 | 12,79 | 7,00 | 169,29 | 4,83 | 50,11 |
| 3 | ME19 | 266,24 | 81,55 | 19,25 | 69,78 | 86,96 | 17,18 | 7,00 | 115,86 | 4,19 | 56,48 |
| 3 | ME19 | 297,36 | 94,29 | 26,67 | 79,63 | 97,52 | 17,89 | 8,00 | 121,04 | 5,98 | 59,30 |
| 3 | ME19 | 371,62 | 95,84 | 22,49 | 78,37 | 93,81 | 15,44 | 8,00 | 166,04 | 5,55 | 55,32 |
| 3 | ME20 | 387,53 | 90,22 | 17,66 | 75,90 | 96,03 | 20,13 | 7,00 | 173,37 | 2,85 | 55,26 |
| 3 | ME20 | 430,23 | 97,83 | 17,01 | 79,53 | 93,72 | 14,19 | 7,00 | 162,68 | 3,47 | 62,19 |
| 3 | ME20 | 335,21 | 86,52 | 20,70 | 78,60 | 87,99 | 9,39 | 6,00 | 128,69 | 1,66 | 61,61 |
| 3 | ME20 | 272,88 | 76,83 | 18,23 | 68,10 | 87,20 | 19,10 | 7,00 | 116,93 | 2,98 | 57,15 |
| 3 | ME20 | 243,87 | 76,10 | 18,22 | 68,96 | 87,10 | 18,14 | 7,00 | 101,46 | 2,14 | 58,40 |
| 3 | ME20 | 169,59 | 68,18 | 16,84 | 58,13 | 77,72 | 19,59 | 7,00 | 94,28 | 1,80 | 44,41 |
| 3 | ME20 | 191,14 | 69,69 | 19,70 | 62,00 | 79,71 | 17,71 | 7,00 | 80,59 | 1,64 | 57,84 |
| 3 | ME20 | 347,07 | 88,56 | 19,74 | 74,88 | 92,69 | 17,81 | 6,00 | 146,22 | 1,28 | 57,87 |
| 3 | ME20 | 364,38 | 85,91 | 25,16 | 76,33 | 89,95 | 13,62 | 7,00 | 178,13 | 2,85 | 51,11 |
| 3 | ME20 | 276,21 | 80,88 | 21,11 | 67,81 | 82,48 | 14,67 | 6,00 | 122,38 | 2,11 | 55,69 |
| 3 | ME21 | 438,38 | 97,00 | 24,47 | 81,06 | 98,86 | 17,80 | 7,00 | 219,87 | 3,01 | 49,84 |
| 3 | ME21 | 298,16 | 86,92 | 21,75 | 62,98 | 80,86 | 17,88 | 6,00 | 133,54 | 2,29 | 55,21 |
| 3 | ME21 | 217,67 | 73,49 | 21,56 | 61,25 | 82,00 | 20,75 | 6,00 | 110,81 | 4,45 | 49,09 |
| 3 | ME21 | 370,28 | 91,48 | 24,90 | 73,67 | 91,96 | 18,29 | 7,00 | 184,17 | 3,22 | 50,26 |
| 3 | ME21 | 385,43 | 90,44 | 20,47 | 78,50 | 94,08 | 15,58 | 7,00 | 177,26 | 3,19 | 54,01 |
| 3 | ME21 | 362,47 | 92,70 | 22,73 | 79,78 | 91,69 | 11,91 | 7,00 | 177,78 | 2,61 | 50,95 |
| 3 | ME21 | 278,32 | 84,00 | 21,03 | 71,40 | 84,52 | 13,12 | 7,00 | 144,82 | 3,38 | 47,97 |
| 3 | ME21 | 404,62 | 89,89 | 21,02 | 82,52 | 97,92 | 15,40 | 7,00 | 183,71 | 2,55 | 54,60 |
| 3 | ME21 | 200,87 | 69,87 | 16,87 | 63,97 | 80,32 | 16,35 | 6,00 | 95,86 | 1,99 | 52,28 |
| 3 | ME21 | 328,96 | 85,64 | 26,64 | 71,82 | 86,89 | 15,07 | 8,00 | 172,25 | 2,77 | 47,64 |
| 3 | MA1 | 439,92 | 96,68 | 19,21 | 84,93 | 102,93 | 18,00 | 6,00 | 186,60 | 4,13 | 57,58 |
| 3 | MA1 | 525,05 | 106,09 | 25,43 | 82,45 | 104,65 | 22,20 | 8,00 | 248,24 | 5,33 | 52,72 |
| 3 | MA1 | 504,42 | 101,50 | 21,75 | 85,23 | 103,90 | 18,67 | 8,00 | 209,91 | 5,05 | 58,39 |
| 3 | MA1 | 574,02 | 103,49 | 19,25 | 90,01 | 107,19 | 17,18 | 5,00 | 259,86 | 5,34 | 54,73 |
| 3 | MA1 | 553,10 | 105,53 | 19,25 | 84,86 | 99,62 | 14,76 | 7,00 | 229,89 | 3,83 | 58,44 |
| 3 | MA1 | 369,45 | 93,08 | 19,12 | 75,21 | 96,69 | 21,48 | 7,00 | 138,99 | 4,90 | 62,38 |
| 3 | MA1 | 392,92 | 91,71 | 18,73 | 76,39 | 89,15 | 12,76 | 6,00 | 162,22 | 3,56 | 58,71 |
| 3 | MA1 | 416,34 | 98,15 | 21,45 | 75,66 | 95,43 | 19,77 | 7,00 | 203,32 | 6,09 | 51,16 |
| 3 | MA1 | 346,22 | 94,63 | 18,57 | 79,35 | 96,23 | 16,88 | 6,00 | 163,71 | 3,65 | 52,72 |
| 3 | MA1 | 337,01 | 85,42 | 26,71 | 74,25 | 94,88 | 20,63 | 7,00 | 173,30 | 5,48 | 48,58 |
| 3 | MA2 | 490,66 | 99,21 | 19,88 | 83,56 | 105,60 | 22,04 | 7,00 | 226,30 | 3,80 | 53,88 |
| 3 | MA2 | 566,38 | 103,56 | 14,84 | 88,69 | 97,96 | 9,27 | 7,00 | 246,17 | 4,24 | 56,54 |
| 3 | MA2 | 336,70 | 91,10 | 22,40 | 71,63 | 87,92 | 16,29 | 7,00 | 156,38 | 4,42 | 53,56 |
| 3 | MA2 | 431,80 | 97,50 | 23,94 | 79,49 | 90,44 | 10,95 | 8,00 | 196,96 | 3,40 | 54,39 |
| 3 | MA2 | 440,65 | 95,83 | 19,82 | 82,45 | 96,86 | 14,41 | 7,00 | 175,70 | 1,84 | 60,13 |
| 3 | MA2 | 308,90 | 87,84 | 25,31 | 71,44 | 89,00 | 17,56 | 7,00 | 134,74 | 1,75 | 56,38 |
| 3 | MA2 | 516,13 | 99,19 | 22,03 | 87,83 | 97,02 | 9,19 | 8,00 | 226,58 | 2,70 | 56,10 |
| 3 | MA2 | 262,78 | 90,16 | 21,23 | 75,87 | 92,23 | 16,36 | 8,00 | 157,68 | 4,25 | 40,00 |
| 3 | MA2 | 560,20 | 103,30 | 19,25 | 88,09 | 106,50 | 18,41 | 8,00 | 227,14 | 2,91 | 59,45 |
| 3 | MA2 | 424,24 | 95,64 | 26,13 | 83,04 | 101,00 | 17,96 | 8,00 | 227,92 | 4,54 | 46,28 |
| 3 | MA3 | 490,57 | 98,79 | 30,09 | 84,70 | 97,90 | 13,20 | 7,00 | 240,30 | 4,73 | 51,02 |
| 3 | MA3 | 403,86 | 83,57 | 21,12 | 80,07 | 92,64 | 12,57 | 6,00 | 203,66 | 3,38 | 49,57 |
| 3 | MA3 | 343,99 | 90,57 | 20,21 | 77,53 | 87,49 | 9,96 | 7,00 | 187,70 | 3,60 | 45,43 |
| 3 | MA3 | 395,40 | 94,87 | 27,55 | 75,34 | 89,94 | 14,60 | 7,00 | 196,88 | 5,23 | 50,21 |
| 3 | MA3 | 382,20 | 91,79 | 18,13 | 75,99 | 88,97 | 12,98 | 5,00 | 152,30 | 4,38 | 60,15 |
| 3 | MA3 | 371,26 | 94,15 | 27,01 | 79,55 | 92,99 | 13,44 | 7,00 | 217,03 | 5,56 | 41,54 |
| 3 | MA3 | 337,67 | 89,54 | 24,73 | 72,66 | 90,37 | 17,71 | 6,00 | 185,00 | 3,36 | 45,21 |
| 3 | MA3 | 304,17 | 83,14 | 24,18 | 78,49 | 95,59 | 17,10 | 6,00 | 145,69 | 4,45 | 52,10 |
| 3 | MA3 | 421,19 | 95,30 | 28,67 | 77,09 | 99,61 | 22,52 | 6,00 | 199,80 | 4,07 | 52,56 |
| 3 | MA3 | 334,28 | 90,68 | 20,02 | 73,75 | 85,97 | 12,22 | 6,00 | 153,27 | 2,90 | 54,15 |
| 3 | MA4 | 411,70 | 95,73 | 17,96 | 80,76 | 98,01 | 17,25 | 8,00 | 193,02 | 4,05 | 53,12 |
| 3 | MA4 | 438,19 | 94,17 | 16,54 | 81,40 | 101,23 | 19,83 | 8,00 | 167,86 | 3,50 | 61,69 |
| 3 | MA4 | 581,63 | 99,72 | 17,02 | 87,84 | 103,39 | 15,55 | 8,00 | 239,43 | 4,07 | 58,83 |
| 3 | MA4 | 408,37 | 89,91 | 19,92 | 81,37 | 103,76 | 22,39 | 6,00 | 185,88 | 2,71 | 54,48 |
| 3 | MA4 | 378,46 | 90,32 | 17,87 | 81,44 | 98,13 | 16,69 | 6,00 | 169,76 | 3,90 | 55,14 |
| 3 | MA4 | 365,60 | 92,96 | 18,41 | 77,77 | 94,50 | 16,73 | 7,00 | 151,01 | 3,49 | 58,70 |
| 3 | MA4 | 411,29 | 93,14 | 15,30 | 79,44 | 94,25 | 14,81 | 7,00 | 197,18 | 4,52 | 52,06 |
| 3 | MA4 | 459,19 | 96,50 | 21,41 | 88,47 | 101,55 | 13,08 | 6,00 | 211,60 | 4,60 | 53,92 |
| 3 | MA4 | 413,98 | 92,23 | 18,99 | 81,24 | 100,22 | 18,98 | 7,00 | 176,74 | 4,16 | 57,31 |
| 3 | MA4 | 222,63 | 79,14 | 17,05 | 69,55 | 79,83 | 10,28 | 6,00 | 99,88 | 5,26 | 55,14 |
| 3 | MA5 | 328,47 | 86,33 | 24,44 | 75,81 | 94,50 | 18,69 | 6,00 | 142,33 | 3,35 | 56,67 |
| 3 | MA5 | 438,80 | 99,08 | 19,79 | 83,73 | 98,72 | 14,99 | 8,00 | 160,16 | 4,08 | 63,50 |
| 3 | MA5 | 384,70 | 93,77 | 21,79 | 78,14 | 96,78 | 18,64 | 7,00 | 154,83 | 4,05 | 59,75 |
| 3 | MA5 | 292,50 | 85,35 | 23,27 | 69,19 | 84,53 | 15,34 | 7,00 | 119,17 | 3,26 | 59,26 |
| 3 | MA5 | 352,08 | 86,92 | 23,23 | 79,80 | 94,97 | 15,17 | 6,00 | 143,72 | 2,79 | 59,18 |
| 3 | MA5 | 303,90 | 83,45 | 21,42 | 72,68 | 88,82 | 16,14 | 7,00 | 136,87 | 3,53 | 54,96 |
| 3 | MA5 | 312,97 | 88,16 | 22,42 | 71,44 | 91,68 | 20,24 | 7,00 | 128,78 | 4,64 | 58,85 |
| 3 | MA5 | 472,43 | 100,72 | 23,06 | 82,40 | 96,33 | 13,93 | 7,00 | 229,07 | 4,50 | 51,51 |
| 3 | MA5 | 348,02 | 91,29 | 17,55 | 74,36 | 90,04 | 15,68 | 7,00 | 145,18 | 4,73 | 58,28 |
| 3 | MA5 | 331,24 | 89,97 | 27,24 | 76,31 | 87,27 | 10,96 | 7,00 | 145,90 | 4,65 | 55,95 |
| 3 | BA1 | 439,16 | 95,19 | 21,53 | 74,45 | 98,33 | 23,88 | 7,00 | 202,39 | 4,03 | 53,91 |
| 3 | BA1 | 398,59 | 92,31 | 18,44 | 78,91 | 91,29 | 12,38 | 7,00 | 185,80 | 3,75 | 53,39 |
| 3 | BA1 | 562,96 | 102,82 | 21,10 | 81,77 | 115,00 | 33,23 | 6,00 | 240,40 | 4,22 | 57,30 |
| 3 | BA1 | 662,30 | 112,24 | 22,39 | 98,21 | 110,13 | 11,92 | 6,00 | 293,84 | 3,98 | 55,63 |
| 3 | BA1 | 548,62 | 100,13 | 26,83 | 85,01 | 103,18 | 18,17 | 7,00 | 244,86 | 4,42 | 55,37 |
| 3 | BA1 | 478,91 | 97,90 | 21,13 | 86,93 | 103,66 | 16,73 | 7,00 | 212,22 | 2,90 | 55,69 |
| 3 | BA1 | 358,65 | 86,91 | 19,82 | 75,19 | 93,71 | 18,52 | 6,00 | 152,99 | 4,24 | 57,34 |
| 3 | BA1 | 470,08 | 96,18 | 22,81 | 75,14 | 100,25 | 25,11 | 7,00 | 194,53 | 4,61 | 58,62 |
| 3 | BA1 | 468,83 | 97,10 | 23,05 | 84,39 | 99,72 | 15,33 | 5,00 | 241,64 | 3,47 | 48,46 |
| 3 | BA1 | 360,35 | 87,74 | 21,87 | 74,65 | 95,57 | 20,92 | 7,00 | 166,94 | 4,21 | 53,67 |
| 3 | VA1 | 350,11 | 93,39 | 19,09 | 78,00 | 92,45 | 14,45 | 7,00 | 203,58 | 5,52 | 41,85 |
| 3 | VA1 | 310,55 | 85,36 | 19,79 | 79,15 | 91,91 | 12,76 | 6,00 | 158,43 | 3,24 | 48,98 |
| 3 | VA1 | 419,84 | 92,89 | 21,71 | 84,69 | 95,93 | 11,24 | 7,00 | 217,16 | 2,42 | 48,28 |
| 3 | VA1 | 319,46 | 84,34 | 17,55 | 78,91 | 93,90 | 14,99 | 6,00 | 169,75 | 3,07 | 46,86 |
| 3 | VA1 | 304,65 | 84,87 | 22,69 | 73,38 | 90,16 | 16,78 | 7,00 | 135,55 | 2,94 | 55,51 |
| 3 | VA1 | 501,16 | 92,87 | 25,82 | 89,75 | 102,52 | 12,77 | 8,00 | 241,64 | 2,26 | 51,78 |
| 3 | VA1 | 372,24 | 89,64 | 27,66 | 75,99 | 95,77 | 19,78 | 6,00 | 201,80 | 4,76 | 45,79 |
| 3 | VA1 | 382,17 | 91,51 | 25,55 | 81,21 | 95,98 | 14,77 | 7,00 | 213,19 | 4,34 | 44,22 |
| 3 | VA1 | 285,19 | 85,97 | 22,97 | 71,88 | 81,87 | 9,99 | 6,00 | 143,29 | 3,05 | 49,76 |
| 3 | VA1 | 294,20 | 87,95 | 22,17 | 74,35 | 92,17 | 17,82 | 6,00 | 148,72 | 2,93 | 49,45 |
| 3 | MC1 | 236,00 | 75,87 | 17,79 | 70,47 | 89,97 | 19,50 | 6,00 | 114,60 | 5,01 | 51,44 |
| 3 | MC1 | 266,49 | 80,58 | 20,22 | 70,00 | 87,34 | 17,34 | 6,00 | 131,59 | 6,24 | 50,62 |
| 3 | MC1 | 272,49 | 81,05 | 13,33 | 65,50 | 78,17 | 12,67 | 6,00 | 133,91 | 4,37 | 50,86 |
| 3 | MO2 | 563,62 | 103,89 | 18,64 | 85,67 | 102,47 | 16,80 | 9,00 | 239,71 | 2,73 | 57,47 |
| 3 | MO2 | 423,01 | 94,63 | 20,40 | 77,41 | 95,24 | 17,83 | 7,00 | 167,82 | 2,48 | 60,33 |
| 3 | MO2 | 213,05 | 71,71 | 16,56 | 67,78 | 80,79 | 13,01 | 7,00 | 111,35 | 1,01 | 47,74 |
| 3 | MO2 | 360,62 | 92,29 | 18,17 | 78,55 | 87,97 | 9,42 | 6,00 | 185,00 | 1,71 | 48,70 |
| 3 | MO2 | 342,13 | 88,83 | 19,27 | 73,96 | 91,30 | 17,34 | 5,00 | 160,97 | 2,67 | 52,95 |
| 3 | MO2 | 299,63 | 83,15 | 19,02 | 72,18 | 88,31 | 16,13 | 5,00 | 142,80 | 2,73 | 52,34 |
| 3 | MO2 | 515,90 | 97,27 | 16,65 | 88,37 | 104,89 | 16,52 | 8,00 | 224,00 | 3,05 | 56,58 |
| 3 | MO2 | 454,18 | 95,88 | 18,48 | 82,58 | 97,86 | 15,28 | 6,00 | 204,27 | 2,01 | 55,02 |
| 3 | MO2 | 375,08 | 89,18 | 15,56 | 77,85 | 92,83 | 14,98 | 7,00 | 126,90 | 2,41 | 66,17 |
| 3 | MO2 | 333,38 | 86,68 | 17,81 | 78,54 | 88,75 | 10,21 | 7,00 | 147,96 | 4,00 | 55,62 |
| 3 | MO3 | 356,52 | 81,07 | 21,71 | 76,45 | 96,54 | 20,09 | 6,00 | 163,00 | 2,34 | 54,28 |
| 3 | MO3 | 444,39 | 97,58 | 16,69 | 85,22 | 108,55 | 23,33 | 7,00 | 208,11 | 2,24 | 53,17 |
| 3 | MO3 | 398,28 | 91,06 | 19,66 | 84,51 | 101,72 | 17,21 | 6,00 | 159,54 | 2,16 | 59,94 |
| 3 | MO3 | 312,41 | 86,48 | 19,60 | 73,03 | 93,03 | 20,00 | 6,00 | 159,47 | 3,57 | 48,95 |
| 3 | MO3 | 359,96 | 92,55 | 18,63 | 76,60 | 97,25 | 20,65 | 7,00 | 166,43 | 1,96 | 53,76 |
| 3 | MO3 | 352,60 | 88,85 | 18,29 | 77,10 | 94,95 | 17,85 | 7,00 | 149,30 | 1,14 | 57,66 |
| 3 | MO3 | 314,44 | 88,93 | 17,27 | 76,53 | 95,11 | 18,58 | 5,00 | 159,16 | 2,55 | 49,38 |
| 3 | MO3 | 307,30 | 91,79 | 19,88 | 80,51 | 98,10 | 17,59 | 6,00 | 137,60 | 1,05 | 55,22 |
| 3 | MO3 | 245,15 | 77,55 | 14,54 | 68,72 | 87,28 | 18,56 | 7,00 | 107,70 | 3,52 | 56,07 |
| 3 | MO3 | 413,96 | 92,94 | 22,73 | 76,91 | 102,07 | 25,16 | 7,00 | 145,46 | 2,33 | 64,86 |
| 3 | MO4 | 436,26 | 98,53 | 23,22 | 82,90 | 97,26 | 14,36 | 8,00 | 197,37 | 4,15 | 54,76 |
| 3 | MO4 | 343,31 | 89,84 | 22,89 | 78,43 | 94,61 | 16,18 | 7,00 | 150,53 | 4,00 | 56,15 |
| 3 | MO4 | 325,62 | 88,27 | 18,92 | 75,48 | 83,32 | 7,84 | 7,00 | 121,29 | 3,72 | 62,75 |
| 3 | MO4 | 348,68 | 88,99 | 21,00 | 76,66 | 93,38 | 16,72 | 6,00 | 143,61 | 4,10 | 58,81 |
| 3 | MO4 | 395,70 | 91,22 | 19,31 | 80,57 | 93,42 | 12,85 | 6,00 | 155,24 | 4,91 | 60,77 |
| 3 | MO4 | 254,12 | 83,00 | 20,70 | 70,92 | 87,89 | 16,97 | 7,00 | 113,07 | 4,21 | 55,51 |
| 3 | MO4 | 363,60 | 90,71 | 22,24 | 80,90 | 95,79 | 14,89 | 8,00 | 152,15 | 3,74 | 58,15 |
| 3 | MO4 | 320,91 | 88,90 | 14,54 | 73,13 | 86,33 | 13,20 | 7,00 | 130,19 | 3,91 | 59,43 |
| 3 | MO4 | 300,11 | 88,03 | 22,76 | 72,42 | 90,65 | 18,23 | 7,00 | 150,40 | 6,17 | 49,89 |
| 3 | MO4 | 465,90 | 97,24 | 19,06 | 83,97 | 102,26 | 18,29 | 6,00 | 220,95 | 5,31 | 52,58 |
| 3 | MO5 | 608,50 | 110,06 | 22,08 | 92,23 | 102,74 | 10,51 | 6,00 | 243,11 | 2,74 | 60,05 |
| 3 | MO5 | 313,86 | 87,70 | 15,74 | 72,86 | 88,25 | 15,39 | 7,00 | 145,01 | 2,67 | 53,80 |
| 3 | MO5 | 313,27 | 87,64 | 16,87 | 76,23 | 96,15 | 19,92 | 7,00 | 159,65 | 2,65 | 49,04 |
| 3 | MO5 | 454,22 | 94,45 | 22,87 | 85,54 | 106,80 | 21,26 | 7,00 | 198,17 | 3,26 | 56,37 |
| 3 | MO5 | 300,15 | 87,81 | 16,58 | 70,47 | 86,75 | 16,28 | 7,00 | 113,80 | 1,49 | 62,09 |
| 3 | MO5 | 368,95 | 89,80 | 19,91 | 78,43 | 95,22 | 16,79 | 6,00 | 118,20 | 3,17 | 67,96 |
| 3 | MO5 | 310,31 | 85,36 | 17,62 | 74,15 | 91,41 | 17,26 | 6,00 | 137,97 | 1,91 | 55,54 |
| 3 | MO5 | 386,62 | 92,14 | 17,08 | 80,35 | 96,09 | 15,74 | 7,00 | 147,62 | 1,48 | 61,82 |
| 3 | MO5 | 305,11 | 80,57 | 19,35 | 71,27 | 90,55 | 19,28 | 6,00 | 126,47 | 2,52 | 58,55 |
| 3 | MO5 | 315,72 | 84,10 | 19,50 | 75,34 | 86,54 | 11,20 | 7,00 | 136,72 | 4,26 | 56,70 |
| 3 | MO6 | 376,78 | 97,28 | 19,01 | 78,23 | 86,38 | 8,15 | 6,00 | 141,35 | 0,95 | 62,48 |
| 3 | MO6 | 535,22 | 104,18 | 18,02 | 85,78 | 100,48 | 14,70 | 7,00 | 229,80 | 4,16 | 57,06 |
| 3 | MO6 | 503,04 | 100,92 | 20,81 | 89,65 | 107,58 | 17,93 | 7,00 | 222,95 | 3,63 | 55,68 |
| 3 | MO6 | 392,87 | 104,11 | 23,05 | 84,30 | 97,93 | 13,63 | 7,00 | 165,32 | 2,26 | 57,92 |
| 3 | MO6 | 433,32 | 101,50 | 26,66 | 78,07 | 102,22 | 24,15 | 6,00 | 216,48 | 2,95 | 50,04 |
| 3 | MO6 | 509,90 | 87,87 | 18,72 | 74,34 | 90,54 | 16,20 | 6,00 | 157,19 | 2,65 | 69,17 |
| 3 | MO6 | 442,71 | 100,01 | 23,52 | 78,77 | 94,53 | 15,76 | 7,00 | 168,79 | 4,03 | 61,87 |
| 3 | MO6 | 327,02 | 89,10 | 12,07 | 74,67 | 85,39 | 10,72 | 6,00 | 125,33 | 2,17 | 61,68 |
| 3 | MO6 | 509,91 | 104,00 | 16,54 | 89,01 | 106,98 | 17,97 | 8,00 | 216,82 | 2,91 | 57,48 |
| 3 | MO6 | 387,85 | 91,32 | 14,68 | 80,62 | 97,64 | 17,02 | 6,00 | 133,31 | 2,45 | 65,63 |
| 3 | AB1 | 386,99 | 89,88 | 17,38 | 84,15 | 97,08 | 12,93 | 8,00 | 198,20 | 4,27 | 48,78 |
| 3 | AB1 | 444,17 | 95,31 | 20,01 | 83,81 | 96,58 | 12,77 | 6,00 | 215,71 | 5,18 | 51,44 |
| 3 | AB1 | 290,82 | 83,42 | 17,45 | 76,70 | 84,99 | 8,29 | 8,00 | 138,15 | 3,81 | 52,50 |
| 3 | AB1 | 445,80 | 94,62 | 15,54 | 87,91 | 94,04 | 6,13 | 9,00 | 196,01 | 3,82 | 56,03 |
| 3 | AB1 | 360,04 | 95,88 | 18,06 | 75,92 | 91,70 | 15,78 | 8,00 | 169,03 | 3,13 | 53,05 |
| 3 | AB1 | 353,71 | 89,57 | 15,36 | 78,26 | 93,62 | 15,36 | 7,00 | 163,41 | 4,13 | 53,80 |
| 3 | AB1 | 347,49 | 85,41 | 16,48 | 75,15 | 90,80 | 15,65 | 6,00 | 156,76 | 4,21 | 54,89 |
| 3 | AB1 | 360,11 | 88,71 | 23,71 | 79,11 | 90,32 | 11,21 | 8,00 | 186,49 | 3,22 | 48,21 |
| 3 | AB1 | 385,66 | 90,45 | 17,43 | 80,98 | 95,99 | 15,01 | 6,00 | 173,88 | 1,13 | 54,91 |
| 3 | AB1 | 444,22 | 94,68 | 17,75 | 87,92 | 99,24 | 11,32 | 8,00 | 225,65 | 4,45 | 49,20 |
| 3 | PB1 | 432,57 | 93,90 | 16,48 | 85,56 | 94,25 | 8,69 | 7,00 | 225,47 | 4,07 | 47,88 |
| 3 | PB1 | 563,86 | 108,85 | 16,32 | 89,91 | 104,64 | 14,73 | 9,00 | 229,21 | 1,85 | 59,35 |
| 3 | PB1 | 416,30 | 96,41 | 17,53 | 81,34 | 94,69 | 13,35 | 7,00 | 195,11 | 3,16 | 53,13 |
| 3 | PTB1 | 428,07 | 96,13 | 19,57 | 84,01 | 104,30 | 20,29 | 5,00 | 200,87 | 3,06 | 53,08 |
| 3 | PTB1 | 491,75 | 104,60 | 17,07 | 82,93 | 95,59 | 12,66 | 5,00 | 221,72 | 2,17 | 54,91 |
| 3 | PTB1 | 577,22 | 109,60 | 16,35 | 88,90 | 106,70 | 17,80 | 6,00 | 320,31 | 7,01 | 44,51 |
| 3 | PTB1 | 417,38 | 96,62 | 15,81 | 81,32 | 89,58 | 8,26 | 6,00 | 210,05 | 0,87 | 49,67 |
| 3 | PTB1 | 436,76 | 98,80 | 13,81 | 82,89 | 98,83 | 15,94 | 7,00 | 157,70 | 1,91 | 63,89 |
| 3 | PTB1 | 324,49 | 91,06 | 16,39 | 74,55 | 92,22 | 17,67 | 7,00 | 155,45 | 1,47 | 52,09 |
| 3 | PTB1 | 325,01 | 91,13 | 15,50 | 74,71 | 88,99 | 14,28 | 7,00 | 156,11 | 2,13 | 51,97 |
| 3 | PTB1 | 563,21 | 106,59 | 20,69 | 88,22 | 106,27 | 18,05 | 6,00 | 226,36 | 1,48 | 59,81 |
| 3 | PTB1 | 493,68 | 104,15 | 16,25 | 85,90 | 105,04 | 19,14 | 7,00 | 263,07 | 2,96 | 46,71 |
| 3 | PTB1 | 669,90 | 109,92 | 16,91 | 94,52 | 117,54 | 23,02 | 7,00 | 268,02 | 2,31 | 59,99 |
| 3 | SFB1 | 468,66 | 96,71 | 20,56 | 90,70 | 104,06 | 13,36 | 6,00 | 165,66 | 2,87 | 64,65 |
| 3 | SFB1 | 218,28 | 72,39 | 18,93 | 66,77 | 84,35 | 17,58 | 7,00 | 101,34 | 2,80 | 53,57 |
| 3 | SFB1 | 425,98 | 96,33 | 19,77 | 82,56 | 83,85 | 1,29 | 7,00 | 171,32 | 3,69 | 59,78 |
| 3 | SFB1 | 263,93 | 79,05 | 16,72 | 74,28 | 84,77 | 10,49 | 6,00 | 112,25 | 1,85 | 57,47 |
| 3 | PTO2 | 265,35 | 85,74 | 23,46 | 72,14 | 94,20 | 22,06 | 5,00 | 142,76 | 3,64 | 46,20 |
| 3 | PTO2 | 355,83 | 98,11 | 16,49 | 76,45 | 96,87 | 20,42 | 5,00 | 99,20 | 0,76 | 72,12 |
| 3 | PTO2 | 627,70 | 116,82 | 19,79 | 93,94 | 114,30 | 20,36 | 6,00 | 239,63 | 3,18 | 61,82 |
| 3 | PTO2 | 392,36 | 97,77 | 20,89 | 82,28 | 101,60 | 19,32 | 6,00 | 155,45 | 3,20 | 60,38 |
| 3 | PTO2 | 547,53 | 107,39 | 17,10 | 91,31 | 115,87 | 24,56 | 7,00 | 235,51 | 1,67 | 56,99 |
| 3 | PTO2 | 443,33 | 103,31 | 17,91 | 80,29 | 90,01 | 9,72 | 7,00 | 122,65 | 2,02 | 72,33 |
| 3 | PTO2 | 610,50 | 115,41 | 21,87 | 91,75 | 112,83 | 21,08 | 7,00 | 248,93 | 2,72 | 59,23 |
| 3 | PTO2 | 457,87 | 101,75 | 20,47 | 86,36 | 106,15 | 19,79 | 7,00 | 163,60 | 2,85 | 64,27 |
| 3 | PTO2 | 361,86 | 93,73 | 18,39 | 79,20 | 98,79 | 19,59 | 7,00 | 160,30 | 3,41 | 55,70 |
| 3 | PTO2 | 424,79 | 98,86 | 15,72 | 87,67 | 109,37 | 21,70 | 6,00 | 161,89 | 2,77 | 61,89 |
| 3 | PTO3 | 428,99 | 96,47 | 18,46 | 80,75 | 98,72 | 17,97 | 6,00 | 163,75 | 4,64 | 61,83 |
| 3 | PTO3 | 476,93 | 101,92 | 20,80 | 83,44 | 102,93 | 19,49 | 6,00 | 197,24 | 3,34 | 58,64 |
| 3 | PTO3 | 411,49 | 95,00 | 17,64 | 75,56 | 96,00 | 20,44 | 7,00 | 148,38 | 3,32 | 63,94 |
| 3 | PTO3 | 381,26 | 90,77 | 20,16 | 78,43 | 97,03 | 18,60 | 6,00 | 128,07 | 3,01 | 66,41 |
| 3 | PTO3 | 520,28 | 103,35 | 19,80 | 88,93 | 103,90 | 14,97 | 7,00 | 179,14 | 3,83 | 65,57 |
| 3 | PTO3 | 533,67 | 109,10 | 22,31 | 89,88 | 106,84 | 16,96 | 7,00 | 181,53 | 3,04 | 65,98 |
| 3 | PTO3 | 467,26 | 115,00 | 20,44 | 83,33 | 101,88 | 18,55 | 7,00 | 169,03 | 3,15 | 63,83 |
| 3 | PTO3 | 456,84 | 97,80 | 18,77 | 83,17 | 95,58 | 12,41 | 7,00 | 221,26 | 5,29 | 51,57 |
| 3 | PTO3 | 396,58 | 88,85 | 15,52 | 83,95 | 105,95 | 22,00 | 6,00 | 160,83 | 3,90 | 59,45 |
| 3 | PTO3 | 540,19 | 105,30 | 16,98 | 89,34 | 107,88 | 18,54 | 8,00 | 171,79 | 3,99 | 68,20 |
| 3 | PTO6 | 374,41 | 91,32 | 18,01 | 75,60 | 95,71 | 20,11 | 7,00 | 163,52 | 2,15 | 56,33 |
| 3 | PTO6 | 415,21 | 99,82 | 21,14 | 77,92 | 100,20 | 22,28 | 6,00 | 152,56 | 1,90 | 63,26 |
| 3 | PTO6 | 420,93 | 92,25 | 17,10 | 83,27 | 104,32 | 21,05 | 6,00 | 175,68 | 1,89 | 58,26 |
| 3 | PDO2 | 406,77 | 86,72 | 14,22 | 83,39 | 101,53 | 18,14 | 7,00 | 148,37 | 0,70 | 63,52 |
| 3 | PDO2 | 629,30 | 112,40 | 17,02 | 90,38 | 108,61 | 18,23 | 8,00 | 242,02 | 1,98 | 61,54 |
| 3 | PDO2 | 323,91 | 88,10 | 19,38 | 73,53 | 93,92 | 20,39 | 7,00 | 115,17 | 3,28 | 64,44 |
| 3 | PDO2 | 665,80 | 109,74 | 18,52 | 94,91 | 113,93 | 19,02 | 7,00 | 271,90 | 1,65 | 59,16 |
| 3 | PDO2 | 496,96 | 105,58 | 14,78 | 88,04 | 97,45 | 9,41 | 7,00 | 227,66 | 2,68 | 54,19 |
| 3 | PDO2 | 451,45 | 95,41 | 14,77 | 84,40 | 98,80 | 14,40 | 7,00 | 186,76 | 1,55 | 58,63 |
| 3 | PDO2 | 366,51 | 93,80 | 13,26 | 75,85 | 91,35 | 15,50 | 6,00 | 146,24 | 1,14 | 60,10 |
| 3 | PDO2 | 482,41 | 101,72 | 19,83 | 86,59 | 101,21 | 14,62 | 7,00 | 215,95 | 1,54 | 55,24 |
| 3 | PDO2 | 378,59 | 90,30 | 14,77 | 85,21 | 101,48 | 16,27 | 5,00 | 154,90 | 2,22 | 59,09 |
| 3 | CRO1 | 491,08 | 99,57 | 15,40 | 85,86 | 105,24 | 19,38 | 7,00 | 170,52 | 2,16 | 65,28 |
| 3 | CRO1 | 347,52 | 86,18 | 17,54 | 74,33 | 91,70 | 17,37 | 6,00 | 122,05 | 4,25 | 64,88 |
| 3 | CRO1 | 618,00 | 109,37 | 15,56 | 92,59 | 105,03 | 12,44 | 6,00 | 246,93 | 3,35 | 60,04 |
| 3 | CRO1 | 445,90 | 92,39 | 25,14 | 81,35 | 100,44 | 19,09 | 6,00 | 175,80 | 3,48 | 60,57 |
| 3 | CRO1 | 536,67 | 105,04 | 19,66 | 86,06 | 96,90 | 10,84 | 7,00 | 163,39 | 5,90 | 69,55 |
| 3 | CRO1 | 534,85 | 103,09 | 20,55 | 85,84 | 103,75 | 17,91 | 7,00 | 188,04 | 2,61 | 64,84 |
| 3 | CRO1 | 368,52 | 97,28 | 19,00 | 73,16 | 85,87 | 12,71 | 7,00 | 114,90 | 1,82 | 68,82 |
| 3 | CRO1 | 517,37 | 100,28 | 17,03 | 87,88 | 100,06 | 12,18 | 6,00 | 173,29 | 0,85 | 66,51 |
| 3 | CRO1 | 369,39 | 86,81 | 16,75 | 77,22 | 87,69 | 10,47 | 6,00 | 179,79 | 2,13 | 51,33 |
| 3 | CRO1 | 823,80 | 117,68 | 22,62 | 104,95 | 120,14 | 15,19 | 7,00 | 259,42 | 1,77 | 68,51 |
| 3 | CRO2 | 326,23 | 84,62 | 17,89 | 75,34 | 96,34 | 21,00 | 6,00 | 123,61 | 3,34 | 62,11 |
| 3 | CRO2 | 462,70 | 94,76 | 21,61 | 86,31 | 105,56 | 19,25 | 7,00 | 170,61 | 1,91 | 63,13 |
| 3 | CRO2 | 336,36 | 92,38 | 20,67 | 74,67 | 97,35 | 22,68 | 7,00 | 146,86 | 3,57 | 56,34 |
| 3 | CRO2 | 397,16 | 95,03 | 22,67 | 75,45 | 86,90 | 11,45 | 6,00 | 156,10 | 3,41 | 60,70 |
| 3 | CRO2 | 335,89 | 91,18 | 20,66 | 71,47 | 91,65 | 20,18 | 7,00 | 142,63 | 3,40 | 57,54 |
| 3 | CRO2 | 282,89 | 83,24 | 16,07 | 73,64 | 82,46 | 8,82 | 7,00 | 108,06 | 1,80 | 61,80 |
| 3 | CRO2 | 255,44 | 79,85 | 19,02 | 72,37 | 92,23 | 19,86 | 6,00 | 124,80 | 0,90 | 51,14 |
| 3 | CRO2 | 349,22 | 87,97 | 20,90 | 77,45 | 96,73 | 19,28 | 8,00 | 137,00 | 4,57 | 60,77 |
| 3 | CRO2 | 462,56 | 95,11 | 20,03 | 87,07 | 102,60 | 15,53 | 8,00 | 183,15 | 4,63 | 60,41 |
| 3 | CRO2 | 409,23 | 90,02 | 24,51 | 80,32 | 101,83 | 21,51 | 6,00 | 187,77 | 3,69 | 54,12 |
| 3 | ADO3 | 241,21 | 81,99 | 17,20 | 64,60 | 71,65 | 7,05 | 5,00 | 91,25 | 2,44 | 62,17 |
| 3 | ADO3 | 402,05 | 94,34 | 19,72 | 82,87 | 97,84 | 14,97 | 7,00 | 141,15 | 2,80 | 64,89 |
| 3 | ADO3 | 407,83 | 95,34 | 15,01 | 84,66 | 99,46 | 14,80 | 9,00 | 136,50 | 2,61 | 66,53 |
| 3 | ADO3 | 444,08 | 98,96 | 15,52 | 82,93 | 93,50 | 10,57 | 6,00 | 132,00 | 3,06 | 70,28 |
| 3 | ADO3 | 374,50 | 92,79 | 16,89 | 73,05 | 94,09 | 21,04 | 6,00 | 148,30 | 2,61 | 60,40 |
| 3 | ADO3 | 423,90 | 99,77 | 17,16 | 80,09 | 98,81 | 18,72 | 7,00 | 140,65 | 3,05 | 66,82 |
| 3 | ADO3 | 669,20 | 108,36 | 21,86 | 95,94 | 106,27 | 10,33 | 7,00 | 210,40 | 2,59 | 68,56 |
| 3 | ADO3 | 471,00 | 90,85 | 13,15 | 70,60 | 74,06 | 3,46 | 7,00 | 172,01 | 4,27 | 63,48 |
| 3 | ADO3 | 482,00 | 98,95 | 18,29 | 85,36 | 106,82 | 21,46 | 7,00 | 150,08 | 2,00 | 68,86 |
| 3 | ADO3 | 635,00 | 111,76 | 18,88 | 88,76 | 98,76 | 10,00 | 8,00 | 198,62 | 2,77 | 68,72 |
| 3 | BO1 | 418,75 | 98,50 | 22,81 | 78,29 | 98,72 | 20,43 | 6,00 | 236,57 | 4,33 | 43,51 |
| 3 | BO1 | 401,46 | 94,18 | 21,62 | 77,64 | 96,86 | 19,22 | 7,00 | 171,17 | 3,89 | 57,36 |
| 3 | BO1 | 362,62 | 92,86 | 22,06 | 76,34 | 92,90 | 16,56 | 8,00 | 205,57 | 5,56 | 43,31 |
| 3 | BO1 | 441,95 | 96,03 | 25,48 | 80,37 | 104,91 | 24,54 | 6,00 | 235,09 | 4,40 | 46,81 |
| 3 | BO1 | 304,55 | 85,99 | 19,03 | 74,24 | 89,84 | 15,60 | 7,00 | 148,35 | 3,09 | 51,29 |
| 3 | BO1 | 441,29 | 95,75 | 22,39 | 79,24 | 104,56 | 25,32 | 7,00 | 189,64 | 4,01 | 57,03 |
| 3 | BO1 | 383,87 | 91,94 | 27,42 | 74,69 | 96,28 | 21,59 | 6,00 | 212,89 | 5,23 | 44,54 |
| 3 | BO1 | 388,70 | 91,04 | 21,11 | 78,71 | 99,97 | 21,26 | 7,00 | 203,39 | 5,01 | 47,67 |
| 3 | BO1 | 551,35 | 103,87 | 23,88 | 90,24 | 113,46 | 23,22 | 7,00 | 255,48 | 4,42 | 53,66 |
| 3 | BO1 | 414,33 | 93,57 | 21,38 | 81,68 | 103,79 | 22,11 | 8,00 | 139,90 | 3,00 | 66,23 |
| 3 | ADO2 | 393,25 | 93,72 | 18,30 | 76,75 | 97,02 | 20,27 | 7,00 | 129,37 | 3,42 | 67,10 |
| 3 | ADO2 | 416,74 | 99,71 | 22,40 | 79,08 | 99,19 | 20,11 | 7,00 | 166,13 | 4,84 | 60,14 |
| 3 | ADO2 | 525,86 | 101,37 | 16,44 | 89,91 | 110,06 | 20,15 | 7,00 | 165,63 | 2,38 | 68,50 |
| 3 | ADO2 | 337,38 | 85,95 | 18,27 | 72,84 | 93,71 | 20,87 | 7,00 | 144,81 | 3,04 | 57,08 |
| 3 | ADO2 | 215,94 | 76,81 | 18,98 | 65,83 | 83,43 | 17,60 | 6,00 | 83,48 | 1,83 | 61,34 |
| 3 | ADO2 | 448,12 | 99,58 | 18,83 | 83,67 | 105,25 | 21,58 | 7,00 | 176,04 | 4,75 | 60,72 |
| 3 | ADO2 | 230,92 | 97,58 | 17,95 | 82,64 | 102,97 | 20,33 | 7,00 | 115,48 | 3,44 | 49,99 |
| 3 | ADO2 | 461,98 | 74,97 | 19,38 | 66,25 | 87,24 | 20,99 | 6,00 | 197,16 | 3,40 | 57,32 |
| 3 | ADO2 | 370,23 | 87,95 | 18,79 | 79,02 | 103,92 | 24,90 | 7,00 | 117,42 | 2,09 | 68,28 |
| 3 | ADO2 | 385,70 | 94,70 | 17,40 | 79,14 | 99,55 | 20,41 | 7,00 | 146,63 | 5,00 | 61,98 |
| 3 | PG | 241,55 | 78,99 | 16,38 | 65,92 | 84,88 | 18,96 | 4,00 | 107,70 | 5,77 | 55,41 |
| 3 | PG | 262,03 | 79,34 | 16,17 | 70,06 | 87,61 | 17,55 | 6,00 | 111,60 | 3,99 | 57,41 |
| 3 | PG | 432,98 | 96,49 | 20,59 | 78,53 | 95,30 | 16,77 | 6,00 | 160,26 | 5,20 | 62,99 |
| 3 | PG | 410,42 | 95,98 | 18,36 | 84,79 | 100,85 | 16,06 | 6,00 | 176,63 | 4,21 | 56,96 |
| 3 | PG | 322,80 | 90,09 | 23,86 | 74,56 | 91,25 | 16,69 | 7,00 | 115,38 | 5,24 | 64,26 |
| 3 | PG | 204,34 | 77,04 | 14,55 | 69,01 | 84,81 | 15,80 | 5,00 | 88,70 | 2,42 | 56,59 |
| 3 | PG | 439,37 | 96,25 | 19,70 | 81,19 | 98,69 | 17,50 | 7,00 | 159,47 | 3,46 | 63,70 |
| 3 | PG | 250,49 | 78,88 | 19,75 | 69,15 | 85,14 | 15,99 | 6,00 | 146,54 | 3,39 | 41,50 |
| 3 | PG | 339,99 | 94,76 | 18,43 | 72,56 | 93,22 | 20,66 | 8,00 | 159,83 | 2,35 | 52,99 |
| 3 | PG | 186,52 | 71,73 | 17,30 | 64,33 | 82,14 | 17,81 | 4,00 | 108,13 | 6,44 | 42,03 |

**Raw Data**

**Data from aril and seed characteristics of pomegranate accessions**

| Period | Variety | SW | SL | Sw | L | W | Wpw | Wpi |
| --- | --- | --- | --- | --- | --- | --- | --- | --- |
| 1 | ME1 | 0,441 | 10,81 | 8,31 | 6,92 | 2,45 | 0,0442 | 10,02 |
| 1 | ME1 | 0,411 | 10,87 | 6,32 | 8,10 | 3,15 | 0,0531 | 12,92 |
| 1 | ME1 | 0,429 | 10,51 | 8,73 | 7,66 | 2,99 | 0,0382 | 8,90 |
| 1 | ME1 | 0,459 | 12,20 | 8,44 | 7,47 | 2,74 | 0,0434 | 9,46 |
| 1 | ME1 | 0,523 | 11,37 | 8,51 | 6,28 | 2,53 | 0,0385 | 7,36 |
| 1 | ME1 | 0,379 | 11,44 | 7,60 | 7,12 | 3,27 | 0,0533 | 14,06 |
| 1 | ME1 | 0,465 | 11,07 | 7,88 | 7,36 | 2,90 | 0,0521 | 11,20 |
| 1 | ME1 | 0,443 | 11,80 | 7,53 | 7,84 | 2,80 | 0,0371 | 8,37 |
| 1 | ME1 | 0,377 | 10,82 | 8,84 | 5,75 | 2,66 | 0,0263 | 6,98 |
| 1 | ME1 | 0,421 | 10,00 | 7,05 | 7,25 | 3,15 | 0,0339 | 8,05 |
| 1 | ME1 | 0,364 | 11,00 | 6,43 | 5,64 | 2,51 | 0,0447 | 12,28 |
| 1 | ME1 | 0,404 | 9,56 | 6,47 | 6,90 | 2,25 | 0,0257 | 6,36 |
| 1 | ME1 | 0,443 | 9,86 | 8,30 | 6,71 | 3,53 | 0,0397 | 8,96 |
| 1 | ME1 | 0,402 | 10,37 | 7,09 | 6,46 | 2,93 | 0,0393 | 9,78 |
| 1 | ME1 | 0,436 | 10,35 | 6,69 | 6,70 | 3,06 | 0,0311 | 7,13 |
| 1 | ME1 | 0,443 | 10,34 | 7,29 | 6,50 | 2,86 | 0,0321 | 7,25 |
| 1 | ME1 | 0,374 | 11,20 | 7,87 | 6,81 | 2,45 | 0,0268 | 7,17 |
| 1 | ME1 | 0,435 | 11,88 | 6,49 | 7,19 | 2,39 | 0,0413 | 9,49 |
| 1 | ME1 | 0,450 | 11,24 | 8,54 | 6,10 | 2,79 | 0,0303 | 6,73 |
| 1 | ME1 | 0,278 | 11,08 | 7,68 | 7,11 | 2,22 | 0,0299 | 10,76 |
| 1 | ME1 | 0,415 | 9,90 | 5,13 | 8,17 | 3,20 | 0,0375 | 9,04 |
| 1 | ME1 | 0,325 | 10,69 | 5,96 | 5,77 | 2,43 | 0,0380 | 11,69 |
| 1 | ME1 | 0,389 | 10,56 | 5,95 | 7,23 | 2,88 | 0,0306 | 7,87 |
| 1 | ME1 | 0,285 | 11,02 | 7,20 | 6,58 | 2,41 | 0,0294 | 10,32 |
| 1 | ME1 | 0,415 | 10,85 | 7,40 | 6,76 | 2,32 | 0,0309 | 7,45 |
| 1 | ME2 | 0,441 | 10,12 | 6,30 | 6,74 | 2,21 | 0,0409 | 9,27 |
| 1 | ME2 | 0,394 | 10,95 | 7,64 | 6,23 | 1,73 | 0,0448 | 11,38 |
| 1 | ME2 | 0,346 | 9,16 | 6,80 | 5,33 | 2,65 | 0,0479 | 13,83 |
| 1 | ME2 | 0,424 | 9,80 | 6,98 | 5,01 | 1,77 | 0,0321 | 7,57 |
| 1 | ME2 | 0,408 | 9,09 | 6,19 | 5,64 | 2,24 | 0,0411 | 10,06 |
| 1 | ME2 | 0,486 | 10,04 | 5,12 | 7,14 | 2,98 | 0,0692 | 14,23 |
| 1 | ME2 | 0,286 | 10,23 | 4,04 | 6,26 | 2,34 | 0,0361 | 12,62 |
| 1 | ME2 | 0,413 | 9,75 | 5,59 | 7,00 | 3,72 | 0,0578 | 14,01 |
| 1 | ME2 | 0,393 | 9,64 | 4,21 | 5,01 | 2,70 | 0,0375 | 9,55 |
| 1 | ME2 | 0,453 | 9,66 | 6,18 | 7,62 | 1,90 | 0,0428 | 9,44 |
| 1 | ME2 | 0,405 | 10,79 | 8,01 | 5,26 | 2,25 | 0,0313 | 7,74 |
| 1 | ME2 | 0,484 | 9,68 | 7,34 | 6,05 | 2,83 | 0,0316 | 6,53 |
| 1 | ME2 | 0,371 | 9,78 | 7,81 | 8,05 | 2,37 | 0,0558 | 15,06 |
| 1 | ME2 | 0,459 | 10,59 | 7,17 | 6,53 | 3,12 | 0,0470 | 10,25 |
| 1 | ME2 | 0,371 | 10,38 | 6,62 | 6,56 | 2,79 | 0,0506 | 13,63 |
| 1 | ME2 | 0,383 | 10,34 | 6,94 | 6,89 | 2,51 | 0,0521 | 13,59 |
| 1 | ME2 | 0,445 | 10,15 | 5,42 | 6,77 | 2,62 | 0,0467 | 10,50 |
| 1 | ME2 | 0,410 | 11,23 | 6,71 | 6,78 | 2,94 | 0,0511 | 12,47 |
| 1 | ME2 | 0,353 | 10,83 | 6,64 | 4,88 | 2,85 | 0,0399 | 11,31 |
| 1 | ME2 | 0,405 | 8,97 | 6,89 | 6,97 | 2,85 | 0,0478 | 11,81 |
| 1 | ME2 | 0,413 | 11,28 | 6,96 | 7,47 | 2,41 | 0,0722 | 17,50 |
| 1 | ME2 | 0,375 | 10,66 | 6,79 | 6,41 | 2,41 | 0,0396 | 10,57 |
| 1 | ME2 | 0,446 | 10,13 | 6,58 | 7,63 | 3,09 | 0,0886 | 19,89 |
| 1 | ME2 | 0,332 | 10,54 | 5,32 | 6,21 | 1,96 | 0,0460 | 13,86 |
| 1 | ME2 | 0,261 | 9,18 | 5,87 | 5,55 | 2,05 | 0,0546 | 20,94 |
| 1 | ME3 | 0,365 | 9,54 | 4,05 | 8,92 | 2,13 | 0,0539 | 14,75 |
| 1 | ME3 | 0,379 | 10,93 | 5,75 | 7,53 | 2,40 | 0,0625 | 16,50 |
| 1 | ME3 | 0,444 | 10,65 | 6,01 | 8,41 | 2,92 | 0,0557 | 12,55 |
| 1 | ME3 | 0,284 | 8,64 | 5,27 | 7,05 | 2,07 | 0,0578 | 20,34 |
| 1 | ME3 | 0,425 | 10,34 | 8,39 | 7,64 | 2,35 | 0,0532 | 12,51 |
| 1 | ME3 | 0,371 | 9,33 | 6,36 | 7,45 | 2,86 | 0,0610 | 16,43 |
| 1 | ME3 | 0,329 | 10,04 | 6,30 | 6,95 | 3,16 | 0,0514 | 15,61 |
| 1 | ME3 | 0,384 | 9,96 | 5,29 | 8,24 | 3,17 | 0,0611 | 15,93 |
| 1 | ME3 | 0,487 | 11,30 | 5,62 | 6,42 | 2,64 | 0,0353 | 7,25 |
| 1 | ME3 | 0,488 | 10,71 | 6,86 | 5,94 | 3,54 | 0,0342 | 7,01 |
| 1 | ME3 | 0,371 | 9,39 | 5,00 | 7,58 | 2,83 | 0,0626 | 16,86 |
| 1 | ME3 | 0,457 | 11,94 | 6,61 | 7,68 | 2,08 | 0,0718 | 15,72 |
| 1 | ME3 | 0,424 | 10,92 | 7,34 | 6,27 | 2,68 | 0,0621 | 14,64 |
| 1 | ME3 | 0,364 | 10,86 | 6,55 | 4,96 | 1,32 | 0,0415 | 11,39 |
| 1 | ME3 | 0,415 | 11,30 | 6,74 | 6,64 | 2,93 | 0,0655 | 15,77 |
| 1 | ME3 | 0,362 | 9,23 | 6,35 | 7,83 | 1,97 | 0,0553 | 15,27 |
| 1 | ME3 | 0,418 | 11,17 | 5,42 | 6,95 | 2,22 | 0,0467 | 11,17 |
| 1 | ME3 | 0,354 | 10,37 | 6,00 | 6,76 | 3,02 | 0,0564 | 15,91 |
| 1 | ME3 | 0,399 | 11,20 | 5,38 | 8,27 | 2,05 | 0,0736 | 18,44 |
| 1 | ME3 | 0,377 | 10,73 | 6,70 | 6,60 | 1,97 | 0,0424 | 11,26 |
| 1 | ME3 | 0,447 | 10,26 | 6,53 | 7,45 | 2,19 | 0,0582 | 13,02 |
| 1 | ME3 | 0,301 | 7,26 | 4,41 | 7,35 | 2,49 | 0,0567 | 18,84 |
| 1 | ME3 | 0,463 | 11,66 | 6,13 | 6,24 | 1,96 | 0,0468 | 10,11 |
| 1 | ME3 | 0,416 | 9,78 | 7,63 | 6,14 | 2,74 | 0,0693 | 16,65 |
| 1 | ME3 | 0,397 | 10,23 | 3,96 | 7,57 | 1,98 | 0,0825 | 20,80 |
| 1 | ME31 | 0,396 | 11,42 | 6,15 | 8,36 | 2,74 | 0,0487 | 12,30 |
| 1 | ME31 | 0,352 | 10,53 | 8,71 | 7,28 | 3,57 | 0,0385 | 10,94 |
| 1 | ME31 | 0,366 | 10,81 | 7,37 | 6,37 | 2,49 | 0,0310 | 8,47 |
| 1 | ME31 | 0,406 | 11,78 | 8,34 | 6,39 | 2,63 | 0,0311 | 7,66 |
| 1 | ME31 | 0,364 | 10,37 | 7,04 | 7,82 | 2,69 | 0,0429 | 11,79 |
| 1 | ME31 | 0,445 | 11,42 | 8,69 | 7,04 | 3,49 | 0,0471 | 10,58 |
| 1 | ME31 | 0,398 | 9,52 | 8,61 | 6,69 | 2,37 | 0,0339 | 8,52 |
| 1 | ME31 | 0,439 | 11,52 | 8,34 | 6,79 | 3,08 | 0,0340 | 7,74 |
| 1 | ME31 | 0,448 | 10,58 | 7,96 | 7,18 | 3,03 | 0,0370 | 8,26 |
| 1 | ME31 | 0,325 | 11,63 | 7,60 | 6,87 | 3,23 | 0,0324 | 9,97 |
| 1 | ME31 | 0,420 | 11,76 | 7,75 | 7,10 | 3,81 | 0,0539 | 12,83 |
| 1 | ME31 | 0,315 | 10,19 | 8,75 | 7,98 | 2,66 | 0,0309 | 9,81 |
| 1 | ME31 | 0,327 | 11,31 | 7,29 | 6,33 | 2,82 | 0,0377 | 11,53 |
| 1 | ME31 | 0,400 | 10,93 | 6,82 | 6,15 | 2,70 | 0,0384 | 9,60 |
| 1 | ME31 | 0,419 | 12,01 | 7,45 | 6,75 | 2,17 | 0,0445 | 10,62 |
| 1 | ME31 | 0,412 | 11,63 | 7,49 | 5,59 | 2,29 | 0,0270 | 6,55 |
| 1 | ME31 | 0,285 | 9,26 | 7,35 | 6,52 | 2,81 | 0,0318 | 11,16 |
| 1 | ME31 | 0,387 | 10,74 | 8,42 | 7,03 | 2,77 | 0,0386 | 9,97 |
| 1 | ME31 | 0,438 | 10,82 | 7,44 | 7,12 | 2,57 | 0,0417 | 9,52 |
| 1 | ME31 | 0,459 | 11,25 | 8,41 | 5,71 | 3,30 | 0,0301 | 6,56 |
| 1 | ME31 | 0,367 | 9,73 | 6,93 | 6,59 | 2,77 | 0,0305 | 8,31 |
| 1 | ME31 | 0,293 | 8,68 | 6,53 | 6,71 | 2,69 | 0,0265 | 9,04 |
| 1 | ME31 | 0,269 | 11,13 | 8,49 | 5,32 | 3,06 | 0,0364 | 13,53 |
| 1 | ME31 | 0,395 | 10,93 | 6,38 | 5,98 | 2,82 | 0,0311 | 7,87 |
| 1 | ME31 | 0,361 | 10,38 | 7,35 | 7,02 | 2,10 | 0,0336 | 9,31 |
| 1 | ME4 | 0,381 | 10,33 | 4,82 | 7,76 | 1,58 | 0,0661 | 17,36 |
| 1 | ME4 | 0,346 | 8,97 | 4,17 | 5,48 | 0,78 | 0,0506 | 14,62 |
| 1 | ME4 | 0,498 | 11,49 | 5,68 | 6,66 | 1,45 | 0,0600 | 12,05 |
| 1 | ME4 | 0,503 | 9,65 | 5,98 | 4,36 | 1,60 | 0,0327 | 6,50 |
| 1 | ME4 | 0,456 | 9,29 | 6,93 | 4,97 | 1,52 | 0,0347 | 7,61 |
| 1 | ME4 | 0,381 | 9,64 | 5,64 | 3,92 | 1,58 | 0,0257 | 6,74 |
| 1 | ME4 | 0,417 | 8,67 | 7,36 | 3,84 | 1,04 | 0,0363 | 8,71 |
| 1 | ME4 | 0,489 | 9,32 | 5,68 | 5,31 | 1,76 | 0,0260 | 5,32 |
| 1 | ME4 | 0,527 | 12,43 | 4,94 | 4,79 | 2,40 | 0,0362 | 6,86 |
| 1 | ME4 | 0,358 | 9,75 | 5,20 | 6,02 | 1,57 | 0,0436 | 12,19 |
| 1 | ME4 | 0,484 | 9,91 | 8,51 | 7,67 | 2,82 | 0,0462 | 9,54 |
| 1 | ME4 | 0,293 | 9,80 | 4,57 | 6,42 | 2,12 | 0,0483 | 16,51 |
| 1 | ME4 | 0,459 | 9,64 | 5,71 | 5,37 | 1,78 | 0,0464 | 10,11 |
| 1 | ME4 | 0,385 | 9,30 | 4,55 | 6,34 | 2,30 | 0,0418 | 10,85 |
| 1 | ME4 | 0,552 | 11,20 | 5,54 | 7,01 | 2,02 | 0,0346 | 6,27 |
| 1 | ME4 | 0,324 | 9,16 | 5,96 | 6,80 | 2,60 | 0,0302 | 9,32 |
| 1 | ME4 | 0,415 | 10,51 | 5,07 | 6,52 | 2,75 | 0,0457 | 11,01 |
| 1 | ME4 | 0,438 | 9,87 | 6,24 | 7,02 | 3,09 | 0,0492 | 11,24 |
| 1 | ME4 | 0,397 | 9,04 | 6,44 | 7,04 | 2,04 | 0,0213 | 5,36 |
| 1 | ME4 | 0,450 | 7,84 | 4,86 | 7,46 | 2,62 | 0,0449 | 9,98 |
| 1 | ME4 | 0,442 | 10,31 | 6,17 | 6,97 | 2,19 | 0,0361 | 8,17 |
| 1 | ME4 | 0,325 | 9,77 | 6,58 | 6,63 | 2,33 | 0,0411 | 12,64 |
| 1 | ME4 | 0,388 | 8,88 | 5,49 | 6,34 | 2,82 | 0,0440 | 11,33 |
| 1 | ME4 | 0,337 | 7,81 | 6,98 | 6,15 | 2,88 | 0,0347 | 10,30 |
| 1 | ME4 | 0,474 | 9,98 | 5,62 | 5,83 | 2,93 | 0,0418 | 8,82 |
| 1 | ME5 | 0,352 | 10,41 | 5,73 | 7,76 | 2,70 | 0,0583 | 16,54 |
| 1 | ME5 | 0,408 | 9,67 | 7,79 | 6,41 | 2,09 | 0,0339 | 8,32 |
| 1 | ME5 | 0,449 | 10,23 | 5,42 | 8,09 | 2,91 | 0,0391 | 8,70 |
| 1 | ME5 | 0,368 | 9,44 | 7,10 | 6,82 | 2,18 | 0,0433 | 11,77 |
| 1 | ME5 | 0,382 | 11,64 | 7,50 | 7,49 | 1,80 | 0,0444 | 11,62 |
| 1 | ME5 | 0,302 | 9,96 | 6,42 | 6,63 | 2,87 | 0,0429 | 14,20 |
| 1 | ME5 | 0,395 | 11,89 | 7,04 | 7,48 | 2,48 | 0,0489 | 12,37 |
| 1 | ME5 | 0,495 | 11,76 | 6,26 | 8,45 | 2,07 | 0,0594 | 12,00 |
| 1 | ME5 | 0,399 | 9,80 | 6,44 | 6,62 | 2,31 | 0,0601 | 15,07 |
| 1 | ME5 | 0,469 | 10,08 | 10,79 | 4,83 | 1,50 | 0,0448 | 9,55 |
| 1 | ME5 | 0,231 | 8,87 | 5,59 | 6,35 | 2,16 | 0,0268 | 11,61 |
| 1 | ME5 | 0,289 | 8,41 | 6,25 | 5,36 | 1,95 | 0,0451 | 15,61 |
| 1 | ME5 | 0,473 | 11,68 | 9,51 | 7,21 | 2,17 | 0,0391 | 8,28 |
| 1 | ME5 | 0,421 | 9,98 | 7,09 | 7,30 | 1,94 | 0,0531 | 12,61 |
| 1 | ME5 | 0,352 | 9,16 | 6,40 | 6,36 | 2,21 | 0,0496 | 14,08 |
| 1 | ME5 | 0,246 | 8,53 | 4,69 | 6,44 | 2,75 | 0,0423 | 17,17 |
| 1 | ME5 | 0,398 | 11,26 | 7,67 | 5,86 | 2,21 | 0,0439 | 11,03 |
| 1 | ME5 | 0,355 | 10,21 | 7,28 | 8,29 | 2,18 | 0,0213 | 6,01 |
| 1 | ME5 | 0,349 | 10,91 | 7,08 | 7,16 | 2,81 | 0,0275 | 7,89 |
| 1 | ME5 | 0,452 | 13,08 | 5,84 | 8,87 | 2,90 | 0,0623 | 13,78 |
| 1 | ME5 | 0,275 | 10,10 | 5,79 | 6,30 | 2,87 | 0,0533 | 19,39 |
| 1 | ME5 | 0,395 | 9,96 | 6,02 | 6,66 | 1,63 | 0,0515 | 13,05 |
| 1 | ME5 | 0,375 | 10,63 | 7,65 | 6,14 | 1,74 | 0,0388 | 10,36 |
| 1 | ME5 | 0,347 | 10,78 | 5,45 | 7,13 | 1,84 | 0,0338 | 9,75 |
| 1 | ME5 | 0,378 | 11,36 | 6,76 | 6,17 | 2,11 | 0,0512 | 13,54 |
| 1 | ME6 | 0,435 | 11,36 | 8,23 | 7,74 | 1,71 | 0,0449 | 10,33 |
| 1 | ME6 | 0,406 | 10,86 | 8,74 | 5,18 | 1,78 | 0,0234 | 5,76 |
| 1 | ME6 | 0,400 | 11,74 | 8,60 | 7,00 | 2,71 | 0,0316 | 7,90 |
| 1 | ME6 | 0,367 | 11,68 | 7,22 | 7,64 | 2,35 | 0,0579 | 15,76 |
| 1 | ME6 | 0,237 | 8,73 | 7,69 | 5,83 | 3,35 | 0,0203 | 8,57 |
| 1 | ME6 | 0,424 | 10,62 | 7,58 | 6,86 | 1,57 | 0,0296 | 6,98 |
| 1 | ME6 | 0,421 | 11,73 | 6,62 | 7,31 | 1,95 | 0,0317 | 7,52 |
| 1 | ME6 | 0,494 | 11,19 | 7,93 | 5,57 | 2,42 | 0,0272 | 5,51 |
| 1 | ME6 | 0,434 | 10,86 | 8,44 | 6,63 | 2,12 | 0,0359 | 8,28 |
| 1 | ME6 | 0,364 | 10,32 | 7,21 | 7,34 | 2,66 | 0,0240 | 6,59 |
| 1 | ME6 | 0,461 | 11,85 | 8,69 | 6,41 | 2,97 | 0,0200 | 4,34 |
| 1 | ME6 | 0,416 | 10,90 | 8,65 | 6,74 | 1,40 | 0,0309 | 7,43 |
| 1 | ME6 | 0,233 | 9,23 | 6,84 | 5,70 | 2,80 | 0,0179 | 7,70 |
| 1 | ME6 | 0,427 | 9,60 | 7,72 | 7,31 | 1,70 | 0,0250 | 5,85 |
| 1 | ME6 | 0,442 | 11,47 | 9,39 | 6,33 | 1,90 | 0,0253 | 5,73 |
| 1 | ME6 | 0,348 | 10,88 | 7,93 | 6,83 | 1,50 | 0,0259 | 7,44 |
| 1 | ME6 | 0,321 | 9,95 | 8,40 | 6,48 | 1,81 | 0,0263 | 8,20 |
| 1 | ME6 | 0,343 | 11,24 | 7,66 | 6,34 | 1,60 | 0,0340 | 9,92 |
| 1 | ME6 | 0,450 | 10,64 | 7,92 | 7,16 | 2,42 | 0,0233 | 5,18 |
| 1 | ME6 | 0,487 | 10,85 | 8,14 | 6,94 | 2,82 | 0,0286 | 5,87 |
| 1 | ME6 | 0,465 | 9,42 | 5,80 | 5,01 | 1,30 | 0,0258 | 5,55 |
| 1 | ME6 | 0,409 | 10,01 | 7,81 | 7,30 | 2,70 | 0,0288 | 7,04 |
| 1 | ME6 | 0,435 | 10,25 | 6,48 | 5,92 | 3,05 | 0,0440 | 10,11 |
| 1 | ME6 | 0,380 | 10,46 | 7,93 | 6,46 | 2,96 | 0,0227 | 5,98 |
| 1 | ME6 | 0,251 | 10,87 | 8,89 | 7,40 | 3,06 | 0,0350 | 13,97 |
| 1 | ME7 | 0,336 | 11,70 | 6,30 | 7,81 | 3,84 | 0,0489 | 14,54 |
| 1 | ME7 | 0,359 | 10,32 | 7,68 | 7,50 | 3,62 | 0,0400 | 11,14 |
| 1 | ME7 | 0,343 | 10,72 | 7,87 | 6,01 | 2,85 | 0,0381 | 11,11 |
| 1 | ME7 | 0,330 | 11,77 | 6,88 | 7,10 | 2,42 | 0,0410 | 12,44 |
| 1 | ME7 | 0,528 | 11,97 | 9,32 | 7,50 | 3,07 | 0,0470 | 8,91 |
| 1 | ME7 | 0,244 | 10,03 | 6,37 | 7,49 | 2,14 | 0,0334 | 13,67 |
| 1 | ME7 | 0,298 | 9,48 | 8,40 | 5,54 | 3,15 | 0,0315 | 10,57 |
| 1 | ME7 | 0,253 | 8,08 | 4,64 | 6,62 | 3,75 | 0,0394 | 15,60 |
| 1 | ME7 | 0,263 | 9,90 | 4,68 | 6,23 | 3,21 | 0,0458 | 17,39 |
| 1 | ME7 | 0,337 | 10,18 | 6,29 | 6,50 | 3,21 | 0,0307 | 9,10 |
| 1 | ME7 | 0,294 | 10,99 | 7,08 | 6,59 | 2,55 | 0,0258 | 8,79 |
| 1 | ME7 | 0,372 | 12,05 | 8,11 | 7,66 | 1,90 | 0,0270 | 7,27 |
| 1 | ME7 | 0,526 | 11,11 | 8,42 | 6,43 | 3,32 | 0,0304 | 5,78 |
| 1 | ME7 | 0,341 | 10,71 | 7,32 | 7,15 | 2,45 | 0,0210 | 6,16 |
| 1 | ME7 | 0,426 | 12,02 | 8,18 | 7,45 | 2,66 | 0,0271 | 6,36 |
| 1 | ME7 | 0,293 | 12,21 | 5,57 | 7,23 | 2,67 | 0,0477 | 16,30 |
| 1 | ME7 | 0,287 | 10,14 | 5,55 | 7,43 | 2,92 | 0,0490 | 17,06 |
| 1 | ME7 | 0,382 | 11,09 | 8,49 | 6,77 | 2,33 | 0,0331 | 8,67 |
| 1 | ME7 | 0,535 | 11,76 | 8,86 | 6,11 | 3,93 | 0,0404 | 7,55 |
| 1 | ME7 | 0,258 | 10,41 | 7,22 | 6,92 | 2,12 | 0,0399 | 15,47 |
| 1 | ME7 | 0,363 | 11,09 | 8,07 | 5,58 | 2,70 | 0,0337 | 9,29 |
| 1 | ME7 | 0,162 | 8,86 | 7,04 | 5,79 | 2,98 | 0,0224 | 13,84 |
| 1 | ME7 | 0,301 | 10,15 | 7,16 | 6,62 | 2,15 | 0,0333 | 11,06 |
| 1 | ME7 | 0,344 | 10,83 | 5,62 | 6,69 | 3,89 | 0,0395 | 11,48 |
| 1 | ME7 | 0,320 | 11,40 | 7,42 | 7,37 | 2,49 | 0,0310 | 9,68 |
| 1 | ME8 | 0,419 | 12,77 | 6,97 | 6,22 | 1,27 | 0,0311 | 7,43 |
| 1 | ME8 | 0,303 | 8,54 | 6,31 | 5,67 | 2,09 | 0,0390 | 12,89 |
| 1 | ME8 | 0,440 | 10,17 | 7,73 | 6,12 | 2,16 | 0,0297 | 6,76 |
| 1 | ME8 | 0,433 | 11,36 | 6,67 | 6,79 | 3,25 | 0,0295 | 6,81 |
| 1 | ME8 | 0,489 | 11,28 | 6,62 | 4,86 | 2,52 | 0,0310 | 6,34 |
| 1 | ME8 | 0,389 | 9,12 | 7,26 | 7,05 | 2,56 | 0,0276 | 7,10 |
| 1 | ME8 | 0,371 | 9,36 | 6,82 | 5,49 | 2,58 | 0,0295 | 7,95 |
| 1 | ME8 | 0,419 | 10,36 | 7,38 | 6,60 | 2,47 | 0,0325 | 7,76 |
| 1 | ME8 | 0,314 | 9,57 | 6,50 | 6,58 | 1,71 | 0,0373 | 11,88 |
| 1 | ME8 | 0,410 | 10,08 | 7,20 | 6,47 | 2,68 | 0,0491 | 11,98 |
| 1 | ME8 | 0,419 | 10,95 | 5,92 | 6,77 | 2,71 | 0,0388 | 9,26 |
| 1 | ME8 | 0,363 | 10,51 | 6,03 | 7,10 | 2,14 | 0,0332 | 9,15 |
| 1 | ME8 | 0,359 | 9,42 | 5,49 | 6,19 | 1,50 | 0,0306 | 8,52 |
| 1 | ME8 | 0,466 | 10,21 | 5,38 | 5,62 | 1,63 | 0,0388 | 8,33 |
| 1 | ME8 | 0,381 | 9,24 | 8,37 | 7,02 | 2,92 | 0,0322 | 8,45 |
| 1 | ME8 | 0,431 | 11,80 | 6,45 | 7,49 | 1,42 | 0,0299 | 6,94 |
| 1 | ME8 | 0,287 | 8,80 | 5,49 | 5,39 | 1,23 | 0,0360 | 12,54 |
| 1 | ME8 | 0,305 | 9,80 | 4,08 | 6,93 | 1,74 | 0,0358 | 11,74 |
| 1 | ME8 | 0,371 | 10,41 | 6,85 | 6,39 | 1,28 | 0,0233 | 6,28 |
| 1 | ME8 | 0,418 | 10,08 | 5,45 | 5,52 | 2,01 | 0,0301 | 7,20 |
| 1 | ME8 | 0,443 | 11,28 | 4,99 | 7,08 | 1,61 | 0,0402 | 9,07 |
| 1 | ME8 | 0,446 | 11,58 | 8,02 | 5,65 | 1,35 | 0,0366 | 8,21 |
| 1 | ME8 | 0,412 | 10,92 | 7,28 | 4,83 | 1,52 | 0,0371 | 9,00 |
| 1 | ME8 | 0,415 | 10,66 | 8,17 | 4,56 | 2,21 | 0,0348 | 8,39 |
| 1 | ME8 | 0,429 | 12,40 | 7,08 | 6,91 | 2,57 | 0,0457 | 10,65 |
| 1 | ME9 | 0,423 | 8,71 | 5,73 | 7,59 | 3,29 | 0,0357 | 8,43 |
| 1 | ME9 | 0,405 | 10,79 | 6,72 | 6,56 | 3,58 | 0,0370 | 9,15 |
| 1 | ME9 | 0,383 | 10,59 | 6,91 | 6,96 | 3,26 | 0,0384 | 10,02 |
| 1 | ME9 | 0,326 | 10,95 | 5,61 | 7,76 | 2,29 | 0,0337 | 10,32 |
| 1 | ME9 | 0,408 | 9,67 | 6,56 | 7,21 | 3,60 | 0,0378 | 9,27 |
| 1 | ME9 | 0,458 | 12,09 | 8,44 | 5,91 | 2,49 | 0,0354 | 7,73 |
| 1 | ME9 | 0,423 | 11,45 | 7,38 | 7,87 | 2,50 | 0,0383 | 9,05 |
| 1 | ME9 | 0,459 | 10,61 | 7,99 | 7,78 | 2,54 | 0,0336 | 7,33 |
| 1 | ME9 | 0,424 | 9,79 | 7,95 | 5,70 | 2,73 | 0,0340 | 8,02 |
| 1 | ME9 | 0,447 | 10,33 | 8,04 | 6,10 | 3,43 | 0,0331 | 7,41 |
| 1 | ME9 | 0,369 | 11,39 | 7,09 | 5,47 | 3,82 | 0,0398 | 10,78 |
| 1 | ME9 | 0,398 | 11,14 | 7,30 | 6,89 | 3,47 | 0,0319 | 8,02 |
| 1 | ME9 | 0,422 | 11,61 | 7,20 | 5,59 | 4,54 | 0,0325 | 7,70 |
| 1 | ME9 | 0,430 | 10,45 | 7,45 | 6,88 | 2,93 | 0,0277 | 6,44 |
| 1 | ME9 | 0,302 | 10,11 | 7,27 | 7,67 | 2,99 | 0,0246 | 8,15 |
| 1 | ME9 | 0,299 | 8,89 | 5,98 | 6,17 | 3,29 | 0,0406 | 13,58 |
| 1 | ME9 | 0,421 | 10,56 | 7,52 | 8,25 | 2,79 | 0,0462 | 10,98 |
| 1 | ME9 | 0,368 | 10,51 | 7,82 | 7,61 | 2,97 | 0,0360 | 9,78 |
| 1 | ME9 | 0,488 | 11,34 | 7,59 | 7,69 | 2,41 | 0,0430 | 8,82 |
| 1 | ME9 | 0,444 | 10,78 | 8,90 | 5,63 | 2,71 | 0,0295 | 6,64 |
| 1 | ME9 | 0,364 | 9,62 | 8,04 | 6,92 | 2,43 | 0,0260 | 7,14 |
| 1 | ME9 | 0,439 | 10,34 | 7,45 | 7,76 | 2,89 | 0,0459 | 10,45 |
| 1 | ME9 | 0,477 | 11,79 | 7,23 | 7,44 | 2,92 | 0,0410 | 8,59 |
| 1 | ME9 | 0,342 | 9,63 | 5,29 | 7,82 | 2,45 | 0,0338 | 9,88 |
| 1 | ME9 | 0,406 | 10,30 | 8,74 | 7,19 | 2,96 | 0,0434 | 10,69 |
| 1 | ME10 | 0,395 | 9,61 | 6,36 | 5,79 | 2,34 | 0,0835 | 21,13 |
| 1 | ME10 | 0,498 | 11,57 | 7,66 | 6,55 | 2,61 | 0,0774 | 15,56 |
| 1 | ME10 | 0,480 | 10,22 | 5,22 | 7,05 | 1,68 | 0,0803 | 16,74 |
| 1 | ME10 | 0,487 | 9,36 | 7,29 | 6,14 | 2,31 | 0,0879 | 18,07 |
| 1 | ME10 | 0,391 | 9,99 | 4,63 | 6,39 | 1,79 | 0,0657 | 16,81 |
| 1 | ME10 | 0,415 | 10,20 | 6,53 | 4,02 | 2,14 | 0,0684 | 16,47 |
| 1 | ME10 | 0,444 | 11,78 | 5,88 | 6,89 | 2,86 | 0,0797 | 17,93 |
| 1 | ME10 | 0,461 | 10,31 | 9,42 | 4,86 | 2,21 | 0,0869 | 18,86 |
| 1 | ME10 | 0,504 | 9,96 | 9,61 | 5,92 | 2,26 | 0,0906 | 17,98 |
| 1 | ME10 | 0,454 | 11,44 | 8,13 | 6,98 | 2,06 | 0,0730 | 16,08 |
| 1 | ME10 | 0,469 | 11,57 | 7,28 | 6,45 | 2,06 | 0,0529 | 11,27 |
| 1 | ME10 | 0,393 | 10,92 | 8,31 | 5,65 | 2,58 | 0,0662 | 16,83 |
| 1 | ME10 | 0,509 | 11,43 | 9,20 | 7,02 | 1,98 | 0,0592 | 11,64 |
| 1 | ME10 | 0,491 | 10,06 | 6,89 | 5,92 | 1,92 | 0,0560 | 11,41 |
| 1 | ME10 | 0,417 | 10,04 | 6,89 | 6,76 | 2,60 | 0,0778 | 18,66 |
| 1 | ME10 | 0,351 | 9,69 | 4,14 | 5,71 | 2,38 | 0,0570 | 16,23 |
| 1 | ME10 | 0,409 | 11,79 | 6,92 | 7,82 | 2,51 | 0,0550 | 13,44 |
| 1 | ME10 | 0,419 | 10,42 | 6,52 | 6,06 | 1,84 | 0,0663 | 15,82 |
| 1 | ME10 | 0,389 | 10,76 | 6,83 | 7,20 | 2,23 | 0,0565 | 14,54 |
| 1 | ME10 | 0,576 | 12,57 | 8,68 | 6,55 | 2,36 | 0,0617 | 10,72 |
| 1 | ME10 | 0,413 | 11,21 | 7,15 | 5,49 | 2,36 | 0,0745 | 18,04 |
| 1 | ME10 | 0,510 | 11,72 | 7,30 | 6,73 | 2,21 | 0,0663 | 12,99 |
| 1 | ME10 | 0,490 | 12,01 | 7,40 | 7,30 | 2,74 | 0,0559 | 11,42 |
| 1 | ME10 | 0,306 | 7,90 | 4,65 | 5,93 | 2,56 | 0,0528 | 17,24 |
| 1 | ME10 | 0,526 | 10,73 | 8,49 | 6,26 | 2,83 | 0,0448 | 8,52 |
| 1 | ME11 | 0,541 | 11,26 | 6,62 | 6,71 | 3,07 | 0,0123 | 2,27 |
| 1 | ME11 | 0,373 | 10,38 | 6,26 | 6,24 | 2,92 | 0,0108 | 2,91 |
| 1 | ME11 | 0,451 | 11,14 | 5,76 | 6,68 | 1,81 | 0,0955 | 21,18 |
| 1 | ME11 | 0,435 | 10,50 | 4,61 | 6,31 | 1,60 | 0,0761 | 17,48 |
| 1 | ME11 | 0,360 | 7,03 | 6,32 | 5,71 | 1,80 | 0,0117 | 3,27 |
| 1 | ME11 | 0,463 | 10,46 | 7,96 | 6,70 | 2,24 | 0,0101 | 2,18 |
| 1 | ME11 | 0,347 | 9,54 | 4,24 | 4,13 | 2,19 | 0,0111 | 3,21 |
| 1 | ME11 | 0,432 | 11,48 | 7,96 | 6,89 | 1,75 | 0,0925 | 21,41 |
| 1 | ME11 | 0,421 | 11,04 | 5,70 | 6,90 | 2,94 | 0,0809 | 19,23 |
| 1 | ME11 | 0,419 | 10,88 | 3,26 | 6,56 | 1,89 | 0,0551 | 13,14 |
| 1 | ME11 | 0,347 | 8,28 | 6,00 | 5,85 | 2,86 | 0,0100 | 2,90 |
| 1 | ME11 | 0,359 | 8,42 | 5,43 | 6,75 | 2,32 | 0,0769 | 21,44 |
| 1 | ME11 | 0,528 | 9,16 | 7,39 | 5,98 | 2,27 | 0,0763 | 14,46 |
| 1 | ME11 | 0,499 | 11,53 | 5,93 | 7,08 | 2,93 | 0,0795 | 15,94 |
| 1 | ME11 | 0,374 | 10,91 | 6,09 | 6,75 | 2,47 | 0,0777 | 20,79 |
| 1 | ME11 | 0,428 | 11,53 | 7,22 | 7,63 | 2,40 | 0,0782 | 18,29 |
| 1 | ME11 | 0,314 | 9,69 | 5,01 | 6,57 | 1,66 | 0,0563 | 17,92 |
| 1 | ME11 | 0,482 | 10,56 | 7,07 | 7,10 | 2,55 | 0,0698 | 14,49 |
| 1 | ME11 | 0,536 | 10,66 | 8,66 | 6,02 | 2,20 | 0,0778 | 14,53 |
| 1 | ME11 | 0,434 | 10,94 | 4,33 | 6,26 | 2,45 | 0,0803 | 18,51 |
| 1 | ME11 | 0,451 | 9,70 | 6,93 | 4,95 | 2,95 | 0,0624 | 13,85 |
| 1 | ME11 | 0,423 | 9,38 | 6,90 | 4,07 | 2,51 | 0,0839 | 19,83 |
| 1 | ME11 | 0,502 | 10,69 | 5,87 | 6,24 | 2,58 | 0,0733 | 14,60 |
| 1 | ME11 | 0,440 | 10,49 | 5,94 | 6,06 | 2,24 | 0,0677 | 15,40 |
| 1 | ME11 | 0,343 | 9,62 | 3,19 | 6,15 | 1,96 | 0,0606 | 17,66 |
| 1 | ME12 | 0,368 | 10,28 | 6,87 | 5,29 | 0,70 | 0,0510 | 13,88 |
| 1 | ME12 | 0,425 | 9,85 | 6,29 | 5,96 | 2,06 | 0,0346 | 8,14 |
| 1 | ME12 | 0,383 | 10,15 | 7,26 | 6,66 | 2,20 | 0,0760 | 19,83 |
| 1 | ME12 | 0,376 | 11,37 | 5,20 | 5,50 | 1,63 | 0,0294 | 7,81 |
| 1 | ME12 | 0,486 | 11,29 | 5,52 | 6,06 | 1,30 | 0,0274 | 5,64 |
| 1 | ME12 | 0,427 | 10,13 | 7,75 | 5,98 | 1,42 | 0,0187 | 4,38 |
| 1 | ME12 | 0,420 | 11,20 | 7,13 | 6,34 | 2,50 | 0,0347 | 8,27 |
| 1 | ME12 | 0,443 | 11,22 | 6,95 | 6,51 | 2,96 | 0,0342 | 7,72 |
| 1 | ME12 | 0,439 | 11,19 | 6,84 | 7,29 | 2,38 | 0,0371 | 8,45 |
| 1 | ME12 | 0,406 | 11,22 | 4,86 | 5,67 | 2,15 | 0,0301 | 7,42 |
| 1 | ME12 | 0,311 | 9,57 | 7,50 | 7,46 | 1,96 | 0,0437 | 14,06 |
| 1 | ME12 | 0,510 | 11,07 | 6,82 | 4,75 | 1,80 | 0,0421 | 8,25 |
| 1 | ME12 | 0,357 | 11,33 | 6,03 | 5,82 | 2,58 | 0,0227 | 6,36 |
| 1 | ME12 | 0,300 | 9,48 | 4,18 | 5,56 | 1,87 | 0,0377 | 12,57 |
| 1 | ME12 | 0,429 | 10,36 | 5,76 | 6,54 | 2,54 | 0,0253 | 5,90 |
| 1 | ME12 | 0,234 | 8,28 | 3,70 | 6,51 | 2,65 | 0,0496 | 21,18 |
| 1 | ME12 | 0,307 | 9,04 | 3,84 | 6,72 | 1,68 | 0,0211 | 6,86 |
| 1 | ME12 | 0,226 | 7,69 | 4,25 | 6,56 | 1,88 | 0,0388 | 17,21 |
| 1 | ME12 | 0,323 | 8,21 | 5,02 | 6,09 | 1,02 | 0,0415 | 12,85 |
| 1 | ME12 | 0,382 | 9,05 | 5,10 | 6,48 | 2,09 | 0,0215 | 5,63 |
| 1 | ME12 | 0,426 | 9,90 | 5,50 | 4,99 | 1,69 | 0,0372 | 8,72 |
| 1 | ME12 | 0,405 | 11,18 | 6,50 | 6,01 | 2,08 | 0,0106 | 2,62 |
| 1 | ME12 | 0,396 | 10,67 | 6,40 | 5,95 | 1,93 | 0,0403 | 10,17 |
| 1 | ME12 | 0,415 | 9,62 | 6,50 | 7,16 | 1,17 | 0,0273 | 6,58 |
| 1 | ME12 | 0,447 | 9,23 | 7,65 | 7,17 | 1,03 | 0,0174 | 3,89 |
| 1 | ME13 | 0,419 | 11,34 | 6,63 | 6,67 | 2,40 | 0,0373 | 8,90 |
| 1 | ME13 | 0,380 | 10,13 | 7,48 | 7,60 | 2,03 | 0,0470 | 12,38 |
| 1 | ME13 | 0,510 | 11,64 | 6,98 | 8,47 | 1,92 | 0,0483 | 9,46 |
| 1 | ME13 | 0,429 | 11,48 | 6,80 | 7,40 | 3,10 | 0,0449 | 10,46 |
| 1 | ME13 | 0,445 | 10,24 | 6,72 | 6,80 | 2,37 | 0,0270 | 6,06 |
| 1 | ME13 | 0,242 | 11,95 | 6,39 | 7,40 | 1,52 | 0,0364 | 15,07 |
| 1 | ME13 | 0,469 | 9,61 | 3,97 | 5,58 | 2,07 | 0,0464 | 9,90 |
| 1 | ME13 | 0,437 | 10,88 | 7,78 | 5,40 | 1,24 | 0,0352 | 8,06 |
| 1 | ME13 | 0,371 | 10,23 | 6,67 | 7,28 | 3,25 | 0,0442 | 11,92 |
| 1 | ME13 | 0,349 | 8,81 | 6,22 | 7,23 | 1,86 | 0,0429 | 12,29 |
| 1 | ME13 | 0,441 | 10,07 | 5,66 | 6,04 | 2,38 | 0,0293 | 6,65 |
| 1 | ME13 | 0,426 | 11,40 | 6,26 | 7,07 | 1,93 | 0,0358 | 8,40 |
| 1 | ME13 | 0,410 | 11,85 | 7,06 | 6,89 | 2,60 | 0,0406 | 9,90 |
| 1 | ME13 | 0,312 | 10,39 | 6,88 | 5,70 | 2,01 | 0,0405 | 12,98 |
| 1 | ME13 | 0,258 | 9,95 | 6,20 | 6,20 | 1,83 | 0,0463 | 17,94 |
| 1 | ME13 | 0,370 | 10,79 | 6,35 | 6,10 | 3,03 | 0,0467 | 12,62 |
| 1 | ME13 | 0,400 | 9,24 | 7,45 | 5,68 | 1,32 | 0,0291 | 7,28 |
| 1 | ME13 | 0,389 | 11,05 | 6,52 | 6,33 | 1,72 | 0,0321 | 8,25 |
| 1 | ME13 | 0,454 | 10,54 | 5,90 | 7,73 | 1,72 | 0,0331 | 7,30 |
| 1 | ME13 | 0,253 | 11,34 | 6,94 | 5,95 | 2,55 | 0,0242 | 9,55 |
| 1 | ME13 | 0,423 | 9,97 | 5,16 | 7,96 | 1,36 | 0,0516 | 12,20 |
| 1 | ME13 | 0,397 | 9,50 | 6,49 | 6,81 | 3,15 | 0,0364 | 9,16 |
| 1 | ME13 | 0,406 | 10,73 | 6,07 | 5,63 | 2,27 | 0,0273 | 6,73 |
| 1 | ME13 | 0,406 | 10,34 | 7,01 | 7,13 | 2,38 | 0,0472 | 11,64 |
| 1 | ME13 | 0,425 | 11,58 | 6,37 | 6,99 | 2,26 | 0,0305 | 7,18 |
| 1 | ME14 | 0,424 | 12,10 | 7,84 | 6,55 | 1,77 | 0,0297 | 7,00 |
| 1 | ME14 | 0,462 | 12,45 | 7,05 | 6,70 | 1,91 | 0,0243 | 5,26 |
| 1 | ME14 | 0,427 | 10,99 | 7,74 | 6,29 | 2,03 | 0,0256 | 5,99 |
| 1 | ME14 | 0,303 | 9,67 | 6,44 | 4,92 | 2,00 | 0,0224 | 7,40 |
| 1 | ME14 | 0,372 | 10,69 | 7,62 | 6,04 | 2,00 | 0,0371 | 9,97 |
| 1 | ME14 | 0,370 | 11,23 | 6,92 | 7,03 | 1,77 | 0,0404 | 10,92 |
| 1 | ME14 | 0,480 | 12,52 | 8,71 | 5,74 | 1,12 | 0,0196 | 4,09 |
| 1 | ME14 | 0,469 | 11,93 | 7,78 | 5,49 | 1,60 | 0,0164 | 3,50 |
| 1 | ME14 | 0,474 | 11,68 | 8,05 | 6,16 | 2,00 | 0,0280 | 5,91 |
| 1 | ME14 | 0,487 | 11,17 | 7,33 | 5,21 | 1,49 | 0,0210 | 4,31 |
| 1 | ME14 | 0,465 | 10,59 | 7,99 | 5,95 | 1,21 | 0,0315 | 6,77 |
| 1 | ME14 | 0,487 | 12,52 | 8,57 | 7,67 | 1,61 | 0,0421 | 8,64 |
| 1 | ME14 | 0,428 | 9,47 | 9,24 | 4,60 | 2,52 | 0,0292 | 6,82 |
| 1 | ME14 | 0,278 | 10,11 | 6,15 | 5,90 | 1,29 | 0,0217 | 7,80 |
| 1 | ME14 | 0,421 | 12,58 | 7,43 | 6,82 | 1,39 | 0,0352 | 8,36 |
| 1 | ME14 | 0,417 | 10,87 | 8,81 | 6,34 | 1,82 | 0,0281 | 6,74 |
| 1 | ME14 | 0,459 | 12,19 | 7,61 | 6,28 | 1,78 | 0,0249 | 5,42 |
| 1 | ME14 | 0,403 | 10,95 | 6,15 | 6,54 | 1,65 | 0,0358 | 8,88 |
| 1 | ME14 | 0,506 | 9,39 | 6,51 | 6,59 | 2,65 | 0,0295 | 5,83 |
| 1 | ME14 | 0,246 | 12,02 | 8,36 | 5,20 | 2,19 | 0,0294 | 11,94 |
| 1 | ME14 | 0,453 | 11,41 | 9,13 | 6,33 | 2,36 | 0,0393 | 8,67 |
| 1 | ME14 | 0,321 | 8,48 | 7,04 | 6,19 | 1,57 | 0,0307 | 9,57 |
| 1 | ME14 | 0,505 | 9,63 | 7,72 | 7,15 | 1,46 | 0,0276 | 5,46 |
| 1 | ME14 | 0,405 | 12,54 | 7,69 | 6,92 | 2,14 | 0,0402 | 9,92 |
| 1 | ME14 | 0,486 | 11,14 | 6,31 | 4,37 | 1,11 | 0,0217 | 4,47 |
| 1 | ME16 | 0,454 | 11,48 | 7,43 | 6,56 | 2,35 | 0,0262 | 5,77 |
| 1 | ME16 | 0,415 | 13,17 | 7,59 | 6,78 | 2,11 | 0,0294 | 7,09 |
| 1 | ME16 | 0,442 | 14,24 | 7,73 | 7,06 | 2,91 | 0,0274 | 6,20 |
| 1 | ME16 | 0,312 | 8,90 | 7,07 | 7,95 | 3,20 | 0,0215 | 6,88 |
| 1 | ME16 | 0,334 | 10,44 | 6,44 | 6,75 | 2,54 | 0,0311 | 9,33 |
| 1 | ME16 | 0,433 | 11,26 | 8,14 | 7,04 | 2,15 | 0,0274 | 6,32 |
| 1 | ME16 | 0,489 | 11,45 | 9,43 | 6,50 | 2,14 | 0,0247 | 5,05 |
| 1 | ME16 | 0,243 | 9,92 | 5,23 | 6,48 | 2,16 | 0,0247 | 10,16 |
| 1 | ME16 | 0,435 | 10,94 | 8,61 | 5,47 | 3,18 | 0,0198 | 4,55 |
| 1 | ME16 | 0,425 | 11,27 | 8,40 | 6,40 | 2,96 | 0,0220 | 5,18 |
| 1 | ME16 | 0,471 | 11,83 | 6,74 | 6,73 | 2,41 | 0,0214 | 4,54 |
| 1 | ME16 | 0,341 | 10,05 | 6,13 | 6,25 | 2,13 | 0,0230 | 6,74 |
| 1 | ME16 | 0,335 | 11,72 | 7,20 | 6,01 | 2,85 | 0,0213 | 6,36 |
| 1 | ME16 | 0,465 | 11,94 | 8,98 | 6,48 | 2,30 | 0,0181 | 3,89 |
| 1 | ME16 | 0,451 | 10,27 | 7,55 | 6,55 | 2,82 | 0,0183 | 4,06 |
| 1 | ME16 | 0,366 | 10,40 | 6,16 | 6,95 | 3,18 | 0,0194 | 5,30 |
| 1 | ME16 | 0,334 | 10,11 | 8,22 | 7,21 | 2,32 | 0,0208 | 6,23 |
| 1 | ME16 | 0,254 | 9,60 | 5,84 | 6,30 | 2,27 | 0,0137 | 5,39 |
| 1 | ME16 | 0,352 | 9,86 | 7,75 | 6,87 | 2,31 | 0,0215 | 6,11 |
| 1 | ME16 | 0,383 | 11,38 | 5,83 | 7,10 | 2,65 | 0,0223 | 5,82 |
| 1 | ME16 | 0,352 | 9,62 | 7,85 | 5,83 | 2,63 | 0,0291 | 8,27 |
| 1 | ME16 | 0,423 | 10,49 | 7,78 | 5,46 | 3,25 | 0,0177 | 4,18 |
| 1 | ME16 | 0,391 | 10,66 | 7,96 | 6,31 | 2,35 | 0,0207 | 5,29 |
| 1 | ME16 | 0,415 | 10,23 | 7,62 | 6,77 | 2,39 | 0,0223 | 5,37 |
| 1 | ME16 | 0,331 | 10,87 | 6,05 | 6,18 | 2,10 | 0,0202 | 6,10 |
| 1 | ME17 | 0,378 | 10,23 | 6,86 | 6,86 | 1,82 | 0,0381 | 10,08 |
| 1 | ME17 | 0,425 | 10,67 | 6,34 | 5,65 | 2,24 | 0,0316 | 7,43 |
| 1 | ME17 | 0,424 | 11,28 | 6,92 | 4,82 | 1,24 | 0,0279 | 6,58 |
| 1 | ME17 | 0,314 | 10,31 | 6,26 | 5,76 | 1,61 | 0,0352 | 11,23 |
| 1 | ME17 | 0,299 | 9,44 | 6,02 | 6,06 | 2,96 | 0,0254 | 8,50 |
| 1 | ME17 | 0,357 | 10,35 | 5,55 | 6,99 | 2,03 | 0,0293 | 8,22 |
| 1 | ME17 | 0,398 | 11,43 | 5,80 | 7,42 | 2,62 | 0,0315 | 7,91 |
| 1 | ME17 | 0,453 | 10,33 | 8,16 | 5,84 | 2,18 | 0,0299 | 6,61 |
| 1 | ME17 | 0,439 | 12,64 | 6,21 | 8,08 | 2,08 | 0,0328 | 7,47 |
| 1 | ME17 | 0,408 | 9,74 | 6,99 | 4,58 | 2,40 | 0,0234 | 5,74 |
| 1 | ME17 | 0,434 | 12,37 | 6,79 | 7,11 | 2,37 | 0,0351 | 8,09 |
| 1 | ME17 | 0,354 | 9,12 | 7,10 | 3,79 | 2,52 | 0,0260 | 7,36 |
| 1 | ME17 | 0,297 | 9,19 | 5,77 | 6,20 | 3,09 | 0,0274 | 9,23 |
| 1 | ME17 | 0,444 | 11,10 | 5,28 | 5,35 | 2,67 | 0,0395 | 8,91 |
| 1 | ME17 | 0,425 | 11,18 | 6,26 | 8,02 | 2,93 | 0,0575 | 13,52 |
| 1 | ME17 | 0,384 | 10,17 | 5,61 | 7,52 | 2,22 | 0,0653 | 17,02 |
| 1 | ME17 | 0,280 | 9,75 | 5,65 | 6,96 | 2,72 | 0,0357 | 12,74 |
| 1 | ME17 | 0,390 | 10,76 | 6,50 | 6,83 | 1,78 | 0,0256 | 6,57 |
| 1 | ME17 | 0,410 | 9,80 | 6,41 | 5,50 | 2,34 | 0,0299 | 7,30 |
| 1 | ME17 | 0,385 | 10,69 | 6,58 | 5,51 | 1,85 | 0,0264 | 6,87 |
| 1 | ME17 | 0,443 | 12,17 | 7,48 | 6,62 | 1,90 | 0,0453 | 10,22 |
| 1 | ME17 | 0,428 | 10,35 | 6,23 | 6,67 | 2,33 | 0,0360 | 8,41 |
| 1 | ME17 | 0,456 | 10,86 | 6,40 | 6,91 | 2,92 | 0,0331 | 7,27 |
| 1 | ME17 | 0,395 | 10,41 | 8,20 | 5,90 | 2,06 | 0,0279 | 7,07 |
| 1 | ME17 | 0,532 | 11,30 | 7,10 | 6,76 | 3,01 | 0,0338 | 6,35 |
| 1 | ME18 | 0,474 | 10,95 | 8,44 | 5,71 | 2,32 | 0,0364 | 7,69 |
| 1 | ME18 | 0,280 | 10,18 | 5,68 | 6,41 | 2,38 | 0,0343 | 12,26 |
| 1 | ME18 | 0,360 | 9,32 | 6,92 | 6,67 | 2,97 | 0,0483 | 13,44 |
| 1 | ME18 | 0,258 | 10,55 | 5,97 | 7,23 | 2,59 | 0,0385 | 14,93 |
| 1 | ME18 | 0,303 | 10,23 | 7,22 | 7,14 | 3,06 | 0,0431 | 14,21 |
| 1 | ME18 | 0,174 | 11,66 | 5,01 | 7,24 | 2,97 | 0,0333 | 19,13 |
| 1 | ME18 | 0,500 | 10,41 | 8,48 | 6,80 | 3,40 | 0,0337 | 6,74 |
| 1 | ME18 | 0,289 | 7,49 | 7,84 | 5,14 | 2,47 | 0,0376 | 13,02 |
| 1 | ME18 | 0,194 | 8,62 | 5,63 | 5,90 | 3,41 | 0,0220 | 11,33 |
| 1 | ME18 | 0,412 | 10,82 | 7,43 | 5,87 | 2,87 | 0,0233 | 5,65 |
| 1 | ME18 | 0,407 | 10,32 | 9,62 | 5,56 | 3,20 | 0,0226 | 5,55 |
| 1 | ME18 | 0,368 | 10,78 | 7,54 | 5,85 | 2,55 | 0,0450 | 12,23 |
| 1 | ME18 | 0,385 | 9,91 | 7,62 | 6,56 | 1,86 | 0,0180 | 4,68 |
| 1 | ME18 | 0,176 | 8,16 | 3,26 | 6,15 | 2,80 | 0,0228 | 12,98 |
| 1 | ME18 | 0,364 | 10,29 | 7,61 | 6,84 | 2,31 | 0,0216 | 5,93 |
| 1 | ME18 | 0,359 | 10,79 | 8,19 | 7,06 | 2,00 | 0,0370 | 10,30 |
| 1 | ME18 | 0,352 | 10,04 | 5,73 | 5,58 | 2,50 | 0,0313 | 8,90 |
| 1 | ME18 | 0,249 | 9,15 | 7,04 | 5,22 | 3,61 | 0,0277 | 11,15 |
| 1 | ME18 | 0,175 | 7,62 | 4,41 | 6,21 | 2,35 | 0,0301 | 17,25 |
| 1 | ME18 | 0,164 | 9,81 | 3,31 | 6,67 | 2,50 | 0,0255 | 15,59 |
| 1 | ME18 | 0,208 | 8,94 | 6,27 | 6,57 | 2,99 | 0,0348 | 16,70 |
| 1 | ME18 | 0,196 | 10,13 | 5,02 | 7,18 | 2,89 | 0,0388 | 19,85 |
| 1 | ME18 | 0,354 | 12,16 | 7,40 | 7,75 | 2,14 | 0,0316 | 8,93 |
| 1 | ME18 | 0,389 | 11,43 | 8,21 | 7,57 | 2,75 | 0,0289 | 7,42 |
| 1 | ME18 | 0,279 | 10,27 | 5,25 | 5,96 | 2,31 | 0,0254 | 9,11 |
| 1 | ME19 | 0,421 | 9,98 | 7,77 | 6,48 | 0,97 | 0,0226 | 5,37 |
| 1 | ME19 | 0,416 | 10,67 | 7,48 | 6,95 | 1,82 | 0,0403 | 9,69 |
| 1 | ME19 | 0,481 | 10,87 | 8,07 | 6,73 | 1,41 | 0,0319 | 6,64 |
| 1 | ME19 | 0,258 | 11,19 | 7,15 | 7,02 | 1,99 | 0,0179 | 6,95 |
| 1 | ME19 | 0,330 | 11,65 | 5,71 | 7,16 | 1,12 | 0,0239 | 7,24 |
| 1 | ME19 | 0,152 | 8,37 | 4,34 | 5,91 | 0,66 | 0,0154 | 10,10 |
| 1 | ME19 | 0,274 | 9,72 | 7,14 | 6,85 | 2,54 | 0,0269 | 9,82 |
| 1 | ME19 | 0,448 | 11,57 | 8,48 | 6,78 | 3,44 | 0,0270 | 6,03 |
| 1 | ME19 | 0,386 | 8,89 | 6,77 | 7,04 | 2,17 | 0,0437 | 11,32 |
| 1 | ME19 | 0,368 | 11,92 | 6,67 | 8,63 | 1,30 | 0,0286 | 7,78 |
| 1 | ME19 | 0,438 | 11,41 | 9,21 | 6,84 | 2,01 | 0,0299 | 6,83 |
| 1 | ME19 | 0,339 | 9,44 | 7,29 | 5,72 | 1,53 | 0,0269 | 7,94 |
| 1 | ME19 | 0,302 | 9,83 | 6,88 | 4,68 | 1,21 | 0,0217 | 7,19 |
| 1 | ME19 | 0,450 | 12,07 | 8,19 | 7,25 | 2,08 | 0,0225 | 5,00 |
| 1 | ME19 | 0,265 | 10,44 | 5,41 | 7,14 | 2,05 | 0,0268 | 10,13 |
| 1 | ME19 | 0,178 | 7,64 | 4,73 | 5,60 | 2,28 | 0,0339 | 19,04 |
| 1 | ME19 | 0,415 | 10,60 | 7,33 | 6,65 | 1,98 | 0,0288 | 6,94 |
| 1 | ME19 | 0,406 | 11,64 | 6,73 | 7,26 | 1,93 | 0,0523 | 12,90 |
| 1 | ME19 | 0,399 | 10,82 | 8,05 | 7,67 | 1,31 | 0,0344 | 8,63 |
| 1 | ME19 | 0,318 | 10,15 | 6,42 | 7,32 | 1,65 | 0,0289 | 9,10 |
| 1 | ME19 | 0,336 | 10,73 | 7,09 | 5,80 | 2,06 | 0,0295 | 8,79 |
| 1 | ME19 | 0,250 | 8,13 | 5,99 | 7,19 | 2,63 | 0,0218 | 8,72 |
| 1 | ME19 | 0,276 | 8,21 | 7,24 | 5,67 | 1,55 | 0,0273 | 9,88 |
| 1 | ME19 | 0,259 | 8,10 | 5,97 | 6,17 | 1,45 | 0,0210 | 8,11 |
| 1 | ME19 | 0,366 | 9,56 | 6,87 | 5,84 | 1,53 | 0,0179 | 4,90 |
| 1 | ME20 | 0,462 | 12,27 | 8,56 | 7,89 | 2,82 | 0,0413 | 8,94 |
| 1 | ME20 | 0,403 | 10,71 | 6,59 | 6,69 | 4,48 | 0,0469 | 11,64 |
| 1 | ME20 | 0,385 | 12,44 | 5,66 | 8,11 | 2,03 | 0,0410 | 10,65 |
| 1 | ME20 | 0,322 | 10,50 | 7,16 | 7,29 | 3,06 | 0,0384 | 11,93 |
| 1 | ME20 | 0,354 | 9,98 | 8,37 | 6,23 | 3,38 | 0,0314 | 8,87 |
| 1 | ME20 | 0,501 | 12,02 | 9,22 | 6,27 | 3,18 | 0,0364 | 7,27 |
| 1 | ME20 | 0,397 | 10,73 | 7,58 | 5,21 | 2,75 | 0,0315 | 7,93 |
| 1 | ME20 | 0,376 | 10,19 | 8,52 | 7,57 | 2,43 | 0,0409 | 10,87 |
| 1 | ME20 | 0,439 | 9,50 | 9,69 | 6,75 | 3,43 | 0,0393 | 8,96 |
| 1 | ME20 | 0,449 | 8,18 | 9,12 | 7,86 | 2,46 | 0,0441 | 9,83 |
| 1 | ME20 | 0,243 | 9,07 | 6,93 | 5,66 | 2,41 | 0,0197 | 8,10 |
| 1 | ME20 | 0,176 | 9,48 | 4,36 | 6,92 | 2,10 | 0,0195 | 11,08 |
| 1 | ME20 | 0,306 | 10,34 | 5,95 | 7,42 | 2,14 | 0,0335 | 10,95 |
| 1 | ME20 | 0,388 | 9,98 | 7,12 | 5,75 | 2,26 | 0,0241 | 6,21 |
| 1 | ME20 | 0,448 | 11,16 | 8,14 | 6,85 | 2,48 | 0,0420 | 9,39 |
| 1 | ME20 | 0,317 | 9,94 | 6,25 | 7,01 | 3,27 | 0,0374 | 11,81 |
| 1 | ME20 | 0,363 | 11,46 | 7,82 | 6,42 | 2,44 | 0,0290 | 7,99 |
| 1 | ME20 | 0,467 | 12,17 | 7,71 | 6,92 | 2,11 | 0,0317 | 6,79 |
| 1 | ME20 | 0,158 | 9,88 | 4,84 | 6,16 | 2,50 | 0,0236 | 14,92 |
| 1 | ME20 | 0,251 | 9,58 | 5,21 | 5,24 | 1,65 | 0,0274 | 10,92 |
| 1 | ME20 | 0,197 | 9,62 | 8,69 | 7,22 | 2,90 | 0,0357 | 18,15 |
| 1 | ME20 | 0,257 | 11,90 | 6,63 | 5,91 | 3,19 | 0,0304 | 11,85 |
| 1 | ME20 | 0,335 | 10,63 | 7,66 | 6,80 | 3,49 | 0,0344 | 10,27 |
| 1 | ME20 | 0,346 | 11,39 | 5,56 | 6,42 | 2,62 | 0,0284 | 8,22 |
| 1 | ME20 | 0,493 | 12,63 | 8,34 | 7,19 | 3,09 | 0,0407 | 8,26 |
| 1 | ME21 | 0,362 | 10,78 | 8,22 | 6,94 | 3,41 | 0,0305 | 8,43 |
| 1 | ME21 | 0,347 | 11,95 | 8,84 | 7,09 | 3,90 | 0,0273 | 7,86 |
| 1 | ME21 | 0,338 | 10,39 | 6,93 | 6,59 | 3,55 | 0,0474 | 14,01 |
| 1 | ME21 | 0,374 | 10,79 | 8,33 | 8,14 | 2,56 | 0,0320 | 8,55 |
| 1 | ME21 | 0,348 | 11,59 | 8,17 | 7,50 | 2,39 | 0,0315 | 9,05 |
| 1 | ME21 | 0,369 | 11,59 | 7,29 | 7,63 | 3,17 | 0,0288 | 7,80 |
| 1 | ME21 | 0,390 | 11,61 | 7,59 | 6,39 | 2,14 | 0,0250 | 6,41 |
| 1 | ME21 | 0,476 | 12,06 | 8,94 | 7,63 | 2,50 | 0,0407 | 8,55 |
| 1 | ME21 | 0,445 | 10,94 | 10,20 | 6,64 | 3,66 | 0,0346 | 7,78 |
| 1 | ME21 | 0,352 | 10,02 | 8,21 | 6,32 | 3,51 | 0,0366 | 10,41 |
| 1 | ME21 | 0,268 | 10,51 | 5,45 | 7,93 | 2,25 | 0,0268 | 9,99 |
| 1 | ME21 | 0,331 | 9,15 | 8,93 | 6,72 | 3,01 | 0,0296 | 8,95 |
| 1 | ME21 | 0,421 | 10,24 | 9,45 | 6,18 | 2,45 | 0,0277 | 6,59 |
| 1 | ME21 | 0,334 | 10,13 | 6,78 | 7,03 | 3,23 | 0,0280 | 8,40 |
| 1 | ME21 | 0,462 | 11,63 | 8,00 | 7,01 | 3,27 | 0,0291 | 6,30 |
| 1 | ME21 | 0,348 | 10,08 | 9,14 | 5,67 | 3,45 | 0,0245 | 7,05 |
| 1 | ME21 | 0,454 | 11,58 | 7,07 | 7,57 | 2,52 | 0,0314 | 6,91 |
| 1 | ME21 | 0,441 | 10,89 | 7,55 | 7,22 | 2,32 | 0,0319 | 7,24 |
| 1 | ME21 | 0,344 | 9,39 | 8,11 | 5,68 | 3,24 | 0,0219 | 6,37 |
| 1 | ME21 | 0,288 | 9,12 | 6,11 | 6,49 | 2,42 | 0,0251 | 8,73 |
| 1 | ME21 | 0,365 | 10,77 | 8,85 | 7,24 | 2,80 | 0,0147 | 4,03 |
| 1 | ME21 | 0,424 | 11,18 | 7,68 | 6,00 | 2,94 | 0,0250 | 5,90 |
| 1 | ME21 | 0,244 | 9,38 | 6,67 | 6,65 | 2,85 | 0,0220 | 9,02 |
| 1 | ME21 | 0,356 | 11,05 | 7,70 | 6,16 | 3,06 | 0,0275 | 7,73 |
| 1 | ME21 | 0,209 | 9,12 | 5,91 | 6,29 | 2,28 | 0,0233 | 11,15 |
| 1 | MA1 | 0,364 | 9,73 | 7,16 | 7,34 | 4,25 | 0,0673 | 18,48 |
| 1 | MA1 | 0,496 | 9,51 | 9,06 | 7,15 | 3,44 | 0,0380 | 7,66 |
| 1 | MA1 | 0,319 | 9,37 | 7,57 | 7,82 | 3,46 | 0,0473 | 14,83 |
| 1 | MA1 | 0,467 | 11,23 | 6,78 | 6,55 | 3,29 | 0,0580 | 12,42 |
| 1 | MA1 | 0,292 | 10,56 | 5,35 | 7,50 | 2,69 | 0,0428 | 14,66 |
| 1 | MA1 | 0,370 | 9,03 | 7,42 | 6,30 | 2,27 | 0,0302 | 8,16 |
| 1 | MA1 | 0,429 | 10,55 | 8,59 | 5,73 | 2,61 | 0,0419 | 9,77 |
| 1 | MA1 | 0,439 | 8,32 | 6,49 | 6,92 | 2,48 | 0,0329 | 7,49 |
| 1 | MA1 | 0,456 | 10,82 | 8,51 | 7,05 | 3,57 | 0,0367 | 8,05 |
| 1 | MA1 | 0,536 | 11,66 | 7,69 | 8,18 | 2,22 | 0,0389 | 7,26 |
| 1 | MA1 | 0,411 | 11,23 | 8,30 | 7,11 | 2,81 | 0,0311 | 7,57 |
| 1 | MA1 | 0,475 | 9,80 | 6,83 | 6,87 | 2,08 | 0,0510 | 10,74 |
| 1 | MA1 | 0,373 | 8,51 | 6,14 | 5,78 | 2,92 | 0,0408 | 10,94 |
| 1 | MA1 | 0,506 | 8,43 | 6,61 | 6,31 | 3,01 | 0,0521 | 10,30 |
| 1 | MA1 | 0,405 | 10,40 | 7,25 | 6,26 | 3,36 | 0,0440 | 10,86 |
| 1 | MA1 | 0,435 | 9,31 | 6,16 | 5,52 | 1,92 | 0,0232 | 5,33 |
| 1 | MA1 | 0,375 | 9,25 | 6,48 | 6,48 | 3,02 | 0,0360 | 9,60 |
| 1 | MA1 | 0,393 | 10,15 | 6,78 | 7,21 | 1,88 | 0,0354 | 9,01 |
| 1 | MA1 | 0,456 | 11,11 | 8,58 | 7,27 | 2,88 | 0,0332 | 7,28 |
| 1 | MA1 | 0,470 | 10,42 | 7,65 | 5,40 | 1,58 | 0,0334 | 7,10 |
| 1 | MA1 | 0,356 | 9,52 | 5,18 | 5,99 | 2,96 | 0,0416 | 11,68 |
| 1 | MA1 | 0,300 | 12,54 | 8,98 | 6,31 | 3,50 | 0,0377 | 12,57 |
| 1 | MA1 | 0,495 | 9,12 | 6,54 | 6,25 | 2,69 | 0,0446 | 9,00 |
| 1 | MA1 | 0,392 | 9,64 | 6,12 | 6,35 | 3,72 | 0,0467 | 11,90 |
| 1 | MA1 | 0,383 | 9,13 | 6,86 | 6,57 | 3,33 | 0,0465 | 12,13 |
| 1 | MA2 | 0,396 | 10,56 | 5,97 | 6,80 | 1,50 | 0,0216 | 5,45 |
| 1 | MA2 | 0,345 | 9,98 | 4,99 | 6,01 | 1,27 | 0,0228 | 6,61 |
| 1 | MA2 | 0,437 | 11,16 | 6,14 | 6,99 | 1,98 | 0,0336 | 7,69 |
| 1 | MA2 | 0,456 | 9,65 | 6,22 | 6,53 | 1,60 | 0,0333 | 7,30 |
| 1 | MA2 | 0,403 | 10,50 | 6,67 | 7,01 | 1,80 | 0,0330 | 8,18 |
| 1 | MA2 | 0,402 | 10,17 | 5,41 | 6,09 | 1,97 | 0,0313 | 7,79 |
| 1 | MA2 | 0,325 | 9,68 | 4,45 | 6,46 | 2,08 | 0,0377 | 11,61 |
| 1 | MA2 | 0,465 | 11,56 | 6,63 | 6,52 | 1,52 | 0,0233 | 5,01 |
| 1 | MA2 | 0,383 | 9,72 | 5,53 | 7,13 | 2,08 | 0,0412 | 10,76 |
| 1 | MA2 | 0,468 | 12,26 | 4,85 | 7,17 | 1,29 | 0,0322 | 6,89 |
| 1 | MA2 | 0,344 | 7,65 | 5,80 | 6,13 | 2,13 | 0,0297 | 8,63 |
| 1 | MA2 | 0,481 | 11,50 | 6,11 | 7,24 | 2,10 | 0,0261 | 5,43 |
| 1 | MA2 | 0,351 | 10,43 | 4,92 | 6,62 | 1,89 | 0,0346 | 9,85 |
| 1 | MA2 | 0,411 | 11,38 | 4,96 | 7,46 | 2,25 | 0,0283 | 6,88 |
| 1 | MA2 | 0,399 | 11,08 | 6,50 | 7,18 | 1,77 | 0,0274 | 6,87 |
| 1 | MA2 | 0,427 | 10,39 | 5,54 | 6,14 | 1,67 | 0,0275 | 6,45 |
| 1 | MA2 | 0,477 | 12,84 | 5,18 | 8,01 | 1,40 | 0,0328 | 6,87 |
| 1 | MA2 | 0,394 | 10,10 | 5,34 | 5,33 | 2,13 | 0,0360 | 9,13 |
| 1 | MA2 | 0,359 | 10,68 | 6,48 | 5,84 | 2,19 | 0,0305 | 8,50 |
| 1 | MA2 | 0,427 | 9,48 | 6,86 | 7,65 | 1,95 | 0,0458 | 10,74 |
| 1 | MA2 | 0,472 | 12,81 | 4,82 | 8,10 | 1,81 | 0,0444 | 9,41 |
| 1 | MA2 | 0,382 | 9,63 | 6,31 | 6,07 | 1,58 | 0,0265 | 6,93 |
| 1 | MA2 | 0,504 | 11,37 | 6,67 | 8,35 | 2,73 | 0,0475 | 9,43 |
| 1 | MA2 | 0,480 | 11,04 | 6,94 | 6,72 | 1,70 | 0,0290 | 6,05 |
| 1 | MA2 | 0,556 | 12,86 | 6,54 | 6,99 | 1,74 | 0,0352 | 6,33 |
| 1 | MA3 | 0,335 | 9,41 | 7,69 | 6,96 | 2,54 | 0,0325 | 9,70 |
| 1 | MA3 | 0,346 | 8,36 | 6,83 | 6,89 | 2,26 | 0,0321 | 9,28 |
| 1 | MA3 | 0,448 | 10,96 | 8,36 | 6,83 | 2,75 | 0,0362 | 8,08 |
| 1 | MA3 | 0,233 | 9,71 | 5,52 | 6,20 | 1,97 | 0,0361 | 15,49 |
| 1 | MA3 | 0,308 | 9,48 | 6,04 | 6,59 | 2,83 | 0,0213 | 6,92 |
| 1 | MA3 | 0,386 | 9,13 | 7,24 | 5,83 | 2,67 | 0,0219 | 5,67 |
| 1 | MA3 | 0,332 | 9,15 | 6,65 | 6,84 | 2,74 | 0,0367 | 11,05 |
| 1 | MA3 | 0,222 | 8,60 | 7,08 | 5,50 | 3,32 | 0,0253 | 11,40 |
| 1 | MA3 | 0,408 | 10,08 | 7,31 | 6,87 | 2,65 | 0,0303 | 7,43 |
| 1 | MA3 | 0,409 | 10,33 | 6,68 | 6,92 | 2,98 | 0,0432 | 10,56 |
| 1 | MA3 | 0,387 | 9,20 | 8,60 | 5,98 | 2,98 | 0,0266 | 6,87 |
| 1 | MA3 | 0,365 | 9,22 | 7,14 | 6,11 | 2,67 | 0,0239 | 6,55 |
| 1 | MA3 | 0,455 | 9,92 | 8,61 | 6,52 | 3,75 | 0,0389 | 8,55 |
| 1 | MA3 | 0,294 | 8,68 | 7,08 | 6,09 | 2,97 | 0,0303 | 10,31 |
| 1 | MA3 | 0,264 | 9,12 | 5,75 | 6,78 | 2,69 | 0,0382 | 14,47 |
| 1 | MA3 | 0,442 | 10,49 | 6,66 | 6,67 | 2,59 | 0,0463 | 10,48 |
| 1 | MA3 | 0,179 | 7,46 | 5,13 | 6,98 | 3,22 | 0,0311 | 17,37 |
| 1 | MA3 | 0,394 | 11,01 | 8,23 | 7,00 | 2,45 | 0,0268 | 6,80 |
| 1 | MA3 | 0,408 | 10,86 | 8,38 | 6,70 | 2,83 | 0,0512 | 12,55 |
| 1 | MA3 | 0,376 | 9,93 | 7,08 | 6,38 | 3,06 | 0,0329 | 8,75 |
| 1 | MA3 | 0,380 | 9,69 | 7,54 | 6,30 | 3,24 | 0,0422 | 11,11 |
| 1 | MA3 | 0,370 | 9,18 | 8,47 | 6,61 | 3,76 | 0,0526 | 14,22 |
| 1 | MA3 | 0,378 | 8,08 | 7,50 | 7,46 | 2,74 | 0,0543 | 14,37 |
| 1 | MA3 | 0,329 | 9,57 | 7,10 | 6,67 | 3,35 | 0,0314 | 9,54 |
| 1 | MA3 | 0,435 | 10,85 | 7,65 | 7,26 | 2,76 | 0,0293 | 6,74 |
| 1 | MA4 | 0,372 | 11,17 | 6,63 | 7,58 | 2,70 | 0,0293 | 7,88 |
| 1 | MA4 | 0,361 | 11,74 | 6,33 | 8,28 | 2,48 | 0,0315 | 8,73 |
| 1 | MA4 | 0,278 | 9,62 | 6,28 | 6,40 | 3,19 | 0,0355 | 12,77 |
| 1 | MA4 | 0,376 | 11,24 | 6,71 | 6,68 | 2,90 | 0,0425 | 11,30 |
| 1 | MA4 | 0,387 | 10,27 | 8,19 | 6,54 | 3,03 | 0,0257 | 6,64 |
| 1 | MA4 | 0,375 | 11,23 | 7,48 | 7,09 | 2,77 | 0,0300 | 8,00 |
| 1 | MA4 | 0,381 | 10,37 | 8,02 | 7,26 | 2,92 | 0,0367 | 9,63 |
| 1 | MA4 | 0,374 | 10,97 | 6,16 | 7,09 | 2,12 | 0,0360 | 9,63 |
| 1 | MA4 | 0,446 | 11,69 | 6,43 | 7,21 | 2,48 | 0,0432 | 9,69 |
| 1 | MA4 | 0,228 | 8,67 | 6,38 | 5,91 | 3,06 | 0,0467 | 20,48 |
| 1 | MA4 | 0,452 | 11,63 | 7,50 | 8,37 | 2,35 | 0,0486 | 10,75 |
| 1 | MA4 | 0,403 | 11,00 | 6,25 | 7,47 | 2,74 | 0,0369 | 9,16 |
| 1 | MA4 | 0,492 | 11,23 | 9,03 | 7,28 | 3,38 | 0,0451 | 9,17 |
| 1 | MA4 | 0,326 | 10,11 | 6,34 | 7,38 | 2,80 | 0,0337 | 10,34 |
| 1 | MA4 | 0,471 | 10,62 | 8,66 | 6,33 | 2,43 | 0,0411 | 8,73 |
| 1 | MA4 | 0,469 | 11,53 | 7,97 | 6,96 | 3,87 | 0,0442 | 9,42 |
| 1 | MA4 | 0,301 | 10,48 | 5,25 | 7,52 | 2,83 | 0,0396 | 13,16 |
| 1 | MA4 | 0,190 | 8,23 | 5,01 | 6,01 | 3,27 | 0,0215 | 11,32 |
| 1 | MA4 | 0,383 | 11,66 | 7,20 | 7,27 | 3,22 | 0,0387 | 10,10 |
| 1 | MA4 | 0,356 | 9,87 | 7,63 | 5,71 | 2,82 | 0,0348 | 9,78 |
| 1 | MA4 | 0,413 | 10,76 | 6,37 | 6,51 | 2,46 | 0,0327 | 7,92 |
| 1 | MA4 | 0,377 | 9,60 | 7,00 | 7,30 | 2,80 | 0,0325 | 8,62 |
| 1 | MA4 | 0,334 | 10,04 | 6,13 | 6,71 | 2,30 | 0,0374 | 11,20 |
| 1 | MA4 | 0,323 | 9,60 | 7,22 | 7,22 | 2,57 | 0,0420 | 13,00 |
| 1 | MA4 | 0,456 | 11,04 | 8,87 | 6,95 | 2,24 | 0,0281 | 6,16 |
| 1 | MA5 | 0,292 | 9,04 | 5,47 | 6,09 | 2,77 | 0,0217 | 7,44 |
| 1 | MA5 | 0,288 | 10,72 | 6,21 | 6,90 | 2,68 | 0,0255 | 8,85 |
| 1 | MA5 | 0,457 | 10,11 | 6,95 | 8,04 | 3,24 | 0,0283 | 6,19 |
| 1 | MA5 | 0,434 | 10,08 | 7,71 | 6,99 | 2,65 | 0,0245 | 5,65 |
| 1 | MA5 | 0,230 | 10,95 | 7,76 | 6,11 | 2,87 | 0,0190 | 8,25 |
| 1 | MA5 | 0,310 | 9,82 | 3,53 | 7,26 | 3,26 | 0,0257 | 8,28 |
| 1 | MA5 | 0,383 | 11,32 | 7,32 | 7,30 | 2,79 | 0,0248 | 6,47 |
| 1 | MA5 | 0,381 | 10,34 | 7,40 | 7,22 | 2,80 | 0,0309 | 8,12 |
| 1 | MA5 | 0,403 | 9,60 | 7,40 | 7,86 | 1,39 | 0,0218 | 5,41 |
| 1 | MA5 | 0,301 | 10,79 | 5,34 | 6,77 | 3,12 | 0,0289 | 9,60 |
| 1 | MA5 | 0,441 | 12,19 | 7,62 | 7,49 | 1,82 | 0,0248 | 5,63 |
| 1 | MA5 | 0,383 | 10,63 | 7,96 | 6,07 | 2,09 | 0,0144 | 3,76 |
| 1 | MA5 | 0,477 | 12,42 | 8,48 | 6,52 | 2,64 | 0,0239 | 5,01 |
| 1 | MA5 | 0,369 | 10,52 | 7,26 | 6,68 | 3,02 | 0,0285 | 7,72 |
| 1 | MA5 | 0,483 | 11,77 | 9,84 | 6,74 | 2,07 | 0,0272 | 5,63 |
| 1 | MA5 | 0,455 | 11,94 | 7,60 | 7,10 | 3,32 | 0,0355 | 7,80 |
| 1 | MA5 | 0,406 | 11,83 | 8,70 | 8,02 | 1,97 | 0,0245 | 6,03 |
| 1 | MA5 | 0,320 | 12,40 | 7,01 | 7,13 | 2,20 | 0,0224 | 7,00 |
| 1 | MA5 | 0,454 | 11,31 | 9,49 | 7,79 | 3,40 | 0,0243 | 5,36 |
| 1 | MA5 | 0,347 | 9,75 | 7,39 | 5,54 | 2,35 | 0,0251 | 7,23 |
| 1 | MA5 | 0,361 | 10,42 | 7,81 | 7,37 | 2,76 | 0,0272 | 7,54 |
| 1 | MA5 | 0,380 | 10,73 | 8,05 | 6,89 | 2,43 | 0,0231 | 6,09 |
| 1 | MA5 | 0,430 | 11,12 | 8,70 | 6,51 | 3,48 | 0,0235 | 5,46 |
| 1 | MA5 | 0,375 | 11,32 | 8,08 | 7,58 | 2,82 | 0,0255 | 6,80 |
| 1 | MA5 | 0,302 | 10,02 | 6,06 | 6,71 | 1,43 | 0,0220 | 7,28 |
| 1 | BA1 | 0,343 | 10,56 | 7,31 | 6,33 | 2,51 | 0,0456 | 13,28 |
| 1 | BA1 | 0,347 | 10,64 | 6,58 | 5,98 | 2,72 | 0,0284 | 8,18 |
| 1 | BA1 | 0,280 | 9,48 | 6,03 | 6,91 | 2,91 | 0,0495 | 17,71 |
| 1 | BA1 | 0,344 | 10,23 | 7,93 | 7,46 | 3,15 | 0,0548 | 15,94 |
| 1 | BA1 | 0,310 | 11,64 | 6,21 | 7,37 | 2,64 | 0,0547 | 17,63 |
| 1 | BA1 | 0,309 | 10,42 | 7,16 | 6,98 | 2,95 | 0,0466 | 15,10 |
| 1 | BA1 | 0,327 | 9,54 | 8,01 | 6,21 | 2,61 | 0,0399 | 12,21 |
| 1 | BA1 | 0,359 | 10,70 | 7,89 | 7,22 | 2,95 | 0,0514 | 14,32 |
| 1 | BA1 | 0,328 | 9,79 | 7,24 | 7,46 | 2,56 | 0,0491 | 14,98 |
| 1 | BA1 | 0,259 | 9,52 | 7,92 | 7,29 | 2,23 | 0,0371 | 14,34 |
| 1 | BA1 | 0,321 | 9,09 | 6,33 | 7,40 | 2,75 | 0,0406 | 12,63 |
| 1 | BA1 | 0,212 | 10,02 | 6,01 | 6,74 | 2,58 | 0,0401 | 18,94 |
| 1 | BA1 | 0,253 | 10,39 | 7,03 | 7,21 | 3,16 | 0,0526 | 20,82 |
| 1 | BA1 | 0,262 | 11,40 | 6,62 | 6,46 | 2,50 | 0,0426 | 16,25 |
| 1 | BA1 | 0,351 | 10,47 | 6,37 | 7,69 | 2,70 | 0,0574 | 16,38 |
| 1 | BA1 | 0,377 | 11,11 | 8,59 | 6,64 | 2,57 | 0,0485 | 12,86 |
| 1 | BA1 | 0,283 | 9,31 | 7,14 | 6,76 | 2,71 | 0,0501 | 17,68 |
| 1 | BA1 | 0,329 | 10,88 | 8,01 | 6,92 | 2,90 | 0,0472 | 14,35 |
| 1 | BA1 | 0,350 | 11,26 | 8,20 | 6,95 | 2,42 | 0,0533 | 15,22 |
| 1 | BA1 | 0,350 | 10,29 | 7,17 | 7,77 | 2,39 | 0,0428 | 12,22 |
| 1 | BA1 | 0,314 | 10,77 | 7,38 | 7,02 | 2,87 | 0,0463 | 14,73 |
| 1 | BA1 | 0,251 | 9,97 | 6,15 | 6,63 | 2,76 | 0,0452 | 17,99 |
| 1 | BA1 | 0,314 | 9,68 | 6,35 | 7,56 | 2,52 | 0,0366 | 11,66 |
| 1 | BA1 | 0,357 | 9,82 | 7,83 | 6,89 | 2,47 | 0,0550 | 15,39 |
| 1 | BA1 | 0,342 | 10,25 | 8,33 | 6,51 | 2,87 | 0,0539 | 15,77 |
| 1 | VA1 | 0,467 | 10,58 | 6,67 | 5,83 | 1,92 | 0,0297 | 6,36 |
| 1 | VA1 | 0,419 | 12,82 | 6,86 | 8,24 | 1,82 | 0,0173 | 4,13 |
| 1 | VA1 | 0,446 | 11,14 | 6,99 | 6,73 | 2,50 | 0,0301 | 6,75 |
| 1 | VA1 | 0,416 | 10,51 | 6,27 | 7,16 | 1,62 | 0,0311 | 7,48 |
| 1 | VA1 | 0,432 | 10,60 | 6,89 | 6,18 | 2,24 | 0,0259 | 6,00 |
| 1 | VA1 | 0,420 | 12,82 | 7,96 | 6,82 | 2,48 | 0,0227 | 5,41 |
| 1 | VA1 | 0,359 | 10,05 | 6,29 | 6,39 | 1,98 | 0,0246 | 6,85 |
| 1 | VA1 | 0,380 | 9,33 | 6,26 | 5,90 | 2,27 | 0,0301 | 7,92 |
| 1 | VA1 | 0,415 | 10,30 | 5,70 | 6,50 | 1,80 | 0,0236 | 5,69 |
| 1 | VA1 | 0,416 | 10,87 | 6,92 | 6,44 | 1,96 | 0,0217 | 5,21 |
| 1 | VA1 | 0,332 | 9,71 | 5,18 | 6,19 | 2,44 | 0,0254 | 7,66 |
| 1 | VA1 | 0,416 | 9,80 | 6,58 | 5,44 | 2,37 | 0,0286 | 6,87 |
| 1 | VA1 | 0,487 | 12,02 | 7,04 | 7,38 | 1,35 | 0,0302 | 6,20 |
| 1 | VA1 | 0,408 | 12,19 | 6,68 | 6,46 | 1,89 | 0,0305 | 7,47 |
| 1 | VA1 | 0,432 | 11,41 | 6,20 | 7,25 | 1,65 | 0,0295 | 6,83 |
| 1 | VA1 | 0,385 | 9,73 | 7,44 | 6,11 | 2,30 | 0,0316 | 8,20 |
| 1 | VA1 | 0,466 | 11,67 | 6,55 | 7,15 | 1,74 | 0,0389 | 8,35 |
| 1 | VA1 | 0,370 | 10,70 | 7,72 | 7,13 | 1,96 | 0,0134 | 3,62 |
| 1 | VA1 | 0,411 | 9,55 | 7,07 | 6,80 | 1,82 | 0,0339 | 8,24 |
| 1 | VA1 | 0,479 | 11,54 | 6,97 | 6,96 | 1,94 | 0,0290 | 6,06 |
| 1 | VA1 | 0,272 | 9,21 | 6,06 | 7,58 | 2,40 | 0,0246 | 9,04 |
| 1 | VA1 | 0,388 | 9,28 | 7,19 | 5,99 | 2,16 | 0,0210 | 5,41 |
| 1 | VA1 | 0,382 | 10,96 | 7,02 | 6,78 | 2,32 | 0,0299 | 7,82 |
| 1 | VA1 | 0,282 | 10,10 | 6,99 | 4,53 | 1,06 | 0,0159 | 5,63 |
| 1 | VA1 | 0,480 | 12,61 | 7,83 | 6,10 | 2,23 | 0,0290 | 6,04 |
| 1 | MC1 | 0,233 | 8,78 | 4,75 | 6,85 | 2,16 | 0,0242 | 10,38 |
| 1 | MC1 | 0,412 | 11,12 | 7,56 | 6,77 | 2,28 | 0,0394 | 9,57 |
| 1 | MC1 | 0,325 | 10,68 | 6,24 | 6,64 | 2,90 | 0,0385 | 11,85 |
| 1 | MC1 | 0,346 | 10,99 | 8,09 | 6,82 | 2,77 | 0,0265 | 7,67 |
| 1 | MC1 | 0,460 | 10,87 | 8,20 | 6,33 | 2,88 | 0,0279 | 6,07 |
| 1 | MC1 | 0,448 | 11,62 | 7,94 | 6,96 | 2,41 | 0,0253 | 5,65 |
| 1 | MC1 | 0,347 | 11,40 | 9,03 | 7,51 | 1,97 | 0,0243 | 7,01 |
| 1 | MC1 | 0,250 | 8,44 | 6,95 | 4,59 | 2,08 | 0,0152 | 6,08 |
| 1 | MC1 | 0,393 | 11,25 | 8,24 | 6,82 | 2,37 | 0,0239 | 6,08 |
| 1 | MC1 | 0,410 | 11,63 | 7,76 | 6,24 | 2,77 | 0,0311 | 7,59 |
| 1 | MC1 | 0,447 | 11,57 | 7,36 | 6,62 | 2,64 | 0,0302 | 6,75 |
| 1 | MC1 | 0,462 | 10,16 | 9,40 | 6,95 | 3,02 | 0,0260 | 5,63 |
| 1 | MC1 | 0,360 | 10,98 | 6,62 | 3,60 | 2,47 | 0,0108 | 3,00 |
| 1 | MC1 | 0,341 | 10,55 | 4,96 | 6,98 | 2,42 | 0,0279 | 8,19 |
| 1 | MC1 | 0,480 | 12,40 | 6,50 | 7,90 | 2,40 | 0,0395 | 8,23 |
| 1 | MC1 | 0,420 | 11,41 | 7,61 | 7,22 | 2,95 | 0,0359 | 8,55 |
| 1 | MC1 | 0,397 | 10,72 | 7,10 | 6,29 | 2,79 | 0,0306 | 7,72 |
| 1 | MC1 | 0,263 | 10,14 | 6,37 | 6,86 | 2,69 | 0,0294 | 11,17 |
| 1 | MC1 | 0,427 | 10,46 | 9,59 | 6,52 | 3,14 | 0,0280 | 6,57 |
| 1 | MC1 | 0,356 | 10,71 | 6,30 | 6,64 | 2,32 | 0,0184 | 5,17 |
| 1 | MC1 | 0,486 | 12,08 | 8,42 | 7,74 | 2,19 | 0,0264 | 5,44 |
| 1 | MC1 | 0,405 | 11,71 | 7,27 | 6,64 | 2,74 | 0,0319 | 7,88 |
| 1 | MC1 | 0,273 | 9,80 | 4,90 | 7,72 | 1,96 | 0,0327 | 11,98 |
| 1 | MC1 | 0,253 | 10,00 | 6,86 | 7,14 | 2,54 | 0,0219 | 8,67 |
| 1 | MC1 | 0,436 | 12,44 | 7,01 | 7,51 | 2,29 | 0,0259 | 5,94 |
| 1 | MO2 | 0,502 | 13,26 | 7,91 | 7,79 | 2,97 | 0,0303 | 6,04 |
| 1 | MO2 | 0,445 | 12,31 | 9,60 | 7,09 | 2,68 | 0,0293 | 6,58 |
| 1 | MO2 | 0,487 | 12,88 | 9,45 | 8,29 | 2,86 | 0,0348 | 7,15 |
| 1 | MO2 | 0,297 | 12,24 | 5,40 | 7,65 | 2,50 | 0,0259 | 8,72 |
| 1 | MO2 | 0,364 | 11,73 | 8,54 | 7,49 | 3,02 | 0,0249 | 6,84 |
| 1 | MO2 | 0,444 | 11,67 | 9,73 | 7,31 | 2,51 | 0,0434 | 9,77 |
| 1 | MO2 | 0,390 | 13,06 | 9,49 | 8,30 | 2,40 | 0,0256 | 6,56 |
| 1 | MO2 | 0,423 | 12,42 | 8,61 | 7,60 | 3,23 | 0,0333 | 7,87 |
| 1 | MO2 | 0,310 | 12,26 | 8,49 | 8,59 | 3,05 | 0,0365 | 11,77 |
| 1 | MO2 | 0,343 | 12,77 | 8,42 | 8,08 | 2,75 | 0,0237 | 6,91 |
| 1 | MO2 | 0,427 | 12,23 | 7,95 | 8,21 | 3,06 | 0,0370 | 8,67 |
| 1 | MO2 | 0,416 | 12,80 | 9,98 | 7,41 | 2,49 | 0,0265 | 6,37 |
| 1 | MO2 | 0,423 | 11,28 | 9,42 | 7,56 | 2,04 | 0,0231 | 5,46 |
| 1 | MO2 | 0,473 | 12,58 | 9,45 | 9,04 | 4,11 | 0,0436 | 9,22 |
| 1 | MO2 | 0,373 | 11,12 | 8,48 | 7,80 | 3,56 | 0,0368 | 9,87 |
| 1 | MO2 | 0,449 | 12,16 | 10,79 | 7,58 | 2,14 | 0,0394 | 8,78 |
| 1 | MO2 | 0,444 | 12,61 | 10,37 | 7,84 | 3,52 | 0,0400 | 9,01 |
| 1 | MO2 | 0,473 | 12,45 | 9,13 | 8,03 | 3,72 | 0,0385 | 8,14 |
| 1 | MO2 | 0,380 | 11,04 | 8,67 | 6,76 | 2,21 | 0,0210 | 5,53 |
| 1 | MO2 | 0,320 | 10,74 | 8,24 | 6,82 | 2,62 | 0,0231 | 7,22 |
| 1 | MO2 | 0,409 | 12,18 | 8,91 | 7,76 | 2,87 | 0,0320 | 7,82 |
| 1 | MO3 | 0,390 | 11,46 | 7,39 | 6,86 | 2,31 | 0,0277 | 7,10 |
| 1 | MO3 | 0,389 | 10,71 | 6,12 | 6,41 | 2,26 | 0,0275 | 7,07 |
| 1 | MO3 | 0,431 | 12,27 | 5,89 | 8,29 | 2,41 | 0,0377 | 8,75 |
| 1 | MO3 | 0,505 | 11,38 | 7,24 | 5,02 | 1,91 | 0,0283 | 5,60 |
| 1 | MO3 | 0,419 | 13,81 | 6,45 | 8,23 | 2,32 | 0,0425 | 10,14 |
| 1 | MO3 | 0,447 | 12,34 | 7,08 | 5,50 | 1,29 | 0,0355 | 7,94 |
| 1 | MO3 | 0,481 | 10,01 | 7,19 | 5,93 | 2,80 | 0,0385 | 8,00 |
| 1 | MO3 | 0,281 | 13,09 | 6,85 | 7,29 | 2,82 | 0,0442 | 15,73 |
| 1 | MO3 | 0,410 | 8,96 | 5,57 | 6,80 | 2,76 | 0,0252 | 6,15 |
| 1 | MO3 | 0,537 | 11,06 | 6,49 | 6,72 | 2,47 | 0,0400 | 7,45 |
| 1 | MO3 | 0,499 | 11,93 | 7,41 | 7,46 | 3,07 | 0,0468 | 9,38 |
| 1 | MO3 | 0,404 | 10,90 | 7,14 | 7,27 | 3,48 | 0,0481 | 11,91 |
| 1 | MO3 | 0,464 | 11,27 | 7,38 | 8,32 | 2,66 | 0,0458 | 9,87 |
| 1 | MO3 | 0,431 | 11,31 | 7,74 | 6,45 | 2,41 | 0,0436 | 10,12 |
| 1 | MO3 | 0,365 | 11,52 | 6,22 | 7,81 | 3,15 | 0,0345 | 9,45 |
| 1 | MO3 | 0,177 | 11,07 | 5,75 | 7,76 | 2,90 | 0,0215 | 12,15 |
| 1 | MO3 | 0,439 | 8,48 | 3,57 | 6,84 | 2,03 | 0,0431 | 9,82 |
| 1 | MO3 | 0,341 | 9,52 | 7,94 | 7,64 | 2,02 | 0,0402 | 11,79 |
| 1 | MO3 | 0,320 | 10,96 | 6,89 | 7,56 | 1,88 | 0,0425 | 13,28 |
| 1 | MO3 | 0,210 | 8,88 | 5,28 | 7,28 | 1,88 | 0,0385 | 18,33 |
| 1 | MO3 | 0,324 | 10,62 | 5,02 | 5,47 | 2,46 | 0,0306 | 9,44 |
| 1 | MO3 | 0,402 | 10,22 | 6,20 | 6,73 | 2,70 | 0,0264 | 6,56 |
| 1 | MO3 | 0,287 | 10,76 | 5,66 | 6,34 | 2,10 | 0,0420 | 14,62 |
| 1 | MO3 | 0,179 | 10,07 | 5,75 | 7,78 | 2,16 | 0,0342 | 19,11 |
| 1 | MO3 | 0,405 | 9,05 | 3,47 | 6,71 | 2,15 | 0,0334 | 8,25 |
| 1 | MO4 | 0,352 | 10,86 | 7,09 | 8,60 | 2,12 | 0,0353 | 10,03 |
| 1 | MO4 | 0,380 | 10,80 | 6,51 | 7,59 | 2,44 | 0,0263 | 6,92 |
| 1 | MO4 | 0,444 | 11,67 | 7,41 | 7,14 | 2,04 | 0,0228 | 5,14 |
| 1 | MO4 | 0,262 | 10,08 | 7,03 | 6,54 | 1,59 | 0,0163 | 6,22 |
| 1 | MO4 | 0,413 | 10,96 | 7,19 | 7,04 | 2,97 | 0,0214 | 5,18 |
| 1 | MO4 | 0,365 | 9,93 | 6,77 | 6,80 | 2,94 | 0,0270 | 7,40 |
| 1 | MO4 | 0,408 | 11,05 | 7,54 | 7,44 | 3,03 | 0,0259 | 6,35 |
| 1 | MO4 | 0,517 | 12,57 | 9,37 | 7,04 | 2,45 | 0,0252 | 4,87 |
| 1 | MO4 | 0,411 | 12,74 | 8,19 | 6,77 | 2,83 | 0,0237 | 5,77 |
| 1 | MO4 | 0,262 | 11,07 | 7,36 | 7,53 | 2,39 | 0,0292 | 11,15 |
| 1 | MO4 | 0,389 | 11,50 | 7,74 | 7,06 | 1,88 | 0,0273 | 7,02 |
| 1 | MO4 | 0,261 | 10,03 | 6,48 | 6,62 | 2,72 | 0,0247 | 9,46 |
| 1 | MO4 | 0,391 | 12,46 | 6,52 | 6,86 | 2,79 | 0,0258 | 6,60 |
| 1 | MO4 | 0,372 | 11,97 | 7,29 | 6,87 | 2,34 | 0,0230 | 6,18 |
| 1 | MO4 | 0,380 | 11,45 | 8,80 | 6,76 | 2,91 | 0,0179 | 4,71 |
| 1 | MO4 | 0,430 | 12,50 | 9,64 | 6,01 | 3,38 | 0,0289 | 6,72 |
| 1 | MO4 | 0,330 | 10,89 | 7,80 | 6,52 | 2,67 | 0,0257 | 7,79 |
| 1 | MO4 | 0,404 | 12,14 | 6,27 | 7,63 | 2,20 | 0,0260 | 6,44 |
| 1 | MO4 | 0,361 | 11,27 | 6,86 | 7,32 | 2,62 | 0,0275 | 7,62 |
| 1 | MO4 | 0,473 | 11,70 | 7,64 | 7,02 | 2,89 | 0,0237 | 5,01 |
| 1 | MO4 | 0,302 | 11,86 | 6,91 | 7,47 | 2,78 | 0,0252 | 8,34 |
| 1 | MO4 | 0,469 | 11,96 | 7,32 | 7,54 | 2,67 | 0,0260 | 5,55 |
| 1 | MO4 | 0,390 | 10,88 | 7,53 | 6,43 | 3,33 | 0,0275 | 7,06 |
| 1 | MO4 | 0,332 | 10,52 | 7,88 | 6,71 | 2,71 | 0,0267 | 8,03 |
| 1 | MO4 | 0,254 | 11,28 | 7,26 | 6,78 | 2,92 | 0,0238 | 9,36 |
| 1 | MO5 | 0,463 | 10,72 | 3,02 | 7,33 | 2,27 | 0,0569 | 12,29 |
| 1 | MO5 | 0,455 | 11,98 | 6,73 | 6,77 | 2,29 | 0,0658 | 14,46 |
| 1 | MO5 | 0,316 | 11,15 | 5,58 | 7,84 | 1,93 | 0,0635 | 20,09 |
| 1 | MO5 | 0,385 | 9,76 | 7,85 | 4,90 | 2,02 | 0,0574 | 14,91 |
| 1 | MO5 | 0,349 | 9,73 | 5,40 | 6,58 | 1,36 | 0,0701 | 20,09 |
| 1 | MO5 | 0,438 | 11,64 | 7,38 | 7,44 | 2,71 | 0,0773 | 17,65 |
| 1 | MO5 | 0,442 | 11,53 | 7,11 | 7,98 | 2,43 | 0,0757 | 17,13 |
| 1 | MO5 | 0,407 | 11,00 | 6,36 | 7,39 | 2,70 | 0,0598 | 14,69 |
| 1 | MO5 | 0,414 | 10,81 | 7,28 | 8,39 | 2,68 | 0,0574 | 13,86 |
| 1 | MO5 | 0,419 | 10,45 | 7,19 | 7,23 | 1,37 | 0,0897 | 21,41 |
| 1 | MO5 | 0,202 | 9,38 | 4,06 | 7,60 | 1,62 | 0,0362 | 17,92 |
| 1 | MO5 | 0,301 | 8,04 | 5,71 | 7,32 | 2,19 | 0,0476 | 15,81 |
| 1 | MO5 | 0,467 | 13,00 | 7,78 | 8,71 | 2,28 | 0,0737 | 15,78 |
| 1 | MO5 | 0,528 | 11,03 | 7,79 | 6,64 | 2,61 | 0,0677 | 12,82 |
| 1 | MO5 | 0,421 | 10,02 | 6,70 | 6,48 | 2,39 | 0,0482 | 11,45 |
| 1 | MO5 | 0,500 | 11,42 | 7,13 | 7,52 | 2,56 | 0,0435 | 8,70 |
| 1 | MO5 | 0,457 | 11,04 | 7,59 | 7,86 | 2,52 | 0,0670 | 14,66 |
| 1 | MO5 | 0,443 | 8,87 | 7,62 | 6,44 | 2,55 | 0,0541 | 12,21 |
| 1 | MO5 | 0,437 | 9,71 | 5,92 | 5,48 | 2,11 | 0,0392 | 8,97 |
| 1 | MO5 | 0,358 | 9,80 | 6,85 | 4,68 | 2,76 | 0,0579 | 16,17 |
| 1 | MO5 | 0,357 | 9,57 | 6,58 | 6,04 | 2,02 | 0,0663 | 18,58 |
| 1 | MO5 | 0,388 | 9,62 | 8,14 | 6,88 | 2,87 | 0,0740 | 19,08 |
| 1 | MO5 | 0,289 | 8,57 | 5,61 | 6,86 | 2,86 | 0,0480 | 16,61 |
| 1 | MO5 | 0,493 | 10,28 | 6,70 | 6,49 | 2,70 | 0,0699 | 14,18 |
| 1 | MO5 | 0,511 | 11,17 | 8,45 | 6,15 | 2,44 | 0,0470 | 9,20 |
| 1 | MO6 | 0,382 | 9,77 | 5,38 | 6,49 | 2,11 | 0,0213 | 5,57 |
| 1 | MO6 | 0,319 | 9,36 | 5,03 | 6,69 | 2,50 | 0,0530 | 16,61 |
| 1 | MO6 | 0,349 | 11,50 | 4,87 | 7,19 | 2,04 | 0,0591 | 16,93 |
| 1 | MO6 | 0,351 | 9,39 | 6,42 | 6,93 | 2,02 | 0,0697 | 19,86 |
| 1 | MO6 | 0,402 | 10,35 | 6,32 | 7,05 | 2,81 | 0,0705 | 17,54 |
| 1 | MO6 | 0,452 | 12,04 | 6,48 | 5,71 | 3,23 | 0,0741 | 16,39 |
| 1 | MO6 | 0,439 | 8,92 | 5,93 | 7,13 | 2,43 | 0,0800 | 18,23 |
| 1 | MO6 | 0,459 | 10,30 | 6,26 | 5,93 | 2,18 | 0,0847 | 18,45 |
| 1 | MO6 | 0,533 | 10,51 | 7,91 | 5,67 | 3,11 | 0,0835 | 15,67 |
| 1 | MO6 | 0,308 | 9,60 | 4,81 | 6,28 | 2,07 | 0,0566 | 18,38 |
| 1 | MO6 | 0,373 | 8,14 | 3,52 | 6,27 | 2,12 | 0,0800 | 21,45 |
| 1 | MO6 | 0,514 | 11,77 | 6,89 | 5,80 | 2,07 | 0,0914 | 17,78 |
| 1 | MO6 | 0,397 | 10,88 | 5,81 | 6,40 | 1,94 | 0,0731 | 18,41 |
| 1 | MO6 | 0,397 | 9,98 | 7,54 | 6,24 | 1,60 | 0,0786 | 19,80 |
| 1 | MO6 | 0,389 | 9,09 | 4,91 | 6,24 | 1,93 | 0,0582 | 14,96 |
| 1 | MO6 | 0,408 | 11,84 | 6,13 | 7,90 | 1,51 | 0,0725 | 17,77 |
| 1 | MO6 | 0,412 | 11,89 | 5,65 | 7,57 | 2,27 | 0,0839 | 20,36 |
| 1 | MO6 | 0,407 | 11,09 | 6,56 | 8,83 | 2,64 | 0,0836 | 20,54 |
| 1 | MO6 | 0,516 | 12,70 | 6,60 | 7,84 | 2,53 | 0,0614 | 11,90 |
| 1 | MO6 | 0,456 | 11,31 | 6,23 | 7,62 | 2,01 | 0,0813 | 17,83 |
| 1 | MO6 | 0,461 | 11,80 | 5,97 | 7,96 | 2,45 | 0,0768 | 16,67 |
| 1 | MO6 | 0,449 | 11,33 | 5,63 | 6,83 | 2,17 | 0,0782 | 17,40 |
| 1 | MO6 | 0,473 | 11,47 | 7,32 | 6,84 | 2,27 | 0,0670 | 14,17 |
| 1 | MO6 | 0,485 | 9,49 | 6,57 | 7,99 | 2,54 | 0,0766 | 15,79 |
| 1 | MO6 | 0,503 | 8,45 | 6,25 | 5,59 | 2,13 | 0,0547 | 10,88 |
| 1 | AB1 | 0,293 | 8,98 | 6,40 | 5,78 | 1,85 | 0,0405 | 13,80 |
| 1 | AB1 | 0,236 | 8,44 | 4,52 | 7,62 | 2,50 | 0,0264 | 11,19 |
| 1 | AB1 | 0,429 | 10,14 | 8,06 | 7,94 | 2,36 | 0,0693 | 16,15 |
| 1 | AB1 | 0,317 | 9,73 | 5,55 | 5,77 | 2,94 | 0,0427 | 13,45 |
| 1 | AB1 | 0,469 | 11,51 | 6,77 | 7,27 | 2,37 | 0,0740 | 15,78 |
| 1 | AB1 | 0,309 | 11,04 | 5,72 | 9,02 | 2,32 | 0,0405 | 13,12 |
| 1 | AB1 | 0,451 | 11,49 | 7,40 | 9,03 | 2,99 | 0,0759 | 16,82 |
| 1 | AB1 | 0,466 | 11,53 | 8,15 | 7,20 | 2,44 | 0,0682 | 14,64 |
| 1 | AB1 | 0,550 | 11,72 | 7,16 | 7,80 | 2,50 | 0,0723 | 13,14 |
| 1 | AB1 | 0,326 | 10,88 | 3,88 | 7,10 | 2,35 | 0,0505 | 15,50 |
| 1 | AB1 | 0,424 | 10,85 | 7,21 | 6,23 | 2,85 | 0,0647 | 15,26 |
| 1 | AB1 | 0,261 | 9,98 | 6,41 | 6,80 | 2,28 | 0,0443 | 16,99 |
| 1 | AB1 | 0,537 | 11,45 | 7,01 | 8,24 | 3,20 | 0,0708 | 13,20 |
| 1 | AB1 | 0,477 | 11,45 | 5,92 | 7,25 | 2,63 | 0,0474 | 9,95 |
| 1 | AB1 | 0,435 | 10,42 | 6,74 | 7,51 | 2,14 | 0,0607 | 13,97 |
| 1 | AB1 | 0,445 | 10,20 | 7,83 | 6,84 | 2,88 | 0,0509 | 11,43 |
| 1 | AB1 | 0,477 | 10,50 | 8,77 | 8,02 | 3,20 | 0,0646 | 13,53 |
| 1 | AB1 | 0,459 | 10,49 | 5,81 | 6,87 | 2,63 | 0,0708 | 15,42 |
| 1 | AB1 | 0,347 | 9,43 | 6,28 | 7,81 | 3,26 | 0,0503 | 14,51 |
| 1 | AB1 | 0,442 | 10,16 | 5,19 | 6,88 | 3,08 | 0,0756 | 17,12 |
| 1 | AB1 | 0,444 | 10,60 | 7,86 | 6,54 | 3,15 | 0,0505 | 11,37 |
| 1 | AB1 | 0,393 | 10,80 | 5,25 | 8,07 | 2,80 | 0,0707 | 18,00 |
| 1 | AB1 | 0,419 | 9,43 | 7,03 | 6,88 | 3,05 | 0,0677 | 16,18 |
| 1 | AB1 | 0,418 | 10,31 | 5,98 | 6,94 | 3,86 | 0,0444 | 10,63 |
| 1 | AB1 | 0,367 | 10,92 | 5,71 | 7,24 | 2,55 | 0,0568 | 15,48 |
| 1 | PB1 | 0,310 | 10,35 | 6,51 | 7,48 | 3,14 | 0,0365 | 11,76 |
| 1 | PB1 | 0,229 | 10,64 | 5,97 | 8,26 | 3,29 | 0,0328 | 14,32 |
| 1 | PB1 | 0,305 | 9,97 | 6,12 | 7,70 | 3,19 | 0,0364 | 11,92 |
| 1 | PB1 | 0,326 | 10,70 | 6,63 | 7,57 | 3,06 | 0,0349 | 10,71 |
| 1 | PB1 | 0,316 | 10,74 | 8,10 | 7,68 | 3,35 | 0,0358 | 11,33 |
| 1 | PB1 | 0,263 | 10,58 | 8,68 | 7,65 | 2,92 | 0,0345 | 13,10 |
| 1 | PB1 | 0,281 | 10,61 | 6,47 | 7,65 | 2,85 | 0,0312 | 11,10 |
| 1 | PB1 | 0,272 | 10,93 | 7,81 | 7,60 | 3,45 | 0,0368 | 13,51 |
| 1 | PB1 | 0,271 | 10,31 | 8,02 | 7,43 | 3,24 | 0,0383 | 14,15 |
| 1 | PB1 | 0,170 | 8,47 | 4,84 | 6,84 | 3,93 | 0,0190 | 11,20 |
| 1 | PB1 | 0,263 | 10,69 | 6,42 | 7,75 | 3,44 | 0,0410 | 15,60 |
| 1 | PB1 | 0,348 | 10,64 | 7,35 | 7,62 | 3,26 | 0,0452 | 12,98 |
| 1 | PB1 | 0,170 | 8,87 | 4,54 | 8,01 | 3,07 | 0,0304 | 17,91 |
| 1 | PB1 | 0,348 | 10,59 | 8,10 | 7,29 | 3,12 | 0,0362 | 10,41 |
| 1 | PB1 | 0,314 | 9,25 | 6,46 | 7,30 | 3,15 | 0,0370 | 11,79 |
| 1 | PB1 | 0,355 | 9,82 | 6,02 | 7,34 | 3,72 | 0,0376 | 10,60 |
| 1 | PB1 | 0,267 | 9,08 | 7,87 | 7,51 | 3,62 | 0,0462 | 17,32 |
| 1 | PB1 | 0,341 | 9,43 | 6,28 | 7,32 | 3,41 | 0,0411 | 12,06 |
| 1 | PB1 | 0,320 | 10,33 | 5,83 | 7,44 | 3,49 | 0,0380 | 11,86 |
| 1 | PB1 | 0,316 | 10,57 | 4,98 | 7,70 | 3,39 | 0,0353 | 11,19 |
| 1 | PB1 | 0,318 | 10,18 | 6,81 | 7,55 | 3,56 | 0,0349 | 10,97 |
| 1 | PB1 | 0,307 | 10,42 | 7,48 | 8,53 | 3,65 | 0,0322 | 10,50 |
| 1 | PB1 | 0,324 | 9,05 | 6,00 | 7,11 | 3,52 | 0,0379 | 11,68 |
| 1 | PB1 | 0,173 | 8,08 | 4,89 | 7,79 | 3,22 | 0,0202 | 11,70 |
| 1 | PB1 | 0,193 | 9,65 | 4,27 | 7,74 | 2,63 | 0,0304 | 15,72 |
| 1 | PTB1 | 0,617 | 12,27 | 10,88 | 7,50 | 3,03 | 0,0360 | 5,83 |
| 1 | PTB1 | 0,596 | 13,94 | 9,56 | 7,78 | 3,13 | 0,0241 | 4,04 |
| 1 | PTB1 | 0,631 | 14,03 | 7,09 | 8,72 | 3,53 | 0,0246 | 3,90 |
| 1 | PTB1 | 0,117 | 13,31 | 8,11 | 8,06 | 2,92 | 0,0166 | 14,19 |
| 1 | PTB1 | 0,485 | 11,04 | 7,89 | 8,60 | 3,85 | 0,0375 | 7,73 |
| 1 | PTB1 | 0,512 | 12,23 | 8,15 | 7,32 | 3,19 | 0,0410 | 8,01 |
| 1 | PTB1 | 0,596 | 13,28 | 8,94 | 8,84 | 4,48 | 0,0577 | 9,68 |
| 1 | PTB1 | 0,479 | 12,86 | 9,47 | 8,68 | 3,59 | 0,0323 | 6,74 |
| 1 | PTB1 | 0,507 | 13,63 | 8,73 | 7,26 | 3,75 | 0,0359 | 7,08 |
| 1 | PTB1 | 0,615 | 12,73 | 8,64 | 7,49 | 3,06 | 0,0375 | 6,10 |
| 1 | PTB1 | 0,539 | 12,83 | 8,49 | 8,71 | 3,45 | 0,0589 | 10,92 |
| 1 | PTB1 | 0,640 | 12,11 | 9,02 | 8,45 | 3,13 | 0,0356 | 5,56 |
| 1 | PTB1 | 0,488 | 13,00 | 8,79 | 7,05 | 3,23 | 0,0419 | 8,58 |
| 1 | PTB1 | 0,463 | 13,46 | 8,36 | 7,61 | 2,57 | 0,0329 | 7,11 |
| 1 | PTB1 | 0,640 | 13,34 | 8,86 | 8,02 | 3,30 | 0,0249 | 3,89 |
| 1 | PTB1 | 0,662 | 12,15 | 8,66 | 8,33 | 3,18 | 0,0269 | 4,06 |
| 1 | PTB1 | 0,425 | 11,18 | 9,36 | 6,57 | 3,85 | 0,0489 | 11,50 |
| 1 | PTB1 | 0,556 | 12,83 | 10,69 | 8,09 | 2,58 | 0,0283 | 5,09 |
| 1 | PTB1 | 0,587 | 13,39 | 9,27 | 8,41 | 3,62 | 0,0517 | 8,81 |
| 1 | PTB1 | 0,503 | 12,52 | 7,09 | 8,21 | 3,24 | 0,0283 | 5,63 |
| 1 | PTB1 | 0,565 | 12,17 | 9,65 | 8,16 | 3,43 | 0,0303 | 5,37 |
| 1 | PTB1 | 0,529 | 12,91 | 8,75 | 8,67 | 2,77 | 0,0303 | 5,73 |
| 1 | PTB1 | 0,555 | 12,88 | 10,51 | 7,92 | 2,65 | 0,0257 | 4,63 |
| 1 | PTB1 | 0,485 | 12,76 | 9,75 | 9,18 | 2,71 | 0,0298 | 6,14 |
| 1 | PTB1 | 0,479 | 11,59 | 8,25 | 7,12 | 3,09 | 0,0280 | 5,85 |
| 1 | SFB1 | 0,657 | 13,03 | 7,23 | 8,38 | 1,51 | 0,0287 | 4,37 |
| 1 | SFB1 | 0,715 | 13,20 | 8,70 | 8,00 | 1,74 | 0,0404 | 5,65 |
| 1 | SFB1 | 0,680 | 12,21 | 8,57 | 7,81 | 2,82 | 0,0404 | 5,94 |
| 1 | SFB1 | 0,718 | 9,84 | 9,07 | 7,42 | 2,61 | 0,0475 | 6,62 |
| 1 | SFB1 | 0,528 | 14,34 | 8,20 | 8,28 | 1,63 | 0,0286 | 5,42 |
| 1 | SFB1 | 0,633 | 13,36 | 7,53 | 7,11 | 2,25 | 0,0241 | 3,81 |
| 1 | SFB1 | 0,613 | 13,24 | 8,01 | 7,75 | 2,02 | 0,0258 | 4,21 |
| 1 | SFB1 | 0,612 | 11,38 | 7,13 | 6,10 | 2,62 | 0,0249 | 4,07 |
| 1 | SFB1 | 0,530 | 12,09 | 8,35 | 7,83 | 2,55 | 0,0341 | 6,43 |
| 1 | SFB1 | 0,435 | 12,99 | 8,90 | 7,02 | 2,20 | 0,0279 | 6,41 |
| 1 | SFB1 | 0,624 | 11,95 | 8,71 | 6,09 | 3,05 | 0,0465 | 7,45 |
| 1 | SFB1 | 0,504 | 12,33 | 7,79 | 7,76 | 1,76 | 0,0352 | 6,99 |
| 1 | SFB1 | 0,519 | 10,68 | 7,78 | 7,03 | 2,06 | 0,0348 | 6,71 |
| 1 | SFB1 | 0,645 | 11,33 | 9,21 | 5,61 | 2,21 | 0,0334 | 5,18 |
| 1 | SFB1 | 0,557 | 10,41 | 9,87 | 7,28 | 2,21 | 0,0466 | 8,37 |
| 1 | SFB1 | 0,530 | 11,48 | 8,39 | 5,09 | 2,68 | 0,0361 | 6,81 |
| 1 | SFB1 | 0,531 | 13,22 | 8,56 | 8,00 | 2,22 | 0,0211 | 3,97 |
| 1 | SFB1 | 0,611 | 12,35 | 8,33 | 7,16 | 1,84 | 0,0224 | 3,67 |
| 1 | SFB1 | 0,505 | 13,97 | 7,44 | 6,17 | 2,74 | 0,0588 | 11,66 |
| 1 | SFB1 | 0,471 | 11,54 | 7,97 | 5,92 | 1,84 | 0,0432 | 9,18 |
| 1 | SFB1 | 0,624 | 13,72 | 7,87 | 7,70 | 2,24 | 0,0468 | 7,50 |
| 1 | SFB1 | 0,549 | 11,14 | 9,02 | 7,61 | 1,97 | 0,0517 | 9,42 |
| 1 | SFB1 | 0,543 | 13,03 | 7,86 | 8,61 | 1,83 | 0,0506 | 9,32 |
| 1 | SFB1 | 0,504 | 12,10 | 9,99 | 8,79 | 2,63 | 0,0751 | 14,91 |
| 1 | SFB1 | 0,513 | 11,67 | 7,73 | 7,27 | 2,25 | 0,0539 | 10,52 |
| 1 | PTO2 | 0,477 | 13,36 | 8,18 | 8,03 | 3,02 | 0,0360 | 7,55 |
| 1 | PTO2 | 0,567 | 13,80 | 10,09 | 8,20 | 2,38 | 0,0274 | 4,83 |
| 1 | PTO2 | 0,668 | 12,57 | 9,49 | 7,70 | 2,71 | 0,0374 | 5,60 |
| 1 | PTO2 | 0,594 | 12,35 | 9,47 | 7,23 | 2,70 | 0,0299 | 5,04 |
| 1 | PTO2 | 0,481 | 13,84 | 8,46 | 9,01 | 2,11 | 0,0325 | 6,75 |
| 1 | PTO2 | 0,586 | 13,01 | 7,78 | 8,10 | 2,67 | 0,0350 | 5,97 |
| 1 | PTO2 | 0,411 | 13,15 | 8,10 | 7,82 | 2,55 | 0,0303 | 7,37 |
| 1 | PTO2 | 0,603 | 13,87 | 9,27 | 7,65 | 3,61 | 0,0416 | 6,90 |
| 1 | PTO2 | 0,622 | 13,31 | 8,75 | 8,22 | 2,79 | 0,0317 | 5,10 |
| 1 | PTO2 | 0,556 | 12,80 | 9,38 | 8,58 | 3,09 | 0,0330 | 5,94 |
| 1 | PTO2 | 0,616 | 14,14 | 9,60 | 8,18 | 2,69 | 0,0318 | 5,16 |
| 1 | PTO2 | 0,501 | 14,67 | 8,67 | 7,73 | 2,63 | 0,0269 | 5,37 |
| 1 | PTO2 | 0,342 | 11,43 | 8,15 | 6,24 | 2,08 | 0,0186 | 5,44 |
| 1 | PTO2 | 0,649 | 13,81 | 9,10 | 8,58 | 2,72 | 0,0381 | 5,87 |
| 1 | PTO2 | 0,623 | 13,06 | 8,88 | 8,30 | 2,11 | 0,0276 | 4,43 |
| 1 | PTO2 | 0,597 | 14,80 | 9,50 | 8,91 | 2,56 | 0,0371 | 6,21 |
| 1 | PTO2 | 0,477 | 13,61 | 9,29 | 7,14 | 3,66 | 0,0343 | 7,19 |
| 1 | PTO2 | 0,549 | 13,28 | 9,28 | 7,31 | 3,12 | 0,0372 | 6,77 |
| 1 | PTO2 | 0,556 | 13,61 | 8,95 | 8,61 | 3,05 | 0,0324 | 5,83 |
| 1 | PTO2 | 0,744 | 15,15 | 9,18 | 8,65 | 3,31 | 0,0380 | 5,11 |
| 1 | PTO2 | 0,790 | 12,94 | 9,66 | 7,94 | 2,95 | 0,0386 | 4,88 |
| 1 | PTO2 | 0,534 | 13,15 | 9,24 | 8,42 | 2,46 | 0,0295 | 5,52 |
| 1 | PTO2 | 0,632 | 12,93 | 10,66 | 7,49 | 3,33 | 0,0307 | 4,86 |
| 1 | PTO2 | 0,618 | 14,58 | 9,35 | 7,80 | 3,23 | 0,0296 | 4,79 |
| 1 | PTO2 | 0,506 | 14,40 | 8,97 | 8,50 | 2,95 | 0,0271 | 5,35 |
| 1 | PTO3 | 0,615 | 13,27 | 8,95 | 8,35 | 2,91 | 0,0254 | 4,13 |
| 1 | PTO3 | 0,629 | 14,02 | 9,33 | 9,38 | 2,64 | 0,0236 | 3,75 |
| 1 | PTO3 | 0,459 | 11,97 | 8,15 | 8,45 | 3,05 | 0,0220 | 4,79 |
| 1 | PTO3 | 0,612 | 15,38 | 8,62 | 8,93 | 3,24 | 0,0249 | 4,07 |
| 1 | PTO3 | 0,551 | 12,30 | 7,70 | 8,59 | 2,67 | 0,0211 | 3,83 |
| 1 | PTO3 | 0,540 | 13,17 | 9,22 | 8,54 | 3,14 | 0,0205 | 3,80 |
| 1 | PTO3 | 0,422 | 13,67 | 7,51 | 8,81 | 2,98 | 0,0257 | 6,09 |
| 1 | PTO3 | 0,601 | 12,47 | 8,22 | 7,58 | 2,42 | 0,0225 | 3,74 |
| 1 | PTO3 | 0,741 | 14,34 | 8,70 | 9,20 | 3,36 | 0,0293 | 3,95 |
| 1 | PTO3 | 0,673 | 13,94 | 8,96 | 9,18 | 2,47 | 0,0221 | 3,28 |
| 1 | PTO3 | 0,562 | 12,68 | 6,92 | 8,00 | 2,59 | 0,0195 | 3,47 |
| 1 | PTO3 | 0,376 | 11,74 | 7,41 | 8,13 | 3,05 | 0,0211 | 5,61 |
| 1 | PTO3 | 0,525 | 12,20 | 9,28 | 7,70 | 2,50 | 0,0183 | 3,48 |
| 1 | PTO3 | 0,380 | 11,22 | 6,53 | 8,11 | 2,73 | 0,0223 | 5,86 |
| 1 | PTO3 | 0,625 | 13,69 | 10,01 | 8,45 | 3,36 | 0,0264 | 4,23 |
| 1 | PTO3 | 0,576 | 13,24 | 8,29 | 7,62 | 3,31 | 0,0238 | 4,13 |
| 1 | PTO3 | 0,640 | 11,84 | 9,98 | 8,06 | 3,02 | 0,0251 | 3,92 |
| 1 | PTO3 | 0,558 | 15,29 | 8,73 | 7,57 | 3,15 | 0,0185 | 3,32 |
| 1 | PTO3 | 0,483 | 12,15 | 8,62 | 8,09 | 2,81 | 0,0213 | 4,41 |
| 1 | PTO3 | 0,513 | 12,29 | 8,98 | 7,53 | 3,58 | 0,0157 | 3,06 |
| 1 | PTO3 | 0,536 | 13,55 | 6,77 | 8,21 | 2,27 | 0,0213 | 3,97 |
| 1 | PTO3 | 0,336 | 12,10 | 6,72 | 7,55 | 3,02 | 0,0207 | 6,16 |
| 1 | PTO3 | 0,565 | 13,21 | 8,17 | 10,34 | 2,42 | 0,0144 | 2,55 |
| 1 | PTO3 | 0,564 | 12,46 | 8,60 | 7,34 | 3,32 | 0,0234 | 4,15 |
| 1 | PTO3 | 0,464 | 11,48 | 7,73 | 7,88 | 2,31 | 0,0161 | 3,47 |
| 1 | PTO4 | 0,572 | 15,19 | 9,40 | 8,59 | 2,79 | 0,0340 | 5,94 |
| 1 | PTO4 | 0,595 | 15,18 | 10,22 | 8,43 | 2,59 | 0,0223 | 3,75 |
| 1 | PTO4 | 0,396 | 13,43 | 8,76 | 7,51 | 2,00 | 0,0320 | 8,08 |
| 1 | PTO4 | 0,731 | 15,76 | 8,92 | 9,84 | 2,33 | 0,0342 | 4,68 |
| 1 | PTO4 | 0,627 | 14,28 | 9,83 | 7,07 | 2,57 | 0,0317 | 5,06 |
| 1 | PTO4 | 0,620 | 13,93 | 10,81 | 8,44 | 2,15 | 0,0224 | 3,61 |
| 1 | PTO4 | 0,714 | 15,31 | 9,83 | 8,47 | 2,39 | 0,0382 | 5,35 |
| 1 | PTO4 | 0,605 | 15,35 | 9,59 | 8,72 | 2,38 | 0,0365 | 6,04 |
| 1 | PTO4 | 0,557 | 15,19 | 9,70 | 8,41 | 2,78 | 0,0325 | 5,83 |
| 1 | PTO4 | 0,644 | 15,64 | 10,39 | 8,54 | 2,96 | 0,0329 | 5,11 |
| 1 | PTO4 | 0,498 | 16,23 | 11,74 | 8,61 | 2,76 | 0,0331 | 6,65 |
| 1 | PTO4 | 0,467 | 13,26 | 8,29 | 8,56 | 2,02 | 0,0290 | 6,21 |
| 1 | PTO4 | 0,435 | 12,25 | 8,67 | 7,90 | 2,48 | 0,0280 | 6,44 |
| 1 | PTO4 | 0,552 | 13,05 | 6,61 | 8,15 | 2,52 | 0,0343 | 6,21 |
| 1 | PTO4 | 0,545 | 14,50 | 10,18 | 8,45 | 2,05 | 0,0270 | 4,95 |
| 1 | PTO4 | 0,610 | 16,38 | 10,37 | 8,97 | 2,53 | 0,0256 | 4,20 |
| 1 | PTO4 | 0,546 | 16,41 | 8,52 | 10,23 | 1,92 | 0,0255 | 4,67 |
| 1 | PTO4 | 0,374 | 11,64 | 7,90 | 6,65 | 1,92 | 0,0207 | 5,54 |
| 1 | PTO4 | 0,548 | 15,80 | 9,18 | 8,44 | 2,89 | 0,0284 | 5,18 |
| 1 | PTO4 | 0,585 | 14,97 | 9,69 | 8,91 | 2,60 | 0,0373 | 6,38 |
| 1 | PTO4 | 0,393 | 12,74 | 9,02 | 8,23 | 2,82 | 0,0319 | 8,11 |
| 1 | PTO4 | 0,503 | 14,28 | 9,58 | 8,95 | 1,57 | 0,0344 | 6,84 |
| 1 | PTO4 | 0,601 | 13,91 | 9,48 | 7,52 | 2,52 | 0,0228 | 3,80 |
| 1 | PTO4 | 0,592 | 13,58 | 9,23 | 7,60 | 2,57 | 0,0283 | 4,78 |
| 1 | PTO4 | 0,399 | 12,92 | 8,23 | 8,49 | 2,22 | 0,0212 | 5,32 |
| 1 | PTO5 | 0,279 | 9,91 | 6,47 | 7,02 | 2,46 | 0,0314 | 11,27 |
| 1 | PTO5 | 0,275 | 9,96 | 7,15 | 7,62 | 3,63 | 0,0373 | 13,55 |
| 1 | PTO5 | 0,321 | 9,76 | 7,22 | 7,52 | 3,17 | 0,0339 | 10,56 |
| 1 | PTO5 | 0,320 | 10,31 | 7,26 | 6,62 | 2,57 | 0,0286 | 8,94 |
| 1 | PTO5 | 0,316 | 10,38 | 7,01 | 7,35 | 2,31 | 0,0313 | 9,91 |
| 1 | PTO5 | 0,250 | 9,80 | 6,01 | 6,83 | 2,46 | 0,0408 | 16,34 |
| 1 | PTO5 | 0,318 | 10,09 | 5,77 | 8,09 | 2,28 | 0,0271 | 8,52 |
| 1 | PTO5 | 0,323 | 10,29 | 6,95 | 7,46 | 2,55 | 0,0268 | 8,29 |
| 1 | PTO5 | 0,286 | 9,42 | 6,67 | 6,73 | 2,74 | 0,0270 | 9,43 |
| 1 | PTO5 | 0,268 | 9,30 | 6,65 | 6,60 | 2,85 | 0,0258 | 9,64 |
| 1 | PTO5 | 0,326 | 11,32 | 7,13 | 7,21 | 3,09 | 0,0367 | 11,25 |
| 1 | PTO5 | 0,329 | 10,81 | 7,47 | 6,70 | 2,47 | 0,0276 | 8,39 |
| 1 | PTO5 | 0,309 | 10,28 | 7,80 | 7,32 | 2,14 | 0,0313 | 10,14 |
| 1 | PTO5 | 0,283 | 9,65 | 6,49 | 7,45 | 3,07 | 0,0346 | 12,21 |
| 1 | PTO5 | 0,279 | 10,39 | 6,70 | 7,33 | 2,88 | 0,0370 | 13,29 |
| 1 | PTO5 | 0,333 | 10,61 | 7,55 | 7,05 | 3,02 | 0,0395 | 11,85 |
| 1 | PTO5 | 0,334 | 11,34 | 7,54 | 7,17 | 2,19 | 0,0285 | 8,53 |
| 1 | PTO5 | 0,290 | 10,10 | 7,70 | 6,90 | 2,86 | 0,0379 | 13,09 |
| 1 | PTO5 | 0,205 | 10,05 | 6,31 | 8,00 | 3,07 | 0,0355 | 17,31 |
| 1 | PTO5 | 0,291 | 9,43 | 7,89 | 7,31 | 2,73 | 0,0317 | 10,90 |
| 1 | PTO5 | 0,306 | 9,32 | 7,27 | 6,87 | 2,17 | 0,0297 | 9,72 |
| 1 | PTO5 | 0,244 | 9,19 | 6,78 | 6,88 | 2,87 | 0,0293 | 11,99 |
| 1 | PTO5 | 0,237 | 8,80 | 6,28 | 7,29 | 2,79 | 0,0336 | 14,21 |
| 1 | PTO5 | 0,164 | 7,97 | 5,97 | 6,34 | 2,86 | 0,0257 | 15,72 |
| 1 | PTO5 | 0,217 | 7,71 | 5,90 | 7,46 | 2,79 | 0,0270 | 12,42 |
| 1 | PTO6 | 0,610 | 12,39 | 8,25 | 7,70 | 2,61 | 0,0353 | 5,79 |
| 1 | PTO6 | 0,601 | 12,35 | 8,16 | 8,87 | 2,89 | 0,0345 | 5,74 |
| 1 | PTO6 | 0,546 | 13,54 | 8,03 | 8,53 | 2,19 | 0,0322 | 5,90 |
| 1 | PTO6 | 0,664 | 14,00 | 9,23 | 8,66 | 2,04 | 0,0360 | 5,42 |
| 1 | PTO6 | 0,508 | 12,40 | 5,94 | 8,43 | 2,54 | 0,0335 | 6,59 |
| 1 | PTO6 | 0,403 | 12,06 | 5,78 | 7,71 | 2,22 | 0,0375 | 9,31 |
| 1 | PTO6 | 0,683 | 12,32 | 7,88 | 7,18 | 3,05 | 0,0365 | 5,34 |
| 1 | PTO6 | 0,701 | 13,11 | 9,13 | 7,78 | 2,08 | 0,0389 | 5,55 |
| 1 | PTO6 | 0,397 | 12,97 | 6,09 | 7,66 | 2,53 | 0,0291 | 7,33 |
| 1 | PTO6 | 0,571 | 14,19 | 7,30 | 9,17 | 2,08 | 0,0339 | 5,94 |
| 1 | PTO6 | 0,615 | 12,15 | 8,16 | 7,14 | 2,40 | 0,0387 | 6,29 |
| 1 | PTO6 | 0,716 | 12,71 | 8,40 | 7,31 | 2,64 | 0,0437 | 6,10 |
| 1 | PTO6 | 0,576 | 13,18 | 7,17 | 7,96 | 2,33 | 0,0289 | 5,02 |
| 1 | PTO6 | 0,618 | 14,82 | 8,65 | 7,84 | 2,63 | 0,0348 | 5,63 |
| 1 | PTO6 | 0,590 | 13,13 | 7,13 | 9,45 | 2,06 | 0,0406 | 6,88 |
| 1 | PTO6 | 0,646 | 14,17 | 10,38 | 7,79 | 3,08 | 0,0354 | 5,48 |
| 1 | PTO6 | 0,743 | 13,60 | 9,39 | 8,04 | 2,24 | 0,0358 | 4,82 |
| 1 | PTO6 | 0,655 | 13,92 | 7,49 | 7,50 | 3,48 | 0,0344 | 5,25 |
| 1 | PTO6 | 0,575 | 14,29 | 7,89 | 8,61 | 2,05 | 0,0372 | 6,47 |
| 1 | PTO6 | 0,474 | 15,02 | 7,59 | 8,88 | 2,62 | 0,0387 | 8,16 |
| 1 | PTO6 | 0,569 | 12,29 | 7,06 | 7,60 | 2,28 | 0,0359 | 6,31 |
| 1 | PTO6 | 0,535 | 13,15 | 7,26 | 7,08 | 2,44 | 0,0341 | 6,37 |
| 1 | PTO6 | 0,426 | 12,57 | 6,69 | 7,74 | 3,33 | 0,0473 | 11,10 |
| 1 | PTO6 | 0,600 | 12,28 | 7,31 | 7,21 | 2,89 | 0,0354 | 5,90 |
| 1 | PTO6 | 0,453 | 14,96 | 7,30 | 9,03 | 2,50 | 0,0344 | 7,59 |
| 1 | PTO7 | 0,525 | 13,44 | 6,77 | 7,35 | 2,28 | 0,0383 | 7,30 |
| 1 | PTO7 | 0,665 | 12,82 | 6,04 | 8,17 | 2,37 | 0,0510 | 7,67 |
| 1 | PTO7 | 0,449 | 9,57 | 5,93 | 7,70 | 1,68 | 0,0465 | 10,35 |
| 1 | PTO7 | 0,627 | 12,93 | 6,73 | 7,96 | 2,38 | 0,0585 | 9,33 |
| 1 | PTO7 | 0,704 | 11,80 | 7,00 | 6,86 | 2,58 | 0,0375 | 5,33 |
| 1 | PTO7 | 0,588 | 14,28 | 8,00 | 7,17 | 1,68 | 0,0394 | 6,70 |
| 1 | PTO7 | 0,668 | 12,85 | 9,58 | 7,75 | 2,54 | 0,0510 | 7,63 |
| 1 | PTO7 | 0,603 | 12,00 | 8,50 | 7,45 | 2,14 | 0,0454 | 7,54 |
| 1 | PTO7 | 0,690 | 11,95 | 7,00 | 6,14 | 1,83 | 0,0573 | 8,31 |
| 1 | PTO7 | 0,442 | 11,03 | 8,11 | 7,02 | 2,04 | 0,0371 | 8,39 |
| 1 | PTO7 | 0,546 | 12,95 | 6,20 | 7,93 | 1,82 | 0,0401 | 7,35 |
| 1 | PTO7 | 0,631 | 12,00 | 7,29 | 7,30 | 2,51 | 0,0454 | 7,19 |
| 1 | PTO7 | 0,418 | 12,34 | 5,92 | 7,26 | 2,46 | 0,0452 | 10,81 |
| 1 | PTO7 | 0,519 | 10,86 | 6,45 | 7,75 | 2,17 | 0,0525 | 10,13 |
| 1 | PTO7 | 0,431 | 10,12 | 6,05 | 7,49 | 2,18 | 0,0451 | 10,47 |
| 1 | PTO7 | 0,601 | 11,24 | 6,92 | 5,95 | 1,86 | 0,0345 | 5,74 |
| 1 | PTO7 | 0,271 | 9,39 | 6,56 | 6,75 | 2,06 | 0,0385 | 14,23 |
| 1 | PTO7 | 0,695 | 12,65 | 7,08 | 8,35 | 2,79 | 0,0496 | 7,14 |
| 1 | PTO7 | 0,242 | 13,32 | 3,87 | 8,31 | 2,06 | 0,0379 | 15,64 |
| 1 | PTO7 | 0,531 | 11,88 | 6,39 | 7,57 | 2,16 | 0,0369 | 6,95 |
| 1 | PTO7 | 0,516 | 11,89 | 6,18 | 7,39 | 2,47 | 0,0394 | 7,64 |
| 1 | PTO7 | 0,458 | 9,63 | 6,73 | 7,12 | 1,97 | 0,0430 | 9,39 |
| 1 | PTO7 | 0,587 | 12,42 | 6,84 | 7,23 | 2,11 | 0,0642 | 10,94 |
| 1 | PTO7 | 0,469 | 12,07 | 4,93 | 7,70 | 2,31 | 0,0469 | 10,00 |
| 1 | PTO7 | 0,409 | 10,40 | 5,78 | 5,07 | 2,35 | 0,0423 | 10,33 |
| 1 | PTO8 | 0,658 | 13,13 | 9,90 | 7,64 | 2,90 | 0,0334 | 5,08 |
| 1 | PTO8 | 0,681 | 14,13 | 9,71 | 6,57 | 3,11 | 0,0338 | 4,96 |
| 1 | PTO8 | 0,696 | 15,64 | 9,90 | 8,57 | 2,64 | 0,0390 | 5,60 |
| 1 | PTO8 | 0,541 | 14,88 | 9,68 | 9,34 | 2,01 | 0,0363 | 6,71 |
| 1 | PTO8 | 0,636 | 13,85 | 10,02 | 6,88 | 2,45 | 0,0388 | 6,10 |
| 1 | PTO8 | 0,681 | 12,96 | 9,94 | 8,08 | 3,75 | 0,0430 | 6,32 |
| 1 | PTO8 | 0,633 | 13,77 | 11,52 | 6,83 | 3,32 | 0,0340 | 5,37 |
| 1 | PTO8 | 0,614 | 13,50 | 7,97 | 7,06 | 2,78 | 0,0506 | 8,25 |
| 1 | PTO8 | 0,741 | 17,15 | 11,19 | 8,87 | 1,90 | 0,0382 | 5,15 |
| 1 | PTO8 | 0,654 | 16,25 | 9,83 | 8,91 | 2,25 | 0,0309 | 4,72 |
| 1 | PTO8 | 0,635 | 14,01 | 8,70 | 9,13 | 2,33 | 0,0297 | 4,68 |
| 1 | PTO8 | 0,526 | 14,75 | 9,64 | 7,75 | 2,66 | 0,0309 | 5,88 |
| 1 | PTO8 | 0,730 | 15,57 | 9,75 | 8,53 | 2,73 | 0,0391 | 5,36 |
| 1 | PTO8 | 0,655 | 14,25 | 9,81 | 7,23 | 2,96 | 0,0327 | 4,99 |
| 1 | PTO8 | 0,694 | 13,92 | 10,23 | 8,10 | 2,43 | 0,0334 | 4,81 |
| 1 | PTO8 | 0,613 | 15,32 | 10,16 | 7,34 | 2,36 | 0,0286 | 4,67 |
| 1 | PTO8 | 0,517 | 15,88 | 8,25 | 9,39 | 2,25 | 0,0285 | 5,52 |
| 1 | PTO8 | 0,453 | 12,20 | 8,44 | 7,43 | 2,49 | 0,0251 | 5,55 |
| 1 | PTO8 | 0,439 | 12,63 | 9,89 | 7,85 | 2,76 | 0,0256 | 5,83 |
| 1 | PTO8 | 0,587 | 14,45 | 9,87 | 8,38 | 2,01 | 0,0325 | 5,54 |
| 1 | PTO8 | 0,386 | 12,54 | 9,43 | 8,53 | 3,21 | 0,0350 | 9,07 |
| 1 | PTO8 | 0,596 | 14,95 | 9,46 | 9,50 | 2,24 | 0,0274 | 4,60 |
| 1 | PTO8 | 0,625 | 13,89 | 10,44 | 7,92 | 2,85 | 0,0291 | 4,66 |
| 1 | PTO8 | 0,640 | 12,30 | 10,71 | 7,49 | 2,75 | 0,0305 | 4,77 |
| 1 | PTO8 | 0,653 | 13,49 | 10,43 | 7,23 | 2,98 | 0,0284 | 4,35 |
| 1 | PTO9 | 0,405 | 9,80 | 9,38 | 6,76 | 3,55 | 0,0394 | 9,74 |
| 1 | PTO9 | 0,632 | 12,26 | 9,80 | 7,01 | 2,81 | 0,0360 | 5,69 |
| 1 | PTO9 | 0,692 | 13,66 | 10,15 | 8,14 | 2,51 | 0,0361 | 5,22 |
| 1 | PTO9 | 0,582 | 14,30 | 9,49 | 8,16 | 1,91 | 0,0321 | 5,52 |
| 1 | PTO9 | 0,708 | 13,50 | 10,70 | 7,88 | 2,17 | 0,0285 | 4,03 |
| 1 | PTO9 | 0,685 | 14,72 | 8,79 | 8,73 | 1,83 | 0,0288 | 4,21 |
| 1 | PTO9 | 0,652 | 14,86 | 11,66 | 7,32 | 1,98 | 0,0274 | 4,20 |
| 1 | PTO9 | 0,729 | 16,34 | 9,19 | 9,07 | 1,93 | 0,0250 | 3,43 |
| 1 | PTO9 | 0,385 | 11,75 | 7,64 | 7,25 | 2,71 | 0,0392 | 10,18 |
| 1 | PTO9 | 0,503 | 14,41 | 9,96 | 8,07 | 2,14 | 0,0248 | 4,93 |
| 1 | PTO9 | 0,718 | 14,79 | 10,07 | 7,77 | 2,19 | 0,0264 | 3,68 |
| 1 | PTO9 | 0,610 | 14,82 | 9,68 | 7,72 | 2,19 | 0,0225 | 3,69 |
| 1 | PTO9 | 0,745 | 14,82 | 9,03 | 8,94 | 2,50 | 0,0282 | 3,79 |
| 1 | PTO9 | 0,680 | 15,60 | 10,18 | 8,13 | 2,01 | 0,0277 | 4,07 |
| 1 | PTO9 | 0,743 | 15,48 | 10,56 | 8,89 | 2,26 | 0,0231 | 3,11 |
| 1 | PTO9 | 0,629 | 13,58 | 10,04 | 8,86 | 2,66 | 0,0347 | 5,51 |
| 1 | PTO9 | 0,384 | 11,82 | 8,18 | 8,26 | 2,75 | 0,0447 | 11,64 |
| 1 | PTO9 | 0,747 | 14,64 | 10,53 | 8,02 | 2,67 | 0,0386 | 5,17 |
| 1 | PTO9 | 0,422 | 12,28 | 8,28 | 8,07 | 2,75 | 0,0434 | 10,28 |
| 1 | PTO9 | 0,773 | 15,02 | 10,40 | 8,12 | 2,55 | 0,0344 | 4,45 |
| 1 | PTO9 | 0,632 | 15,68 | 10,16 | 7,96 | 1,95 | 0,0257 | 4,07 |
| 1 | PTO9 | 0,707 | 14,27 | 11,26 | 8,95 | 1,91 | 0,0260 | 3,68 |
| 1 | PTO9 | 0,768 | 15,03 | 9,72 | 7,91 | 2,41 | 0,0259 | 3,37 |
| 1 | PTO9 | 0,560 | 13,90 | 10,10 | 7,24 | 2,31 | 0,0279 | 4,99 |
| 1 | PTO9 | 0,540 | 13,77 | 9,01 | 7,87 | 3,12 | 0,0255 | 4,72 |
| 1 | PDO2 | 0,577 | 12,86 | 8,61 | 6,78 | 2,18 | 0,0251 | 4,35 |
| 1 | PDO2 | 0,378 | 11,19 | 6,84 | 8,75 | 2,72 | 0,0520 | 13,76 |
| 1 | PDO2 | 0,385 | 10,57 | 6,99 | 7,65 | 3,34 | 0,0478 | 12,42 |
| 1 | PDO2 | 0,395 | 10,73 | 8,86 | 7,85 | 3,49 | 0,0434 | 10,99 |
| 1 | PDO2 | 0,335 | 10,90 | 6,12 | 7,96 | 3,91 | 0,0495 | 14,78 |
| 1 | PDO2 | 0,644 | 12,96 | 8,52 | 7,92 | 2,96 | 0,0437 | 6,79 |
| 1 | PDO2 | 0,398 | 10,72 | 6,50 | 7,82 | 3,15 | 0,0413 | 10,38 |
| 1 | PDO2 | 0,435 | 12,00 | 6,76 | 6,90 | 2,39 | 0,0277 | 6,37 |
| 1 | PDO2 | 0,265 | 10,69 | 5,28 | 5,76 | 3,15 | 0,0450 | 16,98 |
| 1 | PDO2 | 0,266 | 10,02 | 5,85 | 7,59 | 2,93 | 0,0429 | 16,13 |
| 1 | PDO2 | 0,480 | 12,38 | 8,93 | 7,89 | 2,27 | 0,0402 | 8,38 |
| 1 | PDO2 | 0,376 | 12,08 | 6,73 | 7,62 | 3,12 | 0,0607 | 16,14 |
| 1 | PDO2 | 0,361 | 10,76 | 6,72 | 7,62 | 3,20 | 0,0511 | 14,16 |
| 1 | PDO2 | 0,340 | 9,71 | 6,33 | 6,64 | 2,69 | 0,0386 | 11,35 |
| 1 | PDO2 | 0,430 | 12,38 | 6,80 | 8,68 | 2,79 | 0,0563 | 13,09 |
| 1 | PDO2 | 0,483 | 12,44 | 8,22 | 8,52 | 3,08 | 0,0643 | 13,31 |
| 1 | PDO2 | 0,569 | 12,54 | 7,46 | 6,80 | 2,89 | 0,0355 | 6,24 |
| 1 | PDO2 | 0,314 | 8,74 | 5,85 | 6,82 | 2,81 | 0,0409 | 13,03 |
| 1 | PDO2 | 0,303 | 10,56 | 7,54 | 8,12 | 3,44 | 0,0531 | 17,52 |
| 1 | PDO2 | 0,330 | 10,54 | 8,19 | 7,86 | 3,21 | 0,0480 | 14,55 |
| 1 | PDO2 | 0,389 | 11,68 | 6,16 | 6,40 | 3,25 | 0,0480 | 12,34 |
| 1 | PDO2 | 0,390 | 11,87 | 6,33 | 8,29 | 3,23 | 0,0495 | 12,69 |
| 1 | PDO2 | 0,406 | 11,60 | 7,77 | 7,35 | 2,79 | 0,0396 | 9,75 |
| 1 | PDO2 | 0,353 | 11,94 | 5,12 | 8,47 | 1,65 | 0,0447 | 12,66 |
| 1 | PDO2 | 0,381 | 11,75 | 7,61 | 9,04 | 2,70 | 0,0642 | 16,85 |
| 1 | CRO1 | 0,429 | 13,13 | 8,75 | 7,58 | 2,26 | 0,0296 | 6,89 |
| 1 | CRO1 | 0,572 | 12,64 | 8,36 | 8,03 | 2,66 | 0,0398 | 6,96 |
| 1 | CRO1 | 0,573 | 13,87 | 8,08 | 8,03 | 2,31 | 0,0332 | 5,79 |
| 1 | CRO1 | 0,624 | 14,30 | 9,34 | 7,03 | 2,23 | 0,0277 | 4,44 |
| 1 | CRO1 | 0,671 | 12,85 | 9,96 | 6,99 | 3,90 | 0,0348 | 5,19 |
| 1 | CRO1 | 0,506 | 15,33 | 6,99 | 9,58 | 2,54 | 0,0325 | 6,43 |
| 1 | CRO1 | 0,618 | 14,50 | 7,35 | 9,24 | 3,05 | 0,0409 | 6,62 |
| 1 | CRO1 | 0,603 | 13,59 | 9,54 | 6,52 | 2,88 | 0,0233 | 3,86 |
| 1 | CRO1 | 0,590 | 14,21 | 9,17 | 8,76 | 2,63 | 0,0434 | 7,36 |
| 1 | CRO1 | 0,521 | 14,08 | 8,22 | 8,94 | 2,16 | 0,0274 | 5,26 |
| 1 | CRO1 | 0,558 | 13,92 | 9,01 | 7,84 | 1,89 | 0,0260 | 4,66 |
| 1 | CRO1 | 0,432 | 13,30 | 7,41 | 8,40 | 2,36 | 0,0318 | 7,35 |
| 1 | CRO1 | 0,602 | 14,85 | 6,29 | 8,41 | 3,00 | 0,0389 | 6,46 |
| 1 | CRO1 | 0,679 | 12,26 | 7,82 | 6,84 | 3,51 | 0,0284 | 4,19 |
| 1 | CRO1 | 0,423 | 12,57 | 6,63 | 7,30 | 2,53 | 0,0301 | 7,11 |
| 1 | CRO1 | 0,586 | 13,81 | 8,25 | 7,70 | 2,11 | 0,0304 | 5,19 |
| 1 | CRO1 | 0,590 | 11,56 | 8,95 | 6,42 | 2,18 | 0,0322 | 5,46 |
| 1 | CRO1 | 0,597 | 15,36 | 8,73 | 9,26 | 2,29 | 0,0333 | 5,58 |
| 1 | CRO1 | 0,716 | 16,42 | 9,08 | 7,90 | 2,36 | 0,0210 | 2,93 |
| 1 | CRO1 | 0,720 | 13,68 | 9,46 | 7,57 | 2,44 | 0,0301 | 4,18 |
| 1 | CRO1 | 0,641 | 14,17 | 8,50 | 7,66 | 2,36 | 0,0281 | 4,38 |
| 1 | CRO1 | 0,535 | 12,43 | 6,66 | 7,93 | 2,48 | 0,0359 | 6,71 |
| 1 | CRO1 | 0,616 | 13,99 | 7,36 | 7,90 | 2,38 | 0,0425 | 6,90 |
| 1 | CRO1 | 0,593 | 13,47 | 8,22 | 7,69 | 2,60 | 0,0340 | 5,74 |
| 1 | CRO1 | 0,598 | 12,35 | 10,10 | 6,97 | 3,13 | 0,0284 | 4,75 |
| 1 | CRO2 | 0,648 | 14,54 | 8,61 | 9,16 | 3,16 | 0,0418 | 6,45 |
| 1 | CRO2 | 0,693 | 13,80 | 8,40 | 6,72 | 2,15 | 0,0277 | 4,00 |
| 1 | CRO2 | 0,700 | 15,28 | 9,40 | 9,66 | 3,20 | 0,0361 | 5,16 |
| 1 | CRO2 | 0,541 | 12,95 | 7,55 | 7,22 | 2,00 | 0,0281 | 5,20 |
| 1 | CRO2 | 0,598 | 12,59 | 8,17 | 8,51 | 1,84 | 0,0355 | 5,94 |
| 1 | CRO2 | 0,594 | 12,56 | 7,82 | 7,92 | 2,89 | 0,0324 | 5,45 |
| 1 | CRO2 | 0,423 | 12,24 | 5,88 | 6,89 | 3,04 | 0,0244 | 5,78 |
| 1 | CRO2 | 0,559 | 12,90 | 8,06 | 7,35 | 2,89 | 0,0348 | 6,23 |
| 1 | CRO2 | 0,624 | 13,91 | 7,24 | 8,06 | 2,65 | 0,0270 | 4,32 |
| 1 | CRO2 | 0,449 | 10,60 | 8,90 | 5,49 | 2,75 | 0,0308 | 6,87 |
| 1 | CRO2 | 0,597 | 12,90 | 9,05 | 7,66 | 2,85 | 0,0334 | 5,60 |
| 1 | CRO2 | 0,782 | 15,48 | 8,42 | 9,05 | 2,47 | 0,0418 | 5,35 |
| 1 | CRO2 | 0,704 | 13,52 | 9,08 | 8,41 | 2,87 | 0,0361 | 5,13 |
| 1 | CRO2 | 0,589 | 12,60 | 7,35 | 8,16 | 2,70 | 0,0315 | 5,35 |
| 1 | CRO2 | 0,729 | 12,93 | 9,78 | 7,57 | 2,89 | 0,0391 | 5,37 |
| 1 | CRO2 | 0,737 | 12,39 | 7,58 | 7,54 | 2,98 | 0,0348 | 4,72 |
| 1 | CRO2 | 0,570 | 12,02 | 8,74 | 7,63 | 1,37 | 0,0315 | 5,53 |
| 1 | CRO2 | 0,664 | 12,25 | 7,39 | 7,41 | 3,09 | 0,0360 | 5,42 |
| 1 | CRO2 | 0,545 | 10,99 | 6,14 | 6,91 | 3,93 | 0,0310 | 5,68 |
| 1 | CRO2 | 0,673 | 14,05 | 7,74 | 7,65 | 3,20 | 0,0307 | 4,56 |
| 1 | CRO2 | 0,578 | 13,00 | 8,87 | 7,68 | 2,53 | 0,0226 | 3,91 |
| 1 | CRO2 | 0,609 | 14,31 | 8,06 | 8,93 | 2,41 | 0,0329 | 5,40 |
| 1 | CRO2 | 0,579 | 12,44 | 9,35 | 6,12 | 2,88 | 0,0231 | 3,99 |
| 1 | CRO2 | 0,636 | 12,04 | 8,96 | 6,41 | 2,90 | 0,0300 | 4,72 |
| 1 | CRO2 | 0,563 | 12,16 | 7,88 | 6,21 | 2,36 | 0,0235 | 4,18 |
| 1 | ADO4 | 0,550 | 12,45 | 5,57 | 8,31 | 2,21 | 0,0577 | 10,49 |
| 1 | ADO4 | 0,460 | 11,33 | 4,85 | 7,25 | 1,98 | 0,0837 | 18,20 |
| 1 | ADO4 | 0,350 | 10,32 | 5,46 | 7,60 | 2,01 | 0,0511 | 14,59 |
| 1 | ADO4 | 0,302 | 11,03 | 6,40 | 6,57 | 1,36 | 0,0306 | 10,13 |
| 1 | ADO4 | 0,621 | 12,57 | 8,12 | 7,90 | 1,85 | 0,0456 | 7,34 |
| 1 | ADO4 | 0,656 | 15,55 | 9,25 | 7,83 | 2,29 | 0,0380 | 5,80 |
| 1 | ADO4 | 0,682 | 12,24 | 7,92 | 6,95 | 2,10 | 0,0437 | 6,40 |
| 1 | ADO4 | 0,631 | 12,14 | 6,18 | 6,20 | 1,45 | 0,0607 | 9,62 |
| 1 | ADO4 | 0,418 | 11,68 | 5,84 | 7,62 | 1,64 | 0,0393 | 9,41 |
| 1 | ADO4 | 0,168 | 11,98 | 3,50 | 5,42 | 1,86 | 0,0213 | 12,66 |
| 1 | ADO4 | 0,442 | 9,22 | 7,26 | 6,56 | 2,41 | 0,0442 | 10,00 |
| 1 | ADO4 | 0,463 | 11,43 | 5,29 | 7,43 | 2,85 | 0,0495 | 10,68 |
| 1 | ADO4 | 0,690 | 14,21 | 7,46 | 8,33 | 2,46 | 0,0486 | 7,05 |
| 1 | ADO4 | 0,429 | 11,93 | 7,37 | 7,95 | 1,91 | 0,0378 | 8,81 |
| 1 | ADO4 | 0,536 | 10,61 | 6,58 | 6,01 | 2,92 | 0,0442 | 8,25 |
| 1 | ADO4 | 0,466 | 12,12 | 5,43 | 7,87 | 1,86 | 0,0449 | 9,63 |
| 1 | ADO4 | 0,164 | 12,34 | 3,97 | 7,11 | 1,88 | 0,0170 | 10,35 |
| 1 | ADO4 | 0,743 | 12,34 | 8,32 | 7,16 | 2,03 | 0,0460 | 6,19 |
| 1 | ADO4 | 0,536 | 11,95 | 7,43 | 6,55 | 2,37 | 0,0305 | 5,70 |
| 1 | ADO4 | 0,492 | 10,34 | 7,66 | 6,03 | 2,36 | 0,0362 | 7,35 |
| 1 | ADO4 | 0,247 | 9,95 | 3,59 | 6,30 | 2,69 | 0,0359 | 14,55 |
| 1 | ADO4 | 0,301 | 12,15 | 5,59 | 7,34 | 3,34 | 0,0514 | 17,05 |
| 1 | ADO4 | 0,319 | 10,77 | 3,93 | 8,78 | 1,84 | 0,0487 | 15,25 |
| 1 | ADO4 | 0,488 | 11,26 | 6,42 | 7,19 | 1,53 | 0,0425 | 8,71 |
| 1 | ADO4 | 0,277 | 10,05 | 4,05 | 7,11 | 1,43 | 0,0454 | 16,39 |
| 1 | BO1 | 0,198 | 9,21 | 4,74 | 7,17 | 2,00 | 0,0283 | 14,33 |
| 1 | BO1 | 0,249 | 9,07 | 5,72 | 6,31 | 1,96 | 0,0344 | 13,79 |
| 1 | BO1 | 0,284 | 9,92 | 6,57 | 6,62 | 2,16 | 0,0335 | 11,80 |
| 1 | BO1 | 0,327 | 9,32 | 5,75 | 7,07 | 2,03 | 0,0355 | 10,87 |
| 1 | BO1 | 0,279 | 9,06 | 3,94 | 6,29 | 2,01 | 0,0350 | 12,54 |
| 1 | BO1 | 0,363 | 10,84 | 5,68 | 7,70 | 2,30 | 0,0464 | 12,78 |
| 1 | BO1 | 0,224 | 7,26 | 3,60 | 6,30 | 2,14 | 0,0301 | 13,43 |
| 1 | BO1 | 0,200 | 10,32 | 6,90 | 5,49 | 2,77 | 0,0419 | 20,98 |
| 1 | BO1 | 0,412 | 9,43 | 3,33 | 6,28 | 1,85 | 0,0379 | 9,21 |
| 1 | BO1 | 0,344 | 10,03 | 6,15 | 6,59 | 2,16 | 0,0388 | 11,27 |
| 1 | BO1 | 0,288 | 10,15 | 4,87 | 7,05 | 2,81 | 0,0439 | 15,26 |
| 1 | BO1 | 0,365 | 11,07 | 6,00 | 6,84 | 2,47 | 0,0435 | 11,91 |
| 1 | BO1 | 0,262 | 8,08 | 3,88 | 5,25 | 1,92 | 0,0390 | 14,86 |
| 1 | BO1 | 0,289 | 8,93 | 5,39 | 7,13 | 2,79 | 0,0408 | 14,11 |
| 1 | BO1 | 0,270 | 9,69 | 5,11 | 6,83 | 2,64 | 0,0299 | 11,09 |
| 1 | BO1 | 0,336 | 8,37 | 6,22 | 6,66 | 2,39 | 0,0421 | 12,51 |
| 1 | BO1 | 0,274 | 9,38 | 5,07 | 6,30 | 2,42 | 0,0332 | 12,12 |
| 1 | BO1 | 0,220 | 8,80 | 4,67 | 6,84 | 2,46 | 0,0373 | 16,99 |
| 1 | BO1 | 0,424 | 9,70 | 6,98 | 6,24 | 2,90 | 0,0510 | 12,04 |
| 1 | BO1 | 0,386 | 10,45 | 5,22 | 6,75 | 2,08 | 0,0436 | 11,30 |
| 1 | BO1 | 0,373 | 10,13 | 6,38 | 6,70 | 2,57 | 0,0381 | 10,21 |
| 1 | BO1 | 0,289 | 10,02 | 5,47 | 7,11 | 2,10 | 0,0426 | 14,72 |
| 1 | BO1 | 0,296 | 9,01 | 6,18 | 5,73 | 2,46 | 0,0334 | 11,28 |
| 1 | BO1 | 0,393 | 9,88 | 6,10 | 5,94 | 2,93 | 0,0404 | 10,29 |
| 1 | BO1 | 0,320 | 8,86 | 5,81 | 7,07 | 2,34 | 0,0416 | 13,01 |
| 1 | ADO2 | 0,583 | 13,84 | 6,96 | 7,66 | 1,87 | 0,0269 | 4,62 |
| 1 | ADO2 | 0,687 | 12,53 | 8,63 | 5,53 | 2,69 | 0,0272 | 3,96 |
| 1 | ADO2 | 0,504 | 13,72 | 8,24 | 7,94 | 2,35 | 0,0263 | 5,22 |
| 1 | ADO2 | 0,534 | 15,80 | 7,50 | 8,75 | 2,38 | 0,0318 | 5,95 |
| 1 | ADO2 | 0,475 | 13,64 | 8,15 | 8,61 | 1,97 | 0,0211 | 4,45 |
| 1 | ADO2 | 0,530 | 12,64 | 8,20 | 8,33 | 2,63 | 0,0275 | 5,18 |
| 1 | ADO2 | 0,475 | 14,26 | 6,13 | 8,69 | 2,27 | 0,0230 | 4,84 |
| 1 | ADO2 | 0,603 | 15,76 | 7,74 | 9,66 | 1,93 | 0,0337 | 5,59 |
| 1 | ADO2 | 0,569 | 14,00 | 6,83 | 6,12 | 2,84 | 0,0352 | 6,18 |
| 1 | ADO2 | 0,604 | 13,85 | 6,77 | 8,69 | 3,12 | 0,0302 | 5,00 |
| 1 | ADO2 | 0,487 | 12,22 | 7,28 | 8,48 | 1,89 | 0,0371 | 7,62 |
| 1 | ADO2 | 0,437 | 14,39 | 6,14 | 9,15 | 1,69 | 0,0298 | 6,82 |
| 1 | ADO2 | 0,500 | 12,45 | 6,98 | 7,89 | 2,03 | 0,0223 | 4,46 |
| 1 | ADO2 | 0,459 | 13,51 | 6,85 | 8,13 | 1,55 | 0,0214 | 4,66 |
| 1 | ADO2 | 0,459 | 13,86 | 5,98 | 9,10 | 2,60 | 0,0307 | 6,69 |
| 1 | ADO2 | 0,511 | 11,84 | 7,10 | 9,00 | 2,42 | 0,0285 | 5,57 |
| 1 | ADO2 | 0,574 | 13,39 | 7,21 | 7,70 | 2,18 | 0,0310 | 5,40 |
| 1 | ADO2 | 0,457 | 12,61 | 7,49 | 8,09 | 1,95 | 0,0334 | 7,30 |
| 1 | ADO2 | 0,417 | 12,92 | 6,70 | 8,00 | 2,17 | 0,0277 | 6,65 |
| 1 | ADO2 | 0,503 | 11,73 | 8,84 | 8,31 | 2,22 | 0,0244 | 4,85 |
| 1 | ADO2 | 0,466 | 11,37 | 9,28 | 7,14 | 2,10 | 0,0280 | 6,01 |
| 1 | ADO2 | 0,424 | 13,02 | 7,09 | 6,70 | 2,96 | 0,0178 | 4,20 |
| 1 | ADO2 | 0,380 | 13,01 | 6,35 | 7,84 | 2,03 | 0,0185 | 4,87 |
| 1 | ADO2 | 0,312 | 10,45 | 4,86 | 7,48 | 2,40 | 0,0303 | 9,72 |
| 1 | ADO2 | 0,533 | 12,33 | 6,60 | 6,89 | 2,00 | 0,0269 | 5,05 |
| 1 | PG | 0,470 | 10,24 | 7,61 | 7,81 | 2,45 | 0,0371 | 7,90 |
| 1 | PG | 0,582 | 11,78 | 8,68 | 6,72 | 2,46 | 0,0400 | 6,88 |
| 1 | PG | 0,459 | 10,97 | 5,65 | 7,04 | 2,83 | 0,0367 | 7,99 |
| 1 | PG | 0,630 | 10,50 | 6,94 | 7,43 | 3,16 | 0,0337 | 5,35 |
| 1 | PG | 0,575 | 10,48 | 7,67 | 6,88 | 3,36 | 0,0343 | 5,97 |
| 1 | PG | 0,574 | 13,41 | 6,75 | 6,85 | 2,51 | 0,0461 | 8,03 |
| 1 | PG | 0,648 | 11,65 | 9,02 | 6,91 | 2,44 | 0,0444 | 6,85 |
| 1 | PG | 0,613 | 11,21 | 9,43 | 6,48 | 3,01 | 0,0591 | 9,64 |
| 1 | PG | 0,560 | 12,07 | 8,03 | 6,79 | 3,22 | 0,0428 | 7,64 |
| 1 | PG | 0,543 | 11,52 | 7,53 | 7,57 | 2,61 | 0,0474 | 8,73 |
| 1 | PG | 0,443 | 10,85 | 6,89 | 7,97 | 2,26 | 0,0322 | 7,27 |
| 1 | PG | 0,518 | 10,27 | 8,25 | 7,07 | 2,52 | 0,0641 | 12,38 |
| 1 | PG | 0,522 | 10,38 | 10,54 | 6,38 | 2,31 | 0,0231 | 4,43 |
| 1 | PG | 0,446 | 10,91 | 7,82 | 8,09 | 2,43 | 0,0507 | 11,36 |
| 1 | PG | 0,414 | 11,53 | 6,47 | 6,36 | 3,12 | 0,0387 | 9,35 |
| 1 | PG | 0,555 | 11,88 | 8,62 | 6,54 | 1,28 | 0,0466 | 8,39 |
| 1 | PG | 0,464 | 11,48 | 8,50 | 7,00 | 3,00 | 0,0376 | 8,11 |
| 1 | PG | 0,539 | 11,83 | 8,63 | 5,32 | 2,18 | 0,0412 | 7,64 |
| 1 | PG | 0,489 | 11,70 | 7,39 | 7,73 | 1,86 | 0,0327 | 6,68 |
| 1 | PG | 0,627 | 11,40 | 6,31 | 7,25 | 2,28 | 0,0360 | 5,74 |
| 1 | PG | 0,577 | 12,37 | 6,47 | 6,21 | 1,61 | 0,0421 | 7,30 |
| 1 | PG | 0,571 | 11,98 | 7,21 | 5,90 | 2,81 | 0,0572 | 10,01 |
| 1 | PG | 0,520 | 11,07 | 6,10 | 7,33 | 2,87 | 0,0472 | 9,08 |
| 1 | PG | 0,555 | 11,12 | 5,83 | 7,15 | 3,28 | 0,0403 | 7,26 |
| 1 | PG | 0,573 | 11,54 | 7,84 | 7,78 | 2,28 | 0,0432 | 7,54 |
| 2 | ME1 | 0,498 | 12,18 | 10,15 | 7,71 | 2,65 | 0,0328 | 6,58 |
| 2 | ME1 | 0,494 | 12,38 | 9,13 | 5,98 | 2,54 | 0,0262 | 5,30 |
| 2 | ME1 | 0,452 | 11,06 | 7,94 | 5,98 | 2,28 | 0,0329 | 7,28 |
| 2 | ME1 | 0,337 | 11,04 | 6,23 | 6,03 | 2,31 | 0,0260 | 7,72 |
| 2 | ME1 | 0,408 | 12,44 | 7,47 | 6,21 | 3,05 | 0,0426 | 10,45 |
| 2 | ME1 | 0,353 | 12,32 | 7,27 | 6,93 | 1,62 | 0,0236 | 6,68 |
| 2 | ME1 | 0,319 | 11,20 | 6,56 | 6,29 | 2,08 | 0,0343 | 10,75 |
| 2 | ME1 | 0,350 | 10,84 | 8,07 | 6,97 | 2,15 | 0,0283 | 8,09 |
| 2 | ME1 | 0,339 | 11,03 | 7,62 | 7,06 | 2,02 | 0,0332 | 9,80 |
| 2 | ME1 | 0,388 | 11,77 | 6,92 | 7,83 | 2,63 | 0,0334 | 8,61 |
| 2 | ME1 | 0,189 | 9,90 | 5,04 | 5,97 | 2,33 | 0,0234 | 12,36 |
| 2 | ME1 | 0,288 | 9,80 | 5,85 | 6,46 | 2,73 | 0,0287 | 9,97 |
| 2 | ME1 | 0,385 | 11,29 | 7,61 | 5,80 | 2,44 | 0,0375 | 9,75 |
| 2 | ME1 | 0,333 | 10,89 | 6,23 | 5,81 | 3,36 | 0,0335 | 10,07 |
| 2 | ME1 | 0,310 | 10,60 | 7,21 | 6,30 | 2,08 | 0,0267 | 8,61 |
| 2 | ME1 | 0,201 | 11,00 | 5,18 | 6,65 | 2,25 | 0,0303 | 15,10 |
| 2 | ME1 | 0,387 | 10,07 | 7,66 | 5,95 | 2,67 | 0,0340 | 8,80 |
| 2 | ME1 | 0,332 | 10,65 | 6,85 | 5,90 | 2,34 | 0,0329 | 9,91 |
| 2 | ME1 | 0,317 | 10,90 | 6,63 | 6,89 | 1,73 | 0,0277 | 8,74 |
| 2 | ME1 | 0,481 | 11,19 | 7,86 | 6,47 | 2,86 | 0,0316 | 6,58 |
| 2 | ME1 | 0,202 | 10,34 | 3,75 | 7,03 | 1,94 | 0,0369 | 18,23 |
| 2 | ME1 | 0,219 | 9,04 | 5,87 | 6,90 | 2,65 | 0,0359 | 16,38 |
| 2 | ME1 | 0,194 | 10,21 | 4,56 | 6,28 | 2,31 | 0,0325 | 16,75 |
| 2 | ME1 | 0,301 | 9,58 | 7,97 | 5,57 | 2,34 | 0,0318 | 10,56 |
| 2 | ME1 | 0,281 | 11,57 | 6,66 | 7,24 | 1,99 | 0,0288 | 10,27 |
| 2 | ME2 | 0,425 | 11,34 | 6,54 | 6,73 | 2,15 | 0,0250 | 5,89 |
| 2 | ME2 | 0,382 | 11,53 | 8,49 | 6,47 | 2,37 | 0,0261 | 6,84 |
| 2 | ME2 | 0,335 | 9,71 | 7,88 | 6,63 | 2,83 | 0,0325 | 9,71 |
| 2 | ME2 | 0,191 | 10,36 | 7,08 | 5,76 | 2,58 | 0,0268 | 14,01 |
| 2 | ME2 | 0,249 | 9,89 | 4,42 | 6,48 | 2,24 | 0,0266 | 10,67 |
| 2 | ME2 | 0,444 | 9,50 | 5,47 | 5,95 | 2,20 | 0,0260 | 5,85 |
| 2 | ME2 | 0,391 | 10,96 | 6,99 | 6,89 | 2,07 | 0,0302 | 7,73 |
| 2 | ME2 | 0,397 | 11,65 | 7,92 | 7,57 | 2,77 | 0,0406 | 10,22 |
| 2 | ME2 | 0,418 | 11,12 | 6,63 | 6,75 | 2,50 | 0,0322 | 7,70 |
| 2 | ME2 | 0,393 | 11,23 | 8,45 | 7,00 | 2,96 | 0,0315 | 8,02 |
| 2 | ME2 | 0,374 | 10,56 | 7,15 | 5,09 | 3,08 | 0,0269 | 7,20 |
| 2 | ME2 | 0,375 | 10,37 | 7,09 | 6,14 | 2,36 | 0,0300 | 8,01 |
| 2 | ME2 | 0,205 | 11,28 | 6,08 | 7,11 | 2,38 | 0,0346 | 16,90 |
| 2 | ME2 | 0,358 | 8,66 | 4,79 | 5,52 | 2,49 | 0,0367 | 10,25 |
| 2 | ME2 | 0,376 | 8,73 | 7,31 | 6,03 | 2,36 | 0,0314 | 8,34 |
| 2 | ME2 | 0,349 | 11,07 | 7,38 | 6,94 | 2,71 | 0,0364 | 10,44 |
| 2 | ME2 | 0,324 | 11,74 | 5,36 | 6,37 | 2,38 | 0,0272 | 8,40 |
| 2 | ME2 | 0,377 | 9,03 | 7,53 | 5,21 | 2,37 | 0,0279 | 7,40 |
| 2 | ME2 | 0,270 | 11,29 | 6,45 | 7,31 | 2,27 | 0,0376 | 13,92 |
| 2 | ME2 | 0,255 | 8,71 | 6,23 | 6,05 | 2,76 | 0,0282 | 11,08 |
| 2 | ME2 | 0,311 | 9,31 | 5,67 | 7,30 | 2,37 | 0,0259 | 8,34 |
| 2 | ME2 | 0,303 | 11,52 | 8,54 | 7,32 | 2,33 | 0,0310 | 10,24 |
| 2 | ME2 | 0,248 | 9,49 | 6,65 | 6,51 | 2,06 | 0,0244 | 9,85 |
| 2 | ME2 | 0,227 | 10,36 | 6,30 | 6,30 | 1,88 | 0,0298 | 13,11 |
| 2 | ME2 | 0,234 | 9,08 | 5,82 | 6,86 | 2,68 | 0,0272 | 11,65 |
| 2 | ME3 | 0,396 | 11,19 | 7,48 | 6,51 | 2,43 | 0,0324 | 8,18 |
| 2 | ME3 | 0,411 | 11,34 | 7,74 | 5,90 | 2,72 | 0,0280 | 6,82 |
| 2 | ME3 | 0,477 | 12,16 | 8,46 | 6,37 | 3,00 | 0,0320 | 6,71 |
| 2 | ME3 | 0,553 | 11,41 | 9,59 | 7,81 | 3,28 | 0,0409 | 7,40 |
| 2 | ME3 | 0,405 | 11,84 | 8,28 | 5,71 | 2,65 | 0,0306 | 7,56 |
| 2 | ME3 | 0,372 | 10,95 | 6,97 | 5,45 | 2,14 | 0,0304 | 8,17 |
| 2 | ME3 | 0,465 | 10,95 | 8,17 | 6,44 | 1,66 | 0,0317 | 6,81 |
| 2 | ME3 | 0,376 | 11,15 | 5,74 | 5,86 | 2,31 | 0,0235 | 6,25 |
| 2 | ME3 | 0,461 | 11,79 | 7,48 | 5,58 | 2,11 | 0,0317 | 6,87 |
| 2 | ME3 | 0,461 | 11,65 | 7,34 | 6,81 | 2,34 | 0,0335 | 7,27 |
| 2 | ME3 | 0,413 | 10,57 | 7,30 | 6,78 | 2,32 | 0,0298 | 7,22 |
| 2 | ME3 | 0,452 | 12,81 | 8,77 | 7,19 | 2,95 | 0,0384 | 8,50 |
| 2 | ME3 | 0,446 | 12,96 | 6,44 | 7,86 | 2,05 | 0,0335 | 7,50 |
| 2 | ME3 | 0,442 | 11,41 | 7,66 | 6,71 | 2,70 | 0,0314 | 7,10 |
| 2 | ME3 | 0,391 | 10,05 | 6,40 | 6,97 | 2,87 | 0,0312 | 7,99 |
| 2 | ME3 | 0,415 | 11,51 | 7,49 | 6,79 | 2,97 | 0,0348 | 8,40 |
| 2 | ME3 | 0,449 | 11,64 | 8,82 | 6,30 | 2,28 | 0,0286 | 6,37 |
| 2 | ME3 | 0,325 | 11,61 | 7,43 | 6,74 | 2,22 | 0,0304 | 9,36 |
| 2 | ME3 | 0,375 | 9,34 | 7,93 | 7,67 | 2,51 | 0,0354 | 9,45 |
| 2 | ME3 | 0,410 | 11,15 | 7,66 | 6,28 | 2,25 | 0,0254 | 6,20 |
| 2 | ME3 | 0,324 | 10,60 | 6,01 | 6,96 | 2,26 | 0,0343 | 10,60 |
| 2 | ME3 | 0,323 | 10,44 | 7,25 | 6,14 | 2,51 | 0,0276 | 8,54 |
| 2 | ME3 | 0,508 | 11,44 | 9,00 | 6,29 | 3,43 | 0,0386 | 7,61 |
| 2 | ME3 | 0,383 | 7,91 | 7,68 | 5,66 | 4,02 | 0,0378 | 9,86 |
| 2 | ME3 | 0,301 | 11,70 | 5,09 | 7,35 | 2,55 | 0,0343 | 11,39 |
| 2 | ME31 | 0,326 | 11,58 | 8,43 | 7,10 | 1,81 | 0,0170 | 5,22 |
| 2 | ME31 | 0,353 | 11,19 | 7,03 | 7,18 | 2,40 | 0,0354 | 10,04 |
| 2 | ME31 | 0,405 | 12,90 | 6,99 | 8,23 | 2,85 | 0,0471 | 11,64 |
| 2 | ME31 | 0,262 | 7,75 | 6,66 | 5,32 | 2,60 | 0,0270 | 10,31 |
| 2 | ME31 | 0,430 | 9,81 | 9,17 | 5,59 | 2,84 | 0,0352 | 8,19 |
| 2 | ME31 | 0,410 | 11,03 | 6,87 | 6,96 | 2,27 | 0,0320 | 7,80 |
| 2 | ME31 | 0,366 | 10,15 | 7,84 | 6,01 | 2,31 | 0,0251 | 6,86 |
| 2 | ME31 | 0,408 | 11,03 | 9,06 | 5,85 | 3,03 | 0,0304 | 7,45 |
| 2 | ME31 | 0,366 | 9,22 | 8,40 | 6,75 | 3,43 | 0,0320 | 8,75 |
| 2 | ME31 | 0,370 | 10,95 | 7,14 | 6,45 | 2,14 | 0,0313 | 8,47 |
| 2 | ME31 | 0,340 | 8,70 | 8,25 | 6,68 | 3,65 | 0,0361 | 10,62 |
| 2 | ME31 | 0,316 | 10,17 | 6,48 | 6,35 | 3,24 | 0,0203 | 6,43 |
| 2 | ME31 | 0,270 | 10,30 | 6,92 | 6,78 | 3,39 | 0,0417 | 15,47 |
| 2 | ME31 | 0,300 | 9,98 | 5,36 | 6,17 | 3,73 | 0,0390 | 12,98 |
| 2 | ME31 | 0,366 | 11,26 | 7,46 | 6,70 | 2,14 | 0,0431 | 11,79 |
| 2 | ME31 | 0,316 | 11,72 | 6,47 | 7,83 | 2,99 | 0,0464 | 14,68 |
| 2 | ME31 | 0,280 | 10,67 | 6,03 | 6,94 | 3,08 | 0,0362 | 12,91 |
| 2 | ME31 | 0,374 | 11,19 | 8,35 | 6,30 | 2,96 | 0,0292 | 7,80 |
| 2 | ME31 | 0,420 | 11,64 | 7,03 | 8,02 | 3,18 | 0,0508 | 12,11 |
| 2 | ME31 | 0,325 | 11,55 | 5,90 | 6,95 | 3,49 | 0,0443 | 13,65 |
| 2 | ME31 | 0,319 | 9,92 | 4,72 | 6,32 | 2,36 | 0,0364 | 11,42 |
| 2 | ME31 | 0,305 | 9,64 | 6,76 | 6,27 | 3,33 | 0,0340 | 11,14 |
| 2 | ME31 | 0,295 | 10,60 | 5,00 | 7,05 | 3,10 | 0,0371 | 12,58 |
| 2 | ME31 | 0,200 | 7,61 | 7,20 | 6,95 | 2,30 | 0,0374 | 18,69 |
| 2 | ME31 | 0,250 | 10,03 | 5,16 | 7,11 | 3,08 | 0,0414 | 16,54 |
| 2 | ME4 | 0,155 | 6,38 | 6,18 | 5,74 | 2,36 | 0,0220 | 14,18 |
| 2 | ME4 | 0,296 | 8,87 | 7,05 | 7,10 | 2,53 | 0,0415 | 14,02 |
| 2 | ME4 | 0,235 | 8,52 | 6,26 | 5,64 | 2,55 | 0,0237 | 10,07 |
| 2 | ME4 | 0,348 | 10,64 | 7,09 | 6,96 | 2,10 | 0,0496 | 14,27 |
| 2 | ME4 | 0,350 | 10,06 | 7,91 | 6,64 | 1,94 | 0,0379 | 10,83 |
| 2 | ME4 | 0,358 | 9,73 | 7,66 | 5,96 | 2,27 | 0,0356 | 9,94 |
| 2 | ME4 | 0,303 | 9,78 | 6,90 | 6,07 | 3,53 | 0,0517 | 17,08 |
| 2 | ME4 | 0,278 | 9,43 | 5,71 | 6,21 | 3,03 | 0,0432 | 15,53 |
| 2 | ME4 | 0,269 | 9,06 | 6,50 | 6,23 | 2,45 | 0,0452 | 16,82 |
| 2 | ME4 | 0,323 | 10,18 | 7,94 | 4,92 | 1,92 | 0,0322 | 9,97 |
| 2 | ME4 | 0,378 | 10,58 | 4,66 | 6,72 | 1,17 | 0,0512 | 13,55 |
| 2 | ME4 | 0,252 | 8,73 | 5,74 | 5,55 | 2,37 | 0,0498 | 19,77 |
| 2 | ME4 | 0,283 | 10,08 | 6,95 | 6,66 | 2,28 | 0,0372 | 13,17 |
| 2 | ME4 | 0,236 | 9,16 | 5,45 | 6,34 | 2,06 | 0,0285 | 12,08 |
| 2 | ME4 | 0,304 | 10,11 | 7,68 | 6,82 | 1,83 | 0,0332 | 10,93 |
| 2 | ME4 | 0,292 | 9,80 | 6,29 | 6,71 | 2,34 | 0,0545 | 18,70 |
| 2 | ME4 | 0,266 | 9,19 | 5,34 | 5,92 | 2,85 | 0,0438 | 16,45 |
| 2 | ME4 | 0,292 | 9,17 | 6,64 | 7,32 | 3,20 | 0,0453 | 15,50 |
| 2 | ME4 | 0,392 | 10,26 | 7,52 | 5,92 | 2,54 | 0,0434 | 11,06 |
| 2 | ME4 | 0,350 | 10,08 | 7,26 | 5,88 | 2,59 | 0,0301 | 8,60 |
| 2 | ME4 | 0,220 | 10,17 | 5,87 | 6,74 | 2,58 | 0,0301 | 13,71 |
| 2 | ME4 | 0,238 | 10,03 | 5,26 | 6,99 | 2,51 | 0,0395 | 16,61 |
| 2 | ME4 | 0,433 | 11,19 | 5,69 | 6,19 | 2,87 | 0,0466 | 10,77 |
| 2 | ME4 | 0,234 | 8,81 | 7,04 | 5,85 | 2,76 | 0,0315 | 13,47 |
| 2 | ME4 | 0,209 | 9,39 | 5,65 | 6,72 | 3,88 | 0,0372 | 17,78 |
| 2 | ME5 | 0,483 | 12,11 | 7,92 | 7,84 | 3,04 | 0,0596 | 12,34 |
| 2 | ME5 | 0,498 | 11,31 | 8,00 | 8,54 | 3,05 | 0,0837 | 16,81 |
| 2 | ME5 | 0,495 | 11,70 | 7,45 | 8,51 | 2,47 | 0,0784 | 15,83 |
| 2 | ME5 | 0,496 | 13,13 | 8,87 | 6,67 | 2,90 | 0,0897 | 18,08 |
| 2 | ME5 | 0,400 | 10,83 | 6,23 | 5,95 | 1,81 | 0,0621 | 15,53 |
| 2 | ME5 | 0,373 | 12,72 | 6,00 | 6,00 | 2,11 | 0,0586 | 15,73 |
| 2 | ME5 | 0,384 | 10,57 | 7,77 | 7,45 | 2,37 | 0,0557 | 14,50 |
| 2 | ME5 | 0,420 | 12,12 | 8,33 | 8,08 | 2,35 | 0,0715 | 17,03 |
| 2 | ME5 | 0,479 | 12,37 | 6,08 | 6,42 | 2,08 | 0,0983 | 20,50 |
| 2 | ME5 | 0,407 | 10,83 | 8,32 | 6,52 | 2,36 | 0,0536 | 13,17 |
| 2 | ME5 | 0,490 | 12,24 | 9,24 | 8,44 | 2,89 | 0,0589 | 12,03 |
| 2 | ME5 | 0,446 | 10,76 | 7,34 | 7,78 | 2,55 | 0,0864 | 19,39 |
| 2 | ME5 | 0,366 | 11,36 | 7,48 | 6,93 | 1,98 | 0,0470 | 12,86 |
| 2 | ME5 | 0,470 | 12,00 | 7,18 | 7,78 | 3,20 | 0,0705 | 15,00 |
| 2 | ME5 | 0,403 | 11,62 | 7,28 | 6,45 | 2,81 | 0,0433 | 10,74 |
| 2 | ME5 | 0,470 | 13,01 | 7,47 | 6,88 | 2,69 | 0,0775 | 16,49 |
| 2 | ME5 | 0,478 | 12,22 | 7,36 | 7,86 | 2,58 | 0,0526 | 11,00 |
| 2 | ME5 | 0,494 | 12,06 | 8,69 | 7,42 | 2,87 | 0,0598 | 12,11 |
| 2 | ME5 | 0,539 | 11,69 | 8,61 | 7,25 | 2,89 | 0,0678 | 12,58 |
| 2 | ME5 | 0,280 | 9,55 | 7,46 | 6,56 | 2,38 | 0,0283 | 10,10 |
| 2 | ME5 | 0,369 | 11,64 | 7,10 | 6,55 | 2,70 | 0,0495 | 13,41 |
| 2 | ME5 | 0,297 | 11,57 | 6,04 | 7,79 | 1,89 | 0,0477 | 16,08 |
| 2 | ME5 | 0,278 | 9,24 | 5,99 | 6,96 | 2,19 | 0,0503 | 18,10 |
| 2 | ME5 | 0,377 | 11,17 | 8,01 | 7,04 | 2,56 | 0,0686 | 18,20 |
| 2 | ME5 | 0,244 | 9,22 | 5,10 | 8,78 | 2,67 | 0,0437 | 17,94 |
| 2 | ME6 | 0,295 | 10,06 | 7,88 | 5,97 | 2,57 | 0,0356 | 12,07 |
| 2 | ME6 | 0,418 | 10,84 | 6,07 | 7,05 | 3,85 | 0,0552 | 13,21 |
| 2 | ME6 | 0,375 | 10,15 | 7,75 | 5,48 | 2,88 | 0,0491 | 13,09 |
| 2 | ME6 | 0,204 | 10,25 | 7,32 | 6,88 | 2,96 | 0,0425 | 20,83 |
| 2 | ME6 | 0,377 | 9,18 | 5,63 | 6,28 | 4,54 | 0,0476 | 12,63 |
| 2 | ME6 | 0,205 | 11,18 | 7,30 | 5,31 | 2,96 | 0,0273 | 13,32 |
| 2 | ME6 | 0,431 | 8,98 | 7,10 | 7,01 | 4,31 | 0,0488 | 11,32 |
| 2 | ME6 | 0,364 | 9,98 | 7,13 | 7,77 | 3,45 | 0,0568 | 15,60 |
| 2 | ME6 | 0,443 | 11,89 | 9,02 | 6,38 | 3,26 | 0,0487 | 10,99 |
| 2 | ME6 | 0,365 | 12,87 | 9,07 | 6,71 | 3,51 | 0,0654 | 17,92 |
| 2 | ME6 | 0,530 | 11,32 | 7,82 | 6,58 | 3,29 | 0,0526 | 9,92 |
| 2 | ME6 | 0,464 | 10,49 | 6,95 | 7,41 | 3,11 | 0,0611 | 13,17 |
| 2 | ME6 | 0,309 | 11,52 | 7,39 | 7,23 | 3,31 | 0,0401 | 12,98 |
| 2 | ME6 | 0,435 | 9,68 | 11,15 | 6,10 | 2,78 | 0,0565 | 12,99 |
| 2 | ME6 | 0,365 | 10,45 | 8,96 | 6,86 | 2,98 | 0,0487 | 13,34 |
| 2 | ME6 | 0,454 | 10,56 | 7,52 | 6,57 | 3,14 | 0,0543 | 11,96 |
| 2 | ME6 | 0,443 | 11,52 | 7,68 | 6,54 | 2,96 | 0,0453 | 10,23 |
| 2 | ME6 | 0,430 | 10,27 | 8,66 | 5,94 | 2,58 | 0,0587 | 13,65 |
| 2 | ME6 | 0,425 | 10,21 | 8,21 | 6,01 | 3,10 | 0,0496 | 11,67 |
| 2 | ME6 | 0,437 | 12,38 | 8,96 | 7,76 | 2,47 | 0,0521 | 11,92 |
| 2 | ME6 | 0,422 | 11,36 | 6,93 | 7,61 | 2,03 | 0,0563 | 13,34 |
| 2 | ME6 | 0,380 | 10,26 | 7,68 | 7,26 | 3,35 | 0,0496 | 13,05 |
| 2 | ME6 | 0,332 | 12,01 | 8,46 | 7,91 | 3,56 | 0,0496 | 14,94 |
| 2 | ME6 | 0,439 | 11,36 | 6,98 | 6,56 | 2,98 | 0,0486 | 11,07 |
| 2 | ME6 | 0,435 | 10,54 | 7,01 | 6,91 | 3,27 | 0,0531 | 12,21 |
| 2 | ME7 | 0,418 | 10,44 | 6,50 | 5,56 | 1,99 | 0,0581 | 13,90 |
| 2 | ME7 | 0,471 | 10,62 | 6,29 | 6,14 | 1,29 | 0,0521 | 11,06 |
| 2 | ME7 | 0,525 | 9,46 | 7,55 | 5,45 | 3,01 | 0,0728 | 13,87 |
| 2 | ME7 | 0,363 | 9,63 | 5,82 | 4,93 | 1,33 | 0,0541 | 14,90 |
| 2 | ME7 | 0,310 | 10,06 | 6,20 | 6,19 | 1,10 | 0,0535 | 17,26 |
| 2 | ME7 | 0,451 | 10,97 | 8,98 | 6,44 | 1,42 | 0,0579 | 12,84 |
| 2 | ME7 | 0,528 | 11,21 | 7,11 | 6,54 | 2,20 | 0,0786 | 14,89 |
| 2 | ME7 | 0,409 | 9,23 | 7,88 | 5,45 | 2,12 | 0,0630 | 15,40 |
| 2 | ME7 | 0,412 | 9,87 | 7,25 | 6,36 | 2,41 | 0,0460 | 11,17 |
| 2 | ME7 | 0,379 | 9,80 | 7,58 | 8,17 | 1,15 | 0,0738 | 19,47 |
| 2 | ME7 | 0,337 | 9,30 | 6,20 | 5,24 | 1,86 | 0,0624 | 18,52 |
| 2 | ME7 | 0,400 | 6,62 | 6,58 | 5,23 | 2,27 | 0,0591 | 14,78 |
| 2 | ME7 | 0,549 | 8,53 | 6,64 | 5,51 | 1,26 | 0,0846 | 15,41 |
| 2 | ME7 | 0,407 | 10,03 | 7,21 | 5,74 | 1,45 | 0,0630 | 15,48 |
| 2 | ME7 | 0,293 | 9,44 | 5,12 | 4,36 | 0,84 | 0,0411 | 14,03 |
| 2 | ME7 | 0,434 | 10,42 | 7,83 | 4,87 | 1,20 | 0,0562 | 12,95 |
| 2 | ME7 | 0,324 | 7,78 | 6,48 | 5,32 | 1,64 | 0,0431 | 13,30 |
| 2 | ME7 | 0,359 | 9,50 | 6,43 | 5,99 | 1,45 | 0,0644 | 17,94 |
| 2 | ME7 | 0,391 | 9,24 | 6,88 | 6,55 | 2,14 | 0,0774 | 19,80 |
| 2 | ME7 | 0,455 | 11,42 | 9,20 | 6,07 | 2,06 | 0,0416 | 9,14 |
| 2 | ME7 | 0,354 | 10,95 | 7,88 | 5,82 | 1,94 | 0,0535 | 15,11 |
| 2 | ME7 | 0,306 | 10,43 | 5,58 | 6,35 | 1,08 | 0,0495 | 16,18 |
| 2 | ME7 | 0,269 | 7,76 | 4,85 | 5,64 | 1,23 | 0,0434 | 16,13 |
| 2 | ME7 | 0,493 | 8,30 | 6,96 | 4,61 | 1,03 | 0,0391 | 7,93 |
| 2 | ME7 | 0,366 | 9,94 | 7,90 | 5,57 | 1,92 | 0,0541 | 14,78 |
| 2 | ME8 | 0,368 | 8,27 | 8,37 | 6,11 | 2,08 | 0,0641 | 17,42 |
| 2 | ME8 | 0,417 | 9,47 | 5,89 | 5,25 | 2,10 | 0,0678 | 16,26 |
| 2 | ME8 | 0,421 | 11,05 | 7,20 | 6,28 | 1,86 | 0,0694 | 16,48 |
| 2 | ME8 | 0,365 | 8,42 | 7,68 | 8,24 | 1,84 | 0,0567 | 15,53 |
| 2 | ME8 | 0,441 | 10,08 | 6,87 | 6,28 | 1,37 | 0,0631 | 14,31 |
| 2 | ME8 | 0,356 | 7,62 | 5,38 | 4,89 | 2,06 | 0,0657 | 18,46 |
| 2 | ME8 | 0,491 | 9,26 | 7,33 | 4,68 | 1,77 | 0,0362 | 7,37 |
| 2 | ME8 | 0,465 | 10,78 | 7,57 | 5,62 | 1,37 | 0,0629 | 13,53 |
| 2 | ME8 | 0,403 | 9,68 | 3,63 | 5,71 | 1,05 | 0,0359 | 8,91 |
| 2 | ME8 | 0,408 | 9,62 | 5,26 | 5,14 | 2,01 | 0,0526 | 12,89 |
| 2 | ME8 | 0,315 | 7,68 | 5,77 | 5,29 | 0,91 | 0,0476 | 15,11 |
| 2 | ME8 | 0,431 | 9,45 | 4,83 | 5,01 | 1,22 | 0,0574 | 13,32 |
| 2 | ME8 | 0,417 | 10,17 | 5,83 | 6,69 | 2,17 | 0,0544 | 13,05 |
| 2 | ME8 | 0,358 | 9,36 | 4,87 | 6,48 | 1,09 | 0,0551 | 15,39 |
| 2 | ME8 | 0,425 | 9,58 | 7,76 | 6,49 | 2,03 | 0,0463 | 10,89 |
| 2 | ME8 | 0,443 | 11,72 | 6,57 | 6,36 | 1,18 | 0,0717 | 16,19 |
| 2 | ME8 | 0,355 | 9,13 | 4,89 | 4,55 | 3,45 | 0,0335 | 9,44 |
| 2 | ME8 | 0,294 | 8,73 | 4,42 | 5,57 | 1,57 | 0,0463 | 15,75 |
| 2 | ME8 | 0,406 | 9,37 | 4,92 | 5,49 | 3,07 | 0,0423 | 10,42 |
| 2 | ME8 | 0,436 | 7,17 | 5,91 | 5,91 | 1,43 | 0,0646 | 14,82 |
| 2 | ME8 | 0,519 | 10,92 | 6,51 | 5,88 | 1,34 | 0,0595 | 11,46 |
| 2 | ME8 | 0,365 | 9,37 | 4,20 | 5,06 | 1,70 | 0,0530 | 14,52 |
| 2 | ME8 | 0,387 | 8,31 | 5,07 | 4,53 | 2,89 | 0,0620 | 16,02 |
| 2 | ME8 | 0,411 | 8,85 | 5,04 | 3,57 | 0,44 | 0,0388 | 9,44 |
| 2 | ME8 | 0,433 | 9,78 | 5,26 | 4,52 | 2,11 | 0,0574 | 13,26 |
| 2 | ME9 | 0,331 | 7,89 | 3,58 | 5,24 | 1,02 | 0,0387 | 11,69 |
| 2 | ME9 | 0,338 | 9,64 | 4,38 | 8,68 | 0,75 | 0,0421 | 12,46 |
| 2 | ME9 | 0,372 | 6,74 | 5,71 | 5,34 | 1,50 | 0,0426 | 11,45 |
| 2 | ME9 | 0,336 | 9,13 | 5,68 | 6,36 | 1,23 | 0,0368 | 10,95 |
| 2 | ME9 | 0,414 | 8,61 | 7,13 | 7,74 | 1,85 | 0,0428 | 10,34 |
| 2 | ME9 | 0,368 | 8,96 | 5,87 | 7,28 | 0,83 | 0,0518 | 14,08 |
| 2 | ME9 | 0,276 | 8,56 | 5,72 | 7,11 | 1,21 | 0,0443 | 16,05 |
| 2 | ME9 | 0,288 | 8,20 | 6,08 | 6,40 | 0,70 | 0,0454 | 15,76 |
| 2 | ME9 | 0,429 | 9,58 | 5,38 | 5,93 | 0,85 | 0,0293 | 6,83 |
| 2 | ME9 | 0,381 | 8,98 | 6,74 | 4,53 | 1,56 | 0,0285 | 7,48 |
| 2 | ME9 | 0,382 | 9,81 | 4,89 | 5,95 | 0,94 | 0,0367 | 9,61 |
| 2 | ME9 | 0,269 | 8,57 | 4,86 | 8,41 | 1,30 | 0,0316 | 11,75 |
| 2 | ME9 | 0,382 | 9,22 | 4,78 | 5,04 | 0,89 | 0,0416 | 10,89 |
| 2 | ME9 | 0,447 | 9,72 | 6,50 | 6,83 | 1,32 | 0,0384 | 8,59 |
| 2 | ME9 | 0,352 | 8,89 | 5,18 | 8,57 | 0,89 | 0,0586 | 16,65 |
| 2 | ME9 | 0,378 | 8,78 | 5,78 | 3,47 | 0,48 | 0,0566 | 14,97 |
| 2 | ME9 | 0,367 | 8,00 | 4,24 | 5,40 | 0,92 | 0,0516 | 14,06 |
| 2 | ME9 | 0,357 | 8,61 | 7,01 | 6,34 | 0,80 | 0,0570 | 15,97 |
| 2 | ME9 | 0,360 | 8,66 | 7,02 | 4,11 | 1,03 | 0,0609 | 16,92 |
| 2 | ME9 | 0,383 | 10,09 | 6,54 | 5,20 | 0,76 | 0,0447 | 11,67 |
| 2 | ME9 | 0,434 | 5,31 | 8,53 | 5,93 | 0,70 | 0,0353 | 8,13 |
| 2 | ME9 | 0,367 | 8,60 | 5,13 | 6,30 | 0,71 | 0,0544 | 14,82 |
| 2 | ME9 | 0,436 | 8,08 | 4,82 | 4,76 | 0,74 | 0,0539 | 12,36 |
| 2 | ME9 | 0,380 | 9,05 | 5,16 | 4,63 | 0,49 | 0,0669 | 17,61 |
| 2 | ME9 | 0,396 | 7,80 | 6,26 | 7,93 | 0,62 | 0,0727 | 18,36 |
| 2 | ME10 | 0,263 | 8,30 | 4,81 | 5,99 | 0,75 | 0,0310 | 11,79 |
| 2 | ME10 | 0,268 | 8,40 | 4,68 | 3,88 | 0,88 | 0,0314 | 11,72 |
| 2 | ME10 | 0,218 | 6,50 | 3,76 | 3,05 | 0,78 | 0,0216 | 9,91 |
| 2 | ME10 | 0,379 | 8,50 | 5,79 | 6,45 | 1,32 | 0,0730 | 19,26 |
| 2 | ME10 | 0,278 | 8,45 | 4,29 | 4,96 | 1,53 | 0,0485 | 17,45 |
| 2 | ME10 | 0,318 | 7,91 | 3,58 | 5,78 | 1,21 | 0,0593 | 18,65 |
| 2 | ME10 | 0,254 | 8,69 | 2,57 | 4,41 | 0,79 | 0,0498 | 19,61 |
| 2 | ME10 | 0,264 | 6,14 | 3,56 | 2,70 | 1,41 | 0,0499 | 18,90 |
| 2 | ME10 | 0,272 | 9,56 | 5,00 | 4,47 | 1,78 | 0,0484 | 17,79 |
| 2 | ME10 | 0,398 | 6,70 | 4,87 | 5,93 | 1,62 | 0,0456 | 11,46 |
| 2 | ME10 | 0,318 | 7,28 | 4,88 | 6,08 | 1,75 | 0,0403 | 12,67 |
| 2 | ME10 | 0,199 | 10,87 | 4,87 | 5,01 | 1,55 | 0,0242 | 12,16 |
| 2 | ME10 | 0,516 | 9,61 | 4,07 | 4,01 | 1,75 | 0,0537 | 10,41 |
| 2 | ME10 | 0,382 | 7,41 | 4,50 | 5,31 | 1,62 | 0,0335 | 8,77 |
| 2 | ME10 | 0,238 | 8,18 | 5,07 | 6,95 | 1,39 | 0,0421 | 17,69 |
| 2 | ME10 | 0,239 | 7,70 | 4,98 | 5,33 | 0,99 | 0,0420 | 17,57 |
| 2 | ME10 | 0,368 | 9,08 | 4,97 | 7,47 | 2,02 | 0,0685 | 18,61 |
| 2 | ME10 | 0,303 | 9,82 | 5,90 | 4,06 | 2,28 | 0,0339 | 11,19 |
| 2 | ME10 | 0,434 | 9,31 | 3,64 | 6,77 | 2,32 | 0,0425 | 9,79 |
| 2 | ME10 | 0,350 | 8,41 | 4,15 | 3,92 | 0,76 | 0,0351 | 10,03 |
| 2 | ME10 | 0,237 | 9,06 | 3,57 | 4,20 | 0,62 | 0,0359 | 15,15 |
| 2 | ME10 | 0,333 | 10,05 | 4,80 | 4,05 | 0,20 | 0,0455 | 13,66 |
| 2 | ME10 | 0,386 | 8,89 | 4,52 | 4,08 | 0,30 | 0,0523 | 13,55 |
| 2 | ME10 | 0,384 | 7,89 | 3,93 | 5,15 | 1,30 | 0,0428 | 11,15 |
| 2 | ME10 | 0,286 | 7,60 | 2,98 | 6,80 | 2,40 | 0,0495 | 17,31 |
| 2 | ME11 | 0,535 | 11,03 | 5,36 | 6,75 | 0,93 | 0,0598 | 11,18 |
| 2 | ME11 | 0,481 | 9,85 | 5,90 | 4,92 | 1,23 | 0,0823 | 17,11 |
| 2 | ME11 | 0,475 | 10,70 | 4,90 | 7,87 | 1,54 | 0,0729 | 15,35 |
| 2 | ME11 | 0,387 | 10,30 | 5,09 | 6,82 | 1,28 | 0,0721 | 18,63 |
| 2 | ME11 | 0,456 | 9,81 | 4,29 | 4,98 | 1,02 | 0,0666 | 14,61 |
| 2 | ME11 | 0,540 | 9,10 | 5,75 | 5,18 | 2,90 | 0,0703 | 13,02 |
| 2 | ME11 | 0,460 | 10,49 | 7,03 | 6,38 | 0,86 | 0,0696 | 15,13 |
| 2 | ME11 | 0,368 | 9,51 | 4,37 | 4,96 | 0,56 | 0,0646 | 17,55 |
| 2 | ME11 | 0,377 | 8,51 | 4,09 | 4,10 | 1,02 | 0,0503 | 13,34 |
| 2 | ME11 | 0,366 | 7,69 | 4,47 | 7,32 | 0,99 | 0,0413 | 11,28 |
| 2 | ME11 | 0,541 | 9,13 | 7,02 | 4,61 | 0,28 | 0,0568 | 10,50 |
| 2 | ME11 | 0,318 | 7,49 | 5,48 | 5,89 | 1,25 | 0,0462 | 14,53 |
| 2 | ME11 | 0,318 | 7,63 | 3,28 | 5,93 | 0,86 | 0,0371 | 11,67 |
| 2 | ME11 | 0,574 | 8,98 | 3,04 | 3,86 | 0,70 | 0,0548 | 9,55 |
| 2 | ME11 | 0,421 | 8,95 | 2,16 | 2,17 | 0,78 | 0,0653 | 15,51 |
| 2 | ME11 | 0,442 | 8,63 | 6,04 | 5,39 | 2,78 | 0,0530 | 11,99 |
| 2 | ME11 | 0,463 | 9,36 | 4,60 | 2,78 | 1,20 | 0,0593 | 12,81 |
| 2 | ME11 | 0,271 | 8,45 | 5,97 | 5,72 | 2,83 | 0,0441 | 16,27 |
| 2 | ME11 | 0,303 | 7,68 | 6,68 | 2,10 | 0,83 | 0,0597 | 19,70 |
| 2 | ME11 | 0,405 | 6,78 | 3,87 | 4,61 | 1,84 | 0,0481 | 11,88 |
| 2 | ME11 | 0,462 | 8,18 | 3,56 | 5,15 | 0,87 | 0,0509 | 11,02 |
| 2 | ME11 | 0,417 | 10,79 | 5,46 | 5,82 | 2,02 | 0,0517 | 12,40 |
| 2 | ME11 | 0,503 | 6,60 | 4,90 | 8,05 | 1,42 | 0,0516 | 10,26 |
| 2 | ME11 | 0,515 | 7,90 | 5,50 | 3,73 | 0,85 | 0,0416 | 8,08 |
| 2 | ME11 | 0,489 | 11,43 | 5,48 | 4,92 | 0,67 | 0,0693 | 14,17 |
| 2 | ME12 | 0,315 | 9,36 | 4,48 | 5,29 | 0,94 | 0,0250 | 7,93 |
| 2 | ME12 | 0,450 | 11,20 | 5,50 | 5,62 | 0,86 | 0,0247 | 5,49 |
| 2 | ME12 | 0,460 | 10,72 | 6,60 | 4,60 | 0,90 | 0,0286 | 6,22 |
| 2 | ME12 | 0,482 | 10,54 | 6,14 | 4,94 | 1,14 | 0,0345 | 7,17 |
| 2 | ME12 | 0,246 | 8,49 | 3,25 | 4,72 | 0,78 | 0,0299 | 12,18 |
| 2 | ME12 | 0,391 | 9,90 | 6,10 | 4,63 | 0,84 | 0,0260 | 6,64 |
| 2 | ME12 | 0,419 | 10,68 | 4,44 | 5,67 | 0,71 | 0,0307 | 7,33 |
| 2 | ME12 | 0,389 | 9,06 | 4,67 | 5,71 | 0,79 | 0,0366 | 9,41 |
| 2 | ME12 | 0,434 | 9,50 | 5,90 | 5,21 | 1,08 | 0,0284 | 6,55 |
| 2 | ME12 | 0,465 | 10,08 | 4,85 | 6,27 | 0,81 | 0,0311 | 6,69 |
| 2 | ME12 | 0,445 | 11,27 | 5,91 | 5,49 | 1,27 | 0,0404 | 9,09 |
| 2 | ME12 | 0,385 | 8,88 | 6,17 | 5,32 | 1,25 | 0,0370 | 9,61 |
| 2 | ME12 | 0,283 | 7,76 | 4,99 | 4,04 | 0,90 | 0,0354 | 12,53 |
| 2 | ME12 | 0,453 | 9,60 | 5,15 | 5,30 | 1,27 | 0,0405 | 8,94 |
| 2 | ME12 | 0,477 | 11,42 | 4,88 | 5,62 | 0,93 | 0,0355 | 7,44 |
| 2 | ME12 | 0,306 | 7,64 | 3,53 | 3,94 | 0,84 | 0,0335 | 10,93 |
| 2 | ME12 | 0,461 | 9,53 | 5,61 | 5,91 | 0,80 | 0,0333 | 7,22 |
| 2 | ME12 | 0,385 | 8,41 | 4,38 | 5,69 | 0,93 | 0,0409 | 10,62 |
| 2 | ME12 | 0,339 | 7,94 | 4,48 | 5,73 | 0,92 | 0,0534 | 15,75 |
| 2 | ME12 | 0,391 | 6,57 | 5,19 | 4,20 | 0,81 | 0,0321 | 8,21 |
| 2 | ME12 | 0,365 | 9,97 | 4,53 | 5,23 | 0,91 | 0,0354 | 9,71 |
| 2 | ME12 | 0,320 | 8,10 | 5,07 | 5,82 | 0,86 | 0,0423 | 13,23 |
| 2 | ME12 | 0,422 | 9,96 | 5,14 | 5,16 | 0,90 | 0,0409 | 9,70 |
| 2 | ME12 | 0,332 | 10,12 | 4,23 | 5,24 | 0,85 | 0,0351 | 10,56 |
| 2 | ME12 | 0,510 | 10,16 | 5,51 | 6,13 | 1,77 | 0,0402 | 7,88 |
| 2 | ME13 | 0,394 | 10,05 | 4,99 | 5,51 | 0,91 | 0,0296 | 7,51 |
| 2 | ME13 | 0,386 | 10,42 | 4,42 | 5,55 | 0,83 | 0,0308 | 7,99 |
| 2 | ME13 | 0,434 | 10,46 | 4,50 | 6,01 | 0,63 | 0,0275 | 6,34 |
| 2 | ME13 | 0,375 | 9,23 | 4,91 | 4,80 | 0,85 | 0,0440 | 11,75 |
| 2 | ME13 | 0,413 | 10,75 | 6,10 | 6,18 | 0,83 | 0,0408 | 9,88 |
| 2 | ME13 | 0,334 | 8,96 | 5,31 | 5,75 | 0,87 | 0,0414 | 12,41 |
| 2 | ME13 | 0,262 | 9,63 | 3,97 | 5,47 | 1,40 | 0,0386 | 14,71 |
| 2 | ME13 | 0,321 | 8,52 | 3,75 | 4,91 | 0,73 | 0,0271 | 8,44 |
| 2 | ME13 | 0,303 | 9,31 | 5,73 | 4,52 | 0,88 | 0,0339 | 11,17 |
| 2 | ME13 | 0,365 | 9,14 | 5,93 | 4,87 | 0,93 | 0,0370 | 10,15 |
| 2 | ME13 | 0,342 | 10,13 | 4,50 | 5,93 | 0,71 | 0,0415 | 12,12 |
| 2 | ME13 | 0,301 | 9,54 | 3,90 | 5,49 | 0,82 | 0,0273 | 9,07 |
| 2 | ME13 | 0,288 | 6,67 | 2,99 | 5,00 | 1,36 | 0,0284 | 9,88 |
| 2 | ME13 | 0,393 | 8,36 | 5,91 | 4,51 | 0,77 | 0,0289 | 7,36 |
| 2 | ME13 | 0,439 | 10,13 | 6,04 | 4,67 | 0,84 | 0,0319 | 7,26 |
| 2 | ME13 | 0,535 | 9,93 | 6,42 | 5,51 | 1,08 | 0,0440 | 8,23 |
| 2 | ME13 | 0,353 | 10,89 | 4,84 | 4,85 | 0,81 | 0,0348 | 9,86 |
| 2 | ME13 | 0,381 | 10,10 | 5,15 | 5,46 | 0,73 | 0,0395 | 10,38 |
| 2 | ME13 | 0,347 | 9,52 | 5,61 | 5,08 | 0,94 | 0,0373 | 10,75 |
| 2 | ME13 | 0,346 | 10,03 | 3,58 | 5,67 | 0,80 | 0,0422 | 12,19 |
| 2 | ME13 | 0,453 | 9,79 | 5,52 | 5,05 | 0,86 | 0,0382 | 8,44 |
| 2 | ME13 | 0,358 | 9,58 | 4,77 | 5,47 | 0,87 | 0,0423 | 11,82 |
| 2 | ME13 | 0,234 | 6,74 | 3,78 | 5,48 | 0,61 | 0,0263 | 11,22 |
| 2 | ME13 | 0,250 | 8,01 | 3,62 | 4,58 | 0,60 | 0,0222 | 8,89 |
| 2 | ME13 | 0,445 | 9,09 | 6,76 | 5,62 | 1,41 | 0,0331 | 7,44 |
| 2 | ME14 | 0,399 | 11,49 | 6,47 | 5,96 | 1,96 | 0,0364 | 9,12 |
| 2 | ME14 | 0,218 | 8,20 | 5,88 | 6,78 | 1,87 | 0,0286 | 13,12 |
| 2 | ME14 | 0,357 | 10,13 | 6,03 | 6,81 | 1,56 | 0,0358 | 10,03 |
| 2 | ME14 | 0,232 | 12,23 | 4,53 | 5,65 | 2,34 | 0,0234 | 10,09 |
| 2 | ME14 | 0,193 | 9,92 | 5,19 | 6,09 | 1,61 | 0,0354 | 18,34 |
| 2 | ME14 | 0,351 | 7,64 | 6,60 | 6,64 | 2,44 | 0,0363 | 10,34 |
| 2 | ME14 | 0,377 | 10,42 | 5,52 | 6,60 | 1,72 | 0,0298 | 7,90 |
| 2 | ME14 | 0,342 | 11,12 | 6,78 | 6,38 | 2,06 | 0,0320 | 9,36 |
| 2 | ME14 | 0,295 | 9,39 | 6,25 | 6,44 | 2,25 | 0,0268 | 9,08 |
| 2 | ME14 | 0,263 | 8,80 | 5,43 | 5,76 | 1,16 | 0,0249 | 9,47 |
| 2 | ME14 | 0,292 | 10,53 | 6,50 | 4,42 | 2,81 | 0,0252 | 8,63 |
| 2 | ME14 | 0,319 | 9,50 | 5,50 | 5,37 | 1,45 | 0,0247 | 7,74 |
| 2 | ME14 | 0,341 | 10,88 | 6,53 | 6,17 | 2,21 | 0,0287 | 8,42 |
| 2 | ME14 | 0,351 | 10,88 | 7,23 | 6,53 | 2,55 | 0,0343 | 9,77 |
| 2 | ME14 | 0,319 | 10,62 | 4,34 | 6,90 | 1,14 | 0,0263 | 8,24 |
| 2 | ME14 | 0,400 | 11,18 | 6,21 | 6,87 | 1,99 | 0,0287 | 7,18 |
| 2 | ME14 | 0,371 | 11,17 | 7,46 | 6,15 | 1,49 | 0,0322 | 8,68 |
| 2 | ME14 | 0,373 | 10,46 | 6,32 | 6,94 | 2,50 | 0,0437 | 11,72 |
| 2 | ME14 | 0,327 | 10,95 | 4,76 | 8,13 | 1,57 | 0,0288 | 8,81 |
| 2 | ME14 | 0,384 | 12,52 | 5,92 | 6,38 | 1,76 | 0,0326 | 8,49 |
| 2 | ME14 | 0,405 | 12,65 | 5,80 | 7,79 | 2,32 | 0,0322 | 7,95 |
| 2 | ME14 | 0,387 | 9,96 | 7,96 | 5,39 | 1,84 | 0,0335 | 8,66 |
| 2 | ME14 | 0,319 | 9,54 | 6,82 | 5,31 | 1,65 | 0,0448 | 14,04 |
| 2 | ME14 | 0,337 | 10,29 | 5,82 | 7,79 | 1,45 | 0,0278 | 8,25 |
| 2 | ME14 | 0,367 | 11,83 | 7,31 | 7,00 | 1,59 | 0,0438 | 11,93 |
| 2 | ME16 | 0,413 | 9,35 | 6,56 | 4,52 | 1,75 | 0,0251 | 6,07 |
| 2 | ME16 | 0,399 | 9,70 | 6,61 | 5,76 | 2,07 | 0,0314 | 7,88 |
| 2 | ME16 | 0,417 | 9,72 | 7,81 | 5,91 | 1,67 | 0,0258 | 6,19 |
| 2 | ME16 | 0,394 | 10,43 | 5,18 | 5,69 | 1,56 | 0,0339 | 8,61 |
| 2 | ME16 | 0,463 | 11,09 | 7,19 | 5,48 | 1,13 | 0,0306 | 6,61 |
| 2 | ME16 | 0,481 | 10,80 | 6,31 | 5,93 | 1,95 | 0,0302 | 6,28 |
| 2 | ME16 | 0,437 | 11,69 | 6,58 | 6,76 | 1,16 | 0,0244 | 5,58 |
| 2 | ME16 | 0,467 | 10,69 | 6,79 | 4,98 | 1,72 | 0,0263 | 5,63 |
| 2 | ME16 | 0,549 | 10,77 | 6,11 | 5,21 | 2,71 | 0,0329 | 5,99 |
| 2 | ME16 | 0,463 | 11,65 | 6,94 | 5,81 | 2,02 | 0,0330 | 7,13 |
| 2 | ME16 | 0,509 | 10,36 | 6,98 | 5,25 | 1,69 | 0,0368 | 7,24 |
| 2 | ME16 | 0,406 | 10,43 | 5,84 | 4,96 | 1,35 | 0,0281 | 6,92 |
| 2 | ME16 | 0,352 | 8,70 | 5,65 | 5,61 | 1,58 | 0,0366 | 10,39 |
| 2 | ME16 | 0,354 | 9,40 | 5,16 | 5,23 | 2,07 | 0,0351 | 9,90 |
| 2 | ME16 | 0,447 | 10,00 | 6,17 | 5,19 | 2,18 | 0,0400 | 8,95 |
| 2 | ME16 | 0,358 | 11,03 | 6,22 | 5,94 | 1,86 | 0,0299 | 8,35 |
| 2 | ME16 | 0,259 | 7,14 | 5,54 | 5,19 | 1,90 | 0,0253 | 9,78 |
| 2 | ME16 | 0,351 | 10,34 | 5,09 | 6,20 | 1,64 | 0,0336 | 9,57 |
| 2 | ME16 | 0,460 | 9,97 | 6,53 | 4,74 | 1,94 | 0,0334 | 7,25 |
| 2 | ME16 | 0,233 | 8,41 | 4,84 | 6,52 | 1,43 | 0,0365 | 15,65 |
| 2 | ME16 | 0,366 | 10,06 | 5,26 | 6,02 | 1,64 | 0,0290 | 7,93 |
| 2 | ME16 | 0,366 | 9,73 | 5,49 | 6,29 | 2,25 | 0,0321 | 8,78 |
| 2 | ME16 | 0,375 | 9,89 | 5,55 | 5,52 | 1,71 | 0,0256 | 6,83 |
| 2 | ME16 | 0,467 | 11,67 | 7,60 | 5,04 | 2,02 | 0,0302 | 6,46 |
| 2 | ME16 | 0,404 | 8,91 | 4,87 | 4,56 | 2,75 | 0,0307 | 7,61 |
| 2 | ME17 | 0,513 | 11,85 | 7,29 | 6,86 | 2,58 | 0,0540 | 10,53 |
| 2 | ME17 | 0,438 | 12,48 | 8,84 | 6,53 | 3,07 | 0,0345 | 7,88 |
| 2 | ME17 | 0,577 | 13,92 | 8,98 | 9,00 | 1,92 | 0,0612 | 10,61 |
| 2 | ME17 | 0,446 | 12,34 | 6,78 | 8,38 | 2,27 | 0,0501 | 11,23 |
| 2 | ME17 | 0,352 | 11,36 | 6,54 | 6,58 | 2,33 | 0,0364 | 10,34 |
| 2 | ME17 | 0,569 | 12,64 | 8,17 | 7,82 | 1,97 | 0,0441 | 7,75 |
| 2 | ME17 | 0,359 | 11,43 | 7,41 | 7,10 | 1,80 | 0,0395 | 11,00 |
| 2 | ME17 | 0,382 | 12,84 | 7,81 | 7,37 | 2,42 | 0,0466 | 12,20 |
| 2 | ME17 | 0,342 | 10,42 | 7,08 | 6,07 | 1,89 | 0,0367 | 10,73 |
| 2 | ME17 | 0,491 | 13,95 | 7,91 | 8,12 | 1,60 | 0,0378 | 7,70 |
| 2 | ME17 | 0,524 | 12,78 | 6,98 | 6,88 | 1,75 | 0,0414 | 7,90 |
| 2 | ME17 | 0,502 | 12,49 | 5,99 | 7,55 | 1,87 | 0,0396 | 7,89 |
| 2 | ME17 | 0,439 | 12,18 | 7,67 | 6,10 | 1,70 | 0,0434 | 9,89 |
| 2 | ME17 | 0,428 | 13,66 | 6,41 | 7,88 | 1,12 | 0,0406 | 9,49 |
| 2 | ME17 | 0,576 | 10,85 | 5,82 | 6,64 | 1,85 | 0,0264 | 4,58 |
| 2 | ME17 | 0,417 | 10,62 | 8,06 | 5,93 | 1,31 | 0,0299 | 7,17 |
| 2 | ME17 | 0,420 | 13,23 | 9,58 | 7,00 | 1,60 | 0,0387 | 9,21 |
| 2 | ME17 | 0,465 | 12,47 | 9,82 | 6,40 | 1,88 | 0,0324 | 6,97 |
| 2 | ME17 | 0,525 | 13,27 | 8,16 | 7,88 | 1,28 | 0,0550 | 10,48 |
| 2 | ME17 | 0,543 | 12,48 | 7,26 | 5,84 | 1,74 | 0,0417 | 7,68 |
| 2 | ME17 | 0,499 | 12,36 | 9,34 | 6,46 | 1,87 | 0,0437 | 8,76 |
| 2 | ME17 | 0,479 | 13,42 | 8,28 | 6,26 | 0,99 | 0,0431 | 9,00 |
| 2 | ME17 | 0,473 | 11,95 | 8,13 | 6,43 | 1,39 | 0,0363 | 7,67 |
| 2 | ME17 | 0,358 | 11,00 | 8,58 | 6,25 | 1,35 | 0,0291 | 8,13 |
| 2 | ME17 | 0,537 | 11,47 | 8,60 | 5,94 | 1,74 | 0,0351 | 6,54 |
| 2 | ME18 | 0,328 | 10,25 | 5,08 | 4,68 | 0,81 | 0,0327 | 9,98 |
| 2 | ME18 | 0,294 | 9,86 | 4,42 | 4,50 | 0,86 | 0,0272 | 9,25 |
| 2 | ME18 | 0,332 | 9,50 | 3,32 | 5,23 | 1,33 | 0,0410 | 12,36 |
| 2 | ME18 | 0,396 | 9,36 | 5,40 | 5,33 | 1,69 | 0,0342 | 8,63 |
| 2 | ME18 | 0,346 | 9,27 | 4,21 | 6,77 | 0,88 | 0,0384 | 11,10 |
| 2 | ME18 | 0,488 | 10,42 | 4,83 | 4,13 | 1,08 | 0,0465 | 9,53 |
| 2 | ME18 | 0,469 | 10,26 | 5,79 | 5,68 | 1,57 | 0,0486 | 10,35 |
| 2 | ME18 | 0,303 | 7,95 | 4,90 | 4,28 | 1,79 | 0,0398 | 13,16 |
| 2 | ME18 | 0,369 | 9,70 | 4,14 | 5,91 | 0,77 | 0,0331 | 8,98 |
| 2 | ME18 | 0,325 | 9,10 | 5,16 | 3,56 | 0,46 | 0,0371 | 11,41 |
| 2 | ME18 | 0,284 | 8,35 | 3,23 | 4,29 | 0,90 | 0,0331 | 11,66 |
| 2 | ME18 | 0,289 | 8,47 | 3,66 | 3,08 | 0,92 | 0,0326 | 11,27 |
| 2 | ME18 | 0,390 | 9,60 | 6,18 | 4,92 | 0,91 | 0,0446 | 11,43 |
| 2 | ME18 | 0,393 | 9,47 | 5,43 | 5,46 | 1,07 | 0,0445 | 11,31 |
| 2 | ME18 | 0,254 | 8,91 | 2,59 | 4,85 | 0,97 | 0,0346 | 13,61 |
| 2 | ME18 | 0,389 | 7,86 | 5,19 | 3,45 | 1,23 | 0,0222 | 5,71 |
| 2 | ME18 | 0,408 | 8,47 | 5,58 | 4,01 | 0,94 | 0,0222 | 5,44 |
| 2 | ME18 | 0,323 | 8,83 | 4,47 | 5,51 | 0,79 | 0,0241 | 7,47 |
| 2 | ME18 | 0,310 | 8,14 | 2,87 | 5,27 | 0,76 | 0,0374 | 12,05 |
| 2 | ME18 | 0,452 | 9,50 | 5,15 | 5,28 | 0,68 | 0,0424 | 9,37 |
| 2 | ME18 | 0,352 | 9,60 | 3,68 | 5,77 | 0,88 | 0,0364 | 10,33 |
| 2 | ME18 | 0,327 | 9,12 | 4,86 | 5,61 | 0,72 | 0,0345 | 10,55 |
| 2 | ME18 | 0,375 | 9,37 | 5,00 | 4,98 | 0,91 | 0,0341 | 9,09 |
| 2 | ME18 | 0,227 | 8,72 | 3,61 | 4,26 | 0,76 | 0,0301 | 13,24 |
| 2 | ME18 | 0,339 | 9,04 | 4,28 | 4,05 | 0,77 | 0,0308 | 9,09 |
| 2 | ME19 | 0,429 | 10,20 | 5,54 | 6,48 | 0,97 | 0,0303 | 7,06 |
| 2 | ME19 | 0,348 | 9,50 | 5,13 | 6,95 | 1,82 | 0,0244 | 7,01 |
| 2 | ME19 | 0,341 | 10,02 | 4,12 | 6,73 | 1,41 | 0,0286 | 8,39 |
| 2 | ME19 | 0,407 | 11,06 | 5,72 | 7,02 | 1,99 | 0,0312 | 7,67 |
| 2 | ME19 | 0,232 | 9,74 | 5,63 | 7,16 | 1,12 | 0,0355 | 15,32 |
| 2 | ME19 | 0,337 | 8,14 | 3,73 | 5,91 | 0,66 | 0,0294 | 8,71 |
| 2 | ME19 | 0,441 | 9,86 | 5,58 | 6,85 | 2,54 | 0,0496 | 11,24 |
| 2 | ME19 | 0,409 | 9,51 | 5,13 | 6,78 | 3,44 | 0,0650 | 15,90 |
| 2 | ME19 | 0,356 | 10,36 | 5,34 | 7,04 | 2,17 | 0,0427 | 11,98 |
| 2 | ME19 | 0,373 | 12,12 | 5,69 | 8,63 | 1,30 | 0,0311 | 8,34 |
| 2 | ME19 | 0,320 | 11,13 | 6,09 | 6,84 | 2,01 | 0,0469 | 14,64 |
| 2 | ME19 | 0,266 | 10,56 | 4,49 | 5,72 | 1,53 | 0,0490 | 18,44 |
| 2 | ME19 | 0,327 | 9,02 | 6,22 | 4,68 | 1,21 | 0,0610 | 18,65 |
| 2 | ME19 | 0,347 | 11,60 | 5,14 | 7,25 | 2,08 | 0,0541 | 15,60 |
| 2 | ME19 | 0,347 | 8,27 | 6,14 | 7,14 | 2,05 | 0,0455 | 13,13 |
| 2 | ME19 | 0,444 | 11,44 | 5,47 | 5,60 | 2,28 | 0,0395 | 8,91 |
| 2 | ME19 | 0,297 | 7,52 | 6,89 | 6,65 | 1,98 | 0,0391 | 13,19 |
| 2 | ME19 | 0,347 | 9,90 | 3,85 | 7,26 | 1,93 | 0,0521 | 15,02 |
| 2 | ME19 | 0,369 | 11,63 | 5,11 | 7,67 | 1,31 | 0,0571 | 15,49 |
| 2 | ME19 | 0,370 | 10,67 | 6,11 | 7,32 | 1,65 | 0,0544 | 14,71 |
| 2 | ME19 | 0,335 | 10,90 | 6,84 | 5,80 | 2,06 | 0,0351 | 10,49 |
| 2 | ME19 | 0,391 | 10,95 | 5,80 | 7,19 | 2,63 | 0,0426 | 10,89 |
| 2 | ME19 | 0,272 | 10,86 | 5,53 | 5,67 | 1,55 | 0,0435 | 15,97 |
| 2 | ME19 | 0,336 | 11,16 | 5,58 | 6,17 | 1,45 | 0,0431 | 12,85 |
| 2 | ME19 | 0,357 | 10,52 | 6,08 | 5,84 | 1,53 | 0,0356 | 9,98 |
| 2 | ME20 | 0,456 | 9,74 | 5,50 | 5,64 | 0,86 | 0,0312 | 6,84 |
| 2 | ME20 | 0,451 | 11,37 | 6,53 | 4,80 | 1,17 | 0,0336 | 7,45 |
| 2 | ME20 | 0,434 | 10,01 | 7,65 | 4,43 | 0,66 | 0,0289 | 6,66 |
| 2 | ME20 | 0,345 | 10,01 | 6,35 | 4,01 | 0,74 | 0,0259 | 7,51 |
| 2 | ME20 | 0,322 | 7,73 | 6,22 | 3,94 | 0,73 | 0,0189 | 5,87 |
| 2 | ME20 | 0,272 | 9,37 | 5,07 | 3,61 | 1,55 | 0,0181 | 6,65 |
| 2 | ME20 | 0,336 | 8,93 | 6,72 | 5,21 | 1,55 | 0,0247 | 7,36 |
| 2 | ME20 | 0,469 | 10,05 | 6,80 | 5,08 | 0,65 | 0,0296 | 6,32 |
| 2 | ME20 | 0,567 | 9,81 | 9,13 | 5,54 | 1,36 | 0,0343 | 6,05 |
| 2 | ME20 | 0,341 | 8,00 | 6,26 | 4,15 | 0,65 | 0,0219 | 6,42 |
| 2 | ME20 | 0,385 | 9,49 | 7,48 | 5,71 | 0,94 | 0,0254 | 6,60 |
| 2 | ME20 | 0,441 | 9,38 | 7,29 | 3,88 | 1,43 | 0,0270 | 6,12 |
| 2 | ME20 | 0,291 | 9,12 | 6,55 | 4,53 | 0,65 | 0,0268 | 9,21 |
| 2 | ME20 | 0,383 | 8,29 | 7,61 | 3,18 | 1,51 | 0,0231 | 6,04 |
| 2 | ME20 | 0,442 | 11,71 | 6,93 | 5,47 | 0,73 | 0,0284 | 6,42 |
| 2 | ME20 | 0,447 | 10,44 | 9,33 | 4,95 | 1,08 | 0,0359 | 8,03 |
| 2 | ME20 | 0,359 | 9,39 | 7,04 | 4,33 | 1,54 | 0,0332 | 9,24 |
| 2 | ME20 | 0,352 | 8,96 | 7,34 | 4,85 | 1,82 | 0,0353 | 10,04 |
| 2 | ME20 | 0,437 | 9,15 | 6,32 | 4,81 | 1,48 | 0,0358 | 8,19 |
| 2 | ME20 | 0,475 | 10,26 | 7,16 | 4,35 | 0,95 | 0,0312 | 6,57 |
| 2 | ME20 | 0,402 | 8,69 | 8,10 | 5,67 | 1,36 | 0,0311 | 7,73 |
| 2 | ME20 | 0,415 | 11,01 | 7,02 | 5,20 | 0,92 | 0,0239 | 5,76 |
| 2 | ME20 | 0,409 | 8,63 | 6,40 | 5,17 | 1,08 | 0,0324 | 7,93 |
| 2 | ME20 | 0,333 | 7,70 | 6,30 | 3,75 | 0,71 | 0,0244 | 7,33 |
| 2 | ME20 | 0,364 | 9,31 | 5,14 | 5,36 | 0,79 | 0,0259 | 7,12 |
| 2 | ME21 | 0,428 | 9,90 | 7,35 | 4,50 | 1,30 | 0,0261 | 6,10 |
| 2 | ME21 | 0,416 | 10,09 | 6,36 | 5,39 | 1,62 | 0,0301 | 7,24 |
| 2 | ME21 | 0,475 | 9,21 | 7,60 | 5,65 | 1,37 | 0,0349 | 7,34 |
| 2 | ME21 | 0,434 | 8,71 | 6,32 | 3,92 | 1,59 | 0,0269 | 6,20 |
| 2 | ME21 | 0,527 | 11,55 | 6,87 | 6,47 | 1,42 | 0,0284 | 5,39 |
| 2 | ME21 | 0,293 | 9,73 | 5,38 | 5,58 | 1,30 | 0,0324 | 11,08 |
| 2 | ME21 | 0,320 | 8,43 | 6,00 | 4,63 | 1,89 | 0,0267 | 8,35 |
| 2 | ME21 | 0,339 | 8,81 | 7,35 | 4,92 | 1,68 | 0,0348 | 10,26 |
| 2 | ME21 | 0,328 | 7,86 | 6,50 | 5,03 | 2,23 | 0,0347 | 10,59 |
| 2 | ME21 | 0,466 | 9,91 | 5,59 | 5,65 | 1,28 | 0,0359 | 7,70 |
| 2 | ME21 | 0,263 | 8,09 | 4,92 | 5,54 | 0,92 | 0,0353 | 13,40 |
| 2 | ME21 | 0,334 | 10,52 | 5,95 | 4,93 | 1,73 | 0,0333 | 9,97 |
| 2 | ME21 | 0,527 | 8,87 | 6,90 | 6,74 | 2,40 | 0,0423 | 8,02 |
| 2 | ME21 | 0,262 | 8,72 | 4,66 | 5,39 | 1,57 | 0,0275 | 10,49 |
| 2 | ME21 | 0,339 | 8,15 | 6,41 | 5,50 | 2,31 | 0,0408 | 12,03 |
| 2 | ME21 | 0,402 | 9,71 | 7,99 | 4,88 | 2,13 | 0,0346 | 8,60 |
| 2 | ME21 | 0,255 | 8,47 | 4,49 | 5,22 | 1,22 | 0,0368 | 14,43 |
| 2 | ME21 | 0,435 | 10,14 | 7,48 | 5,56 | 1,85 | 0,0419 | 9,63 |
| 2 | ME21 | 0,431 | 10,34 | 5,56 | 5,38 | 1,33 | 0,0309 | 7,18 |
| 2 | ME21 | 0,300 | 8,12 | 6,74 | 3,99 | 1,45 | 0,0322 | 10,72 |
| 2 | ME21 | 0,341 | 10,23 | 7,15 | 5,60 | 2,33 | 0,0270 | 7,93 |
| 2 | ME21 | 0,268 | 8,59 | 5,67 | 5,84 | 1,35 | 0,0332 | 12,37 |
| 2 | ME21 | 0,427 | 8,63 | 6,83 | 3,99 | 1,48 | 0,0258 | 6,04 |
| 2 | ME21 | 0,342 | 8,76 | 6,42 | 4,51 | 1,03 | 0,0239 | 6,99 |
| 2 | ME21 | 0,311 | 10,32 | 5,85 | 5,77 | 1,92 | 0,0327 | 10,53 |
| 2 | MA1 | 0,414 | 10,84 | 6,43 | 7,07 | 1,31 | 0,0309 | 7,46 |
| 2 | MA1 | 0,523 | 9,50 | 7,50 | 7,27 | 3,03 | 0,0560 | 10,71 |
| 2 | MA1 | 0,382 | 8,39 | 3,96 | 6,33 | 1,81 | 0,0620 | 16,23 |
| 2 | MA1 | 0,424 | 8,17 | 3,81 | 6,61 | 1,76 | 0,0360 | 8,49 |
| 2 | MA1 | 0,385 | 11,07 | 4,85 | 7,30 | 2,39 | 0,0357 | 9,27 |
| 2 | MA1 | 0,605 | 9,81 | 5,61 | 6,18 | 1,96 | 0,0632 | 10,45 |
| 2 | MA1 | 0,464 | 10,46 | 5,99 | 6,66 | 1,97 | 0,0770 | 16,59 |
| 2 | MA1 | 0,434 | 8,00 | 8,41 | 6,19 | 2,18 | 0,0570 | 13,13 |
| 2 | MA1 | 0,272 | 8,15 | 7,46 | 7,02 | 2,11 | 0,0535 | 19,67 |
| 2 | MA1 | 0,334 | 9,71 | 6,45 | 5,24 | 1,46 | 0,0650 | 19,46 |
| 2 | MA1 | 0,403 | 8,72 | 5,57 | 6,47 | 2,58 | 0,0670 | 16,63 |
| 2 | MA1 | 0,638 | 11,80 | 8,59 | 8,23 | 2,78 | 0,0580 | 9,09 |
| 2 | MA1 | 0,664 | 10,70 | 6,20 | 6,57 | 2,04 | 0,0450 | 6,78 |
| 2 | MA1 | 0,504 | 10,40 | 5,87 | 8,29 | 1,16 | 0,0510 | 10,12 |
| 2 | MA1 | 0,366 | 10,40 | 4,48 | 6,27 | 2,84 | 0,0560 | 15,30 |
| 2 | MA1 | 0,434 | 10,84 | 5,40 | 7,39 | 1,93 | 0,0740 | 17,05 |
| 2 | MA1 | 0,540 | 8,70 | 6,30 | 6,07 | 1,89 | 0,0607 | 11,24 |
| 2 | MA1 | 0,335 | 7,50 | 5,20 | 5,78 | 2,13 | 0,0530 | 15,82 |
| 2 | MA1 | 0,423 | 8,36 | 6,62 | 4,97 | 2,57 | 0,0580 | 13,71 |
| 2 | MA1 | 0,514 | 11,74 | 7,50 | 6,56 | 2,24 | 0,0420 | 8,17 |
| 2 | MA1 | 0,445 | 11,20 | 6,07 | 6,21 | 3,18 | 0,0380 | 8,54 |
| 2 | MA1 | 0,464 | 9,41 | 7,80 | 5,03 | 2,65 | 0,0328 | 7,07 |
| 2 | MA1 | 0,714 | 6,49 | 5,88 | 6,97 | 1,85 | 0,0530 | 7,42 |
| 2 | MA1 | 0,608 | 10,56 | 4,09 | 6,31 | 1,94 | 0,0490 | 8,06 |
| 2 | MA1 | 0,534 | 11,43 | 3,92 | 5,38 | 2,04 | 0,0520 | 9,74 |
| 2 | MA2 | 0,341 | 9,99 | 7,19 | 6,02 | 2,70 | 0,0494 | 14,49 |
| 2 | MA2 | 0,390 | 11,21 | 6,48 | 6,43 | 3,19 | 0,0652 | 16,72 |
| 2 | MA2 | 0,439 | 10,39 | 7,69 | 7,29 | 2,96 | 0,0543 | 12,37 |
| 2 | MA2 | 0,413 | 9,64 | 6,69 | 6,36 | 3,32 | 0,0605 | 14,65 |
| 2 | MA2 | 0,469 | 11,35 | 8,25 | 6,96 | 2,72 | 0,0626 | 13,35 |
| 2 | MA2 | 0,314 | 11,37 | 7,51 | 7,03 | 4,17 | 0,0580 | 18,47 |
| 2 | MA2 | 0,388 | 10,18 | 6,60 | 7,78 | 4,37 | 0,0482 | 12,42 |
| 2 | MA2 | 0,353 | 9,86 | 6,95 | 5,70 | 3,90 | 0,0439 | 12,44 |
| 2 | MA2 | 0,394 | 11,42 | 7,94 | 5,84 | 2,69 | 0,0642 | 16,29 |
| 2 | MA2 | 0,397 | 10,94 | 7,76 | 6,16 | 3,60 | 0,0546 | 13,75 |
| 2 | MA2 | 0,478 | 11,36 | 8,23 | 5,18 | 2,99 | 0,0421 | 8,81 |
| 2 | MA2 | 0,384 | 10,44 | 7,75 | 8,52 | 2,28 | 0,0656 | 17,08 |
| 2 | MA2 | 0,298 | 11,00 | 8,85 | 6,11 | 4,30 | 0,0417 | 13,99 |
| 2 | MA2 | 0,464 | 10,60 | 8,59 | 8,05 | 2,43 | 0,0462 | 9,96 |
| 2 | MA2 | 0,430 | 11,99 | 6,64 | 8,30 | 3,75 | 0,0381 | 8,86 |
| 2 | MA2 | 0,479 | 9,93 | 7,53 | 7,81 | 3,15 | 0,0632 | 13,19 |
| 2 | MA2 | 0,432 | 8,77 | 6,32 | 5,92 | 2,23 | 0,0508 | 11,76 |
| 2 | MA2 | 0,336 | 10,58 | 7,60 | 7,67 | 2,64 | 0,0439 | 13,07 |
| 2 | MA2 | 0,276 | 11,12 | 9,08 | 6,56 | 2,15 | 0,0541 | 19,60 |
| 2 | MA2 | 0,314 | 11,31 | 7,81 | 6,00 | 1,82 | 0,0444 | 14,14 |
| 2 | MA2 | 0,346 | 11,21 | 6,13 | 5,56 | 2,62 | 0,0534 | 15,43 |
| 2 | MA2 | 0,429 | 9,32 | 6,62 | 6,51 | 2,46 | 0,0593 | 13,82 |
| 2 | MA2 | 0,512 | 10,38 | 6,60 | 5,78 | 2,71 | 0,0515 | 10,06 |
| 2 | MA2 | 0,363 | 10,26 | 7,36 | 8,04 | 2,54 | 0,0320 | 8,82 |
| 2 | MA2 | 0,435 | 9,56 | 6,50 | 6,56 | 2,38 | 0,0631 | 14,51 |
| 2 | MA3 | 0,390 | 9,59 | 7,25 | 6,42 | 2,47 | 0,0673 | 17,26 |
| 2 | MA3 | 0,335 | 7,63 | 4,69 | 6,37 | 2,49 | 0,0595 | 17,76 |
| 2 | MA3 | 0,464 | 11,43 | 6,67 | 5,18 | 1,95 | 0,0520 | 11,21 |
| 2 | MA3 | 0,287 | 10,13 | 6,62 | 5,87 | 2,34 | 0,0560 | 19,51 |
| 2 | MA3 | 0,393 | 11,98 | 6,95 | 7,72 | 1,85 | 0,0665 | 16,92 |
| 2 | MA3 | 0,251 | 7,80 | 6,60 | 6,03 | 0,34 | 0,0270 | 10,76 |
| 2 | MA3 | 0,218 | 6,68 | 6,06 | 6,53 | 1,93 | 0,0455 | 20,87 |
| 2 | MA3 | 0,330 | 10,30 | 6,43 | 6,64 | 1,23 | 0,0590 | 17,88 |
| 2 | MA3 | 0,207 | 8,60 | 5,39 | 5,32 | 1,96 | 0,0435 | 21,01 |
| 2 | MA3 | 0,383 | 9,45 | 7,63 | 6,88 | 1,96 | 0,0636 | 16,61 |
| 2 | MA3 | 0,480 | 11,75 | 6,62 | 6,82 | 1,26 | 0,0445 | 9,27 |
| 2 | MA3 | 0,420 | 9,12 | 6,84 | 5,33 | 3,13 | 0,0632 | 15,05 |
| 2 | MA3 | 0,339 | 8,96 | 4,43 | 6,90 | 2,18 | 0,0569 | 16,78 |
| 2 | MA3 | 0,210 | 7,61 | 5,24 | 6,00 | 2,26 | 0,0333 | 15,86 |
| 2 | MA3 | 0,307 | 9,54 | 4,27 | 5,05 | 1,40 | 0,0433 | 14,10 |
| 2 | MA3 | 0,360 | 9,41 | 8,81 | 5,49 | 2,79 | 0,0352 | 9,78 |
| 2 | MA3 | 0,368 | 11,68 | 6,21 | 6,74 | 1,93 | 0,0625 | 16,98 |
| 2 | MA3 | 0,455 | 8,05 | 6,38 | 7,03 | 2,47 | 0,0508 | 11,16 |
| 2 | MA3 | 0,315 | 11,56 | 7,18 | 4,30 | 1,92 | 0,0523 | 16,60 |
| 2 | MA3 | 0,415 | 10,84 | 5,81 | 5,52 | 1,73 | 0,0597 | 14,39 |
| 2 | MA3 | 0,423 | 7,63 | 6,47 | 6,04 | 2,28 | 0,0531 | 12,55 |
| 2 | MA3 | 0,224 | 8,28 | 3,95 | 6,32 | 3,12 | 0,0421 | 18,79 |
| 2 | MA3 | 0,204 | 10,00 | 6,72 | 6,14 | 2,10 | 0,0427 | 20,93 |
| 2 | MA3 | 0,407 | 8,76 | 5,26 | 5,15 | 3,18 | 0,0548 | 13,46 |
| 2 | MA3 | 0,358 | 10,79 | 4,84 | 5,56 | 1,28 | 0,0440 | 12,29 |
| 2 | MA4 | 0,415 | 12,46 | 7,95 | 7,47 | 2,61 | 0,0343 | 8,26 |
| 2 | MA4 | 0,457 | 11,84 | 8,20 | 6,66 | 2,93 | 0,0476 | 10,43 |
| 2 | MA4 | 0,451 | 12,20 | 9,78 | 7,82 | 2,81 | 0,0470 | 10,43 |
| 2 | MA4 | 0,450 | 12,22 | 8,12 | 6,74 | 2,17 | 0,0386 | 8,58 |
| 2 | MA4 | 0,424 | 12,60 | 7,65 | 6,77 | 2,52 | 0,0260 | 6,14 |
| 2 | MA4 | 0,423 | 10,45 | 8,24 | 6,60 | 2,30 | 0,0269 | 6,36 |
| 2 | MA4 | 0,243 | 10,20 | 9,72 | 6,10 | 2,61 | 0,0364 | 14,96 |
| 2 | MA4 | 0,249 | 9,66 | 6,27 | 6,42 | 1,95 | 0,0269 | 10,79 |
| 2 | MA4 | 0,394 | 11,27 | 7,30 | 6,87 | 2,91 | 0,0373 | 9,47 |
| 2 | MA4 | 0,346 | 10,73 | 5,93 | 6,28 | 2,00 | 0,0297 | 8,58 |
| 2 | MA4 | 0,387 | 10,14 | 7,72 | 6,08 | 2,63 | 0,0311 | 8,04 |
| 2 | MA4 | 0,404 | 11,10 | 9,81 | 6,73 | 2,81 | 0,0325 | 8,05 |
| 2 | MA4 | 0,376 | 10,96 | 5,24 | 6,55 | 2,11 | 0,0287 | 7,63 |
| 2 | MA4 | 0,424 | 10,62 | 8,33 | 6,28 | 3,07 | 0,0284 | 6,70 |
| 2 | MA4 | 0,366 | 9,38 | 7,18 | 4,95 | 3,09 | 0,0358 | 9,79 |
| 2 | MA4 | 0,397 | 10,85 | 7,41 | 6,29 | 2,25 | 0,0292 | 7,35 |
| 2 | MA4 | 0,376 | 11,16 | 7,42 | 7,14 | 2,91 | 0,0369 | 9,81 |
| 2 | MA4 | 0,377 | 9,28 | 7,24 | 5,93 | 2,49 | 0,0240 | 6,37 |
| 2 | MA4 | 0,347 | 9,55 | 6,18 | 6,14 | 2,51 | 0,0315 | 9,08 |
| 2 | MA4 | 0,293 | 9,66 | 5,72 | 6,83 | 1,98 | 0,0260 | 8,88 |
| 2 | MA4 | 0,330 | 10,33 | 7,86 | 6,31 | 2,60 | 0,0266 | 8,06 |
| 2 | MA4 | 0,425 | 9,87 | 7,14 | 6,13 | 2,18 | 0,0283 | 6,66 |
| 2 | MA4 | 0,429 | 9,76 | 7,41 | 6,44 | 2,56 | 0,0269 | 6,28 |
| 2 | MA4 | 0,370 | 10,82 | 7,76 | 5,64 | 2,54 | 0,0231 | 6,25 |
| 2 | MA4 | 0,422 | 11,97 | 7,07 | 6,73 | 1,78 | 0,0241 | 5,72 |
| 2 | MA5 | 0,372 | 11,04 | 6,30 | 4,64 | 1,30 | 0,0519 | 13,95 |
| 2 | MA5 | 0,394 | 10,60 | 5,02 | 6,30 | 1,80 | 0,0510 | 12,94 |
| 2 | MA5 | 0,332 | 11,40 | 6,15 | 6,23 | 2,25 | 0,0480 | 14,45 |
| 2 | MA5 | 0,229 | 7,43 | 3,40 | 5,14 | 2,07 | 0,0438 | 19,13 |
| 2 | MA5 | 0,424 | 11,30 | 6,06 | 7,07 | 2,12 | 0,0526 | 12,41 |
| 2 | MA5 | 0,342 | 11,56 | 6,19 | 8,40 | 2,60 | 0,0640 | 18,71 |
| 2 | MA5 | 0,381 | 10,20 | 7,52 | 8,20 | 3,92 | 0,0591 | 15,51 |
| 2 | MA5 | 0,230 | 11,51 | 4,80 | 8,42 | 2,60 | 0,0340 | 14,78 |
| 2 | MA5 | 0,520 | 9,58 | 4,50 | 7,07 | 2,36 | 0,0660 | 12,69 |
| 2 | MA5 | 0,280 | 10,30 | 5,50 | 10,23 | 2,40 | 0,0340 | 12,14 |
| 2 | MA5 | 0,540 | 9,60 | 7,30 | 8,01 | 2,54 | 0,0636 | 11,78 |
| 2 | MA5 | 0,440 | 12,38 | 7,52 | 7,51 | 2,35 | 0,0539 | 12,25 |
| 2 | MA5 | 0,560 | 10,44 | 5,44 | 10,12 | 2,12 | 0,0593 | 10,59 |
| 2 | MA5 | 0,342 | 9,80 | 6,06 | 6,98 | 2,23 | 0,0679 | 19,85 |
| 2 | MA5 | 0,468 | 9,08 | 5,40 | 7,05 | 2,50 | 0,0385 | 8,23 |
| 2 | MA5 | 0,211 | 8,90 | 8,05 | 8,82 | 3,17 | 0,0412 | 19,53 |
| 2 | MA5 | 0,350 | 9,80 | 5,43 | 9,04 | 2,40 | 0,0518 | 14,80 |
| 2 | MA5 | 0,408 | 12,45 | 6,05 | 7,66 | 3,30 | 0,0515 | 12,62 |
| 2 | MA5 | 0,224 | 11,90 | 4,40 | 9,28 | 2,65 | 0,0340 | 15,18 |
| 2 | MA5 | 0,360 | 6,60 | 4,07 | 6,95 | 2,40 | 0,0364 | 10,11 |
| 2 | MA5 | 0,420 | 11,02 | 6,46 | 7,87 | 2,74 | 0,0240 | 5,71 |
| 2 | MA5 | 0,325 | 10,54 | 6,12 | 8,07 | 3,40 | 0,0261 | 8,03 |
| 2 | MA5 | 0,514 | 11,40 | 9,26 | 8,57 | 2,14 | 0,0323 | 6,28 |
| 2 | MA5 | 0,520 | 12,31 | 7,16 | 8,32 | 2,18 | 0,0234 | 4,50 |
| 2 | MA5 | 0,557 | 11,60 | 7,30 | 8,30 | 2,30 | 0,0340 | 6,10 |
| 2 | BA1 | 0,663 | 11,46 | 4,69 | 4,61 | 1,85 | 0,0650 | 9,80 |
| 2 | BA1 | 0,549 | 12,66 | 5,95 | 8,40 | 3,44 | 0,0830 | 15,12 |
| 2 | BA1 | 0,563 | 12,97 | 6,87 | 10,20 | 4,04 | 0,0660 | 11,72 |
| 2 | BA1 | 0,372 | 11,16 | 5,62 | 9,40 | 3,52 | 0,0550 | 14,78 |
| 2 | BA1 | 0,432 | 12,47 | 7,16 | 3,00 | 4,68 | 0,0460 | 10,65 |
| 2 | BA1 | 0,468 | 10,56 | 7,27 | 6,10 | 3,03 | 0,0255 | 5,45 |
| 2 | BA1 | 0,486 | 9,59 | 5,24 | 6,40 | 1,96 | 0,0434 | 8,92 |
| 2 | BA1 | 0,349 | 11,11 | 8,22 | 9,34 | 3,17 | 0,0540 | 15,46 |
| 2 | BA1 | 0,488 | 7,54 | 5,89 | 7,40 | 4,05 | 0,0830 | 17,01 |
| 2 | BA1 | 0,640 | 11,02 | 6,34 | 6,61 | 2,47 | 0,0865 | 13,52 |
| 2 | BA1 | 0,445 | 11,48 | 7,01 | 6,32 | 2,31 | 0,0640 | 14,38 |
| 2 | BA1 | 0,474 | 12,54 | 8,19 | 8,60 | 2,74 | 0,0510 | 10,76 |
| 2 | BA1 | 0,456 | 11,65 | 8,47 | 7,90 | 2,69 | 0,0340 | 7,45 |
| 2 | BA1 | 0,426 | 11,40 | 6,30 | 7,50 | 2,37 | 0,0327 | 7,68 |
| 2 | BA1 | 0,390 | 8,71 | 7,41 | 8,21 | 2,16 | 0,0474 | 12,17 |
| 2 | BA1 | 0,342 | 11,07 | 8,06 | 7,15 | 1,24 | 0,0681 | 19,94 |
| 2 | BA1 | 0,464 | 11,31 | 2,94 | 7,34 | 2,40 | 0,0590 | 12,70 |
| 2 | BA1 | 0,485 | 11,11 | 4,54 | 7,22 | 2,30 | 0,0988 | 20,37 |
| 2 | BA1 | 0,375 | 11,20 | 4,44 | 7,14 | 2,76 | 0,0601 | 16,03 |
| 2 | BA1 | 0,298 | 11,52 | 6,16 | 7,51 | 2,70 | 0,0598 | 20,07 |
| 2 | BA1 | 0,371 | 10,44 | 4,50 | 8,27 | 2,80 | 0,0665 | 17,92 |
| 2 | BA1 | 0,284 | 11,20 | 5,90 | 8,70 | 2,10 | 0,0447 | 15,74 |
| 2 | BA1 | 0,612 | 11,10 | 5,08 | 7,10 | 1,16 | 0,0668 | 10,92 |
| 2 | BA1 | 0,541 | 7,14 | 6,76 | 7,84 | 2,94 | 0,0664 | 12,27 |
| 2 | BA1 | 0,524 | 7,22 | 6,29 | 7,36 | 2,60 | 0,0718 | 13,70 |
| 2 | VA1 | 0,367 | 10,83 | 8,75 | 5,64 | 2,99 | 0,0305 | 8,32 |
| 2 | VA1 | 0,505 | 12,97 | 7,52 | 7,38 | 2,40 | 0,0339 | 6,71 |
| 2 | VA1 | 0,348 | 10,71 | 5,78 | 6,39 | 2,29 | 0,0360 | 10,36 |
| 2 | VA1 | 0,372 | 10,26 | 7,36 | 6,33 | 2,53 | 0,0356 | 9,58 |
| 2 | VA1 | 0,275 | 8,42 | 5,87 | 5,91 | 2,79 | 0,0273 | 9,92 |
| 2 | VA1 | 0,350 | 9,14 | 7,04 | 6,46 | 2,30 | 0,0427 | 12,21 |
| 2 | VA1 | 0,387 | 11,07 | 8,24 | 5,53 | 2,17 | 0,0305 | 7,88 |
| 2 | VA1 | 0,443 | 10,72 | 7,85 | 6,06 | 2,16 | 0,0307 | 6,93 |
| 2 | VA1 | 0,447 | 11,26 | 7,88 | 6,19 | 2,25 | 0,0376 | 8,42 |
| 2 | VA1 | 0,435 | 10,22 | 7,90 | 5,72 | 2,25 | 0,0277 | 6,36 |
| 2 | VA1 | 0,357 | 9,64 | 7,93 | 5,56 | 2,01 | 0,0281 | 7,86 |
| 2 | VA1 | 0,436 | 10,41 | 9,08 | 6,73 | 2,40 | 0,0323 | 7,40 |
| 2 | VA1 | 0,409 | 10,39 | 8,23 | 6,09 | 3,05 | 0,0270 | 6,60 |
| 2 | VA1 | 0,421 | 11,03 | 8,03 | 7,00 | 1,96 | 0,0365 | 8,68 |
| 2 | VA1 | 0,343 | 10,64 | 5,30 | 7,09 | 2,02 | 0,0265 | 7,72 |
| 2 | VA1 | 0,345 | 10,75 | 6,78 | 6,83 | 2,57 | 0,0312 | 9,06 |
| 2 | VA1 | 0,295 | 10,71 | 5,53 | 6,77 | 2,52 | 0,0300 | 10,17 |
| 2 | VA1 | 0,384 | 10,21 | 6,38 | 6,44 | 2,22 | 0,0344 | 8,96 |
| 2 | VA1 | 0,294 | 11,51 | 5,93 | 6,61 | 1,19 | 0,0251 | 8,53 |
| 2 | VA1 | 0,238 | 7,65 | 6,90 | 4,89 | 2,51 | 0,0403 | 16,93 |
| 2 | VA1 | 0,440 | 10,00 | 7,25 | 5,90 | 2,51 | 0,0483 | 10,98 |
| 2 | VA1 | 0,309 | 9,63 | 7,34 | 5,77 | 1,72 | 0,0337 | 10,89 |
| 2 | VA1 | 0,433 | 11,46 | 7,01 | 7,67 | 1,56 | 0,0217 | 5,02 |
| 2 | VA1 | 0,339 | 10,39 | 6,54 | 6,34 | 1,73 | 0,0369 | 10,89 |
| 2 | VA1 | 0,312 | 9,90 | 6,60 | 6,29 | 2,11 | 0,0288 | 9,22 |
| 2 | MC1 | 0,343 | 10,96 | 6,39 | 8,93 | 3,67 | 0,0712 | 20,78 |
| 2 | MC1 | 0,325 | 10,03 | 6,36 | 6,34 | 1,17 | 0,0355 | 10,93 |
| 2 | MC1 | 0,329 | 9,50 | 5,58 | 6,83 | 1,21 | 0,0650 | 19,76 |
| 2 | MC1 | 0,256 | 9,26 | 6,88 | 6,37 | 1,27 | 0,0380 | 14,84 |
| 2 | MC1 | 0,367 | 9,16 | 5,97 | 5,94 | 1,33 | 0,0323 | 8,80 |
| 2 | MC1 | 0,336 | 10,31 | 6,85 | 6,38 | 1,47 | 0,0605 | 18,01 |
| 2 | MC1 | 0,365 | 9,34 | 5,80 | 8,18 | 1,56 | 0,0780 | 21,37 |
| 2 | MC1 | 0,363 | 9,56 | 6,70 | 6,23 | 1,68 | 0,0660 | 18,18 |
| 2 | MC1 | 0,395 | 10,09 | 6,69 | 5,57 | 1,75 | 0,0501 | 12,68 |
| 2 | MC1 | 0,485 | 10,58 | 6,78 | 3,82 | 0,66 | 0,0290 | 5,98 |
| 2 | MC1 | 0,485 | 10,63 | 6,67 | 5,88 | 1,83 | 0,0333 | 6,87 |
| 2 | MC1 | 0,295 | 9,03 | 5,14 | 5,85 | 1,23 | 0,0430 | 14,58 |
| 2 | MC1 | 0,384 | 9,33 | 5,45 | 6,40 | 1,30 | 0,0446 | 11,61 |
| 2 | MC1 | 0,329 | 8,62 | 6,80 | 5,42 | 1,14 | 0,0420 | 12,77 |
| 2 | MC1 | 0,397 | 8,30 | 5,29 | 6,90 | 1,76 | 0,0767 | 19,32 |
| 2 | MC1 | 0,436 | 11,36 | 6,77 | 6,91 | 1,68 | 0,0628 | 14,40 |
| 2 | MC1 | 0,378 | 9,13 | 5,69 | 6,70 | 1,65 | 0,0245 | 6,48 |
| 2 | MC1 | 0,401 | 8,66 | 5,46 | 5,75 | 1,73 | 0,0558 | 13,92 |
| 2 | MC1 | 0,338 | 9,93 | 5,12 | 5,40 | 1,47 | 0,0415 | 12,28 |
| 2 | MC1 | 0,336 | 10,02 | 5,61 | 6,17 | 1,83 | 0,0590 | 17,56 |
| 2 | MC1 | 0,382 | 10,02 | 6,15 | 5,66 | 1,39 | 0,0367 | 9,61 |
| 2 | MC1 | 0,326 | 9,77 | 5,90 | 6,24 | 0,65 | 0,0490 | 15,03 |
| 2 | MC1 | 0,357 | 9,43 | 5,60 | 5,70 | 1,33 | 0,0480 | 13,45 |
| 2 | MC1 | 0,377 | 10,24 | 6,18 | 8,12 | 1,65 | 0,0450 | 11,94 |
| 2 | MC1 | 0,339 | 9,69 | 5,46 | 5,82 | 0,88 | 0,0585 | 17,26 |
| 2 | MO2 | 0,382 | 9,11 | 5,40 | 5,21 | 0,95 | 0,0305 | 7,98 |
| 2 | MO2 | 0,355 | 8,89 | 4,35 | 5,76 | 0,88 | 0,0391 | 11,02 |
| 2 | MO2 | 0,427 | 10,75 | 3,98 | 5,75 | 0,87 | 0,0356 | 8,35 |
| 2 | MO2 | 0,419 | 9,45 | 4,32 | 5,67 | 1,29 | 0,0397 | 9,47 |
| 2 | MO2 | 0,500 | 11,28 | 6,24 | 5,46 | 1,48 | 0,0344 | 6,89 |
| 2 | MO2 | 0,478 | 11,54 | 5,75 | 6,05 | 1,23 | 0,0464 | 9,71 |
| 2 | MO2 | 0,559 | 10,78 | 6,04 | 5,14 | 1,66 | 0,0334 | 5,97 |
| 2 | MO2 | 0,529 | 11,19 | 5,47 | 4,23 | 1,52 | 0,0298 | 5,63 |
| 2 | MO2 | 0,442 | 10,07 | 6,08 | 5,14 | 1,66 | 0,0346 | 7,83 |
| 2 | MO2 | 0,364 | 10,22 | 4,68 | 5,01 | 1,15 | 0,0316 | 8,67 |
| 2 | MO2 | 0,416 | 9,25 | 5,33 | 4,26 | 0,90 | 0,0364 | 8,76 |
| 2 | MO2 | 0,480 | 10,86 | 4,96 | 6,31 | 1,43 | 0,0589 | 12,28 |
| 2 | MO2 | 0,358 | 8,29 | 4,34 | 5,11 | 0,87 | 0,0445 | 12,43 |
| 2 | MO2 | 0,479 | 11,48 | 4,78 | 5,13 | 0,93 | 0,0349 | 7,29 |
| 2 | MO2 | 0,424 | 8,72 | 6,60 | 4,10 | 1,44 | 0,0290 | 6,83 |
| 2 | MO2 | 0,412 | 10,74 | 5,31 | 6,08 | 1,74 | 0,0452 | 10,97 |
| 2 | MO2 | 0,387 | 9,55 | 4,78 | 4,64 | 0,81 | 0,0285 | 7,37 |
| 2 | MO2 | 0,346 | 8,52 | 4,65 | 6,85 | 2,38 | 0,0363 | 10,48 |
| 2 | MO2 | 0,493 | 10,35 | 6,78 | 4,18 | 2,53 | 0,0455 | 9,23 |
| 2 | MO2 | 0,468 | 10,63 | 5,58 | 4,30 | 1,42 | 0,0354 | 7,57 |
| 2 | MO2 | 0,355 | 8,72 | 4,20 | 6,06 | 1,99 | 0,0440 | 12,40 |
| 2 | MO2 | 0,461 | 10,42 | 4,26 | 4,86 | 1,00 | 0,0371 | 8,05 |
| 2 | MO2 | 0,496 | 10,21 | 5,43 | 4,67 | 1,89 | 0,0307 | 6,19 |
| 2 | MO2 | 0,387 | 7,85 | 5,13 | 4,23 | 1,48 | 0,0345 | 8,91 |
| 2 | MO2 | 0,430 | 9,09 | 5,42 | 5,46 | 1,12 | 0,0293 | 6,81 |
| 2 | MO3 | 0,324 | 6,46 | 6,40 | 5,01 | 1,80 | 0,0452 | 13,95 |
| 2 | MO3 | 0,444 | 8,49 | 6,06 | 3,89 | 0,86 | 0,0464 | 10,45 |
| 2 | MO3 | 0,431 | 9,65 | 7,14 | 4,95 | 1,66 | 0,0532 | 12,34 |
| 2 | MO3 | 0,358 | 10,17 | 5,03 | 6,93 | 1,03 | 0,0614 | 17,15 |
| 2 | MO3 | 0,344 | 11,66 | 6,58 | 5,49 | 0,98 | 0,0345 | 10,03 |
| 2 | MO3 | 0,297 | 8,68 | 4,69 | 5,43 | 1,47 | 0,0356 | 11,99 |
| 2 | MO3 | 0,370 | 7,71 | 5,78 | 4,54 | 1,68 | 0,0479 | 12,95 |
| 2 | MO3 | 0,347 | 6,85 | 5,02 | 4,53 | 0,94 | 0,0478 | 13,78 |
| 2 | MO3 | 0,505 | 12,30 | 6,68 | 6,75 | 1,39 | 0,0588 | 11,64 |
| 2 | MO3 | 0,379 | 8,94 | 4,92 | 5,16 | 1,13 | 0,0489 | 12,90 |
| 2 | MO3 | 0,468 | 10,42 | 5,38 | 6,50 | 0,99 | 0,0600 | 12,82 |
| 2 | MO3 | 0,419 | 10,72 | 5,26 | 6,09 | 0,91 | 0,0353 | 8,42 |
| 2 | MO3 | 0,400 | 8,86 | 6,69 | 5,51 | 1,56 | 0,0560 | 14,00 |
| 2 | MO3 | 0,497 | 10,51 | 5,94 | 4,64 | 2,16 | 0,0640 | 12,88 |
| 2 | MO3 | 0,225 | 7,96 | 4,00 | 4,71 | 1,01 | 0,0387 | 17,20 |
| 2 | MO3 | 0,393 | 10,11 | 4,24 | 5,89 | 0,78 | 0,0520 | 13,23 |
| 2 | MO3 | 0,484 | 9,94 | 6,69 | 4,41 | 1,34 | 0,0571 | 11,80 |
| 2 | MO3 | 0,337 | 8,38 | 5,89 | 4,90 | 0,97 | 0,0297 | 8,81 |
| 2 | MO3 | 0,431 | 8,82 | 5,05 | 5,79 | 0,73 | 0,0362 | 8,40 |
| 2 | MO3 | 0,402 | 9,82 | 5,50 | 5,63 | 1,12 | 0,0344 | 8,56 |
| 2 | MO3 | 0,465 | 8,46 | 6,94 | 4,81 | 1,16 | 0,0245 | 5,27 |
| 2 | MO3 | 0,481 | 10,05 | 5,56 | 5,02 | 0,67 | 0,0396 | 8,23 |
| 2 | MO3 | 0,451 | 10,13 | 4,62 | 5,12 | 1,43 | 0,0316 | 7,01 |
| 2 | MO3 | 0,480 | 9,47 | 5,13 | 5,31 | 1,20 | 0,0329 | 6,85 |
| 2 | MO3 | 0,571 | 9,82 | 7,34 | 4,83 | 1,22 | 0,0562 | 9,84 |
| 2 | MO4 | 0,466 | 8,92 | 5,13 | 3,86 | 0,70 | 0,0350 | 7,51 |
| 2 | MO4 | 0,487 | 7,99 | 6,56 | 4,78 | 1,34 | 0,0515 | 10,57 |
| 2 | MO4 | 0,589 | 9,68 | 7,12 | 5,28 | 1,22 | 0,0571 | 9,69 |
| 2 | MO4 | 0,446 | 7,28 | 5,29 | 6,32 | 1,08 | 0,0461 | 10,34 |
| 2 | MO4 | 0,458 | 10,02 | 6,46 | 4,46 | 1,36 | 0,0552 | 12,05 |
| 2 | MO4 | 0,573 | 11,49 | 8,08 | 5,32 | 1,92 | 0,0688 | 12,01 |
| 2 | MO4 | 0,477 | 9,94 | 6,79 | 5,09 | 0,94 | 0,0692 | 14,51 |
| 2 | MO4 | 0,486 | 11,12 | 7,33 | 5,05 | 1,07 | 0,0589 | 12,12 |
| 2 | MO4 | 0,485 | 11,57 | 6,64 | 6,81 | 1,53 | 0,0726 | 14,97 |
| 2 | MO4 | 0,490 | 8,47 | 7,16 | 5,02 | 1,30 | 0,0581 | 11,86 |
| 2 | MO4 | 0,410 | 9,68 | 7,36 | 6,32 | 1,57 | 0,0675 | 16,46 |
| 2 | MO4 | 0,423 | 9,66 | 4,13 | 5,39 | 1,75 | 0,0527 | 12,46 |
| 2 | MO4 | 0,453 | 7,91 | 6,48 | 5,68 | 2,63 | 0,0596 | 13,16 |
| 2 | MO4 | 0,393 | 8,92 | 6,64 | 4,62 | 1,45 | 0,0361 | 9,19 |
| 2 | MO4 | 0,452 | 8,45 | 5,83 | 5,20 | 0,76 | 0,0708 | 15,66 |
| 2 | MO4 | 0,455 | 8,05 | 6,06 | 4,92 | 0,91 | 0,0502 | 11,03 |
| 2 | MO4 | 0,383 | 8,37 | 5,74 | 6,99 | 1,06 | 0,0566 | 14,78 |
| 2 | MO4 | 0,414 | 9,79 | 6,72 | 4,89 | 0,77 | 0,0474 | 11,45 |
| 2 | MO4 | 0,485 | 9,26 | 5,28 | 5,06 | 1,13 | 0,0719 | 14,82 |
| 2 | MO4 | 0,535 | 8,72 | 6,10 | 5,18 | 1,57 | 0,0509 | 9,51 |
| 2 | MO4 | 0,456 | 8,98 | 6,10 | 4,35 | 1,28 | 0,0560 | 12,28 |
| 2 | MO4 | 0,448 | 5,24 | 6,37 | 4,68 | 0,61 | 0,0437 | 9,75 |
| 2 | MO4 | 0,428 | 10,62 | 4,80 | 6,67 | 0,78 | 0,0400 | 9,35 |
| 2 | MO4 | 0,387 | 9,68 | 6,67 | 5,09 | 0,48 | 0,0395 | 10,21 |
| 2 | MO4 | 0,389 | 8,89 | 5,46 | 4,39 | 0,63 | 0,0416 | 10,69 |
| 2 | MO5 | 0,435 | 9,25 | 3,30 | 4,68 | 0,86 | 0,0236 | 5,43 |
| 2 | MO5 | 0,376 | 8,99 | 3,52 | 5,22 | 2,25 | 0,0321 | 8,54 |
| 2 | MO5 | 0,538 | 9,92 | 4,72 | 4,63 | 0,84 | 0,0425 | 7,90 |
| 2 | MO5 | 0,516 | 8,54 | 4,17 | 5,18 | 0,94 | 0,0276 | 5,35 |
| 2 | MO5 | 0,472 | 11,21 | 6,22 | 5,58 | 1,06 | 0,0331 | 7,01 |
| 2 | MO5 | 0,452 | 8,92 | 5,40 | 6,58 | 1,54 | 0,0326 | 7,21 |
| 2 | MO5 | 0,501 | 9,12 | 5,17 | 5,46 | 0,60 | 0,0417 | 8,32 |
| 2 | MO5 | 0,368 | 8,97 | 4,88 | 3,14 | 0,89 | 0,0378 | 10,27 |
| 2 | MO5 | 0,426 | 11,26 | 5,17 | 5,61 | 1,40 | 0,0388 | 9,11 |
| 2 | MO5 | 0,675 | 8,83 | 3,36 | 5,96 | 0,95 | 0,0291 | 4,31 |
| 2 | MO5 | 0,423 | 10,08 | 4,83 | 3,89 | 0,89 | 0,0315 | 7,45 |
| 2 | MO5 | 0,287 | 9,09 | 3,60 | 4,16 | 1,54 | 0,0526 | 18,33 |
| 2 | MO5 | 0,552 | 9,01 | 4,77 | 5,20 | 1,66 | 0,0316 | 5,72 |
| 2 | MO5 | 0,437 | 10,30 | 6,29 | 6,56 | 0,65 | 0,0345 | 7,89 |
| 2 | MO5 | 0,292 | 9,66 | 5,88 | 4,16 | 1,00 | 0,0367 | 12,57 |
| 2 | MO5 | 0,316 | 11,97 | 5,01 | 4,41 | 1,31 | 0,0415 | 13,13 |
| 2 | MO5 | 0,348 | 7,43 | 3,76 | 3,77 | 1,25 | 0,0288 | 8,28 |
| 2 | MO5 | 0,426 | 7,48 | 5,05 | 6,22 | 1,18 | 0,0313 | 7,35 |
| 2 | MO5 | 0,401 | 8,02 | 6,36 | 4,07 | 1,33 | 0,0205 | 5,11 |
| 2 | MO5 | 0,299 | 10,65 | 4,85 | 5,68 | 0,91 | 0,0370 | 12,37 |
| 2 | MO5 | 0,315 | 8,94 | 4,27 | 5,36 | 0,61 | 0,0496 | 15,75 |
| 2 | MO5 | 0,308 | 9,37 | 5,66 | 5,46 | 0,89 | 0,0521 | 16,92 |
| 2 | MO5 | 0,426 | 8,78 | 5,80 | 4,31 | 0,56 | 0,0486 | 11,41 |
| 2 | MO5 | 0,368 | 9,60 | 5,50 | 3,57 | 0,90 | 0,0311 | 8,45 |
| 2 | MO5 | 0,355 | 10,84 | 5,60 | 5,13 | 1,51 | 0,0563 | 15,86 |
| 2 | MO6 | 0,476 | 11,22 | 8,67 | 7,11 | 3,15 | 0,0443 | 9,31 |
| 2 | MO6 | 0,442 | 12,19 | 8,63 | 6,97 | 3,30 | 0,0427 | 9,67 |
| 2 | MO6 | 0,464 | 12,71 | 8,81 | 7,28 | 2,02 | 0,0379 | 8,16 |
| 2 | MO6 | 0,409 | 10,18 | 8,54 | 6,33 | 4,17 | 0,0423 | 10,34 |
| 2 | MO6 | 0,507 | 12,69 | 8,31 | 7,64 | 2,44 | 0,0553 | 10,91 |
| 2 | MO6 | 0,458 | 10,92 | 9,08 | 6,12 | 1,70 | 0,0383 | 8,37 |
| 2 | MO6 | 0,338 | 10,43 | 7,52 | 6,50 | 2,33 | 0,0395 | 11,67 |
| 2 | MO6 | 0,383 | 12,00 | 6,97 | 7,34 | 1,97 | 0,0480 | 12,55 |
| 2 | MO6 | 0,495 | 12,15 | 9,06 | 6,27 | 2,41 | 0,0388 | 7,84 |
| 2 | MO6 | 0,459 | 10,67 | 7,74 | 7,19 | 2,69 | 0,0671 | 14,62 |
| 2 | MO6 | 0,375 | 11,14 | 8,26 | 5,42 | 2,01 | 0,0406 | 10,82 |
| 2 | MO6 | 0,490 | 13,49 | 7,54 | 9,03 | 2,36 | 0,0525 | 10,72 |
| 2 | MO6 | 0,469 | 11,99 | 5,73 | 7,07 | 2,69 | 0,0499 | 10,64 |
| 2 | MO6 | 0,467 | 11,99 | 7,17 | 6,89 | 2,39 | 0,0497 | 10,63 |
| 2 | MO6 | 0,291 | 11,69 | 6,63 | 6,66 | 1,51 | 0,0317 | 10,90 |
| 2 | MO6 | 0,312 | 10,42 | 6,89 | 7,63 | 2,06 | 0,0438 | 14,04 |
| 2 | MO6 | 0,307 | 9,39 | 5,42 | 5,74 | 2,44 | 0,0526 | 17,11 |
| 2 | MO6 | 0,270 | 8,93 | 5,92 | 6,52 | 1,94 | 0,0417 | 15,47 |
| 2 | MO6 | 0,318 | 11,21 | 7,29 | 7,12 | 1,92 | 0,0394 | 12,39 |
| 2 | MO6 | 0,393 | 13,13 | 7,01 | 7,29 | 1,99 | 0,0288 | 7,32 |
| 2 | MO6 | 0,360 | 8,47 | 8,37 | 6,00 | 2,15 | 0,0443 | 12,32 |
| 2 | MO6 | 0,457 | 10,78 | 8,88 | 5,73 | 2,37 | 0,0348 | 7,61 |
| 2 | MO6 | 0,378 | 11,78 | 6,93 | 6,87 | 1,62 | 0,0373 | 9,87 |
| 2 | MO6 | 0,410 | 11,40 | 8,21 | 5,50 | 1,25 | 0,0435 | 10,61 |
| 2 | MO6 | 0,208 | 10,12 | 5,60 | 6,48 | 1,75 | 0,0284 | 13,65 |
| 2 | AB1 | 0,375 | 7,75 | 5,42 | 4,42 | 1,63 | 0,0364 | 9,70 |
| 2 | AB1 | 0,346 | 10,72 | 5,28 | 7,41 | 1,19 | 0,0405 | 11,69 |
| 2 | AB1 | 0,443 | 10,45 | 6,23 | 6,64 | 2,06 | 0,0508 | 11,46 |
| 2 | AB1 | 0,506 | 10,50 | 7,04 | 6,38 | 1,94 | 0,0535 | 10,57 |
| 2 | AB1 | 0,377 | 9,40 | 6,65 | 6,64 | 1,73 | 0,0492 | 13,04 |
| 2 | AB1 | 0,466 | 9,84 | 7,96 | 6,59 | 1,14 | 0,0482 | 10,35 |
| 2 | AB1 | 0,463 | 9,58 | 5,80 | 6,00 | 1,60 | 0,0636 | 13,73 |
| 2 | AB1 | 0,464 | 10,37 | 6,24 | 3,87 | 1,98 | 0,0496 | 10,69 |
| 2 | AB1 | 0,509 | 9,60 | 8,01 | 5,99 | 2,06 | 0,0552 | 10,84 |
| 2 | AB1 | 0,554 | 10,89 | 6,91 | 6,20 | 1,81 | 0,0584 | 10,54 |
| 2 | AB1 | 0,342 | 8,82 | 5,10 | 5,39 | 1,60 | 0,0527 | 15,39 |
| 2 | AB1 | 0,329 | 9,23 | 5,56 | 6,62 | 2,08 | 0,0526 | 15,99 |
| 2 | AB1 | 0,322 | 8,40 | 6,02 | 5,42 | 3,07 | 0,0502 | 15,57 |
| 2 | AB1 | 0,304 | 9,10 | 5,24 | 6,52 | 1,26 | 0,0397 | 13,05 |
| 2 | AB1 | 0,428 | 10,75 | 7,13 | 6,02 | 1,57 | 0,0432 | 10,09 |
| 2 | AB1 | 0,365 | 10,11 | 6,64 | 7,43 | 3,50 | 0,0615 | 16,84 |
| 2 | AB1 | 0,465 | 9,61 | 6,50 | 6,26 | 2,17 | 0,0448 | 9,63 |
| 2 | AB1 | 0,344 | 8,49 | 7,25 | 5,02 | 2,10 | 0,0328 | 9,52 |
| 2 | AB1 | 0,320 | 8,62 | 6,63 | 5,04 | 1,37 | 0,0344 | 10,74 |
| 2 | AB1 | 0,176 | 8,01 | 3,32 | 5,38 | 1,18 | 0,0235 | 13,32 |
| 2 | AB1 | 0,481 | 10,43 | 6,01 | 6,79 | 1,48 | 0,0423 | 8,79 |
| 2 | AB1 | 0,248 | 6,82 | 4,02 | 4,98 | 1,07 | 0,0369 | 14,87 |
| 2 | AB1 | 0,388 | 8,80 | 5,51 | 5,18 | 1,13 | 0,0361 | 9,30 |
| 2 | AB1 | 0,428 | 8,80 | 4,45 | 5,37 | 1,13 | 0,0364 | 8,51 |
| 2 | AB1 | 0,493 | 10,50 | 6,81 | 6,50 | 1,37 | 0,0375 | 7,61 |
| 2 | PB1 | 0,415 | 10,48 | 4,46 | 6,37 | 0,82 | 0,0561 | 13,52 |
| 2 | PB1 | 0,370 | 10,56 | 3,26 | 6,78 | 1,22 | 0,0585 | 15,81 |
| 2 | PB1 | 0,288 | 7,27 | 3,76 | 5,67 | 1,34 | 0,0528 | 18,33 |
| 2 | PB1 | 0,382 | 8,47 | 3,82 | 6,70 | 1,38 | 0,0644 | 16,86 |
| 2 | PB1 | 0,430 | 10,33 | 4,65 | 5,74 | 1,17 | 0,0636 | 14,79 |
| 2 | PB1 | 0,325 | 7,87 | 3,69 | 6,25 | 1,12 | 0,0463 | 14,25 |
| 2 | PB1 | 0,306 | 7,47 | 4,99 | 5,75 | 1,56 | 0,0536 | 17,52 |
| 2 | PB1 | 0,429 | 10,10 | 5,81 | 5,75 | 0,96 | 0,0549 | 12,80 |
| 2 | PB1 | 0,332 | 9,47 | 3,47 | 6,21 | 1,88 | 0,0505 | 15,21 |
| 2 | PB1 | 0,444 | 9,51 | 5,22 | 6,82 | 0,93 | 0,0652 | 14,68 |
| 2 | PB1 | 0,238 | 7,98 | 5,15 | 6,12 | 1,31 | 0,0467 | 19,62 |
| 2 | PB1 | 0,325 | 8,60 | 3,68 | 6,67 | 1,30 | 0,0679 | 20,89 |
| 2 | PB1 | 0,447 | 9,36 | 5,53 | 5,14 | 1,06 | 0,0512 | 11,45 |
| 2 | PB1 | 0,452 | 9,41 | 4,78 | 6,01 | 1,02 | 0,0593 | 13,12 |
| 2 | PB1 | 0,318 | 10,08 | 2,71 | 6,18 | 0,89 | 0,0506 | 15,91 |
| 2 | PB1 | 0,390 | 9,50 | 4,72 | 5,66 | 1,05 | 0,0592 | 15,18 |
| 2 | PB1 | 0,296 | 9,68 | 2,58 | 6,60 | 0,88 | 0,0437 | 14,76 |
| 2 | PB1 | 0,447 | 9,72 | 5,14 | 5,94 | 0,94 | 0,0465 | 10,40 |
| 2 | PB1 | 0,370 | 9,87 | 3,52 | 6,33 | 1,02 | 0,0605 | 16,35 |
| 2 | PB1 | 0,430 | 9,57 | 4,40 | 5,63 | 0,94 | 0,0510 | 11,86 |
| 2 | PB1 | 0,322 | 8,51 | 4,09 | 5,81 | 0,88 | 0,0443 | 13,76 |
| 2 | PB1 | 0,407 | 10,92 | 4,82 | 5,17 | 0,85 | 0,0499 | 12,26 |
| 2 | PB1 | 0,447 | 11,42 | 4,01 | 6,85 | 1,31 | 0,0601 | 13,45 |
| 2 | PB1 | 0,503 | 11,84 | 5,33 | 6,60 | 1,13 | 0,0681 | 13,54 |
| 2 | PB1 | 0,241 | 8,39 | 4,08 | 5,85 | 1,80 | 0,0456 | 18,92 |
| 2 | PTB1 | 0,636 | 11,46 | 4,72 | 6,67 | 1,50 | 0,0515 | 8,10 |
| 2 | PTB1 | 0,334 | 9,39 | 3,64 | 6,97 | 1,27 | 0,0465 | 13,92 |
| 2 | PTB1 | 0,573 | 12,07 | 4,10 | 7,27 | 1,11 | 0,0439 | 7,66 |
| 2 | PTB1 | 0,531 | 12,36 | 5,80 | 4,62 | 1,92 | 0,0362 | 6,82 |
| 2 | PTB1 | 0,315 | 10,48 | 3,51 | 7,34 | 1,31 | 0,0497 | 15,78 |
| 2 | PTB1 | 0,541 | 10,47 | 6,84 | 5,24 | 1,15 | 0,0475 | 8,78 |
| 2 | PTB1 | 0,442 | 10,38 | 4,50 | 7,08 | 1,10 | 0,0510 | 11,54 |
| 2 | PTB1 | 0,540 | 10,21 | 4,66 | 6,38 | 1,57 | 0,0511 | 9,46 |
| 2 | PTB1 | 0,554 | 9,82 | 6,39 | 8,92 | 2,05 | 0,0418 | 7,55 |
| 2 | PTB1 | 0,462 | 9,93 | 5,92 | 6,30 | 1,62 | 0,0576 | 12,47 |
| 2 | PTB1 | 0,393 | 11,47 | 6,13 | 6,31 | 0,92 | 0,0590 | 15,01 |
| 2 | PTB1 | 0,497 | 9,40 | 4,85 | 5,71 | 0,87 | 0,0440 | 8,85 |
| 2 | PTB1 | 0,583 | 11,52 | 4,40 | 6,10 | 0,88 | 0,0360 | 6,17 |
| 2 | PTB1 | 0,538 | 11,35 | 4,36 | 7,24 | 1,08 | 0,0425 | 7,90 |
| 2 | PTB1 | 0,392 | 11,07 | 4,20 | 6,46 | 0,90 | 0,0471 | 12,02 |
| 2 | PTB1 | 0,459 | 11,30 | 4,51 | 6,97 | 1,08 | 0,0599 | 13,05 |
| 2 | PTB1 | 0,505 | 11,66 | 3,61 | 6,36 | 1,16 | 0,0592 | 11,72 |
| 2 | PTB1 | 0,644 | 11,90 | 4,97 | 6,48 | 0,74 | 0,0460 | 7,14 |
| 2 | PTB1 | 0,540 | 9,77 | 5,28 | 4,70 | 1,50 | 0,0535 | 9,91 |
| 2 | PTB1 | 0,387 | 11,14 | 3,56 | 7,60 | 0,85 | 0,0556 | 14,37 |
| 2 | PTB1 | 0,392 | 7,77 | 3,64 | 6,35 | 0,90 | 0,0522 | 13,32 |
| 2 | PTB1 | 0,510 | 10,58 | 5,84 | 4,86 | 0,83 | 0,0519 | 10,18 |
| 2 | PTB1 | 0,385 | 10,20 | 3,40 | 5,99 | 1,95 | 0,0491 | 12,75 |
| 2 | PTB1 | 0,675 | 11,15 | 6,14 | 6,87 | 1,45 | 0,0607 | 8,99 |
| 2 | PTB1 | 0,409 | 10,02 | 5,09 | 6,61 | 1,37 | 0,0512 | 12,52 |
| 2 | SFB1 | 0,624 | 11,95 | 9,63 | 5,58 | 2,24 | 0,0387 | 6,20 |
| 2 | SFB1 | 0,704 | 12,49 | 7,52 | 6,19 | 1,50 | 0,0465 | 6,60 |
| 2 | SFB1 | 0,767 | 14,72 | 8,74 | 7,42 | 1,55 | 0,0557 | 7,26 |
| 2 | SFB1 | 0,728 | 13,88 | 7,72 | 7,69 | 2,17 | 0,0609 | 8,37 |
| 2 | SFB1 | 0,554 | 12,22 | 8,37 | 7,82 | 1,30 | 0,0468 | 8,45 |
| 2 | SFB1 | 0,757 | 13,91 | 8,63 | 6,34 | 1,47 | 0,0471 | 6,22 |
| 2 | SFB1 | 0,661 | 12,58 | 7,70 | 6,75 | 1,10 | 0,0468 | 7,08 |
| 2 | SFB1 | 0,728 | 10,07 | 9,08 | 5,75 | 1,06 | 0,0624 | 8,57 |
| 2 | SFB1 | 0,701 | 11,69 | 9,46 | 5,39 | 2,30 | 0,0464 | 6,62 |
| 2 | SFB1 | 0,491 | 11,88 | 8,76 | 6,34 | 2,15 | 0,0359 | 7,31 |
| 2 | SFB1 | 0,804 | 13,36 | 9,72 | 6,72 | 1,36 | 0,0384 | 4,77 |
| 2 | SFB1 | 0,710 | 13,30 | 7,40 | 6,64 | 1,40 | 0,0415 | 5,85 |
| 2 | SFB1 | 0,701 | 12,13 | 8,98 | 6,12 | 1,29 | 0,0380 | 5,42 |
| 2 | SFB1 | 0,541 | 12,15 | 6,20 | 6,03 | 2,00 | 0,0554 | 10,24 |
| 2 | SFB1 | 0,391 | 9,62 | 6,67 | 4,46 | 1,02 | 0,0227 | 5,81 |
| 2 | SFB1 | 0,648 | 11,56 | 6,85 | 5,63 | 2,24 | 0,0295 | 4,55 |
| 2 | SFB1 | 0,537 | 13,28 | 5,36 | 6,90 | 2,98 | 0,0431 | 8,03 |
| 2 | SFB1 | 0,584 | 12,10 | 7,52 | 5,22 | 1,69 | 0,0350 | 5,99 |
| 2 | SFB1 | 0,397 | 8,78 | 5,30 | 6,26 | 1,47 | 0,0350 | 8,83 |
| 2 | SFB1 | 0,603 | 14,39 | 6,52 | 6,68 | 1,76 | 0,0371 | 6,16 |
| 2 | SFB1 | 0,513 | 14,34 | 8,23 | 7,51 | 1,94 | 0,0551 | 10,73 |
| 2 | SFB1 | 0,505 | 11,74 | 7,76 | 5,12 | 2,55 | 0,0448 | 8,86 |
| 2 | SFB1 | 0,694 | 13,32 | 7,35 | 6,31 | 1,84 | 0,0536 | 7,73 |
| 2 | SFB1 | 0,492 | 9,73 | 6,52 | 6,18 | 1,51 | 0,0426 | 8,66 |
| 2 | SFB1 | 0,499 | 11,20 | 7,28 | 5,49 | 1,15 | 0,0346 | 6,93 |
| 2 | PTO2 | 0,680 | 15,69 | 7,68 | 11,63 | 3,63 | 0,0781 | 11,49 |
| 2 | PTO2 | 0,633 | 12,40 | 9,22 | 6,71 | 1,48 | 0,0991 | 15,65 |
| 2 | PTO2 | 0,695 | 12,51 | 6,62 | 9,89 | 2,44 | 0,0876 | 12,60 |
| 2 | PTO2 | 0,638 | 13,51 | 4,49 | 14,63 | 4,23 | 0,0640 | 10,03 |
| 2 | PTO2 | 0,734 | 14,68 | 5,60 | 13,14 | 4,24 | 0,0945 | 12,88 |
| 2 | PTO2 | 0,709 | 12,14 | 6,20 | 11,03 | 3,18 | 0,0968 | 13,66 |
| 2 | PTO2 | 0,651 | 15,35 | 9,61 | 8,31 | 1,23 | 0,0978 | 15,02 |
| 2 | PTO2 | 0,677 | 12,28 | 5,03 | 7,80 | 1,40 | 0,0868 | 12,82 |
| 2 | PTO2 | 0,677 | 8,73 | 5,01 | 6,70 | 2,48 | 0,0949 | 14,02 |
| 2 | PTO2 | 0,541 | 13,02 | 6,68 | 9,68 | 4,20 | 0,0989 | 18,27 |
| 2 | PTO2 | 0,374 | 12,30 | 5,58 | 10,98 | 1,22 | 0,0709 | 18,96 |
| 2 | PTO2 | 0,628 | 11,45 | 2,61 | 13,68 | 2,14 | 0,0689 | 10,97 |
| 2 | PTO2 | 0,648 | 13,20 | 8,02 | 8,78 | 3,10 | 0,0649 | 10,02 |
| 2 | PTO2 | 0,632 | 13,19 | 5,79 | 9,68 | 2,76 | 0,0708 | 11,20 |
| 2 | PTO2 | 0,666 | 14,28 | 5,56 | 7,07 | 1,40 | 0,0914 | 13,72 |
| 2 | PTO2 | 0,687 | 12,50 | 4,98 | 8,68 | 3,40 | 0,0879 | 12,79 |
| 2 | PTO2 | 0,718 | 13,20 | 5,03 | 8,31 | 1,50 | 0,0784 | 10,92 |
| 2 | PTO2 | 0,769 | 14,81 | 6,03 | 8,76 | 1,68 | 0,0768 | 9,99 |
| 2 | PTO2 | 0,646 | 12,40 | 6,10 | 9,49 | 2,14 | 0,0948 | 14,67 |
| 2 | PTO2 | 0,657 | 11,29 | 7,69 | 7,09 | 1,78 | 0,0848 | 12,91 |
| 2 | PTO2 | 0,784 | 12,09 | 8,34 | 8,78 | 2,14 | 0,0867 | 11,06 |
| 2 | PTO2 | 0,656 | 11,78 | 6,03 | 8,74 | 3,16 | 0,0720 | 10,98 |
| 2 | PTO2 | 0,646 | 8,43 | 3,03 | 9,16 | 2,18 | 0,0819 | 12,68 |
| 2 | PTO2 | 0,516 | 12,04 | 7,52 | 8,89 | 1,28 | 0,0876 | 16,98 |
| 2 | PTO2 | 0,406 | 13,60 | 6,80 | 10,38 | 1,34 | 0,0768 | 18,92 |
| 2 | PTO3 | 0,733 | 14,61 | 4,68 | 11,20 | 1,85 | 0,0989 | 13,49 |
| 2 | PTO3 | 0,804 | 13,60 | 4,95 | 9,14 | 1,97 | 0,0864 | 10,75 |
| 2 | PTO3 | 0,791 | 12,70 | 6,14 | 7,74 | 1,29 | 0,0914 | 11,55 |
| 2 | PTO3 | 0,662 | 11,60 | 5,89 | 6,14 | 2,39 | 0,0713 | 10,78 |
| 2 | PTO3 | 0,671 | 13,20 | 4,40 | 8,18 | 1,94 | 0,0624 | 9,30 |
| 2 | PTO3 | 0,598 | 11,03 | 6,30 | 11,23 | 2,98 | 0,0562 | 9,40 |
| 2 | PTO3 | 0,541 | 10,55 | 3,20 | 7,41 | 1,93 | 0,0718 | 13,26 |
| 2 | PTO3 | 0,602 | 9,89 | 5,14 | 10,54 | 3,68 | 0,0624 | 10,37 |
| 2 | PTO3 | 0,595 | 13,14 | 5,14 | 10,60 | 2,14 | 0,0940 | 15,81 |
| 2 | PTO3 | 0,667 | 12,58 | 4,14 | 11,38 | 1,74 | 0,0870 | 13,05 |
| 2 | PTO3 | 0,677 | 11,70 | 3,89 | 5,17 | 1,40 | 0,0948 | 14,00 |
| 2 | PTO3 | 0,701 | 12,60 | 4,30 | 12,10 | 2,40 | 0,0768 | 10,95 |
| 2 | PTO3 | 0,698 | 11,40 | 6,68 | 8,40 | 1,20 | 0,0614 | 8,80 |
| 2 | PTO3 | 0,581 | 13,60 | 5,14 | 7,79 | 2,19 | 0,0945 | 16,25 |
| 2 | PTO3 | 0,722 | 11,78 | 6,89 | 8,34 | 1,20 | 0,0869 | 12,04 |
| 2 | PTO3 | 0,447 | 14,68 | 4,14 | 7,14 | 1,19 | 0,0913 | 20,44 |
| 2 | PTO3 | 0,531 | 12,40 | 6,10 | 10,20 | 1,15 | 0,0865 | 16,28 |
| 2 | PTO3 | 0,815 | 13,40 | 5,10 | 9,74 | 2,64 | 0,0950 | 11,66 |
| 2 | PTO3 | 0,711 | 12,02 | 5,18 | 10,14 | 1,89 | 0,0745 | 10,48 |
| 2 | PTO3 | 0,668 | 13,48 | 6,58 | 5,64 | 1,64 | 0,0610 | 9,13 |
| 2 | PTO3 | 0,586 | 8,76 | 5,68 | 14,40 | 3,60 | 0,0590 | 10,06 |
| 2 | PTO3 | 0,567 | 11,49 | 6,13 | 10,78 | 2,68 | 0,0615 | 10,84 |
| 2 | PTO3 | 0,327 | 10,49 | 3,39 | 11,20 | 1,45 | 0,0123 | 3,76 |
| 2 | PTO3 | 0,145 | 11,20 | 3,40 | 7,34 | 2,03 | 0,0113 | 7,79 |
| 2 | PTO3 | 0,158 | 12,40 | 5,01 | 8,90 | 1,87 | 0,0105 | 6,65 |
| 2 | PTO4 | 0,702 | 10,60 | 5,49 | 10,54 | 3,80 | 0,0989 | 14,09 |
| 2 | PTO4 | 0,683 | 15,45 | 4,10 | 6,12 | 1,54 | 0,0879 | 12,87 |
| 2 | PTO4 | 0,755 | 15,16 | 5,80 | 9,78 | 4,14 | 0,0380 | 5,03 |
| 2 | PTO4 | 0,681 | 14,10 | 6,15 | 8,74 | 4,44 | 0,0689 | 10,11 |
| 2 | PTO4 | 0,702 | 12,14 | 4,08 | 12,41 | 3,14 | 0,0714 | 10,17 |
| 2 | PTO4 | 0,731 | 14,38 | 4,38 | 10,07 | 3,40 | 0,0898 | 12,28 |
| 2 | PTO4 | 0,632 | 13,08 | 5,14 | 11,56 | 2,68 | 0,0914 | 14,46 |
| 2 | PTO4 | 0,605 | 16,07 | 5,18 | 10,78 | 1,88 | 0,0838 | 13,85 |
| 2 | PTO4 | 0,368 | 15,08 | 5,10 | 12,41 | 3,39 | 0,0614 | 16,68 |
| 2 | PTO4 | 0,406 | 12,78 | 4,90 | 14,88 | 1,89 | 0,0714 | 17,59 |
| 2 | PTO4 | 0,526 | 13,22 | 7,82 | 10,52 | 2,14 | 0,0856 | 16,27 |
| 2 | PTO4 | 0,687 | 13,62 | 4,62 | 11,38 | 1,87 | 0,0914 | 13,30 |
| 2 | PTO4 | 0,711 | 14,32 | 5,89 | 10,68 | 2,14 | 0,0868 | 12,20 |
| 2 | PTO4 | 0,811 | 13,08 | 5,06 | 12,41 | 4,07 | 0,0756 | 9,32 |
| 2 | PTO4 | 0,721 | 14,74 | 4,89 | 13,06 | 1,75 | 0,0814 | 11,28 |
| 2 | PTO4 | 0,581 | 12,68 | 5,14 | 9,78 | 2,38 | 0,0918 | 15,80 |
| 2 | PTO4 | 0,481 | 12,59 | 6,50 | 8,68 | 1,34 | 0,0308 | 6,40 |
| 2 | PTO4 | 0,381 | 11,40 | 3,80 | 8,68 | 1,38 | 0,0689 | 18,07 |
| 2 | PTO4 | 0,148 | 12,23 | 7,90 | 11,20 | 2,14 | 0,0187 | 12,65 |
| 2 | PTO4 | 0,319 | 11,90 | 6,70 | 12,60 | 1,76 | 0,0287 | 9,00 |
| 2 | PTO4 | 0,682 | 10,77 | 3,48 | 11,40 | 2,70 | 0,0650 | 9,53 |
| 2 | PTO4 | 0,706 | 10,89 | 6,62 | 12,60 | 1,70 | 0,0714 | 10,11 |
| 2 | PTO4 | 0,681 | 9,18 | 3,48 | 11,58 | 2,36 | 0,0808 | 11,86 |
| 2 | PTO4 | 0,549 | 11,14 | 5,06 | 10,90 | 3,50 | 0,0915 | 16,67 |
| 2 | PTO4 | 0,638 | 12,28 | 3,18 | 8,80 | 1,75 | 0,0875 | 13,72 |
| 2 | PTO5 | 0,323 | 8,59 | 9,40 | 3,94 | 1,03 | 0,0530 | 16,41 |
| 2 | PTO5 | 0,322 | 8,24 | 4,06 | 4,13 | 0,97 | 0,0473 | 14,68 |
| 2 | PTO5 | 0,341 | 6,54 | 6,10 | 7,79 | 1,01 | 0,0475 | 13,95 |
| 2 | PTO5 | 0,131 | 7,74 | 3,14 | 5,21 | 1,21 | 0,0160 | 12,25 |
| 2 | PTO5 | 0,247 | 8,14 | 5,10 | 7,72 | 0,87 | 0,0431 | 17,46 |
| 2 | PTO5 | 0,182 | 8,28 | 3,25 | 5,25 | 1,47 | 0,0307 | 16,83 |
| 2 | PTO5 | 0,255 | 10,40 | 6,85 | 4,02 | 1,02 | 0,0430 | 16,87 |
| 2 | PTO5 | 0,318 | 11,20 | 3,40 | 6,45 | 1,80 | 0,0511 | 16,07 |
| 2 | PTO5 | 0,201 | 6,64 | 3,22 | 6,34 | 1,68 | 0,0280 | 13,90 |
| 2 | PTO5 | 0,157 | 8,65 | 6,20 | 8,01 | 1,94 | 0,0191 | 12,18 |
| 2 | PTO5 | 0,418 | 8,48 | 7,10 | 6,54 | 2,27 | 0,0610 | 14,59 |
| 2 | PTO5 | 0,143 | 7,14 | 3,10 | 3,98 | 1,63 | 0,0165 | 11,54 |
| 2 | PTO5 | 0,281 | 8,10 | 4,14 | 6,09 | 0,93 | 0,0550 | 19,57 |
| 2 | PTO5 | 0,369 | 6,93 | 5,10 | 3,99 | 1,31 | 0,0640 | 17,34 |
| 2 | PTO5 | 0,187 | 11,10 | 6,60 | 7,65 | 1,06 | 0,0284 | 15,19 |
| 2 | PTO5 | 0,322 | 12,14 | 5,30 | 7,63 | 1,03 | 0,0670 | 20,81 |
| 2 | PTO5 | 0,143 | 10,60 | 6,14 | 6,52 | 1,31 | 0,0180 | 12,61 |
| 2 | PTO5 | 0,234 | 11,20 | 3,14 | 6,31 | 1,61 | 0,0314 | 13,42 |
| 2 | PTO5 | 0,193 | 10,49 | 5,18 | 8,41 | 1,50 | 0,0198 | 10,26 |
| 2 | PTO5 | 0,243 | 7,42 | 5,10 | 7,68 | 2,03 | 0,0520 | 21,40 |
| 2 | PTO5 | 0,314 | 8,31 | 5,14 | 5,50 | 1,46 | 0,0633 | 20,16 |
| 2 | PTO5 | 0,358 | 8,58 | 4,18 | 6,39 | 1,20 | 0,0603 | 16,84 |
| 2 | PTO5 | 0,252 | 8,97 | 3,14 | 5,23 | 1,21 | 0,0539 | 21,39 |
| 2 | PTO5 | 0,374 | 7,19 | 6,14 | 3,68 | 1,25 | 0,0490 | 13,10 |
| 2 | PTO5 | 0,282 | 7,83 | 5,10 | 6,50 | 0,95 | 0,0397 | 14,09 |
| 2 | PTO6 | 0,637 | 12,13 | 5,06 | 8,31 | 2,48 | 0,0265 | 4,16 |
| 2 | PTO6 | 0,539 | 15,26 | 8,50 | 7,84 | 2,19 | 0,0306 | 5,68 |
| 2 | PTO6 | 0,595 | 13,81 | 5,51 | 7,09 | 2,00 | 0,0270 | 4,54 |
| 2 | PTO6 | 0,677 | 13,34 | 8,14 | 7,72 | 1,75 | 0,0302 | 4,46 |
| 2 | PTO6 | 0,608 | 13,54 | 8,41 | 8,00 | 2,41 | 0,0366 | 6,02 |
| 2 | PTO6 | 0,552 | 12,88 | 6,64 | 8,03 | 2,56 | 0,0337 | 6,11 |
| 2 | PTO6 | 0,689 | 13,27 | 8,14 | 7,37 | 2,54 | 0,0379 | 5,50 |
| 2 | PTO6 | 0,485 | 10,74 | 6,86 | 7,44 | 2,51 | 0,0340 | 7,01 |
| 2 | PTO6 | 0,528 | 14,11 | 6,18 | 7,60 | 2,59 | 0,0345 | 6,54 |
| 2 | PTO6 | 0,636 | 13,05 | 7,81 | 7,07 | 2,04 | 0,0321 | 5,05 |
| 2 | PTO6 | 0,676 | 13,92 | 8,75 | 9,09 | 2,16 | 0,0308 | 4,55 |
| 2 | PTO6 | 0,418 | 8,87 | 6,66 | 7,02 | 1,75 | 0,0337 | 8,07 |
| 2 | PTO6 | 0,673 | 14,00 | 8,91 | 7,69 | 2,16 | 0,0259 | 3,85 |
| 2 | PTO6 | 0,604 | 11,67 | 8,22 | 6,20 | 3,08 | 0,0382 | 6,33 |
| 2 | PTO6 | 0,529 | 13,28 | 7,63 | 8,26 | 2,16 | 0,0327 | 6,18 |
| 2 | PTO6 | 0,462 | 11,20 | 7,52 | 7,56 | 2,66 | 0,0520 | 11,27 |
| 2 | PTO6 | 0,614 | 13,16 | 8,25 | 7,49 | 2,24 | 0,0344 | 5,61 |
| 2 | PTO6 | 0,357 | 12,52 | 6,02 | 7,41 | 2,83 | 0,0346 | 9,71 |
| 2 | PTO6 | 0,396 | 13,38 | 6,41 | 8,72 | 1,78 | 0,0332 | 8,39 |
| 2 | PTO6 | 0,396 | 11,86 | 6,37 | 7,45 | 2,51 | 0,0339 | 8,57 |
| 2 | PTO6 | 0,489 | 11,81 | 7,14 | 8,06 | 2,59 | 0,0445 | 9,10 |
| 2 | PTO6 | 0,669 | 14,31 | 8,19 | 8,19 | 2,12 | 0,0287 | 4,29 |
| 2 | PTO6 | 0,506 | 12,10 | 7,14 | 8,75 | 1,69 | 0,0343 | 6,78 |
| 2 | PTO6 | 0,641 | 15,03 | 7,48 | 8,55 | 1,95 | 0,0398 | 6,21 |
| 2 | PTO6 | 0,512 | 14,16 | 7,51 | 7,90 | 1,83 | 0,0348 | 6,80 |
| 2 | PTO7 | 0,505 | 9,88 | 7,61 | 6,72 | 1,71 | 0,0421 | 8,34 |
| 2 | PTO7 | 0,310 | 11,03 | 6,21 | 6,26 | 2,32 | 0,0361 | 11,64 |
| 2 | PTO7 | 0,448 | 12,33 | 9,11 | 7,31 | 1,84 | 0,0430 | 9,59 |
| 2 | PTO7 | 0,649 | 13,00 | 8,52 | 6,83 | 2,93 | 0,0439 | 6,76 |
| 2 | PTO7 | 0,424 | 12,11 | 6,67 | 6,30 | 1,98 | 0,0304 | 7,17 |
| 2 | PTO7 | 0,601 | 13,87 | 8,81 | 7,71 | 3,03 | 0,0417 | 6,94 |
| 2 | PTO7 | 0,498 | 13,07 | 7,50 | 7,95 | 1,75 | 0,0323 | 6,48 |
| 2 | PTO7 | 0,712 | 12,61 | 8,37 | 6,58 | 2,48 | 0,0413 | 5,80 |
| 2 | PTO7 | 0,570 | 12,75 | 7,48 | 6,52 | 2,00 | 0,0365 | 6,40 |
| 2 | PTO7 | 0,445 | 12,86 | 4,35 | 6,68 | 1,87 | 0,0336 | 7,54 |
| 2 | PTO7 | 0,458 | 11,93 | 6,64 | 6,95 | 2,23 | 0,0282 | 6,16 |
| 2 | PTO7 | 0,506 | 12,48 | 6,20 | 6,72 | 2,58 | 0,0461 | 9,11 |
| 2 | PTO7 | 0,613 | 11,86 | 7,40 | 6,90 | 2,09 | 0,0459 | 7,49 |
| 2 | PTO7 | 0,348 | 10,27 | 5,67 | 5,72 | 2,71 | 0,0342 | 9,83 |
| 2 | PTO7 | 0,503 | 11,43 | 7,28 | 7,56 | 2,52 | 0,0429 | 8,53 |
| 2 | PTO7 | 0,745 | 13,74 | 8,46 | 6,95 | 1,83 | 0,0339 | 4,55 |
| 2 | PTO7 | 0,653 | 13,20 | 8,31 | 7,50 | 2,63 | 0,0428 | 6,55 |
| 2 | PTO7 | 0,653 | 13,10 | 8,92 | 6,93 | 1,99 | 0,0294 | 4,50 |
| 2 | PTO7 | 0,644 | 14,32 | 9,14 | 7,09 | 2,28 | 0,0391 | 6,07 |
| 2 | PTO7 | 0,509 | 10,47 | 7,11 | 6,41 | 2,24 | 0,0320 | 6,29 |
| 2 | PTO7 | 0,288 | 9,98 | 5,74 | 7,44 | 1,74 | 0,0315 | 10,93 |
| 2 | PTO7 | 0,569 | 11,60 | 7,74 | 6,63 | 2,07 | 0,0431 | 7,57 |
| 2 | PTO7 | 0,320 | 9,57 | 6,03 | 5,19 | 2,53 | 0,0285 | 8,90 |
| 2 | PTO7 | 0,338 | 9,86 | 6,73 | 6,47 | 2,16 | 0,0277 | 8,20 |
| 2 | PTO7 | 0,331 | 11,01 | 6,22 | 6,61 | 1,72 | 0,0225 | 6,80 |
| 2 | PTO8 | 0,615 | 10,00 | 7,32 | 5,73 | 1,21 | 0,0470 | 7,64 |
| 2 | PTO8 | 0,593 | 12,08 | 6,71 | 7,30 | 1,22 | 0,0521 | 8,79 |
| 2 | PTO8 | 0,657 | 11,21 | 5,33 | 5,62 | 2,34 | 0,0618 | 9,40 |
| 2 | PTO8 | 0,733 | 10,53 | 7,36 | 5,85 | 1,08 | 0,0436 | 5,95 |
| 2 | PTO8 | 0,743 | 13,56 | 6,16 | 8,10 | 2,66 | 0,0471 | 6,34 |
| 2 | PTO8 | 0,741 | 10,92 | 8,24 | 7,24 | 1,48 | 0,0518 | 6,99 |
| 2 | PTO8 | 0,759 | 13,64 | 5,87 | 6,90 | 1,82 | 0,0536 | 7,06 |
| 2 | PTO8 | 0,771 | 13,63 | 8,11 | 6,87 | 1,98 | 0,0576 | 7,47 |
| 2 | PTO8 | 0,604 | 11,78 | 6,52 | 5,72 | 2,04 | 0,0522 | 8,64 |
| 2 | PTO8 | 0,642 | 10,28 | 9,09 | 6,95 | 0,99 | 0,0598 | 9,32 |
| 2 | PTO8 | 0,670 | 11,13 | 6,97 | 5,79 | 1,49 | 0,0678 | 10,13 |
| 2 | PTO8 | 0,631 | 11,67 | 6,78 | 5,31 | 1,13 | 0,0539 | 8,54 |
| 2 | PTO8 | 0,694 | 10,83 | 6,40 | 5,61 | 0,82 | 0,0498 | 7,18 |
| 2 | PTO8 | 0,599 | 9,41 | 6,47 | 6,74 | 3,09 | 0,0567 | 9,47 |
| 2 | PTO8 | 0,676 | 11,16 | 6,90 | 6,98 | 2,53 | 0,0476 | 7,04 |
| 2 | PTO8 | 0,529 | 11,17 | 6,45 | 6,19 | 1,29 | 0,0533 | 10,08 |
| 2 | PTO8 | 0,542 | 10,95 | 5,44 | 3,46 | 1,51 | 0,0671 | 12,38 |
| 2 | PTO8 | 0,810 | 13,78 | 7,33 | 5,88 | 1,30 | 0,0652 | 8,05 |
| 2 | PTO8 | 0,780 | 14,03 | 6,61 | 6,88 | 1,24 | 0,0640 | 8,21 |
| 2 | PTO8 | 0,756 | 13,21 | 5,62 | 4,68 | 1,19 | 0,0645 | 8,53 |
| 2 | PTO8 | 0,786 | 11,73 | 6,46 | 5,33 | 1,24 | 0,0544 | 6,92 |
| 2 | PTO8 | 0,765 | 9,84 | 7,80 | 5,55 | 1,26 | 0,0562 | 7,35 |
| 2 | PTO8 | 0,673 | 11,22 | 8,46 | 6,25 | 1,45 | 0,0646 | 9,59 |
| 2 | PTO8 | 0,679 | 12,69 | 6,51 | 7,48 | 1,30 | 0,0587 | 8,65 |
| 2 | PTO8 | 0,592 | 11,10 | 5,20 | 6,02 | 1,40 | 0,0533 | 9,00 |
| 2 | PTO9 | 0,409 | 9,87 | 5,59 | 6,15 | 1,26 | 0,0603 | 14,74 |
| 2 | PTO9 | 0,398 | 14,03 | 6,25 | 6,62 | 0,80 | 0,0611 | 15,35 |
| 2 | PTO9 | 0,414 | 9,07 | 6,90 | 5,38 | 0,88 | 0,0615 | 14,86 |
| 2 | PTO9 | 0,434 | 7,82 | 4,67 | 5,25 | 0,79 | 0,0608 | 14,01 |
| 2 | PTO9 | 0,608 | 9,37 | 4,75 | 5,54 | 1,19 | 0,0718 | 11,81 |
| 2 | PTO9 | 0,359 | 8,98 | 4,65 | 5,21 | 1,06 | 0,0480 | 13,37 |
| 2 | PTO9 | 0,423 | 8,44 | 4,76 | 5,48 | 0,99 | 0,0537 | 12,70 |
| 2 | PTO9 | 0,393 | 9,22 | 5,11 | 5,41 | 1,17 | 0,0439 | 11,17 |
| 2 | PTO9 | 0,376 | 10,48 | 5,03 | 7,14 | 1,36 | 0,0382 | 10,16 |
| 2 | PTO9 | 0,474 | 9,63 | 5,49 | 5,94 | 1,64 | 0,0390 | 8,23 |
| 2 | PTO9 | 0,612 | 10,96 | 4,59 | 7,40 | 0,95 | 0,0523 | 8,55 |
| 2 | PTO9 | 0,377 | 9,67 | 4,93 | 6,21 | 0,86 | 0,0508 | 13,47 |
| 2 | PTO9 | 0,465 | 12,44 | 6,01 | 4,95 | 1,32 | 0,0458 | 9,85 |
| 2 | PTO9 | 0,481 | 9,90 | 4,58 | 5,37 | 1,11 | 0,0458 | 9,52 |
| 2 | PTO9 | 0,388 | 9,28 | 5,23 | 6,18 | 1,27 | 0,0510 | 13,14 |
| 2 | PTO9 | 0,427 | 8,60 | 5,12 | 6,60 | 1,14 | 0,0519 | 12,15 |
| 2 | PTO9 | 0,629 | 13,58 | 5,60 | 7,60 | 0,78 | 0,0507 | 8,06 |
| 2 | PTO9 | 0,549 | 13,90 | 4,96 | 6,41 | 0,87 | 0,0715 | 13,02 |
| 2 | PTO9 | 0,577 | 10,80 | 5,57 | 5,47 | 0,83 | 0,0610 | 10,57 |
| 2 | PTO9 | 0,502 | 9,50 | 5,69 | 6,14 | 0,62 | 0,0615 | 12,25 |
| 2 | PTO9 | 0,577 | 9,53 | 3,86 | 6,21 | 0,81 | 0,0511 | 8,86 |
| 2 | PTO9 | 0,549 | 8,35 | 5,44 | 5,35 | 1,70 | 0,0550 | 10,02 |
| 2 | PTO9 | 0,527 | 7,78 | 3,47 | 5,91 | 1,02 | 0,0520 | 9,87 |
| 2 | PTO9 | 0,580 | 9,54 | 5,40 | 5,89 | 0,90 | 0,0490 | 8,45 |
| 2 | PTO9 | 0,480 | 8,66 | 5,11 | 5,53 | 0,13 | 0,0397 | 8,27 |
| 2 | PDO1 | 0,735 | 17,05 | 9,05 | 9,27 | 2,33 | 0,0514 | 6,99 |
| 2 | PDO1 | 0,468 | 14,70 | 7,13 | 9,01 | 2,35 | 0,0439 | 9,38 |
| 2 | PDO1 | 0,689 | 14,57 | 7,14 | 9,20 | 2,25 | 0,0791 | 11,48 |
| 2 | PDO1 | 0,679 | 15,10 | 6,68 | 8,20 | 2,10 | 0,0512 | 7,54 |
| 2 | PDO1 | 0,603 | 13,22 | 9,61 | 7,45 | 2,53 | 0,0621 | 10,30 |
| 2 | PDO1 | 0,518 | 14,38 | 9,01 | 6,19 | 1,79 | 0,0650 | 12,55 |
| 2 | PDO1 | 0,562 | 13,49 | 9,10 | 9,64 | 2,62 | 0,1000 | 17,79 |
| 2 | PDO1 | 0,653 | 12,19 | 7,72 | 8,21 | 1,98 | 0,0700 | 10,72 |
| 2 | PDO1 | 0,604 | 12,24 | 6,39 | 8,42 | 2,39 | 0,0800 | 13,25 |
| 2 | PDO1 | 0,729 | 16,35 | 6,47 | 9,46 | 2,81 | 0,0750 | 10,29 |
| 2 | PDO1 | 0,753 | 15,22 | 8,30 | 8,53 | 2,21 | 0,0580 | 7,70 |
| 2 | PDO1 | 0,510 | 13,50 | 6,10 | 9,23 | 2,12 | 0,0545 | 10,69 |
| 2 | PDO1 | 0,441 | 13,68 | 7,20 | 8,21 | 2,01 | 0,0782 | 17,73 |
| 2 | PDO1 | 0,523 | 11,70 | 8,01 | 9,01 | 1,18 | 0,0746 | 14,26 |
| 2 | PDO1 | 0,618 | 14,02 | 6,78 | 7,20 | 1,15 | 0,0547 | 8,85 |
| 2 | PDO1 | 0,465 | 12,35 | 6,21 | 8,44 | 2,03 | 0,0526 | 11,31 |
| 2 | PDO1 | 0,635 | 17,20 | 6,70 | 9,73 | 2,02 | 0,0540 | 8,50 |
| 2 | PDO1 | 0,450 | 13,38 | 6,31 | 8,39 | 2,73 | 0,0820 | 18,22 |
| 2 | PDO1 | 0,791 | 17,80 | 7,40 | 9,78 | 2,25 | 0,0535 | 6,76 |
| 2 | PDO1 | 0,472 | 15,20 | 6,30 | 8,25 | 1,76 | 0,0710 | 15,04 |
| 2 | PDO1 | 0,612 | 15,80 | 7,30 | 7,55 | 1,28 | 0,0602 | 9,84 |
| 2 | PDO1 | 0,715 | 15,10 | 14,20 | 7,81 | 1,31 | 0,0540 | 7,55 |
| 2 | PDO1 | 0,713 | 14,66 | 7,72 | 7,91 | 1,82 | 0,0759 | 10,65 |
| 2 | PDO1 | 0,643 | 13,20 | 8,33 | 8,32 | 1,27 | 0,0703 | 10,93 |
| 2 | PDO1 | 0,699 | 14,43 | 6,79 | 9,02 | 1,61 | 0,0810 | 11,59 |
| 2 | PDO2 | 0,311 | 9,09 | 5,96 | 6,66 | 2,03 | 0,0601 | 19,32 |
| 2 | PDO2 | 0,365 | 10,56 | 5,51 | 7,47 | 2,94 | 0,0578 | 15,84 |
| 2 | PDO2 | 0,522 | 11,86 | 9,78 | 7,90 | 2,41 | 0,0447 | 8,56 |
| 2 | PDO2 | 0,343 | 6,55 | 5,25 | 6,87 | 2,36 | 0,0558 | 16,27 |
| 2 | PDO2 | 0,286 | 8,34 | 4,92 | 7,92 | 2,26 | 0,0490 | 17,13 |
| 2 | PDO2 | 0,298 | 9,00 | 6,60 | 7,31 | 2,35 | 0,0470 | 15,77 |
| 2 | PDO2 | 0,457 | 11,26 | 8,15 | 8,29 | 2,44 | 0,0770 | 16,85 |
| 2 | PDO2 | 0,227 | 9,57 | 4,14 | 5,92 | 2,49 | 0,0420 | 18,50 |
| 2 | PDO2 | 0,170 | 9,24 | 4,08 | 7,96 | 1,97 | 0,0370 | 21,76 |
| 2 | PDO2 | 0,288 | 9,24 | 6,42 | 6,30 | 2,14 | 0,0580 | 20,14 |
| 2 | PDO2 | 0,349 | 11,08 | 7,21 | 7,88 | 1,91 | 0,0420 | 12,03 |
| 2 | PDO2 | 0,388 | 10,30 | 5,44 | 7,43 | 3,01 | 0,0650 | 16,75 |
| 2 | PDO2 | 0,449 | 11,08 | 7,07 | 6,98 | 2,95 | 0,0630 | 14,03 |
| 2 | PDO2 | 0,447 | 10,82 | 6,28 | 6,97 | 2,73 | 0,0790 | 17,67 |
| 2 | PDO2 | 0,378 | 11,19 | 6,87 | 7,90 | 2,49 | 0,0730 | 19,31 |
| 2 | PDO2 | 0,332 | 10,40 | 6,37 | 7,73 | 1,99 | 0,0560 | 16,87 |
| 2 | PDO2 | 0,301 | 10,13 | 6,37 | 7,39 | 1,78 | 0,0640 | 21,26 |
| 2 | PDO2 | 0,337 | 10,12 | 6,59 | 6,82 | 2,36 | 0,0590 | 17,51 |
| 2 | PDO2 | 0,227 | 9,45 | 6,12 | 6,03 | 3,30 | 0,0315 | 13,86 |
| 2 | PDO2 | 0,247 | 9,62 | 7,39 | 7,30 | 2,41 | 0,0390 | 15,79 |
| 2 | PDO2 | 0,235 | 9,35 | 8,50 | 7,29 | 2,01 | 0,0182 | 7,74 |
| 2 | PDO2 | 0,165 | 7,42 | 5,96 | 5,70 | 2,70 | 0,0162 | 9,82 |
| 2 | PDO2 | 0,254 | 9,77 | 6,82 | 7,71 | 2,27 | 0,0440 | 17,32 |
| 2 | PDO2 | 0,437 | 10,89 | 8,37 | 7,08 | 2,86 | 0,0620 | 14,19 |
| 2 | PDO2 | 0,406 | 10,66 | 7,93 | 5,74 | 2,43 | 0,0620 | 15,27 |
| 2 | CRO1 | 0,618 | 12,38 | 8,46 | 5,77 | 1,19 | 0,0586 | 9,48 |
| 2 | CRO1 | 0,467 | 10,47 | 7,47 | 7,26 | 2,13 | 0,0633 | 13,55 |
| 2 | CRO1 | 0,583 | 11,16 | 7,42 | 8,13 | 1,03 | 0,0556 | 9,54 |
| 2 | CRO1 | 0,577 | 14,18 | 7,13 | 7,89 | 0,99 | 0,0479 | 8,30 |
| 2 | CRO1 | 0,598 | 14,62 | 6,65 | 6,67 | 0,97 | 0,0524 | 8,76 |
| 2 | CRO1 | 0,441 | 11,98 | 8,13 | 6,55 | 0,90 | 0,0498 | 11,29 |
| 2 | CRO1 | 0,502 | 10,22 | 5,66 | 6,44 | 1,12 | 0,0453 | 9,02 |
| 2 | CRO1 | 0,634 | 13,79 | 7,74 | 6,74 | 0,97 | 0,0773 | 12,19 |
| 2 | CRO1 | 0,734 | 14,01 | 8,33 | 7,66 | 1,84 | 0,0752 | 10,25 |
| 2 | CRO1 | 0,585 | 10,22 | 7,00 | 5,13 | 1,96 | 0,0535 | 9,15 |
| 2 | CRO1 | 0,653 | 12,19 | 7,16 | 8,67 | 1,17 | 0,0601 | 9,20 |
| 2 | CRO1 | 0,656 | 12,89 | 7,32 | 5,72 | 2,67 | 0,0706 | 10,76 |
| 2 | CRO1 | 0,603 | 11,57 | 7,47 | 6,78 | 2,99 | 0,0473 | 7,84 |
| 2 | CRO1 | 0,688 | 13,32 | 8,40 | 6,41 | 1,87 | 0,0513 | 7,46 |
| 2 | CRO1 | 0,507 | 10,95 | 5,32 | 7,48 | 1,59 | 0,0802 | 15,82 |
| 2 | CRO1 | 0,678 | 13,48 | 7,67 | 4,65 | 1,83 | 0,0598 | 8,82 |
| 2 | CRO1 | 0,479 | 12,04 | 7,90 | 5,95 | 1,67 | 0,0573 | 11,96 |
| 2 | CRO1 | 0,384 | 10,74 | 6,48 | 5,97 | 1,16 | 0,0394 | 10,26 |
| 2 | CRO1 | 0,629 | 12,15 | 7,46 | 5,84 | 1,44 | 0,0538 | 8,55 |
| 2 | CRO1 | 0,832 | 12,96 | 8,88 | 5,32 | 2,62 | 0,0793 | 9,53 |
| 2 | CRO1 | 0,796 | 12,92 | 8,49 | 6,35 | 1,58 | 0,0773 | 9,71 |
| 2 | CRO1 | 0,544 | 11,15 | 9,73 | 6,30 | 2,58 | 0,0792 | 14,56 |
| 2 | CRO1 | 0,549 | 10,98 | 7,73 | 6,12 | 1,86 | 0,0745 | 13,57 |
| 2 | CRO1 | 0,623 | 12,22 | 8,48 | 6,56 | 3,45 | 0,0742 | 11,91 |
| 2 | CRO1 | 0,691 | 11,09 | 7,57 | 5,36 | 1,51 | 0,0793 | 11,48 |
| 2 | CRO2 | 0,695 | 11,87 | 4,96 | 7,69 | 2,11 | 0,0601 | 8,65 |
| 2 | CRO2 | 0,698 | 11,78 | 5,46 | 6,70 | 2,36 | 0,0603 | 8,64 |
| 2 | CRO2 | 0,691 | 12,55 | 7,85 | 7,59 | 1,87 | 0,0688 | 9,96 |
| 2 | CRO2 | 0,560 | 11,25 | 7,80 | 6,53 | 2,14 | 0,0502 | 8,96 |
| 2 | CRO2 | 0,680 | 12,57 | 6,64 | 8,04 | 1,34 | 0,0602 | 8,85 |
| 2 | CRO2 | 0,596 | 10,11 | 6,37 | 6,47 | 1,64 | 0,0613 | 10,29 |
| 2 | CRO2 | 0,513 | 11,44 | 5,40 | 6,08 | 2,07 | 0,0500 | 9,75 |
| 2 | CRO2 | 0,697 | 13,00 | 6,91 | 6,96 | 2,39 | 0,0612 | 8,78 |
| 2 | CRO2 | 0,588 | 13,06 | 6,79 | 8,27 | 2,11 | 0,0721 | 12,26 |
| 2 | CRO2 | 0,689 | 13,04 | 6,48 | 6,49 | 1,92 | 0,0730 | 10,60 |
| 2 | CRO2 | 0,558 | 12,47 | 6,69 | 7,26 | 2,24 | 0,0611 | 10,95 |
| 2 | CRO2 | 0,663 | 12,02 | 5,68 | 7,26 | 1,43 | 0,0710 | 10,71 |
| 2 | CRO2 | 0,418 | 10,13 | 5,68 | 7,18 | 1,60 | 0,0615 | 14,71 |
| 2 | CRO2 | 0,668 | 12,67 | 6,07 | 6,70 | 1,01 | 0,0671 | 10,04 |
| 2 | CRO2 | 0,778 | 12,08 | 7,27 | 6,55 | 1,79 | 0,0598 | 7,69 |
| 2 | CRO2 | 0,514 | 8,36 | 6,97 | 5,37 | 1,36 | 0,0658 | 12,80 |
| 2 | CRO2 | 0,630 | 11,25 | 4,90 | 7,96 | 2,02 | 0,0633 | 10,05 |
| 2 | CRO2 | 0,580 | 11,70 | 6,97 | 5,87 | 2,16 | 0,0608 | 10,48 |
| 2 | CRO2 | 0,527 | 9,89 | 5,91 | 6,27 | 1,62 | 0,0627 | 11,90 |
| 2 | CRO2 | 0,666 | 12,16 | 6,93 | 6,78 | 1,95 | 0,0655 | 9,83 |
| 2 | CRO2 | 0,608 | 12,28 | 7,65 | 7,28 | 1,20 | 0,0726 | 11,94 |
| 2 | CRO2 | 0,673 | 12,12 | 6,60 | 6,20 | 1,85 | 0,0608 | 9,03 |
| 2 | CRO2 | 0,746 | 13,10 | 7,05 | 6,72 | 1,80 | 0,0649 | 8,70 |
| 2 | CRO2 | 0,558 | 12,16 | 4,92 | 6,76 | 1,75 | 0,0718 | 12,87 |
| 2 | CRO2 | 0,480 | 11,89 | 5,22 | 8,02 | 2,00 | 0,0653 | 13,60 |
| 2 | ADO4 | 0,584 | 12,94 | 6,93 | 7,04 | 2,42 | 0,0495 | 8,48 |
| 2 | ADO4 | 0,372 | 7,22 | 5,78 | 5,92 | 1,92 | 0,0444 | 11,94 |
| 2 | ADO4 | 0,463 | 12,86 | 7,42 | 8,26 | 2,01 | 0,0507 | 10,96 |
| 2 | ADO4 | 0,419 | 12,77 | 7,25 | 6,51 | 3,20 | 0,0451 | 10,77 |
| 2 | ADO4 | 0,624 | 14,49 | 8,04 | 7,17 | 2,54 | 0,0465 | 7,45 |
| 2 | ADO4 | 0,667 | 13,46 | 7,70 | 7,68 | 2,08 | 0,0477 | 7,15 |
| 2 | ADO4 | 0,669 | 12,73 | 8,43 | 7,31 | 2,18 | 0,0464 | 6,94 |
| 2 | ADO4 | 0,620 | 12,36 | 8,69 | 6,80 | 2,06 | 0,0520 | 8,39 |
| 2 | ADO4 | 0,618 | 13,00 | 7,72 | 7,30 | 2,48 | 0,0464 | 7,51 |
| 2 | ADO4 | 0,326 | 11,78 | 5,84 | 8,58 | 2,01 | 0,0406 | 12,47 |
| 2 | ADO4 | 0,596 | 12,64 | 7,32 | 7,87 | 1,44 | 0,0519 | 8,71 |
| 2 | ADO4 | 0,701 | 12,09 | 6,73 | 7,38 | 1,58 | 0,0397 | 5,66 |
| 2 | ADO4 | 0,503 | 11,03 | 6,81 | 7,11 | 2,49 | 0,0388 | 7,72 |
| 2 | ADO4 | 0,500 | 10,77 | 7,12 | 6,45 | 2,40 | 0,0430 | 8,61 |
| 2 | ADO4 | 0,536 | 11,77 | 7,16 | 7,41 | 1,66 | 0,0353 | 6,59 |
| 2 | ADO4 | 0,432 | 14,55 | 6,12 | 7,58 | 3,41 | 0,0393 | 9,11 |
| 2 | ADO4 | 0,564 | 11,40 | 8,66 | 5,55 | 2,17 | 0,0317 | 5,62 |
| 2 | ADO4 | 0,422 | 11,95 | 6,92 | 6,67 | 2,70 | 0,0287 | 6,80 |
| 2 | ADO4 | 0,509 | 11,26 | 6,37 | 5,89 | 1,88 | 0,0303 | 5,95 |
| 2 | ADO4 | 0,487 | 11,95 | 6,02 | 7,84 | 2,35 | 0,0445 | 9,14 |
| 2 | ADO4 | 0,686 | 12,10 | 7,86 | 6,95 | 2,10 | 0,0449 | 6,55 |
| 2 | ADO4 | 0,534 | 12,50 | 8,52 | 7,45 | 2,62 | 0,0352 | 6,60 |
| 2 | ADO4 | 0,365 | 10,83 | 6,91 | 6,25 | 1,60 | 0,0439 | 12,03 |
| 2 | ADO4 | 0,621 | 11,70 | 9,00 | 6,75 | 2,46 | 0,0324 | 5,22 |
| 2 | ADO4 | 0,481 | 14,13 | 7,43 | 8,29 | 1,61 | 0,0341 | 7,09 |
| 2 | BO1 | 0,218 | 8,88 | 3,55 | 6,61 | 1,98 | 0,0409 | 18,74 |
| 2 | BO1 | 0,336 | 10,43 | 8,20 | 4,50 | 2,04 | 0,0229 | 6,82 |
| 2 | BO1 | 0,368 | 12,89 | 7,32 | 5,55 | 1,67 | 0,0340 | 9,25 |
| 2 | BO1 | 0,363 | 11,17 | 7,33 | 6,10 | 2,43 | 0,0216 | 5,95 |
| 2 | BO1 | 0,331 | 11,53 | 6,68 | 7,90 | 2,30 | 0,0180 | 5,44 |
| 2 | BO1 | 0,385 | 10,81 | 7,44 | 6,70 | 2,03 | 0,0540 | 14,01 |
| 2 | BO1 | 0,338 | 11,09 | 7,76 | 5,53 | 1,92 | 0,0520 | 15,40 |
| 2 | BO1 | 0,254 | 9,95 | 7,15 | 6,27 | 1,94 | 0,0140 | 5,51 |
| 2 | BO1 | 0,256 | 9,85 | 6,43 | 9,44 | 2,56 | 0,0240 | 9,38 |
| 2 | BO1 | 0,381 | 8,27 | 6,02 | 8,31 | 2,40 | 0,0395 | 10,38 |
| 2 | BO1 | 0,210 | 10,06 | 5,19 | 6,97 | 2,44 | 0,0430 | 20,52 |
| 2 | BO1 | 0,224 | 10,37 | 6,06 | 6,40 | 2,37 | 0,0326 | 14,55 |
| 2 | BO1 | 0,384 | 10,53 | 6,12 | 6,29 | 2,40 | 0,0170 | 4,43 |
| 2 | BO1 | 0,314 | 10,66 | 6,15 | 8,21 | 2,15 | 0,0233 | 7,43 |
| 2 | BO1 | 0,324 | 10,08 | 5,66 | 7,50 | 3,01 | 0,0440 | 13,57 |
| 2 | BO1 | 0,167 | 10,15 | 4,56 | 7,63 | 2,50 | 0,0134 | 8,04 |
| 2 | BO1 | 0,287 | 10,69 | 4,81 | 4,10 | 3,01 | 0,0540 | 18,85 |
| 2 | BO1 | 0,289 | 10,59 | 5,59 | 8,20 | 2,50 | 0,0520 | 17,97 |
| 2 | BO1 | 0,251 | 10,78 | 6,12 | 9,63 | 1,98 | 0,0450 | 17,96 |
| 2 | BO1 | 0,242 | 10,85 | 4,19 | 8,28 | 2,78 | 0,0530 | 21,94 |
| 2 | BO1 | 0,277 | 9,54 | 6,05 | 5,91 | 2,80 | 0,0540 | 19,49 |
| 2 | BO1 | 0,361 | 10,06 | 7,02 | 7,60 | 2,30 | 0,0350 | 9,68 |
| 2 | BO1 | 0,220 | 9,70 | 6,42 | 5,96 | 2,20 | 0,0360 | 16,39 |
| 2 | BO1 | 0,240 | 12,79 | 6,02 | 6,71 | 1,40 | 0,0140 | 5,84 |
| 2 | BO1 | 0,196 | 9,32 | 6,00 | 6,80 | 1,50 | 0,0230 | 11,75 |
| 2 | ADO2 | 0,543 | 14,26 | 7,50 | 6,80 | 0,86 | 0,0443 | 8,16 |
| 2 | ADO2 | 0,538 | 13,06 | 6,54 | 6,24 | 1,14 | 0,0508 | 9,44 |
| 2 | ADO2 | 0,658 | 13,90 | 5,46 | 6,30 | 0,97 | 0,0525 | 7,98 |
| 2 | ADO2 | 0,551 | 13,63 | 7,58 | 7,17 | 0,80 | 0,0559 | 10,15 |
| 2 | ADO2 | 0,569 | 14,03 | 7,96 | 7,64 | 0,84 | 0,0480 | 8,44 |
| 2 | ADO2 | 0,688 | 15,46 | 6,85 | 7,54 | 0,83 | 0,0537 | 7,81 |
| 2 | ADO2 | 0,541 | 13,70 | 5,70 | 6,52 | 0,82 | 0,0466 | 8,61 |
| 2 | ADO2 | 0,496 | 10,20 | 9,72 | 5,79 | 1,40 | 0,0540 | 10,89 |
| 2 | ADO2 | 0,564 | 13,48 | 7,42 | 8,40 | 0,83 | 0,0634 | 11,24 |
| 2 | ADO2 | 0,556 | 12,73 | 7,78 | 6,30 | 1,25 | 0,0538 | 9,68 |
| 2 | ADO2 | 0,447 | 11,92 | 4,90 | 5,40 | 0,80 | 0,0439 | 9,82 |
| 2 | ADO2 | 0,362 | 11,60 | 4,69 | 5,61 | 1,96 | 0,0382 | 10,55 |
| 2 | ADO2 | 0,380 | 12,47 | 3,96 | 5,75 | 0,76 | 0,0390 | 10,26 |
| 2 | ADO2 | 0,697 | 13,02 | 6,91 | 6,94 | 0,84 | 0,0531 | 7,62 |
| 2 | ADO2 | 0,578 | 13,18 | 5,96 | 5,72 | 0,72 | 0,0339 | 5,87 |
| 2 | ADO2 | 0,489 | 13,02 | 5,20 | 7,04 | 0,84 | 0,0523 | 10,70 |
| 2 | ADO2 | 0,491 | 8,76 | 7,46 | 5,94 | 0,98 | 0,0394 | 8,02 |
| 2 | ADO2 | 0,666 | 12,85 | 5,84 | 5,93 | 0,73 | 0,0380 | 5,71 |
| 2 | ADO2 | 0,645 | 13,28 | 6,78 | 7,66 | 0,90 | 0,0535 | 8,29 |
| 2 | ADO2 | 0,714 | 13,92 | 5,94 | 7,10 | 1,45 | 0,0507 | 7,10 |
| 2 | ADO2 | 0,688 | 13,80 | 6,27 | 7,27 | 0,99 | 0,0446 | 6,48 |
| 2 | ADO2 | 0,664 | 13,30 | 4,43 | 7,80 | 0,78 | 0,0597 | 8,99 |
| 2 | ADO2 | 0,630 | 12,94 | 4,64 | 5,90 | 0,92 | 0,0435 | 6,90 |
| 2 | ADO2 | 0,500 | 9,42 | 5,27 | 7,28 | 0,94 | 0,0508 | 10,16 |
| 2 | ADO2 | 0,482 | 9,96 | 4,86 | 5,21 | 0,43 | 0,0267 | 5,54 |
| 2 | ADO3 | 0,674 | 12,23 | 8,03 | 7,03 | 2,10 | 0,0400 | 5,93 |
| 2 | ADO3 | 0,553 | 12,61 | 8,20 | 0,41 | 2,86 | 0,0428 | 7,74 |
| 2 | ADO3 | 0,807 | 13,81 | 8,91 | 7,36 | 2,14 | 0,0378 | 4,68 |
| 2 | ADO3 | 0,642 | 12,89 | 7,89 | 8,11 | 2,03 | 0,0455 | 7,09 |
| 2 | ADO3 | 0,493 | 12,94 | 6,83 | 7,12 | 2,98 | 0,0510 | 10,34 |
| 2 | ADO3 | 0,493 | 10,99 | 7,09 | 7,11 | 2,00 | 0,0415 | 8,42 |
| 2 | ADO3 | 0,772 | 13,22 | 10,23 | 6,35 | 2,23 | 0,0310 | 4,02 |
| 2 | ADO3 | 0,675 | 14,81 | 8,72 | 8,23 | 1,76 | 0,0337 | 4,99 |
| 2 | ADO3 | 0,684 | 11,61 | 8,26 | 6,19 | 2,03 | 0,0405 | 5,92 |
| 2 | ADO3 | 0,706 | 13,86 | 8,50 | 7,13 | 2,10 | 0,0492 | 6,97 |
| 2 | ADO3 | 0,595 | 14,16 | 8,80 | 7,11 | 2,26 | 0,0483 | 8,12 |
| 2 | ADO3 | 0,568 | 11,20 | 8,11 | 6,20 | 1,71 | 0,0277 | 4,88 |
| 2 | ADO3 | 0,840 | 12,80 | 9,64 | 6,37 | 2,70 | 0,0526 | 6,27 |
| 2 | ADO3 | 0,763 | 12,86 | 7,93 | 6,48 | 2,07 | 0,0400 | 5,24 |
| 2 | ADO3 | 0,533 | 12,02 | 7,00 | 6,96 | 2,40 | 0,0451 | 8,46 |
| 2 | ADO3 | 0,521 | 13,23 | 5,56 | 7,92 | 2,38 | 0,0490 | 9,41 |
| 2 | ADO3 | 0,799 | 13,65 | 8,82 | 6,98 | 2,82 | 0,0446 | 5,58 |
| 2 | ADO3 | 0,778 | 13,91 | 9,13 | 6,65 | 2,67 | 0,0468 | 6,01 |
| 2 | ADO3 | 0,667 | 12,92 | 7,86 | 6,20 | 2,08 | 0,0301 | 4,51 |
| 2 | ADO3 | 0,712 | 13,90 | 8,65 | 6,93 | 2,88 | 0,0508 | 7,13 |
| 2 | ADO3 | 0,730 | 13,78 | 9,54 | 5,97 | 2,43 | 0,0324 | 4,44 |
| 2 | ADO3 | 0,627 | 13,40 | 8,39 | 7,91 | 2,37 | 0,0367 | 5,86 |
| 2 | ADO3 | 0,710 | 13,32 | 7,96 | 6,02 | 2,53 | 0,0392 | 5,52 |
| 2 | ADO3 | 0,544 | 11,83 | 7,58 | 7,52 | 2,36 | 0,0458 | 8,42 |
| 2 | ADO3 | 0,587 | 12,87 | 9,21 | 8,29 | 1,61 | 0,0329 | 5,60 |
| 2 | PG | 0,540 | 10,39 | 6,72 | 7,30 | 2,45 | 0,0780 | 14,44 |
| 2 | PG | 0,480 | 10,98 | 7,42 | 6,43 | 1,41 | 0,0570 | 11,88 |
| 2 | PG | 0,380 | 9,46 | 4,85 | 5,81 | 1,64 | 0,0620 | 16,32 |
| 2 | PG | 0,560 | 10,43 | 7,39 | 6,28 | 1,86 | 0,0510 | 9,11 |
| 2 | PG | 0,360 | 8,08 | 4,62 | 6,76 | 1,78 | 0,0730 | 20,28 |
| 2 | PG | 0,610 | 10,24 | 7,88 | 7,26 | 2,69 | 0,0760 | 12,46 |
| 2 | PG | 0,280 | 7,14 | 4,47 | 5,22 | 3,04 | 0,0460 | 16,43 |
| 2 | PG | 0,620 | 11,26 | 6,12 | 6,69 | 3,03 | 0,0610 | 9,84 |
| 2 | PG | 0,290 | 7,74 | 3,80 | 5,17 | 1,64 | 0,0450 | 15,52 |
| 2 | PG | 0,650 | 7,10 | 7,39 | 6,51 | 2,23 | 0,0920 | 14,15 |
| 2 | PG | 0,560 | 11,08 | 7,46 | 6,33 | 2,31 | 0,0810 | 14,46 |
| 2 | PG | 0,450 | 10,55 | 6,04 | 6,42 | 1,68 | 0,0830 | 18,44 |
| 2 | PG | 0,520 | 8,23 | 6,81 | 5,31 | 0,70 | 0,0600 | 11,54 |
| 2 | PG | 0,380 | 6,90 | 5,05 | 6,68 | 1,34 | 0,0630 | 16,58 |
| 2 | PG | 0,540 | 10,47 | 7,18 | 7,01 | 1,67 | 0,0510 | 9,44 |
| 2 | PG | 0,270 | 10,37 | 5,51 | 8,29 | 2,15 | 0,0410 | 15,19 |
| 2 | PG | 0,360 | 7,83 | 4,10 | 6,78 | 3,06 | 0,0670 | 18,61 |
| 2 | PG | 0,550 | 10,34 | 6,54 | 6,36 | 2,01 | 0,0680 | 12,36 |
| 2 | PG | 0,440 | 9,65 | 6,48 | 4,83 | 0,77 | 0,0400 | 9,09 |
| 2 | PG | 0,510 | 9,50 | 7,97 | 7,28 | 1,52 | 0,0820 | 16,08 |
| 2 | PG | 0,460 | 10,21 | 7,48 | 5,32 | 0,48 | 0,0720 | 15,65 |
| 2 | PG | 0,570 | 10,32 | 6,63 | 6,02 | 2,01 | 0,0760 | 13,33 |
| 2 | PG | 0,430 | 9,63 | 9,82 | 6,27 | 1,66 | 0,0810 | 18,84 |
| 2 | PG | 0,540 | 9,91 | 6,07 | 5,07 | 0,70 | 0,0470 | 8,70 |
| 2 | PG | 0,550 | 9,61 | 6,21 | 5,53 | 0,55 | 0,0400 | 7,27 |

| 3 | ME1 | 0,424 | 10,00 | 5,51 | 4,67 | 1,15 | 0,0247 | 5,83 |
| --- | --- | --- | --- | --- | --- | --- | --- | --- |
| 3 | ME1 | 0,400 | 11,48 | 7,38 | 6,09 | 0,79 | 0,0325 | 8,13 |
| 3 | ME1 | 0,414 | 10,39 | 4,94 | 6,06 | 0,71 | 0,0255 | 6,16 |
| 3 | ME1 | 0,373 | 10,96 | 8,20 | 6,03 | 0,45 | 0,0377 | 10,11 |
| 3 | ME1 | 0,433 | 9,22 | 5,97 |  | 1,36 | 0,0316 | 7,30 |
| 3 | ME1 | 0,466 | 10,76 | 5,97 | 4,94 | 1,28 | 0,0315 | 6,76 |
| 3 | ME1 | 0,496 | 10,63 | 5,97 | 6,18 | 1,72 | 0,0399 | 8,04 |
| 3 | ME1 | 0,438 | 8,83 | 6,37 | 4,68 | 0,79 | 0,0239 | 5,46 |
| 3 | ME1 | 0,431 | 10,47 | 5,82 | 5,72 | 0,95 | 0,0353 | 8,19 |
| 3 | ME1 | 0,449 | 10,05 | 5,97 | 5,49 | 0,85 | 0,0411 | 9,15 |
| 3 | ME1 | 0,481 | 10,22 | 5,52 | 4,44 | 1,24 | 0,0456 | 9,48 |
| 3 | ME1 | 0,364 | 9,40 | 5,43 | 4,90 | 1,90 | 0,0296 | 8,13 |
| 3 | ME1 | 0,365 | 10,60 | 5,57 | 6,40 | 1,04 | 0,0355 | 9,73 |
| 3 | ME1 | 0,326 | 9,72 | 4,87 | 5,53 | 0,89 | 0,0428 | 13,13 |
| 3 | ME1 | 0,444 | 10,30 | 5,45 | 5,46 | 0,76 | 0,0372 | 8,38 |
| 3 | ME1 | 0,334 | 9,36 | 5,43 | 5,63 | 0,64 | 0,0340 | 10,18 |
| 3 | ME1 | 0,351 | 10,98 | 6,40 | 4,81 | 0,80 | 0,0253 | 7,21 |
| 3 | ME1 | 0,241 | 9,97 | 3,27 | 5,37 | 0,59 | 0,0339 | 14,07 |
| 3 | ME1 | 0,383 | 9,72 | 6,55 | 4,42 | 0,91 | 0,0326 | 8,51 |
| 3 | ME1 | 0,507 | 10,57 | 5,81 | 4,76 | 0,95 | 0,0406 | 8,01 |
| 3 | ME1 | 0,426 | 9,39 | 7,39 | 4,44 | 0,95 | 0,0343 | 8,05 |
| 3 | ME1 | 0,411 | 9,84 | 5,69 | 4,97 | 1,19 | 0,0419 | 10,19 |
| 3 | ME1 | 0,438 | 9,54 | 5,50 | 5,49 | 0,95 | 0,0404 | 9,22 |
| 3 | ME1 | 0,397 | 9,90 | 4,23 | 4,38 | 1,21 | 0,0397 | 10,00 |
| 3 | ME1 | 0,418 | 10,28 | 6,03 | 5,94 | 1,13 | 0,0392 | 9,38 |
| 3 | ME2 | 0,436 | 10,21 | 4,85 | 5,14 | 1,35 | 0,0229 | 5,25 |
| 3 | ME2 | 0,492 | 11,48 | 4,07 | 4,82 | 0,96 | 0,0342 | 6,95 |
| 3 | ME2 | 0,598 | 9,74 | 4,72 | 4,41 | 1,87 | 0,0234 | 3,91 |
| 3 | ME2 | 0,442 | 11,41 | 6,45 | 5,55 | 0,85 | 0,0284 | 6,42 |
| 3 | ME2 | 0,447 | 12,01 | 7,97 | 5,26 | 1,84 | 0,0318 | 7,12 |
| 3 | ME2 | 0,431 | 10,76 | 7,49 | 4,00 | 1,73 | 0,0234 | 5,42 |
| 3 | ME2 | 0,543 | 11,53 | 5,69 | 6,39 | 0,80 | 0,0291 | 5,36 |
| 3 | ME2 | 0,457 | 9,32 | 5,64 | 5,34 | 0,97 | 0,0278 | 6,09 |
| 3 | ME2 | 0,390 | 9,54 | 4,70 | 4,57 | 0,75 | 0,0220 | 5,64 |
| 3 | ME2 | 0,395 | 10,61 | 6,04 | 4,75 | 0,77 | 0,0258 | 6,53 |
| 3 | ME2 | 0,383 | 10,37 | 7,21 | 3,77 | 1,70 | 0,0241 | 6,29 |
| 3 | ME2 | 0,376 | 11,04 | 5,79 | 4,05 | 0,54 | 0,0251 | 6,68 |
| 3 | ME2 | 0,456 | 13,14 | 9,04 | 2,93 | 0,84 | 0,0305 | 6,69 |
| 3 | ME2 | 0,396 | 10,14 | 6,70 | 6,55 | 1,20 | 0,0330 | 8,33 |
| 3 | ME2 | 0,478 | 11,16 | 6,98 | 3,83 | 0,84 | 0,0303 | 6,35 |
| 3 | ME2 | 0,360 | 11,10 | 7,00 | 5,86 | 0,60 | 0,0258 | 7,18 |
| 3 | ME2 | 0,454 | 12,54 | 9,33 | 4,62 | 2,26 | 0,0367 | 8,09 |
| 3 | ME2 | 0,496 | 11,52 | 6,75 | 5,08 | 1,86 | 0,0415 | 8,38 |
| 3 | ME2 | 0,367 | 9,30 | 6,33 | 3,78 | 0,93 | 0,0350 | 9,53 |
| 3 | ME2 | 0,474 | 10,96 | 5,94 | 5,21 | 0,69 | 0,0312 | 6,58 |
| 3 | ME2 | 0,411 | 10,87 | 6,90 | 2,74 | 2,16 | 0,0318 | 7,74 |
| 3 | ME2 | 0,481 | 10,33 | 7,85 | 4,42 | 0,88 | 0,0351 | 7,30 |
| 3 | ME2 | 0,435 | 11,30 | 7,91 | 5,90 | 1,77 | 0,0351 | 8,07 |
| 3 | ME2 | 0,431 | 9,93 | 8,15 | 4,86 | 1,72 | 0,0402 | 9,34 |
| 3 | ME2 | 0,522 | 11,96 | 9,48 | 5,45 | 0,65 | 0,0285 | 5,46 |
| 3 | ME3 | 0,409 | 10,51 | 6,30 | 4,62 | 0,71 | 0,0290 | 7,10 |
| 3 | ME3 | 0,492 | 10,38 | 6,08 | 6,40 | 0,66 | 0,0312 | 6,34 |
| 3 | ME3 | 0,383 | 11,17 | 5,96 | 6,53 | 0,77 | 0,0316 | 8,25 |
| 3 | ME3 | 0,393 | 7,95 | 7,00 | 4,63 | 0,78 | 0,0843 | 21,47 |
| 3 | ME3 | 0,467 | 10,85 | 4,36 | 4,94 | 0,76 | 0,0341 | 7,30 |
| 3 | ME3 | 0,328 | 5,20 | 5,02 | 3,98 | 0,96 | 0,0237 | 7,22 |
| 3 | ME3 | 0,546 | 10,05 | 6,03 | 4,26 | 1,02 | 0,0456 | 8,36 |
| 3 | ME3 | 0,483 | 10,79 | 5,55 | 4,34 | 1,18 | 0,0459 | 9,51 |
| 3 | ME3 | 0,490 | 9,70 | 4,06 | 4,60 | 0,82 | 0,0485 | 9,90 |
| 3 | ME3 | 0,336 | 9,83 | 5,31 | 5,86 | 0,57 | 0,0318 | 9,46 |
| 3 | ME3 | 0,356 | 10,80 | 3,97 | 5,52 | 1,09 | 0,0420 | 11,80 |
| 3 | ME3 | 0,409 | 10,58 | 5,12 | 7,16 | 0,52 | 0,0279 | 6,82 |
| 3 | ME3 | 0,412 | 9,74 | 6,15 | 4,63 | 0,82 | 0,0258 | 6,27 |
| 3 | ME3 | 0,459 | 9,03 | 5,59 | 5,64 | 1,06 | 0,0381 | 8,30 |
| 3 | ME3 | 0,503 | 12,23 | 4,40 | 5,83 | 2,03 | 0,0615 | 12,24 |
| 3 | ME3 | 0,460 | 10,07 | 4,70 | 5,58 | 0,61 | 0,0373 | 8,10 |
| 3 | ME3 | 0,356 | 9,35 | 5,00 | 4,80 | 1,25 | 0,0453 | 12,74 |
| 3 | ME3 | 0,450 | 9,76 | 4,84 | 5,63 | 1,34 | 0,0371 | 8,24 |
| 3 | ME3 | 0,322 | 8,13 | 4,69 | 5,08 | 1,06 | 0,0427 | 13,27 |
| 3 | ME3 | 0,529 | 9,43 | 5,22 | 6,52 | 1,72 | 0,0457 | 8,64 |
| 3 | ME3 | 0,296 | 9,90 | 3,97 | 4,90 | 0,72 | 0,0260 | 8,77 |
| 3 | ME3 | 0,490 | 11,38 | 5,98 | 5,15 | 1,66 | 0,0555 | 11,32 |
| 3 | ME3 | 0,410 | 10,36 | 4,90 | 5,58 | 1,20 | 0,0331 | 8,08 |
| 3 | ME3 | 0,467 | 10,19 | 5,94 | 5,51 | 1,18 | 0,0348 | 7,46 |
| 3 | ME3 | 0,528 | 11,25 | 5,72 | 5,12 | 0,78 | 0,0363 | 6,88 |
| 3 | ME31 | 0,499 | 10,36 | 6,22 | 3,53 | 0,65 | 0,0204 | 4,09 |
| 3 | ME31 | 0,474 | 10,16 | 4,78 | 5,04 | 0,70 | 0,0357 | 7,53 |
| 3 | ME31 | 0,297 | 9,15 | 4,22 | 4,24 | 0,63 | 0,0282 | 9,49 |
| 3 | ME31 | 0,472 | 9,77 | 5,82 | 3,36 | 0,69 | 0,0213 | 4,51 |
| 3 | ME31 | 0,481 | 10,58 | 5,73 | 5,50 | 0,91 | 0,0254 | 5,29 |
| 3 | ME31 | 0,543 | 11,37 | 5,28 | 5,14 | 0,81 | 0,0385 | 7,10 |
| 3 | ME31 | 0,459 | 12,00 | 5,24 | 5,68 | 1,04 | 0,0353 | 7,69 |
| 3 | ME31 | 0,417 | 10,21 | 4,72 | 4,92 | 1,08 | 0,0279 | 6,69 |
| 3 | ME31 | 0,412 | 9,33 | 4,99 | 4,79 | 0,78 | 0,0265 | 6,43 |
| 3 | ME31 | 0,419 | 8,92 | 6,48 | 4,55 | 1,12 | 0,0248 | 5,91 |
| 3 | ME31 | 0,440 | 9,53 | 5,32 | 4,75 | 0,76 | 0,0360 | 8,19 |
| 3 | ME31 | 0,440 | 11,16 | 6,15 | 5,97 | 0,65 | 0,0345 | 7,84 |
| 3 | ME31 | 0,478 | 10,61 | 5,16 | 4,58 | 1,63 | 0,0354 | 7,40 |
| 3 | ME31 | 0,496 | 10,41 | 5,26 | 4,49 | 0,67 | 0,0391 | 7,88 |
| 3 | ME31 | 0,505 | 11,18 | 5,41 | 5,00 | 0,87 | 0,0276 | 5,47 |
| 3 | ME31 | 0,393 | 9,62 | 5,48 | 4,73 | 0,83 | 0,0280 | 7,12 |
| 3 | ME31 | 0,345 | 8,37 | 5,62 | 4,37 | 0,77 | 0,0305 | 8,84 |
| 3 | ME31 | 0,323 | 7,51 | 4,17 | 4,73 | 0,48 | 0,0219 | 6,79 |
| 3 | ME31 | 0,399 | 9,48 | 5,28 | 5,27 | 0,63 | 0,0303 | 7,59 |
| 3 | ME31 | 0,408 | 8,90 | 5,55 | 5,81 | 0,68 | 0,0451 | 11,05 |
| 3 | ME31 | 0,518 | 11,07 | 4,72 | 3,98 | 0,68 | 0,0293 | 5,66 |
| 3 | ME31 | 0,381 | 8,91 | 5,14 | 4,97 | 0,62 | 0,0384 | 10,07 |
| 3 | ME31 | 0,354 | 8,79 | 4,24 | 3,70 | 0,64 | 0,0247 | 6,99 |
| 3 | ME31 | 0,512 | 10,98 | 6,92 | 4,46 | 0,61 | 0,0269 | 5,26 |
| 3 | ME31 | 0,397 | 9,12 | 5,05 | 4,49 | 0,66 | 0,0243 | 6,11 |
| 3 | ME4 | 0,321 | 8,23 | 4,71 | 5,75 | 0,74 | 0,0325 | 10,12 |
| 3 | ME4 | 0,463 | 10,52 | 5,52 | 4,56 | 0,95 | 0,0491 | 10,60 |
| 3 | ME4 | 0,484 | 10,22 | 7,81 | 4,50 | 0,94 | 0,0510 | 10,54 |
| 3 | ME4 | 0,260 | 7,00 | 4,03 | 3,76 | 1,00 | 0,0465 | 17,88 |
| 3 | ME4 | 0,280 | 8,02 | 5,39 | 4,60 | 0,70 | 0,0508 | 18,14 |
| 3 | ME4 | 0,496 | 11,17 | 5,62 | 5,58 | 1,47 | 0,0662 | 13,35 |
| 3 | ME4 | 0,327 | 8,27 | 5,47 | 6,00 | 1,52 | 0,0663 | 20,28 |
| 3 | ME4 | 0,420 | 9,67 | 6,41 | 3,96 | 1,66 | 0,0330 | 7,86 |
| 3 | ME4 | 0,252 | 7,17 | 5,16 | 4,12 | 0,75 | 0,0417 | 16,55 |
| 3 | ME4 | 0,440 | 11,27 | 5,18 | 4,09 | 0,72 | 0,0359 | 8,16 |
| 3 | ME4 | 0,373 | 9,63 | 6,70 | 7,38 | 0,65 | 0,0478 | 12,82 |
| 3 | ME4 | 0,340 | 8,80 | 5,34 | 5,12 | 0,62 | 0,0370 | 10,88 |
| 3 | ME4 | 0,387 | 5,77 | 5,29 | 4,40 | 0,60 | 0,0379 | 9,79 |
| 3 | ME4 | 0,517 | 9,77 | 5,74 | 6,85 | 0,79 | 0,0850 | 16,44 |
| 3 | ME4 | 0,411 | 7,64 | 4,56 | 4,47 | 1,30 | 0,0467 | 11,36 |
| 3 | ME4 | 0,518 | 9,27 | 8,00 | 3,23 | 0,85 | 0,0370 | 7,14 |
| 3 | ME4 | 0,370 | 7,81 | 4,75 | 4,76 | 0,92 | 0,0565 | 15,27 |
| 3 | ME4 | 0,453 | 9,79 | 6,80 | 3,75 | 1,41 | 0,0393 | 8,68 |
| 3 | ME4 | 0,510 | 9,00 | 6,75 | 3,85 | 1,50 | 0,0330 | 6,47 |
| 3 | ME4 | 0,413 | 10,80 | 5,50 | 4,40 | 0,95 | 0,0583 | 14,12 |
| 3 | ME4 | 0,447 | 9,96 | 7,06 | 5,70 | 0,84 | 0,0484 | 10,83 |
| 3 | ME4 | 0,371 | 9,75 | 4,76 | 5,80 | 1,24 | 0,0431 | 11,62 |
| 3 | ME4 | 0,453 | 9,97 | 6,26 | 5,75 | 0,68 | 0,0437 | 9,65 |
| 3 | ME4 | 0,264 | 9,28 | 3,19 | 4,00 | 0,74 | 0,0375 | 14,20 |
| 3 | ME4 | 0,460 | 10,53 | 6,97 | 3,42 | 0,84 | 0,0344 | 7,48 |
| 3 | ME5 | 0,323 | 9,97 | 6,25 | 6,07 | 2,88 | 0,0302 | 9,35 |
| 3 | ME5 | 0,459 | 12,74 | 7,44 | 7,14 | 2,87 | 0,0393 | 8,55 |
| 3 | ME5 | 0,392 | 10,61 | 7,32 | 6,69 | 3,13 | 0,0310 | 7,90 |
| 3 | ME5 | 0,467 | 12,47 | 7,09 | 6,53 | 2,17 | 0,0331 | 7,09 |
| 3 | ME5 | 0,389 | 10,24 | 7,28 | 6,66 | 2,17 | 0,0415 | 10,68 |
| 3 | ME5 | 0,465 | 11,98 | 6,93 | 7,47 | 2,12 | 0,0413 | 8,88 |
| 3 | ME5 | 0,495 | 11,62 | 7,99 | 7,34 | 2,87 | 0,0542 | 10,96 |
| 3 | ME5 | 0,453 | 11,71 | 7,76 | 6,52 | 2,39 | 0,0196 | 4,33 |
| 3 | ME5 | 0,364 | 10,20 | 7,55 | 6,65 | 2,82 | 0,0386 | 10,60 |
| 3 | ME5 | 0,485 | 11,61 | 5,67 | 6,72 | 2,49 | 0,0383 | 7,89 |
| 3 | ME5 | 0,493 | 11,07 | 8,23 | 5,72 | 3,41 | 0,0364 | 7,38 |
| 3 | ME5 | 0,379 | 9,50 | 6,99 | 5,25 | 2,27 | 0,0307 | 8,10 |
| 3 | ME5 | 0,389 | 10,79 | 5,52 | 4,64 | 2,02 | 0,0237 | 6,09 |
| 3 | ME5 | 0,374 | 11,14 | 7,03 | 5,85 | 2,22 | 0,0450 | 12,02 |
| 3 | ME5 | 0,494 | 11,83 | 7,75 | 5,32 | 2,12 | 0,0418 | 8,46 |
| 3 | ME5 | 0,502 | 13,28 | 6,82 | 6,85 | 1,53 | 0,0403 | 8,02 |
| 3 | ME5 | 0,442 | 12,22 | 6,24 | 7,09 | 1,95 | 0,0390 | 8,83 |
| 3 | ME5 | 0,461 | 11,42 | 6,87 | 6,64 | 1,53 | 0,0452 | 9,80 |
| 3 | ME5 | 0,463 | 11,50 | 6,32 | 6,05 | 1,57 | 0,0417 | 9,01 |
| 3 | ME5 | 0,421 | 11,03 | 6,31 | 6,64 | 1,98 | 0,0362 | 8,59 |
| 3 | ME5 | 0,378 | 9,52 | 6,47 | 5,91 | 2,29 | 0,0409 | 10,83 |
| 3 | ME5 | 0,392 | 11,39 | 5,43 | 6,75 | 1,99 | 0,0378 | 9,64 |
| 3 | ME5 | 0,463 | 11,73 | 6,80 | 5,21 | 0,81 | 0,0231 | 4,99 |
| 3 | ME5 | 0,492 | 10,88 | 6,92 | 5,47 | 1,16 | 0,0260 | 5,29 |
| 3 | ME5 | 0,419 | 11,85 | 6,84 | 6,62 | 2,43 | 0,0368 | 8,78 |
| 3 | ME6 | 0,392 | 10,53 | 6,20 | 5,23 | 2,69 | 0,0301 | 7,68 |
| 3 | ME6 | 0,551 | 11,75 | 7,27 | 5,94 | 2,63 | 0,0583 | 10,58 |
| 3 | ME6 | 0,481 | 11,47 | 5,91 | 6,43 | 2,24 | 0,0522 | 10,85 |
| 3 | ME6 | 0,487 | 10,98 | 6,46 | 5,15 | 3,55 | 0,0400 | 8,21 |
| 3 | ME6 | 0,395 | 9,07 | 6,14 | 5,95 | 1,85 | 0,0555 | 14,05 |
| 3 | ME6 | 0,365 | 9,80 | 5,60 | 4,27 | 3,99 | 0,0575 | 15,75 |
| 3 | ME6 | 0,365 | 10,81 | 4,83 | 4,90 | 1,15 | 0,0239 | 6,55 |
| 3 | ME6 | 0,500 | 11,02 | 5,07 | 6,50 | 1,52 | 0,0505 | 10,10 |
| 3 | ME6 | 0,553 | 12,54 | 8,22 | 6,09 | 3,19 | 0,0588 | 10,63 |
| 3 | ME6 | 0,403 | 11,48 | 5,44 | 6,30 | 1,93 | 0,0477 | 11,84 |
| 3 | ME6 | 0,473 | 11,13 | 5,64 | 6,46 | 1,92 | 0,0709 | 14,99 |
| 3 | ME6 | 0,477 | 10,37 | 6,75 | 6,45 | 1,90 | 0,0587 | 12,31 |
| 3 | ME6 | 0,385 | 9,66 | 6,05 | 5,13 | 1,95 | 0,0470 | 12,21 |
| 3 | ME6 | 0,427 | 11,90 | 5,32 | 6,26 | 0,79 | 0,0459 | 10,75 |
| 3 | ME6 | 0,339 | 10,41 | 4,18 | 7,45 | 1,69 | 0,0459 | 13,54 |
| 3 | ME6 | 0,435 | 11,62 | 6,63 | 6,69 | 1,63 | 0,0660 | 15,17 |
| 3 | ME6 | 0,393 | 8,82 | 6,73 | 4,95 | 2,01 | 0,0439 | 11,17 |
| 3 | ME6 | 0,557 | 11,88 | 6,45 | 5,82 | 1,82 | 0,0446 | 8,01 |
| 3 | ME6 | 0,423 | 10,77 | 7,99 | 4,61 | 1,16 | 0,0481 | 11,37 |
| 3 | ME6 | 0,406 | 10,71 | 7,95 | 6,58 | 2,00 | 0,0407 | 10,02 |
| 3 | ME6 | 0,370 | 7,55 | 9,07 | 5,32 | 2,16 | 0,0310 | 8,38 |
| 3 | ME6 | 0,446 | 10,90 | 8,13 | 5,40 | 1,20 | 0,0520 | 11,66 |
| 3 | ME6 | 0,304 | 7,90 | 5,20 | 4,74 | 1,96 | 0,0450 | 14,80 |
| 3 | ME6 | 0,445 | 11,14 | 6,57 | 5,22 | 1,06 | 0,0440 | 9,89 |
| 3 | ME6 | 0,482 | 10,16 | 6,57 | 6,32 | 1,77 | 0,0450 | 9,34 |
| 3 | ME7 | 0,452 | 11,03 | 6,49 | 7,02 | 2,36 | 0,0377 | 8,34 |
| 3 | ME7 | 0,532 | 10,13 | 7,14 | 5,40 | 2,07 | 0,0399 | 7,50 |
| 3 | ME7 | 0,386 | 8,68 | 7,02 | 6,58 | 2,08 | 0,0392 | 10,16 |
| 3 | ME7 | 0,448 | 10,76 | 7,41 | 6,58 | 2,03 | 0,0310 | 6,92 |
| 3 | ME7 | 0,438 | 8,40 | 6,04 | 8,14 | 2,50 | 0,0536 | 12,24 |
| 3 | ME7 | 0,414 | 9,43 | 6,76 | 6,24 | 2,79 | 0,0266 | 6,43 |
| 3 | ME7 | 0,425 | 11,41 | 6,26 | 6,95 | 2,39 | 0,0191 | 4,49 |
| 3 | ME7 | 0,448 | 10,67 | 6,77 | 6,92 | 2,66 | 0,0237 | 5,29 |
| 3 | ME7 | 0,515 | 11,63 | 8,42 | 7,90 | 2,59 | 0,0268 | 5,20 |
| 3 | ME7 | 0,422 | 10,64 | 6,55 | 6,22 | 1,85 | 0,0250 | 5,92 |
| 3 | ME7 | 0,420 | 10,20 | 8,26 | 6,28 | 1,34 | 0,0520 | 12,38 |
| 3 | ME7 | 0,474 | 11,36 | 7,37 | 6,69 | 2,80 | 0,0371 | 7,83 |
| 3 | ME7 | 0,383 | 9,89 | 7,60 | 6,26 | 1,83 | 0,0387 | 10,10 |
| 3 | ME7 | 0,319 | 9,27 | 5,13 | 7,41 | 1,93 | 0,0335 | 10,50 |
| 3 | ME7 | 0,489 | 11,58 | 6,08 | 4,89 | 2,68 | 0,0410 | 8,38 |
| 3 | ME7 | 0,410 | 8,87 | 6,43 | 5,42 | 2,14 | 0,0275 | 6,71 |
| 3 | ME7 | 0,461 | 11,01 | 6,77 | 7,52 | 2,54 | 0,0584 | 12,67 |
| 3 | ME7 | 0,463 | 11,05 | 7,78 | 6,72 | 1,60 | 0,0336 | 7,26 |
| 3 | ME7 | 0,476 | 11,19 | 6,46 | 6,36 | 2,90 | 0,0421 | 8,84 |
| 3 | ME7 | 0,405 | 11,75 | 6,58 | 6,58 | 1,65 | 0,0271 | 6,69 |
| 3 | ME7 | 0,406 | 11,56 | 6,81 | 6,62 | 1,72 | 0,0299 | 7,36 |
| 3 | ME7 | 0,328 | 10,27 | 5,11 | 3,41 | 1,65 | 0,0142 | 4,33 |
| 3 | ME7 | 0,435 | 10,11 | 6,77 | 5,53 | 2,44 | 0,0304 | 6,99 |
| 3 | ME7 | 0,360 | 10,27 | 6,72 | 4,27 | 1,73 | 0,0135 | 3,75 |
| 3 | ME7 | 0,397 | 9,39 | 7,05 | 5,95 | 2,70 | 0,0322 | 8,11 |
| 3 | ME8 | 0,334 | 11,49 | 5,10 | 6,00 | 3,32 | 0,0314 | 9,42 |
| 3 | ME8 | 0,434 | 12,87 | 6,88 | 6,94 | 2,15 | 0,0343 | 7,91 |
| 3 | ME8 | 0,423 | 10,74 | 7,55 | 6,31 | 2,28 | 0,0213 | 5,04 |
| 3 | ME8 | 0,400 | 9,66 | 7,04 | 5,34 | 2,73 | 0,0253 | 6,32 |
| 3 | ME8 | 0,464 | 12,33 | 7,92 | 6,67 | 2,44 | 0,0306 | 6,59 |
| 3 | ME8 | 0,444 | 10,77 | 7,59 | 6,72 | 2,21 | 0,0271 | 6,11 |
| 3 | ME8 | 0,438 | 11,10 | 7,97 | 6,74 | 2,43 | 0,0243 | 5,55 |
| 3 | ME8 | 0,230 | 9,17 | 5,27 | 4,57 | 2,28 | 0,0360 | 15,67 |
| 3 | ME8 | 0,367 | 9,14 | 7,94 | 7,42 | 3,80 | 0,0244 | 6,64 |
| 3 | ME8 | 0,503 | 12,21 | 8,62 | 7,94 | 2,60 | 0,0298 | 5,93 |
| 3 | ME8 | 0,260 | 12,28 | 4,35 | 6,81 | 2,96 | 0,0315 | 12,13 |
| 3 | ME8 | 0,500 | 11,89 | 9,23 | 7,21 | 2,38 | 0,0285 | 5,70 |
| 3 | ME8 | 0,523 | 12,32 | 8,00 | 5,97 | 2,08 | 0,0185 | 3,54 |
| 3 | ME8 | 0,365 | 9,77 | 7,46 | 5,68 | 2,60 | 0,0389 | 10,65 |
| 3 | ME8 | 0,474 | 10,52 | 7,35 | 8,27 | 2,50 | 0,0251 | 5,29 |
| 3 | ME8 | 0,564 | 12,47 | 6,52 | 6,34 | 1,69 | 0,0229 | 4,06 |
| 3 | ME8 | 0,314 | 10,70 | 4,02 | 6,66 | 3,28 | 0,0374 | 11,93 |
| 3 | ME8 | 0,477 | 12,17 | 7,17 | 7,51 | 2,40 | 0,0290 | 6,08 |
| 3 | ME8 | 0,470 | 11,16 | 6,27 | 7,16 | 2,18 | 0,0211 | 4,49 |
| 3 | ME8 | 0,531 | 11,48 | 7,62 | 7,49 | 2,18 | 0,0371 | 6,99 |
| 3 | ME8 | 0,503 | 12,67 | 6,70 | 6,22 | 1,83 | 0,0129 | 2,56 |
| 3 | ME8 | 0,264 | 10,75 | 6,27 | 6,29 | 2,14 | 0,0204 | 7,74 |
| 3 | ME8 | 0,366 | 9,22 | 7,15 | 6,00 | 2,34 | 0,0273 | 7,45 |
| 3 | ME8 | 0,507 | 11,87 | 8,55 | 7,18 | 2,08 | 0,0221 | 4,36 |
| 3 | ME8 | 0,245 | 9,48 | 3,95 | 6,96 | 1,95 | 0,0263 | 10,74 |
| 3 | ME9 | 0,460 | 12,39 | 4,87 | 7,80 | 2,48 | 0,0382 | 8,31 |
| 3 | ME9 | 0,383 | 11,77 | 5,89 | 7,87 | 1,59 | 0,0389 | 10,15 |
| 3 | ME9 | 0,410 | 11,88 | 7,01 | 7,38 | 2,48 | 0,0495 | 12,07 |
| 3 | ME9 | 0,441 | 11,09 | 5,58 | 6,19 | 2,37 | 0,0248 | 5,63 |
| 3 | ME9 | 0,435 | 11,44 | 6,76 | 6,42 | 2,00 | 0,0425 | 9,77 |
| 3 | ME9 | 0,389 | 9,43 | 4,46 | 7,05 | 2,39 | 0,0259 | 6,65 |
| 3 | ME9 | 0,437 | 12,17 | 6,58 | 6,72 | 2,43 | 0,0278 | 6,36 |
| 3 | ME9 | 0,434 | 11,71 | 6,83 | 5,67 | 1,48 | 0,0319 | 7,36 |
| 3 | ME9 | 0,398 | 10,98 | 6,88 | 6,26 | 1,96 | 0,0315 | 7,92 |
| 3 | ME9 | 0,314 | 10,89 | 5,32 | 7,94 | 1,80 | 0,0400 | 12,73 |
| 3 | ME9 | 0,439 | 10,71 | 6,94 | 7,03 | 1,65 | 0,0253 | 5,76 |
| 3 | ME9 | 0,341 | 12,00 | 6,31 | 6,83 | 1,36 | 0,0214 | 6,27 |
| 3 | ME9 | 0,372 | 11,85 | 6,82 | 7,53 | 1,92 | 0,0343 | 9,23 |
| 3 | ME9 | 0,382 | 12,18 | 7,78 | 6,35 | 2,09 | 0,0244 | 6,39 |
| 3 | ME9 | 0,432 | 11,90 | 7,23 | 6,88 | 1,95 | 0,0398 | 9,22 |
| 3 | ME9 | 0,445 | 11,22 | 6,45 | 6,53 | 2,79 | 0,0550 | 12,35 |
| 3 | ME9 | 0,452 | 12,12 | 8,24 | 6,24 | 2,11 | 0,0378 | 8,37 |
| 3 | ME9 | 0,328 | 12,01 | 6,32 | 6,67 | 2,18 | 0,0291 | 8,88 |
| 3 | ME9 | 0,200 | 10,14 | 6,84 | 6,29 | 1,13 | 0,0260 | 12,97 |
| 3 | ME9 | 0,321 | 10,62 | 5,47 | 6,57 | 1,52 | 0,0329 | 10,27 |
| 3 | ME9 | 0,423 | 11,07 | 7,70 | 7,02 | 2,47 | 0,0330 | 7,80 |
| 3 | ME9 | 0,309 | 12,38 | 5,75 | 7,36 | 1,84 | 0,0333 | 10,79 |
| 3 | ME9 | 0,272 | 11,06 | 6,60 | 7,15 | 1,73 | 0,0445 | 16,35 |
| 3 | ME9 | 0,470 | 11,66 | 7,04 | 7,40 | 2,28 | 0,0441 | 9,39 |
| 3 | ME9 | 0,340 | 11,06 | 5,46 | 5,43 | 1,54 | 0,0198 | 5,82 |
| 3 | ME10 | 0,486 | 11,24 | 5,67 | 7,53 | 2,37 | 0,0465 | 9,57 |
| 3 | ME10 | 0,479 | 10,48 | 8,14 | 6,88 | 2,34 | 0,0544 | 11,36 |
| 3 | ME10 | 0,441 | 8,48 | 7,58 | 6,86 | 2,87 | 0,0458 | 10,39 |
| 3 | ME10 | 0,462 | 11,40 | 8,47 | 5,47 | 2,24 | 0,0483 | 10,45 |
| 3 | ME10 | 0,250 | 8,50 | 5,48 | 6,00 | 2,53 | 0,0366 | 14,64 |
| 3 | ME10 | 0,480 | 9,36 | 7,57 | 7,06 | 2,03 | 0,0531 | 11,06 |
| 3 | ME10 | 0,554 | 12,28 | 8,97 | 7,23 | 2,41 | 0,0489 | 8,83 |
| 3 | ME10 | 0,498 | 10,62 | 7,46 | 6,50 | 2,14 | 0,0395 | 7,93 |
| 3 | ME10 | 0,440 | 10,14 | 6,57 | 6,20 | 2,00 | 0,0411 | 9,34 |
| 3 | ME10 | 0,442 | 10,76 | 7,19 | 6,52 | 1,50 | 0,0354 | 8,01 |
| 3 | ME10 | 0,490 | 11,91 | 8,55 | 6,30 | 2,23 | 0,0324 | 6,61 |
| 3 | ME10 | 0,386 | 10,10 | 7,77 | 5,42 | 2,23 | 0,0411 | 10,65 |
| 3 | ME10 | 0,430 | 9,10 | 7,44 | 6,14 | 2,60 | 0,0399 | 9,28 |
| 3 | ME10 | 0,521 | 10,14 | 8,03 | 6,88 | 2,39 | 0,0547 | 10,50 |
| 3 | ME10 | 0,531 | 12,88 | 8,52 | 7,87 | 2,13 | 0,0581 | 10,94 |
| 3 | ME10 | 0,539 | 11,69 | 7,55 | 6,49 | 3,00 | 0,0380 | 7,05 |
| 3 | ME10 | 0,395 | 13,00 | 5,85 | 2,75 | 1,57 | 0,0329 | 8,33 |
| 3 | ME10 | 0,495 | 12,58 | 7,44 | 6,23 | 1,79 | 0,0569 | 11,49 |
| 3 | ME10 | 0,442 | 11,00 | 6,34 | 5,14 | 2,40 | 0,0408 | 9,23 |
| 3 | ME10 | 0,425 | 10,41 | 7,07 | 5,69 | 1,94 | 0,0404 | 9,51 |
| 3 | ME10 | 0,265 | 9,40 | 7,03 | 5,72 | 1,37 | 0,0183 | 6,91 |
| 3 | ME10 | 0,496 | 11,87 | 7,60 | 6,88 | 2,91 | 0,0513 | 10,34 |
| 3 | ME10 | 0,385 | 9,72 | 8,42 | 7,10 | 1,51 | 0,0387 | 10,05 |
| 3 | ME10 | 0,375 | 10,76 | 7,30 | 5,78 | 1,58 | 0,0235 | 6,27 |
| 3 | ME10 | 0,433 | 10,28 | 7,55 | 5,90 | 3,33 | 0,0355 | 8,20 |
| 3 | ME11 | 0,399 | 8,20 | 6,86 | 5,84 | 3,45 | 0,0490 | 12,28 |
| 3 | ME11 | 0,445 | 9,46 | 6,16 | 6,36 | 3,47 | 0,0660 | 14,83 |
| 3 | ME11 | 0,362 | 8,22 | 6,24 | 6,98 | 2,94 | 0,0480 | 13,26 |
| 3 | ME11 | 0,359 | 8,87 | 6,02 | 6,26 | 3,12 | 0,0350 | 9,75 |
| 3 | ME11 | 0,481 | 9,97 | 5,36 | 7,07 | 2,67 | 0,0430 | 8,94 |
| 3 | ME11 | 0,378 | 9,18 | 5,70 | 5,64 | 3,01 | 0,0570 | 15,08 |
| 3 | ME11 | 0,542 | 8,00 | 6,35 | 6,83 | 2,44 | 0,0310 | 5,72 |
| 3 | ME11 | 0,438 | 8,74 | 4,93 | 6,32 | 3,19 | 0,0420 | 9,59 |
| 3 | ME11 | 0,412 | 9,92 | 5,69 | 6,65 | 2,47 | 0,0350 | 8,50 |
| 3 | ME11 | 0,392 | 9,93 | 5,09 | 6,30 | 4,36 | 0,0780 | 19,90 |
| 3 | ME11 | 0,248 | 8,09 | 5,03 | 7,17 | 3,39 | 0,0390 | 15,73 |
| 3 | ME11 | 0,362 | 7,91 | 5,20 | 5,09 | 1,94 | 0,0240 | 6,63 |
| 3 | ME11 | 0,477 | 9,86 | 5,81 | 7,27 | 3,04 | 0,0330 | 6,92 |
| 3 | ME11 | 0,333 | 6,61 | 4,85 | 5,09 | 2,92 | 0,0370 | 11,11 |
| 3 | ME11 | 0,273 | 7,30 | 3,84 | 6,42 | 2,09 | 0,0270 | 9,89 |
| 3 | ME11 | 0,333 | 6,14 | 5,78 | 5,27 | 2,58 | 0,0260 | 7,81 |
| 3 | ME11 | 0,403 | 8,61 | 5,09 | 6,64 | 2,30 | 0,0410 | 10,17 |
| 3 | ME11 | 0,320 | 6,39 | 3,71 | 5,95 | 2,64 | 0,0420 | 13,13 |
| 3 | ME11 | 0,418 | 8,09 | 4,59 | 6,95 | 2,92 | 0,0610 | 14,59 |
| 3 | ME11 | 0,507 | 9,29 | 7,92 | 6,84 | 3,70 | 0,0530 | 10,45 |
| 3 | ME11 | 0,490 | 9,92 | 6,20 | 7,14 | 3,77 | 0,0510 | 10,41 |
| 3 | ME11 | 0,476 | 9,20 | 5,46 | 6,88 | 2,46 | 0,0430 | 9,03 |
| 3 | ME11 | 0,422 | 7,93 | 5,16 | 6,71 | 3,21 | 0,0480 | 11,37 |
| 3 | ME11 | 0,389 | 7,86 | 6,41 | 5,66 | 3,23 | 0,0290 | 7,46 |
| 3 | ME11 | 0,376 | 8,39 | 5,94 | 6,87 | 2,92 | 0,0560 | 14,89 |
| 3 | ME12 | 0,437 | 8,90 | 6,58 | 5,47 | 0,32 | 0,0426 | 9,75 |
| 3 | ME12 | 0,361 | 8,45 | 6,63 | 5,10 | 1,12 | 0,0466 | 12,91 |
| 3 | ME12 | 0,365 | 9,67 | 6,00 | 5,29 | 0,74 | 0,0331 | 9,07 |
| 3 | ME12 | 0,371 | 8,75 | 5,87 | 6,98 | 0,68 | 0,0398 | 10,73 |
| 3 | ME12 | 0,305 | 8,82 | 4,92 | 3,89 | 0,95 | 0,0248 | 8,13 |
| 3 | ME12 | 0,350 | 5,28 | 4,77 | 3,96 | 0,85 | 0,0335 | 9,57 |
| 3 | ME12 | 0,363 | 5,65 | 5,82 | 3,42 | 1,05 | 0,0221 | 6,09 |
| 3 | ME12 | 0,348 | 6,66 | 4,41 | 3,80 | 0,60 | 0,0230 | 6,61 |
| 3 | ME12 | 0,418 | 9,52 | 5,73 | 5,04 | 0,85 | 0,0278 | 6,65 |
| 3 | ME12 | 0,461 | 8,43 | 6,58 | 4,05 | 0,85 | 0,0444 | 9,63 |
| 3 | ME12 | 0,396 | 9,43 | 6,08 | 3,34 | 0,67 | 0,0235 | 5,93 |
| 3 | ME12 | 0,509 | 11,69 | 6,27 | 5,45 | 0,80 | 0,0400 | 7,86 |
| 3 | ME12 | 0,276 | 10,12 | 9,89 | 6,51 | 0,68 | 0,0520 | 18,84 |
| 3 | ME12 | 0,471 | 11,62 | 6,31 | 6,05 | 0,95 | 0,0524 | 11,13 |
| 3 | ME12 | 0,383 | 9,52 | 5,00 | 6,02 | 0,91 | 0,0418 | 10,91 |
| 3 | ME12 | 0,373 | 8,78 | 4,72 | 5,84 | 1,43 | 0,0350 | 9,38 |
| 3 | ME12 | 0,508 | 11,05 | 7,13 | 6,41 | 2,31 | 0,0463 | 9,11 |
| 3 | ME12 | 0,410 | 12,18 | 7,33 | 6,71 | 0,82 | 0,0451 | 11,00 |
| 3 | ME12 | 0,389 | 9,52 | 6,41 | 7,07 | 1,23 | 0,0442 | 11,36 |
| 3 | ME12 | 0,259 | 8,00 | 5,63 | 8,18 | 2,09 | 0,0353 | 13,63 |
| 3 | ME12 | 0,314 | 9,77 | 6,89 | 5,11 | 2,00 | 0,0457 | 14,55 |
| 3 | ME12 | 0,308 | 10,00 | 5,28 | 6,41 | 1,40 | 0,0442 | 14,35 |
| 3 | ME12 | 0,199 | 8,42 | 2,40 | 5,48 | 0,93 | 0,0392 | 19,70 |
| 3 | ME12 | 0,352 | 9,48 | 5,95 | 5,68 | 0,58 | 0,0385 | 10,94 |
| 3 | ME12 | 0,472 | 10,34 | 4,31 | 5,71 | 0,74 | 0,0240 | 5,08 |
| 3 | ME13 | 0,322 | 11,91 | 7,32 | 5,76 | 1,17 | 0,0294 | 9,12 |
| 3 | ME13 | 0,397 | 11,62 | 6,02 | 6,55 | 1,33 | 0,0355 | 8,95 |
| 3 | ME13 | 0,398 | 12,52 | 6,03 | 6,32 | 1,22 | 0,0284 | 7,14 |
| 3 | ME13 | 0,323 | 12,17 | 5,33 | 7,11 | 1,07 | 0,0335 | 10,37 |
| 3 | ME13 | 0,470 | 12,13 | 7,18 | 6,30 | 2,03 | 0,0311 | 6,62 |
| 3 | ME13 | 0,413 | 9,52 | 6,74 | 7,13 | 1,50 | 0,0362 | 8,76 |
| 3 | ME13 | 0,183 | 10,73 | 4,06 | 5,60 | 1,46 | 0,0220 | 12,00 |
| 3 | ME13 | 0,381 | 12,27 | 4,48 | 6,37 | 2,33 | 0,0362 | 9,51 |
| 3 | ME13 | 0,204 | 8,96 | 2,77 | 6,46 | 1,42 | 0,0318 | 15,57 |
| 3 | ME13 | 0,336 | 11,09 | 6,44 | 5,80 | 1,83 | 0,0298 | 8,88 |
| 3 | ME13 | 0,344 | 11,60 | 4,99 | 5,57 | 1,74 | 0,0248 | 7,20 |
| 3 | ME13 | 0,454 | 11,20 | 7,03 | 5,49 | 1,96 | 0,0322 | 7,10 |
| 3 | ME13 | 0,314 | 11,22 | 4,85 | 6,67 | 1,75 | 0,0342 | 10,88 |
| 3 | ME13 | 0,441 | 11,41 | 8,00 | 7,21 | 2,13 | 0,0255 | 5,78 |
| 3 | ME13 | 0,453 | 11,73 | 6,76 | 7,15 | 2,02 | 0,0296 | 6,54 |
| 3 | ME13 | 0,364 | 10,82 | 6,01 | 5,94 | 0,80 | 0,0231 | 6,34 |
| 3 | ME13 | 0,332 | 10,08 | 6,31 | 6,36 | 1,50 | 0,0203 | 6,12 |
| 3 | ME13 | 0,390 | 10,20 | 7,25 | 5,55 | 1,86 | 0,0188 | 4,82 |
| 3 | ME13 | 0,455 | 12,09 | 7,18 | 7,21 | 1,73 | 0,0374 | 8,22 |
| 3 | ME13 | 0,413 | 9,71 | 6,64 | 7,48 | 2,07 | 0,0275 | 6,66 |
| 3 | ME13 | 0,277 | 9,80 | 5,32 | 5,46 | 1,67 | 0,0202 | 7,30 |
| 3 | ME13 | 0,418 | 12,50 | 6,68 | 7,53 | 1,65 | 0,0331 | 7,91 |
| 3 | ME13 | 0,273 | 9,94 | 4,37 | 6,01 | 1,27 | 0,0242 | 8,85 |
| 3 | ME13 | 0,333 | 11,07 | 5,76 | 6,75 | 1,36 | 0,0319 | 9,59 |
| 3 | ME13 | 0,426 | 10,92 | 7,18 | 6,21 | 1,98 | 0,0226 | 5,31 |
| 3 | ME14 | 0,427 | 11,61 | 7,92 | 5,84 | 1,29 | 0,0212 | 4,97 |
| 3 | ME14 | 0,320 | 9,15 | 6,64 | 5,05 | 1,87 | 0,0123 | 3,86 |
| 3 | ME14 | 0,501 | 13,02 | 8,55 | 5,79 | 2,68 | 0,0414 | 8,27 |
| 3 | ME14 | 0,491 | 13,32 | 6,64 | 5,37 | 1,01 | 0,0431 | 8,77 |
| 3 | ME14 | 0,348 | 11,11 | 7,08 | 5,42 | 2,00 | 0,0124 | 3,55 |
| 3 | ME14 | 0,559 | 13,76 | 8,28 | 7,00 | 2,06 | 0,0461 | 8,25 |
| 3 | ME14 | 0,270 | 12,73 | 7,13 | 5,61 | 3,44 | 0,0319 | 11,81 |
| 3 | ME14 | 0,446 | 11,37 | 7,50 | 4,74 | 1,16 | 0,0202 | 4,53 |
| 3 | ME14 | 0,465 | 13,13 | 8,82 | 5,27 | 0,56 | 0,0350 | 7,52 |
| 3 | ME14 | 0,494 | 13,45 | 9,07 | 3,49 | 1,61 | 0,0361 | 7,31 |
| 3 | ME14 | 0,429 | 12,61 | 6,90 | 6,36 | 1,19 | 0,0484 | 11,27 |
| 3 | ME14 | 0,477 | 12,03 | 8,09 | 5,57 | 0,70 | 0,0348 | 7,29 |
| 3 | ME14 | 0,324 | 9,47 | 7,16 | 4,88 | 0,75 | 0,0396 | 12,21 |
| 3 | ME14 | 0,448 | 12,16 | 6,20 | 4,73 | 0,76 | 0,0255 | 5,69 |
| 3 | ME14 | 0,375 | 10,60 | 6,22 | 4,08 | 0,68 | 0,0222 | 5,92 |
| 3 | ME14 | 0,353 | 10,54 | 5,53 | 5,58 | 0,54 | 0,0245 | 6,95 |
| 3 | ME14 | 0,487 | 11,76 | 6,02 | 5,52 | 0,68 | 0,0284 | 5,83 |
| 3 | ME14 | 0,381 | 11,15 | 7,47 | 3,90 | 1,18 | 0,0250 | 6,57 |
| 3 | ME14 | 0,479 | 11,64 | 8,33 | 4,42 | 0,72 | 0,0318 | 6,63 |
| 3 | ME14 | 0,482 | 12,57 | 7,73 | 4,67 | 0,87 | 0,0229 | 4,75 |
| 3 | ME14 | 0,442 | 8,78 | 6,50 | 4,92 | 0,69 | 0,0334 | 7,56 |
| 3 | ME14 | 0,529 | 9,92 | 6,53 | 4,12 | 0,73 | 0,0225 | 4,25 |
| 3 | ME14 | 0,378 | 10,72 | 6,73 | 5,74 | 0,72 | 0,0366 | 9,68 |
| 3 | ME14 | 0,385 | 9,80 | 6,24 | 4,87 | 0,53 | 0,0268 | 6,95 |
| 3 | ME14 | 0,439 | 9,32 | 7,60 | 4,98 | 0,72 | 0,0214 | 4,88 |
| 3 | ME16 | 0,457 | 11,60 | 8,16 | 5,72 | 2,12 | 0,0369 | 8,07 |
| 3 | ME16 | 0,452 | 11,60 | 8,18 | 5,14 | 1,35 | 0,0328 | 7,26 |
| 3 | ME16 | 0,546 | 11,37 | 9,34 | 5,21 | 3,39 | 0,0409 | 7,49 |
| 3 | ME16 | 0,402 | 10,16 | 6,89 | 6,62 | 2,00 | 0,0384 | 9,55 |
| 3 | ME16 | 0,479 | 11,97 | 9,00 | 5,53 | 1,01 | 0,0306 | 6,39 |
| 3 | ME16 | 0,474 | 11,56 | 6,82 | 5,60 | 1,44 | 0,0516 | 10,89 |
| 3 | ME16 | 0,404 | 10,88 | 7,08 | 4,02 | 2,59 | 0,0319 | 7,90 |
| 3 | ME16 | 0,395 | 11,66 | 7,62 | 6,34 | 2,53 | 0,0506 | 12,81 |
| 3 | ME16 | 0,482 | 11,51 | 7,68 | 5,01 | 1,78 | 0,0424 | 8,80 |
| 3 | ME16 | 0,503 | 10,84 | 7,96 | 4,61 | 1,22 | 0,0393 | 7,81 |
| 3 | ME16 | 0,602 | 12,81 | 9,92 | 5,01 | 1,56 | 0,0573 | 9,52 |
| 3 | ME16 | 0,563 | 11,29 | 8,92 | 5,52 | 1,25 | 0,0467 | 8,29 |
| 3 | ME16 | 0,508 | 12,28 | 7,32 | 4,39 | 0,88 | 0,0251 | 4,94 |
| 3 | ME16 | 0,541 | 10,02 | 9,28 | 4,71 | 1,18 | 0,0344 | 6,36 |
| 3 | ME16 | 0,531 | 10,87 | 8,04 | 6,39 | 1,00 | 0,0384 | 7,23 |
| 3 | ME16 | 0,526 | 9,62 | 8,75 | 4,09 | 1,44 | 0,0256 | 4,87 |
| 3 | ME16 | 0,382 | 8,42 | 6,60 | 5,18 | 0,95 | 0,0296 | 7,75 |
| 3 | ME16 | 0,340 | 9,35 | 6,61 | 4,94 | 1,60 | 0,0311 | 9,15 |
| 3 | ME16 | 0,378 | 9,27 | 6,66 | 4,65 | 0,66 | 0,0295 | 7,80 |
| 3 | ME16 | 0,440 | 11,02 | 8,01 | 5,14 | 1,40 | 0,0155 | 3,52 |
| 3 | ME16 | 0,468 | 9,79 | 7,21 | 5,48 | 1,06 | 0,0462 | 9,87 |
| 3 | ME16 | 0,530 | 11,11 | 8,00 | 5,23 | 0,95 | 0,0326 | 6,15 |
| 3 | ME16 | 0,462 | 10,50 | 9,64 | 5,09 | 1,19 | 0,0297 | 6,43 |
| 3 | ME16 | 0,286 | 8,60 | 6,08 | 5,64 | 2,96 | 0,0265 | 9,27 |
| 3 | ME16 | 0,423 | 10,18 | 7,01 | 5,77 | 3,40 | 0,0384 | 9,08 |
| 3 | ME17 | 0,410 | 9,72 | 5,18 | 4,68 | 1,15 | 0,0358 | 8,72 |
| 3 | ME17 | 0,378 | 8,06 | 5,66 | 5,87 | 0,63 | 0,0676 | 17,87 |
| 3 | ME17 | 0,388 | 1,19 | 5,00 | 4,92 | 0,97 | 0,0313 | 8,07 |
| 3 | ME17 | 0,375 | 9,80 | 6,50 | 5,44 | 1,00 | 0,0456 | 12,15 |
| 3 | ME17 | 0,445 | 9,50 | 6,30 | 6,14 | 1,05 | 0,0444 | 9,98 |
| 3 | ME17 | 0,236 | 8,89 | 1,89 | 5,94 | 1,08 | 0,0428 | 18,15 |
| 3 | ME17 | 0,403 | 10,41 | 6,39 | 6,18 | 1,11 | 0,0544 | 13,51 |
| 3 | ME17 | 0,416 | 10,95 | 6,22 | 6,78 | 1,36 | 0,0540 | 12,99 |
| 3 | ME17 | 0,401 | 10,28 | 7,54 | 5,71 | 1,92 | 0,0662 | 16,51 |
| 3 | ME17 | 0,379 | 9,47 | 6,12 | 6,75 | 1,72 | 0,0589 | 15,56 |
| 3 | ME17 | 0,516 | 10,40 | 6,25 | 5,52 | 0,92 | 0,0385 | 7,46 |
| 3 | ME17 | 0,507 | 12,18 | 4,63 | 5,35 | 0,94 | 0,0357 | 7,04 |
| 3 | ME17 | 0,484 | 10,12 | 3,65 | 5,00 | 1,37 | 0,0272 | 5,62 |
| 3 | ME17 | 0,532 | 10,82 | 7,01 | 5,15 | 2,07 | 0,0283 | 5,32 |
| 3 | ME17 | 0,351 | 10,17 | 5,05 | 5,54 | 1,15 | 0,0234 | 6,68 |
| 3 | ME17 | 0,427 | 9,58 | 5,47 | 4,46 | 1,05 | 0,0261 | 6,12 |
| 3 | ME17 | 0,335 | 8,35 | 5,45 | 4,47 | 0,67 | 0,0182 | 5,43 |
| 3 | ME17 | 0,356 | 8,50 | 4,19 | 4,74 | 1,30 | 0,0344 | 9,67 |
| 3 | ME17 | 0,324 | 10,85 | 7,27 | 3,06 | 0,59 | 0,0194 | 5,98 |
| 3 | ME17 | 0,412 | 9,25 | 5,04 | 5,54 | 0,68 | 0,0327 | 7,93 |
| 3 | ME17 | 0,470 | 9,92 | 6,79 | 5,79 | 0,84 | 0,0345 | 7,35 |
| 3 | ME17 | 0,319 | 7,74 | 4,24 | 4,75 | 1,26 | 0,0295 | 9,26 |
| 3 | ME17 | 0,532 | 10,56 | 7,10 | 4,19 | 0,82 | 0,0268 | 5,04 |
| 3 | ME17 | 0,621 | 12,72 | 7,47 | 6,21 | 1,42 | 0,0290 | 4,67 |
| 3 | ME17 | 0,249 | 9,84 | 6,28 | 6,02 | 0,60 | 0,0291 | 11,70 |
| 3 | ME18 | 0,333 | 10,55 | 5,38 | 7,77 | 2,44 | 0,0290 | 8,72 |
| 3 | ME18 | 0,474 | 12,26 | 7,55 | 7,02 | 2,86 | 0,0244 | 5,15 |
| 3 | ME18 | 0,430 | 11,88 | 7,60 | 6,87 | 2,17 | 0,0283 | 6,58 |
| 3 | ME18 | 0,531 | 12,44 | 6,56 | 6,32 | 2,12 | 0,0300 | 5,65 |
| 3 | ME18 | 0,325 | 10,94 | 6,10 | 7,40 | 1,84 | 0,0210 | 6,47 |
| 3 | ME18 | 0,309 | 10,65 | 6,12 | 6,28 | 2,15 | 0,0387 | 12,52 |
| 3 | ME18 | 0,375 | 10,51 | 7,09 | 6,36 | 1,95 | 0,0301 | 8,02 |
| 3 | ME18 | 0,370 | 11,38 | 6,11 | 5,90 | 2,67 | 0,0388 | 10,49 |
| 3 | ME18 | 0,220 | 10,77 | 4,24 | 6,95 | 2,61 | 0,0244 | 11,09 |
| 3 | ME18 | 0,363 | 10,56 | 6,65 | 7,48 | 2,72 | 0,0452 | 12,47 |
| 3 | ME18 | 0,336 | 10,55 | 4,85 | 7,58 | 2,22 | 0,0551 | 16,42 |
| 3 | ME18 | 0,375 | 9,89 | 5,66 | 6,00 | 2,24 | 0,0235 | 6,27 |
| 3 | ME18 | 0,402 | 12,16 | 6,92 | 6,51 | 2,24 | 0,0340 | 8,47 |
| 3 | ME18 | 0,309 | 9,85 | 5,60 | 7,27 | 2,20 | 0,0458 | 14,82 |
| 3 | ME18 | 0,303 | 10,10 | 4,28 | 6,17 | 1,98 | 0,0220 | 7,26 |
| 3 | ME18 | 0,334 | 9,58 | 6,76 | 7,36 | 3,37 | 0,0489 | 14,62 |
| 3 | ME18 | 0,368 | 11,11 | 7,01 | 6,31 | 2,13 | 0,0441 | 12,00 |
| 3 | ME18 | 0,350 | 11,09 | 4,96 | 6,51 | 2,54 | 0,0233 | 6,65 |
| 3 | ME18 | 0,382 | 11,73 | 6,14 | 6,70 | 2,00 | 0,0334 | 8,73 |
| 3 | ME18 | 0,486 | 11,21 | 6,31 | 7,84 | 2,12 | 0,0380 | 7,82 |
| 3 | ME18 | 0,326 | 10,41 | 4,50 | 7,48 | 2,97 | 0,0324 | 9,94 |
| 3 | ME18 | 0,322 | 10,08 | 4,51 | 8,18 | 2,46 | 0,0631 | 19,58 |
| 3 | ME18 | 0,319 | 10,60 | 5,30 | 7,90 | 1,67 | 0,0343 | 10,74 |
| 3 | ME18 | 0,486 | 10,86 | 6,80 | 8,09 | 2,19 | 0,0437 | 9,00 |
| 3 | ME18 | 0,459 | 11,54 | 5,45 | 7,24 | 2,33 | 0,0391 | 8,52 |
| 3 | ME19 | 0,333 | 9,51 | 6,35 | 6,13 | 1,45 | 0,0289 | 8,68 |
| 3 | ME19 | 0,355 | 9,36 | 6,94 | 8,32 | 2,21 | 0,0457 | 12,87 |
| 3 | ME19 | 0,511 | 9,79 | 8,98 | 5,95 | 3,24 | 0,0290 | 5,68 |
| 3 | ME19 | 0,496 | 11,24 | 9,24 | 6,34 | 2,83 | 0,0362 | 7,30 |
| 3 | ME19 | 0,335 | 9,72 | 6,42 | 6,62 | 2,41 | 0,0382 | 11,40 |
| 3 | ME19 | 0,418 | 10,22 | 6,84 | 6,74 | 2,87 | 0,0290 | 6,94 |
| 3 | ME19 | 0,477 | 11,65 | 8,35 | 6,74 | 4,27 | 0,0263 | 5,51 |
| 3 | ME19 | 0,456 | 10,85 | 7,16 | 7,71 | 2,08 | 0,0324 | 7,11 |
| 3 | ME19 | 0,492 | 12,79 | 6,49 | 8,54 | 2,44 | 0,0487 | 9,90 |
| 3 | ME19 | 0,489 | 12,00 | 7,63 | 6,68 | 1,21 | 0,0239 | 4,89 |
| 3 | ME19 | 0,424 | 10,00 | 8,29 | 6,79 | 1,82 | 0,0336 | 7,92 |
| 3 | ME19 | 0,372 | 10,14 | 7,14 | 6,78 | 2,44 | 0,0389 | 10,46 |
| 3 | ME19 | 0,338 | 11,15 | 5,42 | 7,13 | 2,45 | 0,0471 | 13,93 |
| 3 | ME19 | 0,468 | 8,89 | 7,80 | 6,05 | 2,37 | 0,0347 | 7,41 |
| 3 | ME19 | 0,424 | 11,49 | 7,59 | 5,54 | 2,79 | 0,0360 | 8,49 |
| 3 | ME19 | 0,497 | 11,77 | 6,55 | 6,25 | 2,58 | 0,0263 | 5,29 |
| 3 | ME19 | 0,486 | 9,74 | 7,65 | 6,56 | 2,45 | 0,0358 | 7,37 |
| 3 | ME19 | 0,475 | 11,23 | 8,71 | 4,82 | 2,87 | 0,0336 | 7,07 |
| 3 | ME19 | 0,429 | 11,73 | 7,82 | 5,67 | 2,29 | 0,0424 | 9,88 |
| 3 | ME19 | 0,373 | 9,30 | 7,89 | 5,78 | 1,97 | 0,0486 | 13,03 |
| 3 | ME19 | 0,534 | 10,60 | 7,88 | 5,00 | 2,06 | 0,0462 | 8,65 |
| 3 | ME19 | 0,492 | 11,80 | 6,85 | 7,09 | 2,62 | 0,0333 | 6,77 |
| 3 | ME19 | 0,392 | 11,22 | 8,11 | 7,95 | 2,49 | 0,0410 | 10,46 |
| 3 | ME19 | 0,447 | 11,26 | 7,29 | 6,14 | 2,59 | 0,0206 | 4,61 |
| 3 | ME19 | 0,471 | 11,04 | 8,33 | 6,98 | 2,34 | 0,0149 | 3,16 |
| 3 | ME20 | 0,200 | 9,04 | 3,94 | 6,99 | 0,85 | 0,0313 | 15,64 |
| 3 | ME20 | 0,343 | 8,59 | 4,92 | 5,42 | 0,59 | 0,0491 | 14,33 |
| 3 | ME20 | 0,330 | 8,86 | 5,73 | 6,15 | 0,94 | 0,0423 | 12,81 |
| 3 | ME20 | 0,407 | 10,29 | 7,30 | 5,18 | 1,16 | 0,0464 | 11,39 |
| 3 | ME20 | 0,524 | 10,78 | 8,19 | 6,48 | 0,94 | 0,0689 | 13,14 |
| 3 | ME20 | 0,292 | 10,92 | 6,14 | 5,36 | 0,81 | 0,0463 | 15,87 |
| 3 | ME20 | 0,349 | 10,75 | 5,95 | 5,16 | 0,92 | 0,0460 | 13,18 |
| 3 | ME20 | 0,268 | 8,85 | 4,98 | 5,07 | 0,72 | 0,0382 | 14,25 |
| 3 | ME20 | 0,197 | 8,11 | 5,89 | 4,73 | 0,84 | 0,0284 | 14,44 |
| 3 | ME20 | 0,470 | 12,55 | 7,50 | 3,66 | 1,05 | 0,0522 | 11,11 |
| 3 | ME20 | 0,477 | 9,53 | 8,22 | 5,41 | 0,93 | 0,0367 | 7,70 |
| 3 | ME20 | 0,203 | 8,71 | 5,50 | 6,01 | 3,00 | 0,0263 | 12,96 |
| 3 | ME20 | 0,391 | 8,52 | 6,66 | 4,61 | 1,06 | 0,0440 | 11,25 |
| 3 | ME20 | 0,237 | 10,58 | 6,55 | 9,96 | 0,73 | 0,0325 | 13,73 |
| 3 | ME20 | 0,280 | 10,19 | 5,22 | 3,48 | 1,90 | 0,0324 | 11,57 |
| 3 | ME20 | 0,333 | 11,15 | 6,80 | 5,31 | 1,59 | 0,0443 | 13,30 |
| 3 | ME20 | 0,263 | 9,16 | 6,15 | 4,82 | 0,72 | 0,0470 | 17,85 |
| 3 | ME20 | 0,255 | 8,70 | 4,81 | 7,17 | 0,97 | 0,0452 | 17,72 |
| 3 | ME20 | 0,495 | 10,98 | 7,23 | 4,17 | 1,87 | 0,0401 | 8,09 |
| 3 | ME20 | 0,352 | 9,31 | 6,84 | 6,34 | 0,85 | 0,0456 | 12,97 |
| 3 | ME20 | 0,279 | 9,50 | 3,71 | 4,73 | 0,88 | 0,0335 | 12,02 |
| 3 | ME20 | 0,374 | 7,97 | 6,10 | 4,87 | 0,77 | 0,0328 | 8,77 |
| 3 | ME20 | 0,368 | 6,96 | 5,12 | 5,55 | 1,21 | 0,0428 | 11,63 |
| 3 | ME20 | 0,258 | 8,47 | 4,27 | 6,87 | 1,05 | 0,0328 | 12,73 |
| 3 | ME20 | 0,371 | 11,68 | 5,94 | 5,37 | 0,82 | 0,0395 | 10,64 |
| 3 | ME21 | 0,483 | 11,00 | 5,11 | 3,33 | 1,49 | 0,0308 | 6,38 |
| 3 | ME21 | 0,402 | 8,76 | 5,80 | 5,93 | 1,16 | 0,0339 | 8,43 |
| 3 | ME21 | 0,294 | 6,49 | 4,47 | 4,71 | 2,65 | 0,0116 | 3,95 |
| 3 | ME21 | 0,433 | 9,55 | 4,86 | 6,61 | 2,56 | 0,0462 | 10,67 |
| 3 | ME21 | 0,444 | 9,72 | 4,88 | 5,29 | 0,71 | 0,0285 | 6,42 |
| 3 | ME21 | 0,390 | 9,90 | 4,67 | 5,25 | 0,97 | 0,0413 | 10,59 |
| 3 | ME21 | 0,473 | 10,14 | 6,24 | 6,03 | 1,53 | 0,0645 | 13,64 |
| 3 | ME21 | 0,363 | 9,94 | 4,94 | 5,21 | 2,10 | 0,0260 | 7,16 |
| 3 | ME21 | 0,491 | 10,61 | 5,30 | 6,01 | 1,21 | 0,0380 | 7,74 |
| 3 | ME21 | 0,402 | 8,07 | 6,63 | 5,09 | 1,41 | 0,0378 | 9,40 |
| 3 | ME21 | 0,565 | 11,39 | 5,53 | 5,74 | 0,90 | 0,0434 | 7,68 |
| 3 | ME21 | 0,421 | 9,87 | 4,78 | 5,78 | 0,91 | 0,0439 | 10,43 |
| 3 | ME21 | 0,481 | 10,45 | 6,00 | 4,59 | 0,92 | 0,0340 | 7,07 |
| 3 | ME21 | 0,364 | 9,91 | 6,31 | 6,38 | 1,77 | 0,0532 | 14,62 |
| 3 | ME21 | 0,365 | 8,25 | 5,75 | 5,31 | 0,77 | 0,0233 | 6,38 |
| 3 | ME21 | 0,416 | 8,77 | 5,40 | 5,23 | 1,21 | 0,0434 | 10,43 |
| 3 | ME21 | 0,422 | 8,11 | 6,20 | 5,32 | 1,03 | 0,0283 | 6,71 |
| 3 | ME21 | 0,464 | 9,82 | 6,49 | 5,34 | 1,27 | 0,0410 | 8,84 |
| 3 | ME21 | 0,420 | 7,96 | 6,14 | 4,64 | 0,90 | 0,0430 | 10,24 |
| 3 | ME21 | 0,401 | 8,72 | 6,10 | 5,91 | 0,83 | 0,0459 | 11,45 |
| 3 | ME21 | 0,523 | 11,03 | 7,04 | 6,78 | 0,96 | 0,0475 | 9,08 |
| 3 | ME21 | 0,513 | 10,63 | 6,53 | 5,28 | 0,83 | 0,0477 | 9,30 |
| 3 | ME21 | 0,445 | 9,81 | 6,69 | 5,70 | 0,61 | 0,0260 | 5,84 |
| 3 | ME21 | 0,453 | 8,69 | 6,40 | 5,72 | 1,49 | 0,0364 | 8,04 |
| 3 | ME21 | 0,332 | 8,19 | 4,56 | 4,52 | 0,61 | 0,0153 | 4,61 |
| 3 | MA1 | 0,477 | 12,08 | 8,57 | 5,62 | 1,32 | 0,0215 | 4,51 |
| 3 | MA1 | 0,368 | 10,14 | 8,13 | 5,40 | 1,58 | 0,0315 | 8,56 |
| 3 | MA1 | 0,396 | 12,60 | 6,79 | 6,55 | 1,79 | 0,0536 | 13,54 |
| 3 | MA1 | 0,406 | 11,46 | 7,30 | 6,39 | 1,42 | 0,0487 | 12,00 |
| 3 | MA1 | 0,289 | 10,28 | 6,69 | 5,93 | 1,05 | 0,0238 | 8,24 |
| 3 | MA1 | 0,416 | 11,57 | 5,19 | 6,56 | 1,39 | 0,0457 | 10,99 |
| 3 | MA1 | 0,367 | 11,16 | 6,52 | 5,48 | 1,55 | 0,0446 | 12,15 |
| 3 | MA1 | 0,356 | 11,16 | 6,37 | 6,26 | 2,48 | 0,0441 | 12,39 |
| 3 | MA1 | 0,501 | 11,74 | 8,57 | 4,36 | 2,06 | 0,0265 | 5,29 |
| 3 | MA1 | 0,444 | 11,37 | 6,25 | 7,00 | 2,43 | 0,0517 | 11,64 |
| 3 | MA1 | 0,449 | 12,69 | 7,96 | 5,84 | 1,67 | 0,0558 | 12,43 |
| 3 | MA1 | 0,467 | 13,30 | 9,53 | 5,68 | 1,43 | 0,0337 | 7,22 |
| 3 | MA1 | 0,544 | 12,36 | 7,38 | 4,88 | 0,92 | 0,0257 | 4,72 |
| 3 | MA1 | 0,414 | 11,51 | 7,70 | 5,70 | 2,42 | 0,0413 | 9,98 |
| 3 | MA1 | 0,484 | 11,77 | 7,90 | 5,55 | 2,84 | 0,0426 | 8,80 |
| 3 | MA1 | 0,411 | 12,05 | 6,78 | 5,66 | 2,64 | 0,0481 | 11,70 |
| 3 | MA1 | 0,443 | 11,92 | 7,19 | 5,91 | 2,02 | 0,0602 | 13,59 |
| 3 | MA1 | 0,288 | 9,78 | 5,42 | 4,85 | 0,89 | 0,0286 | 9,93 |
| 3 | MA1 | 0,483 | 11,01 | 7,64 | 6,50 | 1,31 | 0,0371 | 7,68 |
| 3 | MA1 | 0,578 | 12,68 | 9,33 | 5,06 | 1,61 | 0,0493 | 8,53 |
| 3 | MA1 | 0,413 | 12,44 | 6,06 | 5,27 | 1,75 | 0,0426 | 10,31 |
| 3 | MA1 | 0,436 | 10,44 | 7,21 | 6,11 | 1,04 | 0,0476 | 10,92 |
| 3 | MA1 | 0,366 | 11,03 | 6,49 | 7,46 | 1,10 | 0,0505 | 13,80 |
| 3 | MA1 | 0,468 | 12,54 | 6,59 | 5,74 | 1,92 | 0,0423 | 9,04 |
| 3 | MA1 | 0,346 | 11,01 | 8,42 | 5,38 | 1,02 | 0,0297 | 8,58 |
| 3 | MA2 | 0,458 | 10,50 | 7,10 | 6,87 | 3,68 | 0,0559 | 12,21 |
| 3 | MA2 | 0,289 | 8,35 | 4,56 | 3,86 | 1,19 | 0,0233 | 8,06 |
| 3 | MA2 | 0,451 | 9,99 | 6,08 | 6,13 | 1,91 | 0,0258 | 5,72 |
| 3 | MA2 | 0,251 | 7,68 | 3,82 | 5,52 | 1,37 | 0,0266 | 10,61 |
| 3 | MA2 | 0,420 | 10,53 | 6,88 | 3,92 | 0,70 | 0,0274 | 6,53 |
| 3 | MA2 | 0,442 | 10,40 | 5,40 | 4,18 | 2,58 | 0,0259 | 5,86 |
| 3 | MA2 | 0,397 | 10,09 | 6,68 | 5,35 | 0,59 | 0,0263 | 6,63 |
| 3 | MA2 | 0,432 | 9,51 | 8,53 | 5,25 | 0,74 | 0,0337 | 7,80 |
| 3 | MA2 | 0,406 | 9,66 | 7,68 | 4,38 | 1,59 | 0,0340 | 8,37 |
| 3 | MA2 | 0,465 | 10,59 | 6,61 | 5,01 | 1,60 | 0,0332 | 7,15 |
| 3 | MA2 | 0,401 | 9,37 | 4,70 | 6,42 | 0,72 | 0,0467 | 11,66 |
| 3 | MA2 | 0,323 | 9,77 | 5,68 | 4,89 | 0,72 | 0,0408 | 12,65 |
| 3 | MA2 | 0,405 | 10,63 | 5,48 | 6,22 | 0,83 | 0,0399 | 9,86 |
| 3 | MA2 | 0,395 | 9,92 | 5,37 | 3,99 | 0,58 | 0,0263 | 6,66 |
| 3 | MA2 | 0,445 | 9,88 | 6,44 | 4,71 | 0,72 | 0,0315 | 7,09 |
| 3 | MA2 | 0,369 | 7,39 | 5,70 | 4,70 | 1,15 | 0,0363 | 9,83 |
| 3 | MA2 | 0,386 | 9,57 | 6,39 | 4,29 | 1,04 | 0,0233 | 6,03 |
| 3 | MA2 | 0,333 | 13,08 | 4,76 | 6,18 | 0,99 | 0,0375 | 11,26 |
| 3 | MA2 | 0,283 | 9,53 | 5,77 | 7,37 | 0,56 | 0,0248 | 8,75 |
| 3 | MA2 | 0,300 | 7,23 | 5,92 | 4,10 | 0,71 | 0,0171 | 5,70 |
| 3 | MA2 | 0,294 | 9,31 | 6,27 | 4,92 | 1,47 | 0,0341 | 11,59 |
| 3 | MA2 | 0,356 | 9,14 | 5,96 | 4,96 | 0,94 | 0,0354 | 9,95 |
| 3 | MA2 | 0,268 | 10,90 | 4,38 | 5,02 | 0,69 | 0,0486 | 18,13 |
| 3 | MA2 | 0,332 | 8,02 | 5,63 | 5,35 | 0,66 | 0,0197 | 5,94 |
| 3 | MA2 | 0,398 | 11,71 | 5,33 | 5,29 | 1,03 | 0,0214 | 5,38 |
| 3 | MA3 | 0,469 | 11,54 | 6,16 | 5,51 | 1,64 | 0,0276 | 5,88 |
| 3 | MA3 | 0,463 | 10,06 | 5,04 | 4,50 | 1,51 | 0,0430 | 9,29 |
| 3 | MA3 | 0,445 | 10,22 | 6,67 | 5,80 | 0,98 | 0,0613 | 13,78 |
| 3 | MA3 | 0,495 | 9,65 | 5,94 | 4,75 | 1,73 | 0,0366 | 7,39 |
| 3 | MA3 | 0,352 | 7,26 | 5,60 | 2,98 | 1,28 | 0,0345 | 9,80 |
| 3 | MA3 | 0,391 | 9,14 | 6,59 | 5,31 | 1,10 | 0,0481 | 12,30 |
| 3 | MA3 | 0,436 | 7,32 | 6,36 | 5,25 | 0,80 | 0,0384 | 8,81 |
| 3 | MA3 | 0,515 | 9,44 | 5,56 | 5,28 | 0,92 | 0,0361 | 7,01 |
| 3 | MA3 | 0,481 | 9,35 | 7,48 | 3,88 | 0,95 | 0,0267 | 5,55 |
| 3 | MA3 | 0,484 | 10,73 | 5,12 | 4,78 | 2,61 | 0,0346 | 7,15 |
| 3 | MA3 | 0,381 | 7,40 | 6,66 | 4,13 | 1,04 | 0,0394 | 10,34 |
| 3 | MA3 | 0,436 | 8,96 | 4,80 | 5,61 | 2,14 | 0,0539 | 12,36 |
| 3 | MA3 | 0,360 | 6,98 | 5,64 | 4,30 | 2,57 | 0,0450 | 12,50 |
| 3 | MA3 | 0,482 | 9,54 | 6,85 | 4,61 | 1,19 | 0,0442 | 9,17 |
| 3 | MA3 | 0,428 | 9,57 | 6,10 | 4,91 | 0,84 | 0,0314 | 7,34 |
| 3 | MA3 | 0,492 | 10,72 | 6,23 | 4,43 | 0,63 | 0,0355 | 7,22 |
| 3 | MA3 | 0,442 | 9,17 | 6,34 | 4,27 | 1,22 | 0,0275 | 6,22 |
| 3 | MA3 | 0,316 | 6,21 | 3,65 | 4,29 | 2,46 | 0,0423 | 13,39 |
| 3 | MA3 | 0,507 | 8,99 | 6,37 | 4,69 | 1,24 | 0,0428 | 8,44 |
| 3 | MA3 | 0,356 | 8,42 | 6,18 | 4,84 | 1,31 | 0,0318 | 8,93 |
| 3 | MA3 | 0,477 | 9,78 | 6,06 | 3,88 | 0,95 | 0,0376 | 7,88 |
| 3 | MA3 | 0,383 | 8,38 | 4,81 | 4,13 | 0,73 | 0,0257 | 6,71 |
| 3 | MA3 | 0,359 | 8,17 | 5,30 | 4,06 | 0,66 | 0,0208 | 5,79 |
| 3 | MA3 | 0,435 | 8,23 | 4,25 | 5,16 | 0,87 | 0,0496 | 11,40 |
| 3 | MA3 | 0,436 | 9,27 | 5,80 | 5,17 | 0,37 | 0,0422 | 9,68 |
| 3 | MA4 | 0,519 | 12,65 | 6,64 | 6,75 | 0,75 | 0,0370 | 7,13 |
| 3 | MA4 | 0,463 | 11,16 | 7,28 | 6,50 | 2,20 | 0,0294 | 6,35 |
| 3 | MA4 | 0,465 | 12,33 | 7,65 | 7,12 | 2,20 | 0,0328 | 7,06 |
| 3 | MA4 | 0,441 | 11,87 | 6,62 | 8,16 | 2,50 | 0,0580 | 13,15 |
| 3 | MA4 | 0,412 | 10,65 | 6,89 | 5,53 | 1,57 | 0,0193 | 4,69 |
| 3 | MA4 | 0,369 | 9,25 | 6,28 | 5,46 | 2,39 | 0,0327 | 8,86 |
| 3 | MA4 | 0,242 | 11,65 | 6,40 | 7,86 | 2,46 | 0,0369 | 15,27 |
| 3 | MA4 | 0,427 | 11,03 | 7,00 | 5,96 | 2,25 | 0,0233 | 5,46 |
| 3 | MA4 | 0,447 | 10,04 | 5,06 | 7,06 | 0,95 | 0,0397 | 8,88 |
| 3 | MA4 | 0,362 | 11,62 | 6,23 | 6,30 | 1,57 | 0,0409 | 11,29 |
| 3 | MA4 | 0,192 | 7,66 | 4,24 | 5,32 | 1,90 | 0,0307 | 15,96 |
| 3 | MA4 | 0,222 | 8,49 | 4,37 | 6,42 | 0,91 | 0,0314 | 14,14 |
| 3 | MA4 | 0,404 | 9,47 | 7,29 | 4,66 | 1,76 | 0,0202 | 5,00 |
| 3 | MA4 | 0,385 | 10,26 | 6,28 | 5,46 | 1,44 | 0,0280 | 7,27 |
| 3 | MA4 | 0,245 | 9,92 | 4,62 | 6,61 | 0,46 | 0,0160 | 6,54 |
| 3 | MA4 | 0,490 | 11,26 | 5,72 | 4,82 | 1,19 | 0,0178 | 3,63 |
| 3 | MA4 | 0,433 | 11,46 | 6,83 | 5,95 | 2,02 | 0,0346 | 7,99 |
| 3 | MA4 | 0,473 | 11,29 | 7,11 | 6,17 | 1,74 | 0,0361 | 7,64 |
| 3 | MA4 | 0,333 | 8,90 | 4,49 | 6,26 | 1,67 | 0,0417 | 12,54 |
| 3 | MA4 | 0,394 | 10,83 | 6,69 | 6,34 | 1,59 | 0,0486 | 12,34 |
| 3 | MA4 | 0,260 | 11,23 | 6,70 | 6,85 | 1,83 | 0,0307 | 11,83 |
| 3 | MA4 | 0,406 | 10,26 | 6,75 | 5,87 | 2,15 | 0,0288 | 7,10 |
| 3 | MA4 | 0,424 | 11,55 | 8,30 | 6,90 | 1,19 | 0,0315 | 7,43 |
| 3 | MA4 | 0,413 | 11,28 | 5,88 | 5,93 | 1,62 | 0,0297 | 7,20 |
| 3 | MA4 | 0,451 | 9,52 | 5,46 | 7,17 | 1,48 | 0,0346 | 7,68 |
| 3 | MA5 | 0,350 | 9,62 | 5,78 | 5,66 | 2,38 | 0,0300 | 8,57 |
| 3 | MA5 | 0,394 | 11,15 | 4,84 | 7,34 | 1,32 | 0,0440 | 11,17 |
| 3 | MA5 | 0,431 | 11,44 | 6,34 | 7,41 | 2,19 | 0,0450 | 10,44 |
| 3 | MA5 | 0,334 | 11,03 | 3,60 | 6,42 | 3,05 | 0,0380 | 11,38 |
| 3 | MA5 | 0,363 | 11,29 | 3,67 | 5,32 | 2,04 | 0,0330 | 9,09 |
| 3 | MA5 | 0,459 | 10,63 | 7,30 | 7,95 | 1,88 | 0,0440 | 9,59 |
| 3 | MA5 | 0,366 | 10,51 | 7,28 | 5,60 | 1,96 | 0,0230 | 6,28 |
| 3 | MA5 | 0,363 | 10,67 | 7,46 | 6,29 | 2,17 | 0,0520 | 14,33 |
| 3 | MA5 | 0,358 | 9,94 | 5,80 | 7,08 | 2,30 | 0,0450 | 12,57 |
| 3 | MA5 | 0,429 | 9,87 | 6,97 | 7,22 | 2,25 | 0,0430 | 10,02 |
| 3 | MA5 | 0,387 | 10,37 | 5,65 | 7,92 | 2,22 | 0,0580 | 14,99 |
| 3 | MA5 | 0,425 | 9,86 | 5,91 | 6,35 | 1,86 | 0,0460 | 10,82 |
| 3 | MA5 | 0,447 | 10,34 | 6,61 | 6,60 | 3,32 | 0,0430 | 9,62 |
| 3 | MA5 | 0,386 | 9,92 | 7,11 | 6,34 | 1,45 | 0,0280 | 7,25 |
| 3 | MA5 | 0,329 | 10,73 | 7,07 | 6,86 | 2,15 | 0,0620 | 18,84 |
| 3 | MA5 | 0,403 | 9,40 | 8,38 | 6,91 | 3,23 | 0,0510 | 12,66 |
| 3 | MA5 | 0,425 | 10,83 | 7,14 | 6,01 | 1,50 | 0,0360 | 8,47 |
| 3 | MA5 | 0,436 | 10,83 | 6,60 | 6,88 | 3,35 | 0,0480 | 11,01 |
| 3 | MA5 | 0,421 | 10,55 | 6,82 | 6,28 | 2,66 | 0,0430 | 10,21 |
| 3 | MA5 | 0,403 | 10,35 | 6,30 | 4,93 | 2,36 | 0,0290 | 7,20 |
| 3 | MA5 | 0,487 | 13,16 | 6,94 | 7,48 | 2,26 | 0,0360 | 7,39 |
| 3 | MA5 | 0,389 | 8,50 | 6,32 | 5,60 | 1,53 | 0,0320 | 8,23 |
| 3 | MA5 | 0,414 | 10,54 | 6,16 | 5,53 | 3,16 | 0,0370 | 8,94 |
| 3 | MA5 | 0,448 | 11,35 | 7,72 | 5,89 | 2,55 | 0,0260 | 5,80 |
| 3 | MA5 | 0,451 | 11,20 | 7,58 | 6,08 | 1,63 | 0,0380 | 8,43 |
| 3 | BA1 | 0,335 | 7,93 | 6,58 | 5,00 | 0,97 | 0,0355 | 10,61 |
| 3 | BA1 | 0,292 | 8,12 | 4,56 | 5,95 | 0,58 | 0,0558 | 19,10 |
| 3 | BA1 | 0,236 | 8,64 | 3,16 | 5,48 | 1,01 | 0,0369 | 15,63 |
| 3 | BA1 | 0,403 | 10,00 | 5,03 | 5,66 | 0,86 | 0,0458 | 11,36 |
| 3 | BA1 | 0,365 | 10,74 | 6,71 | 6,46 | 0,72 | 0,0396 | 10,85 |
| 3 | BA1 | 0,375 | 9,22 | 4,83 | 4,53 | 1,76 | 0,0436 | 11,63 |
| 3 | BA1 | 0,162 | 8,25 | 2,51 | 4,76 | 0,71 | 0,0344 | 21,18 |
| 3 | BA1 | 0,261 | 7,61 | 3,93 | 6,09 | 1,15 | 0,0471 | 18,03 |
| 3 | BA1 | 0,404 | 9,42 | 5,81 | 4,95 | 0,96 | 0,0399 | 9,87 |
| 3 | BA1 | 0,367 | 9,38 | 3,87 | 5,71 | 0,90 | 0,0403 | 10,97 |
| 3 | BA1 | 0,481 | 10,10 | 5,51 | 5,34 | 1,14 | 0,0305 | 6,34 |
| 3 | BA1 | 0,249 | 8,19 | 4,84 | 5,11 | 1,00 | 0,0333 | 13,40 |
| 3 | BA1 | 0,355 | 9,88 | 5,11 | 6,11 | 0,71 | 0,0329 | 9,26 |
| 3 | BA1 | 0,404 | 9,74 | 5,16 | 5,90 | 0,68 | 0,0423 | 10,46 |
| 3 | BA1 | 0,374 | 10,45 | 5,25 | 6,00 | 0,84 | 0,0385 | 10,28 |
| 3 | BA1 | 0,232 | 8,88 | 3,14 | 4,99 | 0,75 | 0,0338 | 14,59 |
| 3 | BA1 | 0,315 | 8,63 | 4,01 | 5,02 | 1,87 | 0,0302 | 9,60 |
| 3 | BA1 | 0,239 | 7,86 | 3,35 | 5,09 | 0,87 | 0,0509 | 21,31 |
| 3 | BA1 | 0,257 | 6,97 | 3,31 | 5,50 | 0,76 | 0,0379 | 14,75 |
| 3 | BA1 | 0,442 | 9,08 | 5,34 | 4,55 | 0,87 | 0,0418 | 9,45 |
| 3 | BA1 | 0,380 | 9,17 | 3,63 | 4,80 | 0,75 | 0,0293 | 7,70 |
| 3 | BA1 | 0,209 | 8,32 | 4,08 | 5,97 | 0,67 | 0,0430 | 20,57 |
| 3 | BA1 | 0,288 | 7,14 | 7,37 | 5,00 | 0,76 | 0,0343 | 11,93 |
| 3 | BA1 | 0,212 | 8,30 | 4,19 | 4,82 | 0,92 | 0,0321 | 15,16 |
| 3 | BA1 | 0,240 | 8,24 | 6,68 | 4,99 | 0,89 | 0,0298 | 12,40 |
| 3 | VA1 | 0,298 | 7,50 | 6,90 | 2,88 | 0,85 | 0,0241 | 8,09 |
| 3 | VA1 | 0,453 | 10,11 | 5,99 | 4,16 | 0,80 | 0,0368 | 8,12 |
| 3 | VA1 | 0,447 | 8,67 | 5,79 | 6,46 | 1,19 | 0,0207 | 4,63 |
| 3 | VA1 | 0,285 | 4,70 | 6,99 | 4,81 | 0,72 | 0,0266 | 9,33 |
| 3 | VA1 | 0,433 | 9,70 | 6,28 | 5,24 | 0,73 | 0,0340 | 7,85 |
| 3 | VA1 | 0,425 | 10,02 | 6,21 | 4,06 | 0,93 | 0,0330 | 7,76 |
| 3 | VA1 | 0,263 | 7,08 | 4,45 | 6,20 | 0,90 | 0,0407 | 15,48 |
| 3 | VA1 | 0,287 | 9,10 | 6,87 | 3,15 | 0,71 | 0,0208 | 7,25 |
| 3 | VA1 | 0,415 | 10,85 | 7,33 | 4,64 | 0,81 | 0,0236 | 5,69 |
| 3 | VA1 | 0,421 | 9,41 | 5,99 | 5,14 | 0,97 | 0,0484 | 11,50 |
| 3 | VA1 | 0,441 | 8,13 | 7,07 | 3,92 | 0,94 | 0,0282 | 6,39 |
| 3 | VA1 | 0,335 | 8,03 | 7,65 | 4,95 | 0,95 | 0,0387 | 11,55 |
| 3 | VA1 | 0,401 | 9,28 | 8,06 | 4,52 | 0,78 | 0,0485 | 12,09 |
| 3 | VA1 | 0,486 | 10,17 | 6,47 | 4,18 | 1,26 | 0,0384 | 7,90 |
| 3 | VA1 | 0,364 | 8,62 | 6,70 | 4,66 | 1,20 | 0,0354 | 9,73 |
| 3 | VA1 | 0,454 | 10,15 | 7,20 | 4,62 | 1,04 | 0,0325 | 7,16 |
| 3 | VA1 | 0,414 | 9,28 | 7,31 | 3,80 | 0,90 | 0,0314 | 7,58 |
| 3 | VA1 | 0,316 | 9,77 | 5,26 | 3,93 | 0,65 | 0,0465 | 14,72 |
| 3 | VA1 | 0,375 | 8,57 | 6,35 | 5,15 | 0,88 | 0,0322 | 8,59 |
| 3 | VA1 | 0,268 | 9,66 | 4,28 | 5,53 | 0,93 | 0,0334 | 12,46 |
| 3 | VA1 | 0,423 | 10,31 | 8,95 | 4,11 | 1,19 | 0,0444 | 10,50 |
| 3 | VA1 | 0,532 | 10,95 | 8,35 | 4,12 | 1,02 | 0,0388 | 7,29 |
| 3 | VA1 | 0,275 | 7,86 | 6,86 | 4,48 | 0,94 | 0,0247 | 8,98 |
| 3 | VA1 | 0,287 | 10,22 | 6,31 | 5,35 | 0,85 | 0,0297 | 10,35 |
| 3 | VA1 | 0,458 | 10,83 | 6,52 | 4,56 | 0,91 | 0,0364 | 7,95 |
| 3 | MC1 | 0,377 | 10,00 | 7,34 | 5,94 | 0,93 | 0,0370 | 9,81 |
| 3 | MC1 | 0,354 | 11,17 | 4,61 | 5,95 | 0,62 | 0,0620 | 17,51 |
| 3 | MC1 | 0,343 | 9,91 | 6,03 | 5,89 | 0,86 | 0,0730 | 21,28 |
| 3 | MC1 | 0,312 | 8,74 | 6,16 | 5,36 | 0,67 | 0,0280 | 8,97 |
| 3 | MC1 | 0,364 | 9,46 | 4,45 | 5,84 | 0,67 | 0,0360 | 9,89 |
| 3 | MC1 | 0,260 | 7,84 | 4,43 | 5,17 | 0,51 | 0,0470 | 18,08 |
| 3 | MC1 | 0,265 | 8,77 | 6,17 | 5,00 | 0,55 | 0,0240 | 9,06 |
| 3 | MC1 | 0,353 | 8,74 | 4,50 | 5,21 | 0,69 | 0,0480 | 13,60 |
| 3 | MC1 | 0,374 | 9,07 | 5,74 | 5,20 | 0,72 | 0,0410 | 10,96 |
| 3 | MC1 | 0,300 | 9,12 | 4,32 | 5,40 | 0,69 | 0,0290 | 9,67 |
| 3 | MC1 | 0,383 | 9,06 | 6,09 | 5,39 | 1,05 | 0,0390 | 10,18 |
| 3 | MC1 | 0,370 | 8,14 | 3,90 | 6,41 | 1,33 | 0,0420 | 11,35 |
| 3 | MC1 | 0,367 | 9,37 | 4,95 | 2,99 | 0,64 | 0,0230 | 6,27 |
| 3 | MC1 | 0,346 | 10,38 | 4,07 | 5,50 | 0,53 | 0,0190 | 5,49 |
| 3 | MC1 | 0,361 | 9,07 | 5,44 | 5,76 | 0,64 | 0,0230 | 6,37 |
| 3 | MC1 | 0,314 | 7,32 | 4,50 | 4,71 | 0,36 | 0,0210 | 6,69 |
| 3 | MC1 | 0,341 | 9,48 | 5,86 | 4,54 | 1,04 | 0,0260 | 7,62 |
| 3 | MC1 | 0,382 | 8,71 | 4,82 | 4,52 | 0,63 | 0,0210 | 5,50 |
| 3 | MC1 | 0,346 | 9,92 | 3,73 | 4,71 | 0,69 | 0,0240 | 6,94 |
| 3 | MC1 | 0,300 | 9,47 | 3,52 | 5,35 | 0,77 | 0,0240 | 8,00 |
| 3 | MC1 | 0,365 | 8,80 | 5,70 | 4,26 | 0,87 | 0,0180 | 4,93 |
| 3 | MC1 | 0,311 | 8,65 | 3,88 | 3,85 | 0,64 | 0,0240 | 7,72 |
| 3 | MC1 | 0,314 | 9,06 | 4,50 | 4,10 | 0,64 | 0,0190 | 6,05 |
| 3 | MC1 | 0,311 | 9,32 | 5,47 | 5,59 | 3,45 | 0,0230 | 7,40 |
| 3 | MC1 | 0,361 | 9,84 | 6,14 | 6,03 | 0,66 | 0,0250 | 6,93 |
| 3 | MO2 | 0,437 | 9,61 | 5,65 | 4,82 |  | 0,0680 | 15,56 |
| 3 | MO2 | 0,321 | 8,50 | 5,45 | 4,69 | 0,93 | 0,0243 | 7,58 |
| 3 | MO2 | 0,382 | 9,62 | 5,65 | 5,15 | 0,97 | 0,0375 | 9,81 |
| 3 | MO2 | 0,479 | 9,50 | 7,03 | 4,71 | 1,83 | 0,0380 | 7,93 |
| 3 | MO2 | 0,358 | 8,73 | 4,91 | 5,38 | 0,89 | 0,0444 | 12,40 |
| 3 | MO2 | 0,548 | 12,89 | 6,54 | 7,03 | 0,95 | 0,0426 | 7,78 |
| 3 | MO2 | 0,413 | 10,15 | 4,32 | 5,15 | 1,11 | 0,0416 | 10,06 |
| 3 | MO2 | 0,357 | 10,21 | 7,80 | 5,67 | 0,66 | 0,0367 | 10,29 |
| 3 | MO2 | 0,511 | 9,46 | 6,27 | 4,53 | 1,46 | 0,0328 | 6,42 |
| 3 | MO2 | 0,395 | 10,57 | 4,18 | 5,79 | 1,19 | 0,0329 | 8,34 |
| 3 | MO2 | 0,431 | 9,60 | 6,35 | 5,31 | 0,84 | 0,0456 | 10,58 |
| 3 | MO2 | 0,393 | 9,71 | 4,91 | 5,02 | 1,19 | 0,0331 | 8,43 |
| 3 | MO2 | 0,248 | 8,08 | 2,99 | 5,15 | 1,32 | 0,0376 | 15,17 |
| 3 | MO2 | 0,436 | 9,61 | 4,50 | 5,49 | 0,98 | 0,0423 | 9,70 |
| 3 | MO2 | 0,406 | 9,32 | 4,18 | 5,60 | 1,66 | 0,0458 | 11,28 |
| 3 | MO2 | 0,414 | 8,63 | 4,74 | 5,21 | 0,96 | 0,0408 | 9,85 |
| 3 | MO2 | 0,362 | 9,04 | 4,12 | 5,52 | 0,90 | 0,0346 | 9,57 |
| 3 | MO2 | 0,358 | 8,30 | 4,68 | 5,12 | 1,23 | 0,0447 | 12,49 |
| 3 | MO2 | 0,387 | 9,62 | 5,11 | 3,80 | 0,65 | 0,0215 | 5,56 |
| 3 | MO2 | 0,378 | 7,50 | 4,33 | 5,05 | 0,73 | 0,0309 | 8,18 |
| 3 | MO2 | 0,441 | 11,12 | 4,91 | 5,43 | 0,68 | 0,0274 | 6,21 |
| 3 | MO2 | 0,444 | 8,70 | 4,03 | 5,63 | 1,76 | 0,0483 | 10,88 |
| 3 | MO2 | 0,397 | 8,08 | 5,14 | 5,27 | 1,35 | 0,0451 | 11,37 |
| 3 | MO2 | 0,366 | 9,31 | 4,16 | 5,91 | 1,40 | 0,0443 | 12,12 |
| 3 | MO2 | 0,456 | 9,50 | 5,56 | 6,37 | 1,97 | 0,0585 | 12,82 |
| 3 | MO3 | 0,484 | 10,81 | 7,00 | 4,80 | 0,75 | 0,0213 | 4,40 |
| 3 | MO3 | 0,497 | 11,43 | 7,44 | 4,82 | 0,95 | 0,0268 | 5,39 |
| 3 | MO3 | 0,396 | 10,66 | 5,51 | 5,74 | 0,75 | 0,0350 | 8,85 |
| 3 | MO3 | 0,518 | 12,43 | 5,56 | 6,34 | 1,04 | 0,0353 | 6,81 |
| 3 | MO3 | 0,488 | 11,43 | 6,92 | 5,77 | 1,11 | 0,0307 | 6,29 |
| 3 | MO3 | 0,330 | 8,51 | 5,33 | 5,52 | 0,75 | 0,0274 | 8,30 |
| 3 | MO3 | 0,427 | 9,74 | 5,40 | 6,37 | 0,75 | 0,0315 | 7,37 |
| 3 | MO3 | 0,430 | 12,17 | 6,44 | 6,09 | 0,74 | 0,0334 | 7,77 |
| 3 | MO3 | 0,422 | 9,60 | 6,05 | 5,35 | 1,04 | 0,0271 | 6,42 |
| 3 | MO3 | 0,520 | 11,13 | 6,70 | 4,36 | 0,56 | 0,0319 | 6,14 |
| 3 | MO3 | 0,400 | 10,65 | 6,04 | 4,10 | 0,93 | 0,0287 | 7,18 |
| 3 | MO3 | 0,359 | 10,39 | 7,28 | 4,82 | 0,77 | 0,0409 | 11,40 |
| 3 | MO3 | 0,408 | 9,59 | 5,57 | 5,52 | 2,14 | 0,0414 | 10,16 |
| 3 | MO3 | 0,457 | 9,91 | 5,18 | 4,83 | 1,03 | 0,0533 | 11,66 |
| 3 | MO3 | 0,509 | 10,66 | 6,64 | 5,65 | 0,99 | 0,0447 | 8,79 |
| 3 | MO3 | 0,480 | 11,14 | 5,91 | 5,46 | 0,74 | 0,0405 | 8,44 |
| 3 | MO3 | 0,251 | 8,97 | 3,56 | 5,49 | 0,69 | 0,0348 | 13,85 |
| 3 | MO3 | 0,363 | 10,13 | 3,57 | 3,32 | 0,74 | 0,0338 | 9,31 |
| 3 | MO3 | 0,275 | 8,06 | 4,93 | 5,11 | 1,06 | 0,0157 | 5,71 |
| 3 | MO3 | 0,277 | 10,12 | 3,82 | 5,57 | 1,02 | 0,0339 | 12,25 |
| 3 | MO3 | 0,461 | 11,26 | 6,03 | 5,58 | 1,33 | 0,0246 | 5,34 |
| 3 | MO3 | 0,346 | 8,72 | 5,37 | 4,98 | 0,84 | 0,0442 | 12,79 |
| 3 | MO3 | 0,296 | 9,57 | 3,55 | 4,83 | 1,08 | 0,0302 | 10,20 |
| 3 | MO3 | 0,425 | 10,23 | 6,40 | 4,85 | 0,86 | 0,0437 | 10,28 |
| 3 | MO3 | 0,275 | 9,90 | 5,12 | 6,56 | 0,84 | 0,0295 | 10,74 |
| 3 | MO4 | 0,435 | 11,62 | 6,00 | 5,51 | 0,92 | 0,0466 | 10,72 |
| 3 | MO4 | 0,443 | 11,31 | 4,95 | 4,70 | 0,89 | 0,0411 | 9,28 |
| 3 | MO4 | 0,404 | 10,04 | 5,16 | 5,25 | 0,77 | 0,0312 | 7,72 |
| 3 | MO4 | 0,465 | 10,65 | 5,88 | 6,08 | 0,63 | 0,0243 | 5,23 |
| 3 | MO4 | 0,443 | 11,20 | 6,12 | 5,55 | 0,73 | 0,0271 | 6,12 |
| 3 | MO4 | 0,393 | 9,16 | 6,66 | 4,92 | 1,10 | 0,0401 | 10,21 |
| 3 | MO4 | 0,433 | 11,02 | 6,19 | 4,79 | 0,85 | 0,0400 | 9,24 |
| 3 | MO4 | 0,446 | 10,72 | 7,84 | 5,00 | 0,99 | 0,0317 | 7,10 |
| 3 | MO4 | 0,467 | 9,81 | 6,68 | 5,42 | 1,04 | 0,0395 | 8,45 |
| 3 | MO4 | 0,391 | 9,37 | 7,96 | 4,14 | 0,92 | 0,0249 | 6,37 |
| 3 | MO4 | 0,332 | 9,33 | 4,18 | 6,50 | 0,79 | 0,0429 | 12,93 |
| 3 | MO4 | 0,335 | 10,76 | 5,17 | 5,80 | 1,72 | 0,0450 | 13,45 |
| 3 | MO4 | 0,361 | 8,30 | 6,54 | 4,51 | 1,82 | 0,0427 | 11,84 |
| 3 | MO4 | 0,448 | 10,75 | 5,49 | 4,68 | 1,35 | 0,0320 | 7,14 |
| 3 | MO4 | 0,458 | 11,21 | 4,61 | 5,19 | 1,04 | 0,0423 | 9,24 |
| 3 | MO4 | 0,408 | 10,39 | 6,03 | 6,63 | 0,62 | 0,0320 | 7,84 |
| 3 | MO4 | 0,557 | 10,99 | 7,17 | 4,85 | 1,47 | 0,0353 | 6,33 |
| 3 | MO4 | 0,322 | 8,82 | 6,65 | 3,86 | 0,99 | 0,0242 | 7,51 |
| 3 | MO4 | 0,503 | 11,65 | 5,88 | 5,59 | 1,14 | 0,0448 | 8,91 |
| 3 | MO4 | 0,307 | 8,63 | 5,67 | 6,22 | 1,05 | 0,0298 | 9,70 |
| 3 | MO4 | 0,399 | 8,97 | 4,95 | 4,90 | 1,91 | 0,0447 | 11,20 |
| 3 | MO4 | 0,440 | 11,33 | 5,71 | 5,96 | 1,23 | 0,0490 | 11,13 |
| 3 | MO4 | 0,503 | 10,28 | 7,71 | 4,20 | 1,11 | 0,0345 | 6,86 |
| 3 | MO4 | 0,350 | 8,87 | 5,53 | 5,58 | 0,93 | 0,0316 | 9,04 |
| 3 | MO4 | 0,499 | 11,70 | 6,66 | 6,34 | 0,61 | 0,0420 | 8,42 |
| 3 | MO5 | 0,381 | 11,69 | 7,06 | 5,75 | 0,86 | 0,0261 | 6,85 |
| 3 | MO5 | 0,448 | 12,44 | 5,53 | 4,86 | 1,07 | 0,0279 | 6,23 |
| 3 | MO5 | 0,372 | 10,97 | 6,32 | 5,14 | 0,70 | 0,0196 | 5,26 |
| 3 | MO5 | 0,356 | 9,69 | 5,95 | 4,56 | 0,81 | 0,0344 | 9,67 |
| 3 | MO5 | 0,415 | 11,60 | 8,60 | 5,07 | 1,35 | 0,0224 | 5,40 |
| 3 | MO5 | 0,447 | 10,72 | 7,33 | 4,85 | 2,25 | 0,0394 | 8,82 |
| 3 | MO5 | 0,483 | 12,92 | 8,30 | 7,61 | 1,99 | 0,0309 | 6,40 |
| 3 | MO5 | 0,384 | 11,06 | 7,89 | 5,07 | 1,22 | 0,0213 | 5,55 |
| 3 | MO5 | 0,399 | 9,43 | 6,67 | 5,17 | 1,45 | 0,0213 | 5,35 |
| 3 | MO5 | 0,461 | 10,78 | 5,66 | 6,62 | 0,94 | 0,0282 | 6,12 |
| 3 | MO5 | 0,398 | 10,51 | 6,60 | 5,27 | 1,51 | 0,0344 | 8,64 |
| 3 | MO5 | 0,303 | 8,89 | 5,70 | 5,97 | 2,03 | 0,0309 | 10,19 |
| 3 | MO5 | 0,511 | 11,54 | 6,61 | 6,80 | 1,80 | 0,0388 | 7,59 |
| 3 | MO5 | 0,441 | 11,05 | 6,70 | 6,51 | 1,60 | 0,0288 | 6,53 |
| 3 | MO5 | 0,388 | 12,58 | 7,08 | 7,54 | 1,82 | 0,0291 | 7,49 |
| 3 | MO5 | 0,501 | 11,16 | 5,91 | 5,17 | 2,58 | 0,0354 | 7,06 |
| 3 | MO5 | 0,376 | 10,65 | 7,36 | 5,42 | 1,59 | 0,0242 | 6,44 |
| 3 | MO5 | 0,412 | 10,90 | 7,55 | 6,47 | 1,40 | 0,0305 | 7,40 |
| 3 | MO5 | 0,447 | 10,88 | 7,82 | 5,69 | 2,51 | 0,0250 | 5,59 |
| 3 | MO5 | 0,370 | 10,81 | 7,30 | 5,81 | 1,66 | 0,0270 | 7,31 |
| 3 | MO5 | 0,362 | 9,35 | 6,79 | 5,37 | 2,50 | 0,0310 | 8,57 |
| 3 | MO5 | 0,433 | 10,44 | 7,17 | 6,45 | 1,64 | 0,0317 | 7,32 |
| 3 | MO5 | 0,412 | 10,70 | 7,00 | 5,98 | 1,60 | 0,0249 | 6,04 |
| 3 | MO5 | 0,376 | 9,68 | 6,56 | 4,53 | 2,64 | 0,0201 | 5,34 |
| 3 | MO5 | 0,308 | 7,60 | 5,29 | 6,13 | 2,30 | 0,0239 | 7,75 |
| 3 | MO6 | 0,260 | 11,31 | 5,22 | 6,70 | 1,18 | 0,0243 | 9,33 |
| 3 | MO6 | 0,419 | 9,76 | 8,64 | 5,77 | 1,29 | 0,0209 | 4,99 |
| 3 | MO6 | 0,434 | 9,76 | 6,68 | 6,13 | 2,31 | 0,0252 | 5,80 |
| 3 | MO6 | 0,433 | 9,86 | 6,66 | 5,51 | 1,28 | 0,0417 | 9,64 |
| 3 | MO6 | 0,384 | 10,55 | 5,78 | 6,33 | 1,25 | 0,0294 | 7,66 |
| 3 | MO6 | 0,310 | 11,20 | 5,42 | 4,93 | 1,47 | 0,0279 | 9,00 |
| 3 | MO6 | 0,387 | 10,11 | 6,78 | 6,40 | 1,49 | 0,0197 | 5,09 |
| 3 | MO6 | 0,547 | 11,87 | 7,75 | 5,73 | 2,42 | 0,0242 | 4,42 |
| 3 | MO6 | 0,457 | 12,35 | 5,53 | 6,92 | 1,18 | 0,0246 | 5,38 |
| 3 | MO6 | 0,411 | 10,88 | 6,00 | 4,30 | 1,49 | 0,0181 | 4,40 |
| 3 | MO6 | 0,447 | 11,02 | 6,11 | 5,84 | 1,33 | 0,0274 | 6,13 |
| 3 | MO6 | 0,482 | 12,09 | 4,79 | 6,98 | 1,30 | 0,0356 | 7,39 |
| 3 | MO6 | 0,428 | 11,20 | 8,05 | 6,45 | 0,53 | 0,0200 | 4,68 |
| 3 | MO6 | 0,421 | 11,75 | 5,67 | 5,51 | 1,25 | 0,0207 | 4,92 |
| 3 | MO6 | 0,394 | 11,04 | 7,22 | 5,70 | 1,39 | 0,0382 | 9,69 |
| 3 | MO6 | 0,436 | 10,90 | 10,70 | 4,60 | 0,82 | 0,0416 | 9,53 |
| 3 | MO6 | 0,313 | 9,44 | 4,41 | 6,16 | 1,08 | 0,0417 | 13,33 |
| 3 | MO6 | 0,371 | 10,75 | 6,20 | 6,15 | 2,20 | 0,0300 | 8,08 |
| 3 | MO6 | 0,451 | 11,76 | 6,51 | 6,02 | 1,75 | 0,0298 | 6,61 |
| 3 | MO6 | 0,424 | 10,84 | 6,72 | 5,58 | 1,29 | 0,0205 | 4,83 |
| 3 | MO6 | 0,487 | 12,67 | 6,15 | 6,45 | 1,67 | 0,0341 | 7,01 |
| 3 | MO6 | 0,419 | 11,83 | 7,40 | 7,03 | 2,15 | 0,0319 | 7,61 |
| 3 | MO6 | 0,671 | 11,97 | 4,54 | 6,49 | 1,60 | 0,0343 | 5,11 |
| 3 | MO6 | 0,651 | 10,47 | 4,88 | 6,00 | 2,55 | 0,0363 | 5,58 |
| 3 | MO6 | 0,415 | 11,28 | 7,26 | 7,43 | 1,60 | 0,0253 | 6,10 |
| 3 | AB1 | 0,437 | 11,77 | 7,51 | 7,60 | 3,84 | 0,0523 | 11,97 |
| 3 | AB1 | 0,535 | 11,85 | 8,80 | 7,44 | 3,20 | 0,0406 | 7,59 |
| 3 | AB1 | 0,465 | 11,79 | 9,33 | 7,92 | 3,16 | 0,0497 | 10,69 |
| 3 | AB1 | 0,264 | 7,81 | 5,41 | 7,07 | 3,83 | 0,0505 | 19,13 |
| 3 | AB1 | 0,420 | 10,51 | 8,05 | 8,09 | 3,55 | 0,0408 | 9,71 |
| 3 | AB1 | 0,447 | 10,66 | 7,96 | 8,14 | 3,73 | 0,0460 | 10,29 |
| 3 | AB1 | 0,551 | 12,66 | 6,21 | 8,48 | 2,94 | 0,0443 | 8,04 |
| 3 | AB1 | 0,377 | 12,23 | 7,84 | 9,98 | 2,47 | 0,0797 | 21,14 |
| 3 | AB1 | 0,402 | 12,51 | 7,55 | 8,87 | 3,73 | 0,0645 | 16,04 |
| 3 | AB1 | 0,392 | 10,90 | 10,00 | 7,67 | 2,50 | 0,0613 | 15,64 |
| 3 | AB1 | 0,443 | 11,84 | 7,53 | 7,55 | 3,21 | 0,0564 | 12,73 |
| 3 | AB1 | 0,442 | 11,19 | 6,73 | 9,50 | 3,59 | 0,0842 | 19,05 |
| 3 | AB1 | 0,439 | 11,72 | 8,70 | 7,18 | 3,06 | 0,0509 | 11,59 |
| 3 | AB1 | 0,418 | 12,21 | 8,52 | 7,16 | 3,85 | 0,0628 | 15,02 |
| 3 | AB1 | 0,384 | 13,74 | 7,19 | 7,27 | 4,05 | 0,0611 | 15,91 |
| 3 | AB1 | 0,389 | 12,05 | 7,33 | 8,22 | 3,06 | 0,0640 | 16,45 |
| 3 | AB1 | 0,372 | 10,44 | 9,36 | 7,13 | 3,24 | 0,0387 | 10,40 |
| 3 | AB1 | 0,293 | 11,66 | 7,58 | 5,86 | 3,34 | 0,0503 | 17,17 |
| 3 | AB1 | 0,317 | 10,96 | 8,60 | 7,82 | 3,11 | 0,0658 | 20,76 |
| 3 | AB1 | 0,280 | 11,04 | 7,90 | 4,55 | 3,33 | 0,0554 | 19,79 |
| 3 | AB1 | 0,404 | 11,18 | 7,63 | 7,67 | 3,10 | 0,0584 | 14,46 |
| 3 | AB1 | 0,416 | 11,88 | 7,88 | 7,47 | 3,69 | 0,0588 | 14,13 |
| 3 | AB1 | 0,224 | 12,23 | 7,33 | 7,23 | 3,79 | 0,0438 | 19,55 |
| 3 | AB1 | 0,420 | 12,25 | 7,58 | 9,23 | 3,58 | 0,0731 | 17,40 |
| 3 | AB1 | 0,460 | 8,31 | 7,06 | 7,62 | 3,24 | 0,0501 | 10,89 |
| 3 | PB1 | 0,301 | 9,60 | 5,65 | 7,20 | 3,00 | 0,0458 | 15,22 |
| 3 | PB1 | 0,346 | 10,68 | 7,31 | 6,96 | 3,92 | 0,0485 | 14,02 |
| 3 | PB1 | 0,435 | 11,92 | 7,77 | 7,68 | 3,28 | 0,0496 | 11,40 |
| 3 | PB1 | 0,411 | 12,13 | 7,55 | 8,41 | 3,06 | 0,0546 | 13,28 |
| 3 | PB1 | 0,307 | 11,09 | 6,09 | 6,56 | 2,55 | 0,0434 | 14,14 |
| 3 | PB1 | 0,279 | 10,25 | 6,63 | 7,47 | 3,11 | 0,0507 | 18,17 |
| 3 | PB1 | 0,332 | 10,71 | 6,00 | 6,41 | 3,22 | 0,0409 | 12,32 |
| 3 | PB1 | 0,390 | 11,43 | 8,58 | 7,12 | 3,36 | 0,0397 | 10,18 |
| 3 | PB1 | 0,401 | 11,35 | 8,50 | 8,11 | 3,27 | 0,0755 | 18,83 |
| 3 | PB1 | 0,356 | 9,58 | 9,58 | 7,18 | 3,29 | 0,0481 | 13,51 |
| 3 | PB1 | 0,251 | 10,14 | 5,79 | 7,55 | 2,96 | 0,0484 | 19,28 |
| 3 | PB1 | 0,316 | 11,31 | 7,94 | 7,53 | 2,74 | 0,0480 | 15,19 |
| 3 | PB1 | 0,307 | 9,66 | 8,15 | 7,28 | 3,04 | 0,0394 | 12,83 |
| 3 | PB1 | 0,367 | 10,87 | 8,51 | 7,12 | 3,14 | 0,0552 | 15,04 |
| 3 | PB1 | 0,345 | 10,31 | 7,22 | 6,25 | 3,39 | 0,0543 | 15,74 |
| 3 | PB1 | 0,427 | 11,47 | 8,97 | 7,69 | 3,26 | 0,0560 | 13,11 |
| 3 | PB1 | 0,257 | 9,79 | 6,58 | 8,02 | 2,92 | 0,0402 | 15,64 |
| 3 | PB1 | 0,321 | 9,71 | 7,49 | 6,73 | 0,27 | 0,0577 | 17,98 |
| 3 | PB1 | 0,350 | 11,04 | 7,25 | 7,11 | 0,25 | 0,0461 | 13,17 |
| 3 | PB1 | 0,304 | 9,99 | 6,92 | 6,75 | 3,45 | 0,0476 | 15,66 |
| 3 | PB1 | 0,352 | 10,59 | 7,57 | 8,13 | 3,42 | 0,0517 | 14,69 |
| 3 | PB1 | 0,353 | 9,99 | 7,53 | 7,31 | 2,28 | 0,0460 | 13,03 |
| 3 | PB1 | 0,373 | 11,84 | 7,72 | 7,85 | 2,66 | 0,0618 | 16,57 |
| 3 | PB1 | 0,360 | 10,95 | 7,57 | 7,71 | 3,00 | 0,0479 | 13,31 |
| 3 | PB1 | 0,362 | 9,69 | 8,10 | 6,74 | 3,33 | 0,0480 | 13,26 |
| 3 | PTB1 | 0,561 | 9,44 | 4,44 | 6,11 | 2,36 | 0,0340 | 6,06 |
| 3 | PTB1 | 0,573 | 10,83 | 5,88 | 6,70 | 3,01 | 0,0410 | 7,16 |
| 3 | PTB1 | 0,621 | 12,50 | 6,29 | 5,85 | 1,46 | 0,0580 | 9,34 |
| 3 | PTB1 | 0,466 | 11,66 | 3,89 | 5,24 | 3,10 | 0,0420 | 9,01 |
| 3 | PTB1 | 0,467 | 9,94 | 5,82 | 8,63 | 2,02 | 0,0790 | 16,92 |
| 3 | PTB1 | 0,512 | 10,70 | 5,00 | 6,45 | 1,59 | 0,0440 | 8,59 |
| 3 | PTB1 | 0,514 | 12,39 | 5,64 | 8,52 | 1,99 | 0,0520 | 10,12 |
| 3 | PTB1 | 0,561 | 10,80 | 8,19 | 8,22 | 2,18 | 0,0770 | 13,73 |
| 3 | PTB1 | 0,645 | 12,64 | 7,05 | 6,86 | 1,55 | 0,0700 | 10,85 |
| 3 | PTB1 | 0,605 | 12,59 | 6,97 | 7,83 | 2,46 | 0,0920 | 15,21 |
| 3 | PTB1 | 0,662 | 12,15 | 8,79 | 7,80 | 3,20 | 0,0770 | 11,63 |
| 3 | PTB1 | 0,568 | 11,63 | 4,90 | 6,87 | 2,33 | 0,0560 | 9,86 |
| 3 | PTB1 | 0,370 | 9,08 | 4,05 | 6,94 | 2,43 | 0,0750 | 20,27 |
| 3 | PTB1 | 0,443 | 9,72 | 4,28 | 9,99 | 2,75 | 0,0460 | 10,38 |
| 3 | PTB1 | 0,443 | 9,74 | 5,25 | 6,68 | 2,08 | 0,0740 | 16,70 |
| 3 | PTB1 | 0,574 | 11,42 | 6,50 | 7,36 | 2,62 | 0,0580 | 10,10 |
| 3 | PTB1 | 0,491 | 10,82 | 4,13 | 8,96 | 3,05 | 0,0790 | 16,09 |
| 3 | PTB1 | 0,362 | 10,38 | 4,90 | 8,14 | 1,98 | 0,0750 | 20,72 |
| 3 | PTB1 | 0,472 | 10,66 | 5,43 | 9,12 | 2,04 | 0,0560 | 11,86 |
| 3 | PTB1 | 0,574 | 12,11 | 8,23 | 6,31 | 1,76 | 0,0490 | 8,54 |
| 3 | PTB1 | 0,427 | 11,64 | 4,56 | 6,71 | 1,59 | 0,0420 | 9,84 |
| 3 | PTB1 | 0,612 | 11,07 | 5,98 | 8,75 | 2,11 | 0,0510 | 8,33 |
| 3 | PTB1 | 0,546 | 11,72 | 5,14 | 7,64 | 2,27 | 0,0350 | 6,41 |
| 3 | PTB1 | 0,522 | 10,17 | 6,16 | 7,39 | 2,40 | 0,0730 | 13,98 |
| 3 | PTB1 | 0,350 | 9,73 | 4,31 | 9,31 | 3,07 | 0,0660 | 18,86 |
| 3 | SFB1 | 0,743 | 12,77 | 5,36 | 7,28 | 0,78 | 0,0479 | 6,45 |
| 3 | SFB1 | 0,576 | 11,47 | 5,52 | 5,89 | 0,69 | 0,0452 | 7,85 |
| 3 | SFB1 | 0,588 | 11,30 | 7,23 | 5,13 | 0,94 | 0,0405 | 6,89 |
| 3 | SFB1 | 0,675 | 10,99 | 5,89 | 5,35 | 0,77 | 0,0480 | 7,11 |
| 3 | SFB1 | 0,585 | 9,52 | 6,51 | 4,59 | 0,72 | 0,0305 | 5,21 |
| 3 | SFB1 | 0,676 | 10,75 | 6,65 | 4,99 | 0,84 | 0,0468 | 6,92 |
| 3 | SFB1 | 0,620 | 11,60 | 7,01 | 5,37 | 1,06 | 0,0412 | 6,65 |
| 3 | SFB1 | 0,587 | 9,50 | 6,30 | 4,07 | 0,78 | 0,0245 | 4,17 |
| 3 | SFB1 | 0,632 | 9,83 | 8,00 | 4,07 | 0,71 | 0,0567 | 8,97 |
| 3 | SFB1 | 0,563 | 11,07 | 4,28 | 5,31 | 0,63 | 0,0578 | 10,27 |
| 3 | SFB1 | 0,576 | 10,07 | 5,94 | 4,83 | 1,09 | 0,0338 | 5,87 |
| 3 | SFB1 | 0,650 | 10,30 | 5,81 | 5,19 | 0,91 | 0,0506 | 7,78 |
| 3 | SFB1 | 0,602 | 9,50 | 5,83 | 4,60 | 0,65 | 0,0341 | 5,66 |
| 3 | SFB1 | 0,578 | 12,35 | 5,30 | 6,25 | 0,86 | 0,0319 | 5,52 |
| 3 | SFB1 | 0,442 | 11,69 | 5,82 | 5,42 | 0,72 | 0,0334 | 7,56 |
| 3 | SFB1 | 0,526 | 10,71 | 6,51 | 6,01 | 0,98 | 0,0293 | 5,57 |
| 3 | SFB1 | 0,613 | 8,80 | 7,01 | 4,70 | 0,93 | 0,0443 | 7,23 |
| 3 | SFB1 | 0,582 | 9,62 | 5,41 | 5,30 | 0,84 | 0,0453 | 7,78 |
| 3 | SFB1 | 0,500 | 10,48 | 4,63 | 5,46 | 0,75 | 0,0338 | 6,76 |
| 3 | SFB1 | 0,662 | 12,20 | 6,51 | 4,56 | 0,71 | 0,0375 | 5,66 |
| 3 | SFB1 | 0,572 | 10,56 | 6,53 | 5,39 | 0,91 | 0,0375 | 6,56 |
| 3 | SFB1 | 0,387 | 11,63 | 2,43 | 5,33 | 0,75 | 0,0471 | 12,17 |
| 3 | SFB1 | 0,632 | 12,06 | 5,07 | 6,01 | 0,70 | 0,0395 | 6,25 |
| 3 | SFB1 | 0,420 | 8,90 | 5,96 | 4,48 | 0,62 | 0,0287 | 6,83 |
| 3 | SFB1 | 0,444 | 9,18 | 6,37 | 5,53 | 0,86 | 0,0505 | 11,37 |
| 3 | PTO2 | 0,716 | 15,32 | 10,55 | 7,37 | 1,93 | 0,0471 | 6,58 |
| 3 | PTO2 | 0,694 | 14,40 | 6,73 | 7,89 | 1,86 | 0,0671 | 9,67 |
| 3 | PTO2 | 0,695 | 14,40 | 6,89 | 8,38 | 1,50 | 0,0394 | 5,67 |
| 3 | PTO2 | 0,695 | 15,22 | 8,44 | 8,41 | 1,52 | 0,0344 | 4,95 |
| 3 | PTO2 | 0,595 | 14,36 | 4,30 | 8,53 | 0,81 | 0,0349 | 5,87 |
| 3 | PTO2 | 0,668 | 13,98 | 7,14 | 8,16 | 1,68 | 0,0342 | 5,12 |
| 3 | PTO2 | 0,668 | 14,48 | 7,74 | 7,66 | 1,60 | 0,0353 | 5,28 |
| 3 | PTO2 | 0,703 | 12,66 | 7,17 | 5,86 | 1,79 | 0,0349 | 4,96 |
| 3 | PTO2 | 0,339 | 11,77 | 5,08 | 6,46 | 0,99 | 0,0129 | 3,81 |
| 3 | PTO2 | 0,642 | 13,10 | 5,39 | 6,80 | 2,70 | 0,0494 | 7,69 |
| 3 | PTO2 | 0,570 | 13,49 | 7,92 | 7,38 | 1,85 | 0,0362 | 6,35 |
| 3 | PTO2 | 0,500 | 11,61 | 6,54 | 6,24 | 2,36 | 0,0293 | 5,86 |
| 3 | PTO2 | 0,573 | 13,66 | 8,38 | 8,88 | 2,26 | 0,0431 | 7,52 |
| 3 | PTO2 | 0,603 | 13,88 | 8,02 | 7,25 | 2,02 | 0,0336 | 5,57 |
| 3 | PTO2 | 0,438 | 11,96 | 6,25 | 7,37 | 1,78 | 0,0182 | 4,16 |
| 3 | PTO2 | 0,619 | 12,64 | 6,49 | 7,80 | 1,16 | 0,0369 | 5,96 |
| 3 | PTO2 | 0,585 | 13,92 | 8,96 | 7,86 | 1,84 | 0,0244 | 4,17 |
| 3 | PTO2 | 0,742 | 13,20 | 7,65 | 8,32 | 2,10 | 0,0343 | 4,62 |
| 3 | PTO2 | 0,614 | 12,38 | 6,30 | 6,78 | 2,02 | 0,0255 | 4,15 |
| 3 | PTO2 | 0,494 | 11,55 | 8,44 | 5,40 | 1,15 | 0,0316 | 6,40 |
| 3 | PTO2 | 0,675 | 14,53 | 7,26 | 6,70 | 2,24 | 0,0386 | 5,72 |
| 3 | PTO2 | 0,670 | 14,79 | 7,33 | 8,14 | 1,80 | 0,0420 | 6,27 |
| 3 | PTO2 | 0,641 | 13,10 | 7,55 | 6,64 | 1,70 | 0,0233 | 3,63 |
| 3 | PTO2 | 0,458 | 10,85 | 6,61 | 8,08 | 1,29 | 0,0202 | 4,41 |
| 3 | PTO2 | 0,448 | 12,58 | 7,49 | 8,38 | 1,28 | 0,0232 | 5,18 |
| 3 | PTO3 | 0,638 | 13,30 | 7,66 | 7,18 | 3,51 | 0,0550 | 8,62 |
| 3 | PTO3 | 0,785 | 14,35 | 8,35 | 7,74 | 0,91 | 0,0470 | 5,99 |
| 3 | PTO3 | 0,665 | 13,18 | 6,75 | 7,52 | 1,61 | 0,0480 | 7,22 |
| 3 | PTO3 | 0,584 | 12,64 | 7,80 | 6,80 | 1,10 | 0,0320 | 5,48 |
| 3 | PTO3 | 0,516 | 13,30 | 8,71 | 9,60 | 1,26 | 0,0560 | 10,85 |
| 3 | PTO3 | 0,753 | 14,30 | 7,92 | 6,55 | 1,19 | 0,0440 | 5,84 |
| 3 | PTO3 | 0,730 | 13,49 | 7,56 | 7,92 | 1,29 | 0,0420 | 5,75 |
| 3 | PTO3 | 0,656 | 14,88 | 7,06 | 8,20 | 1,28 | 0,0420 | 6,40 |
| 3 | PTO3 | 0,631 | 13,47 | 9,00 | 5,96 | 1,81 | 0,0360 | 5,71 |
| 3 | PTO3 | 0,619 | 13,11 | 8,66 | 7,36 | 0,98 | 0,0390 | 6,30 |
| 3 | PTO3 | 0,674 | 13,29 | 8,01 | 6,91 | 1,42 | 0,0370 | 5,49 |
| 3 | PTO3 | 0,603 | 13,60 | 8,83 | 5,82 | 1,20 | 0,0340 | 5,64 |
| 3 | PTO3 | 0,666 | 12,85 | 6,31 | 6,56 | 2,06 | 0,0600 | 9,01 |
| 3 | PTO3 | 0,713 | 13,60 | 7,41 | 6,99 | 2,55 | 0,0450 | 6,31 |
| 3 | PTO3 | 0,518 | 11,69 | 7,34 | 8,34 | 1,32 | 0,0560 | 10,81 |
| 3 | PTO3 | 0,575 | 11,00 | 7,31 | 5,86 | 1,95 | 0,0510 | 8,87 |
| 3 | PTO3 | 0,755 | 13,03 | 8,46 | 5,70 | 2,09 | 0,0620 | 8,21 |
| 3 | PTO3 | 0,427 | 11,40 | 6,58 | 7,18 | 1,87 | 0,0260 | 6,09 |
| 3 | PTO3 | 0,425 | 12,33 | 5,68 | 7,34 | 2,10 | 0,0480 | 11,29 |
| 3 | PTO3 | 0,713 | 13,25 | 9,26 | 7,70 | 2,73 | 0,0410 | 5,75 |
| 3 | PTO3 | 0,443 | 12,05 | 5,70 | 6,83 | 2,30 | 0,0370 | 8,35 |
| 3 | PTO3 | 0,735 | 14,47 | 8,85 | 7,60 | 2,96 | 0,0430 | 5,85 |
| 3 | PTO3 | 0,571 | 12,96 | 7,13 | 6,87 | 2,01 | 0,0450 | 7,88 |
| 3 | PTO3 | 0,568 | 11,71 | 6,36 | 6,13 | 1,87 | 0,0330 | 5,81 |
| 3 | PTO3 | 0,569 | 13,01 | 8,64 | 9,85 | 1,66 | 0,0410 | 7,21 |
| 3 | PTO6 | 0,713 | 11,60 | 8,64 | 5,83 | 0,88 | 0,0264 | 3,70 |
| 3 | PTO6 | 0,606 | 12,39 | 6,41 | 5,84 | 1,32 | 0,0457 | 7,55 |
| 3 | PTO6 | 0,583 | 11,02 | 6,21 | 6,94 | 1,45 | 0,0477 | 8,18 |
| 3 | PTO6 | 0,523 | 13,58 | 4,65 | 6,61 | 0,66 | 0,0469 | 8,98 |
| 3 | PTO6 | 0,673 | 11,28 | 7,26 | 5,69 | 0,91 | 0,0466 | 6,93 |
| 3 | PTO6 | 0,682 | 11,73 | 6,93 | 5,70 | 2,13 | 0,0452 | 6,63 |
| 3 | PTO6 | 0,536 | 11,06 | 6,09 | 4,90 | 0,94 | 0,0431 | 8,05 |
| 3 | PTO6 | 0,683 | 12,82 | 7,26 | 6,11 | 0,72 | 0,0385 | 5,64 |
| 3 | PTO6 | 0,589 | 12,34 | 5,90 | 5,55 | 1,41 | 0,0316 | 5,36 |
| 3 | PTO6 | 0,728 | 11,00 | 6,53 | 5,41 | 0,66 | 0,0430 | 5,91 |
| 3 | PTO6 | 0,616 | 12,73 | 5,50 | 6,31 | 0,58 | 0,0454 | 7,37 |
| 3 | PTO6 | 0,437 | 10,74 | 4,30 | 5,80 | 0,76 | 0,0545 | 12,47 |
| 3 | PTO6 | 0,556 | 11,34 | 7,07 | 7,00 | 0,66 | 0,0512 | 9,21 |
| 3 | PTO6 | 0,708 | 14,50 | 4,73 | 6,40 | 0,70 | 0,0372 | 5,25 |
| 3 | PTO6 | 0,704 | 10,65 | 5,36 | 5,83 | 0,65 | 0,0532 | 7,55 |
| 3 | PTO6 | 0,609 | 13,35 | 5,32 | 6,50 | 0,61 | 0,0382 | 6,28 |
| 3 | PTO6 | 0,626 | 11,84 | 6,14 | 5,82 | 1,31 | 0,0559 | 8,93 |
| 3 | PTO6 | 0,650 | 11,50 | 6,03 | 7,76 | 0,55 | 0,0434 | 6,68 |
| 3 | PTO6 | 0,619 | 10,06 | 5,98 | 5,71 | 0,70 | 0,0319 | 5,15 |
| 3 | PTO6 | 0,550 | 12,44 | 6,39 | 5,69 | 0,78 | 0,0318 | 5,78 |
| 3 | PTO6 | 0,649 | 12,00 | 5,24 | 5,68 | 0,85 | 0,0561 | 8,65 |
| 3 | PTO6 | 0,626 | 11,80 | 5,67 | 8,85 | 1,15 | 0,0620 | 9,91 |
| 3 | PTO6 | 0,623 | 11,35 | 7,80 | 6,50 | 0,64 | 0,0444 | 7,13 |
| 3 | PTO6 | 0,597 | 13,36 | 5,76 | 6,11 | 0,81 | 0,0363 | 6,08 |
| 3 | PTO6 | 0,589 | 11,40 | 7,25 | 5,48 | 0,56 | 0,0424 | 7,20 |
| 3 | PDO1 | 0,535 | 13,61 | 7,61 | 6,25 | 2,51 | 0,0381 | 7,12 |
| 3 | PDO1 | 0,638 | 11,36 | 8,33 | 7,68 | 3,37 | 0,0478 | 7,49 |
| 3 | PDO1 | 0,634 | 13,01 | 6,77 | 8,31 | 2,16 | 0,0652 | 10,28 |
| 3 | PDO1 | 0,463 | 10,67 | 6,79 | 7,19 | 2,74 | 0,0503 | 10,86 |
| 3 | PDO1 | 0,679 | 13,07 | 8,27 | 7,93 | 2,29 | 0,0704 | 10,37 |
| 3 | PDO1 | 0,490 | 10,24 | 7,97 | 7,02 | 2,52 | 0,0429 | 8,76 |
| 3 | PDO1 | 0,796 | 13,28 | 8,17 | 5,78 | 2,29 | 0,0269 | 3,38 |
| 3 | PDO1 | 0,400 | 12,47 | 6,52 | 6,78 | 2,96 | 0,0509 | 12,73 |
| 3 | PDO1 | 0,513 | 11,94 | 5,58 | 7,46 | 1,94 | 0,0542 | 10,57 |
| 3 | PDO1 | 0,587 | 9,84 | 7,47 | 7,42 | 1,75 | 0,0363 | 6,18 |
| 3 | PDO1 | 0,272 | 9,96 | 5,91 | 6,55 | 1,69 | 0,0307 | 11,29 |
| 3 | PDO1 | 0,649 | 11,88 | 6,54 | 6,00 | 3,07 | 0,0467 | 7,20 |
| 3 | PDO1 | 0,597 | 13,01 | 7,66 | 6,48 | 2,33 | 0,0340 | 5,70 |
| 3 | PDO1 | 0,569 | 15,40 | 7,34 | 7,16 | 2,42 | 0,0571 | 10,04 |
| 3 | PDO1 | 0,719 | 14,60 | 6,19 | 7,90 | 1,70 | 0,0370 | 5,15 |
| 3 | PDO1 | 0,784 | 13,38 | 6,81 | 5,68 | 2,23 | 0,0340 | 4,34 |
| 3 | PDO1 | 0,240 | 14,66 | 7,88 | 6,86 | 3,25 | 0,0368 | 15,33 |
| 3 | PDO1 | 0,619 | 10,78 | 4,14 | 6,13 | 1,53 | 0,0332 | 5,36 |
| 3 | PDO1 | 0,633 | 12,84 | 5,89 | 8,15 | 1,70 | 0,0863 | 13,63 |
| 3 | PDO1 | 0,489 | 13,42 | 6,60 | 7,22 | 1,87 | 0,0381 | 7,79 |
| 3 | PDO1 | 0,545 | 12,79 | 6,54 | 8,25 | 1,65 | 0,0468 | 8,59 |
| 3 | PDO1 | 0,603 | 14,01 | 7,76 | 7,31 | 1,91 | 0,0381 | 6,32 |
| 3 | PDO1 | 0,285 | 11,93 | 7,77 | 6,06 | 3,00 | 0,0296 | 10,39 |
| 3 | PDO1 | 0,676 | 10,16 | 5,81 | 5,77 | 2,00 | 0,0355 | 5,25 |
| 3 | PDO1 | 0,715 | 14,53 | 6,82 | 5,83 | 1,96 | 0,0341 | 4,77 |
| 3 | PDO2 | 0,368 | 11,33 | 7,23 | 7,35 | 3,00 | 0,0398 | 10,82 |
| 3 | PDO2 | 0,465 | 12,24 | 7,71 | 8,63 | 2,97 | 0,0517 | 11,12 |
| 3 | PDO2 | 0,412 | 10,19 | 8,38 | 6,67 | 3,72 | 0,0564 | 13,69 |
| 3 | PDO2 | 0,385 | 10,80 | 6,90 | 7,35 | 3,01 | 0,0412 | 10,70 |
| 3 | PDO2 | 0,327 | 9,43 | 6,50 | 6,85 | 2,41 | 0,0343 | 10,49 |
| 3 | PDO2 | 0,479 | 10,52 | 9,00 | 7,60 | 3,06 | 0,0661 | 13,80 |
| 3 | PDO2 | 0,455 | 11,90 | 8,38 | 7,84 | 3,66 | 0,0540 | 11,87 |
| 3 | PDO2 | 0,474 | 11,98 | 8,97 | 8,30 | 3,33 | 0,0560 | 11,81 |
| 3 | PDO2 | 0,439 | 10,92 | 9,52 | 6,54 | 3,75 | 0,0533 | 12,14 |
| 3 | PDO2 | 0,383 | 11,05 | 8,56 | 7,66 | 3,73 | 0,0628 | 16,40 |
| 3 | PDO2 | 0,363 | 10,70 | 8,01 | 8,16 | 3,05 | 0,0587 | 16,17 |
| 3 | PDO2 | 0,401 | 10,46 | 8,62 | 7,11 | 2,91 | 0,0559 | 13,94 |
| 3 | PDO2 | 0,374 | 10,39 | 7,99 | 7,21 | 3,56 | 0,0442 | 11,82 |
| 3 | PDO2 | 0,364 | 11,20 | 7,78 | 8,64 | 3,46 | 0,0701 | 19,26 |
| 3 | PDO2 | 0,432 | 11,00 | 8,34 | 6,62 | 3,30 | 0,0596 | 13,80 |
| 3 | PDO2 | 0,446 | 12,00 | 9,88 | 8,03 | 2,71 | 0,0667 | 14,96 |
| 3 | PDO2 | 0,477 | 11,07 | 9,34 | 6,93 | 3,89 | 0,0560 | 11,74 |
| 3 | PDO2 | 0,347 | 11,33 | 8,15 | 7,49 | 3,02 | 0,0417 | 12,02 |
| 3 | PDO2 | 0,409 | 11,41 | 7,62 | 7,50 | 3,18 | 0,0534 | 13,06 |
| 3 | PDO2 | 0,358 | 10,64 | 7,83 | 6,80 | 3,08 | 0,0396 | 11,06 |
| 3 | PDO2 | 0,431 | 12,77 | 9,23 | 8,02 | 2,50 | 0,0510 | 11,83 |
| 3 | PDO2 | 0,384 | 10,90 | 7,93 | 7,62 | 3,31 | 0,0624 | 16,25 |
| 3 | PDO2 | 0,301 | 12,73 | 5,86 | 8,65 | 2,61 | 0,0428 | 14,22 |
| 3 | PDO2 | 0,309 | 10,04 | 7,27 | 6,38 | 3,03 | 0,0480 | 15,53 |
| 3 | PDO2 | 0,370 | 9,30 | 7,90 | 7,37 | 3,72 | 0,0554 | 14,97 |
| 3 | CRO1 | 0,568 | 14,21 | 7,86 | 10,62 | 1,69 | 0,0712 | 12,54 |
| 3 | CRO1 | 0,606 | 13,94 | 6,40 | 8,54 | 1,88 | 0,0431 | 7,11 |
| 3 | CRO1 | 0,478 | 14,11 | 5,28 | 9,61 | 2,08 | 0,0302 | 6,32 |
| 3 | CRO1 | 0,832 | 13,62 | 8,31 | 7,50 | 2,08 | 0,0728 | 8,75 |
| 3 | CRO1 | 0,608 | 13,76 | 8,89 | 6,29 | 2,23 | 0,0512 | 8,42 |
| 3 | CRO1 | 0,847 | 13,66 | 8,77 | 9,03 | 2,66 | 0,0116 | 1,37 |
| 3 | CRO1 | 0,755 | 15,56 | 7,14 | 9,90 | 2,70 | 0,0770 | 10,20 |
| 3 | CRO1 | 0,660 | 12,94 | 8,34 | 7,35 | 2,35 | 0,0534 | 8,09 |
| 3 | CRO1 | 0,709 | 14,19 | 7,31 | 10,51 | 2,13 | 0,0903 | 12,74 |
| 3 | CRO1 | 0,596 | 11,41 | 7,60 | 7,32 | 2,06 | 0,0445 | 7,47 |
| 3 | CRO1 | 0,699 | 12,87 | 7,57 | 7,62 | 2,20 | 0,0582 | 8,33 |
| 3 | CRO1 | 0,657 | 13,90 | 6,03 | 8,64 | 1,79 | 0,0753 | 11,46 |
| 3 | CRO1 | 0,666 | 12,02 | 9,41 | 8,03 | 2,93 | 0,0708 | 10,63 |
| 3 | CRO1 | 0,676 | 13,56 | 6,95 | 8,55 | 1,76 | 0,0670 | 9,91 |
| 3 | CRO1 | 0,590 | 13,57 | 6,43 | 8,73 | 2,06 | 0,0600 | 10,17 |
| 3 | CRO1 | 0,649 | 12,28 | 6,30 | 6,79 | 2,07 | 0,0553 | 8,52 |
| 3 | CRO1 | 0,663 | 11,20 | 8,39 | 7,64 | 3,07 | 0,0541 | 8,16 |
| 3 | CRO1 | 0,607 | 11,65 | 6,40 | 7,20 | 1,80 | 0,0388 | 6,39 |
| 3 | CRO1 | 0,718 | 12,95 | 5,80 | 8,62 | 2,00 | 0,0428 | 5,96 |
| 3 | CRO1 | 0,560 | 12,46 | 5,57 | 8,12 | 2,37 | 0,0552 | 9,86 |
| 3 | CRO1 | 0,679 | 13,36 | 6,99 | 6,35 | 2,15 | 0,0390 | 5,74 |
| 3 | CRO1 | 0,666 | 12,76 | 8,55 | 7,72 | 2,60 | 0,0632 | 9,49 |
| 3 | CRO1 | 0,749 | 13,47 | 7,74 | 7,62 | 2,18 | 0,0723 | 9,65 |
| 3 | CRO1 | 0,708 | 13,93 | 7,34 | 9,17 | 1,93 | 0,0644 | 9,10 |
| 3 | CRO1 | 0,637 | 13,45 | 7,05 | 8,81 | 1,07 | 0,0554 | 8,70 |
| 3 | CRO2 | 0,763 | 12,72 | 8,67 | 6,31 | 2,72 | 0,0554 | 7,26 |
| 3 | CRO2 | 0,671 | 11,19 | 7,86 | 6,15 | 2,79 | 0,0333 | 4,96 |
| 3 | CRO2 | 0,660 | 13,40 | 9,21 | 6,19 | 2,52 | 0,0385 | 5,83 |
| 3 | CRO2 | 0,649 | 12,24 | 8,19 | 6,32 | 2,42 | 0,0369 | 5,69 |
| 3 | CRO2 | 0,662 | 12,37 | 10,22 | 5,56 | 2,56 | 0,0291 | 4,40 |
| 3 | CRO2 | 0,688 | 10,91 | 8,68 | 7,20 | 2,62 | 0,0685 | 9,96 |
| 3 | CRO2 | 0,724 | 12,89 | 9,10 | 7,26 | 2,05 | 0,0466 | 6,44 |
| 3 | CRO2 | 0,528 | 9,15 | 6,03 | 7,41 | 2,35 | 0,0776 | 14,70 |
| 3 | CRO2 | 0,741 | 12,11 | 8,44 | 9,00 | 1,82 | 0,0475 | 6,41 |
| 3 | CRO2 | 0,712 | 13,65 | 8,48 | 7,20 | 2,98 | 0,0641 | 9,00 |
| 3 | CRO2 | 0,791 | 13,03 | 8,82 | 8,23 | 2,45 | 0,0395 | 4,99 |
| 3 | CRO2 | 0,618 | 11,54 | 7,50 | 7,65 | 1,77 | 0,0314 | 5,08 |
| 3 | CRO2 | 0,540 | 11,93 | 9,18 | 7,82 | 1,97 | 0,0495 | 9,17 |
| 3 | CRO2 | 0,723 | 13,27 | 9,72 | 6,74 | 1,66 | 0,0533 | 7,37 |
| 3 | CRO2 | 0,435 | 11,05 | 4,98 | 7,63 | 3,13 | 0,0619 | 14,23 |
| 3 | CRO2 | 0,552 | 10,04 | 7,58 | 7,90 | 2,42 | 0,0526 | 9,53 |
| 3 | CRO2 | 0,779 | 13,40 | 8,08 | 6,75 | 2,46 | 0,0372 | 4,78 |
| 3 | CRO2 | 0,720 | 12,78 | 8,62 | 6,80 | 3,32 | 0,0478 | 6,64 |
| 3 | CRO2 | 0,635 | 12,31 | 7,54 | 8,68 | 2,04 | 0,0652 | 10,27 |
| 3 | CRO2 | 0,691 | 12,66 | 7,69 | 7,92 | 2,09 | 0,0541 | 7,83 |
| 3 | CRO2 | 0,652 | 11,93 | 8,42 | 5,29 | 2,69 | 0,0455 | 6,98 |
| 3 | CRO2 | 0,754 | 12,36 | 8,07 | 7,85 | 2,59 | 0,0381 | 5,05 |
| 3 | CRO2 | 0,572 | 11,72 | 5,40 | 7,50 | 3,12 | 0,0341 | 5,96 |
| 3 | CRO2 | 0,723 | 14,00 | 8,79 | 7,23 | 2,37 | 0,0485 | 6,71 |
| 3 | CRO2 | 0,741 | 13,78 | 8,77 | 6,92 | 2,61 | 0,0498 | 6,72 |
| 3 | ADO4 | 0,729 | 12,81 | 9,72 | 7,54 | 3,28 | 0,0685 | 9,40 |
| 3 | ADO4 | 0,429 | 13,54 | 7,11 | 8,77 | 2,79 | 0,0541 | 12,61 |
| 3 | ADO4 | 0,626 | 14,41 | 7,60 | 7,92 | 1,85 | 0,0332 | 5,30 |
| 3 | ADO4 | 0,709 | 13,18 | 7,86 | 6,92 | 2,56 | 0,0613 | 8,65 |
| 3 | ADO4 | 0,577 | 13,26 | 7,14 | 8,64 | 2,68 | 0,0508 | 8,80 |
| 3 | ADO4 | 0,426 | 12,83 | 9,00 | 8,00 | 1,95 | 0,0326 | 7,65 |
| 3 | ADO4 | 0,538 | 11,63 | 9,76 | 6,12 | 2,16 | 0,0330 | 6,13 |
| 3 | ADO4 | 0,604 | 13,65 | 8,00 | 7,31 | 2,09 | 0,0461 | 7,63 |
| 3 | ADO4 | 0,445 | 13,39 | 7,09 | 7,83 | 2,14 | 0,0471 | 10,58 |
| 3 | ADO4 | 0,532 | 13,66 | 8,32 | 6,51 | 1,32 | 0,0428 | 8,05 |
| 3 | ADO4 | 0,430 | 13,84 | 7,95 | 6,56 | 1,30 | 0,0410 | 9,53 |
| 3 | ADO4 | 0,480 | 13,27 | 7,02 | 8,00 | 1,28 | 0,0541 | 11,27 |
| 3 | ADO4 | 0,400 | 12,17 | 9,07 | 7,50 | 2,30 | 0,0657 | 16,43 |
| 3 | ADO4 | 0,326 | 9,65 | 7,39 | 6,83 | 1,72 | 0,0327 | 10,03 |
| 3 | ADO4 | 0,576 | 13,61 | 6,43 | 6,35 | 1,61 | 0,0494 | 8,58 |
| 3 | ADO4 | 0,600 | 13,60 | 8,10 | 6,98 | 1,30 | 0,0379 | 6,32 |
| 3 | ADO4 | 0,505 | 12,90 | 7,31 | 6,80 | 1,21 | 0,0362 | 7,17 |
| 3 | ADO4 | 0,558 | 13,18 | 9,07 | 7,00 | 0,94 | 0,0400 | 7,17 |
| 3 | ADO4 | 0,663 | 11,89 | 7,12 | 7,40 | 1,92 | 0,0520 | 7,84 |
| 3 | ADO4 | 0,696 | 14,65 | 7,82 | 5,05 | 1,26 | 0,0260 | 3,74 |
| 3 | ADO4 | 0,441 | 11,77 | 7,94 | 6,45 | 1,66 | 0,0430 | 9,75 |
| 3 | ADO4 | 0,572 | 13,74 | 8,00 | 6,50 | 1,55 | 0,0415 | 7,26 |
| 3 | ADO4 | 0,379 | 12,41 | 8,00 | 7,59 | 1,28 | 0,0567 | 14,96 |
| 3 | ADO4 | 0,449 | 12,38 | 6,14 | 5,51 | 1,24 | 0,0430 | 9,58 |
| 3 | ADO4 | 0,603 | 12,30 | 8,10 | 6,35 | 2,18 | 0,0397 | 6,58 |
| 3 | BO1 | 0,257 | 9,70 | 3,40 | 5,44 | 0,81 | 0,0316 | 12,29 |
| 3 | BO1 | 0,422 | 10,03 | 5,47 | 5,74 | 0,92 | 0,0463 | 10,98 |
| 3 | BO1 | 0,310 | 9,50 | 5,08 | 5,17 | 0,88 | 0,0582 | 18,80 |
| 3 | BO1 | 0,474 | 9,64 | 6,10 | 5,83 | 2,14 | 0,0521 | 11,00 |
| 3 | BO1 | 0,423 | 9,14 | 6,37 | 5,35 | 0,75 | 0,0385 | 9,11 |
| 3 | BO1 | 0,399 | 9,87 | 4,79 | 5,25 | 0,96 | 0,0379 | 9,51 |
| 3 | BO1 | 0,511 | 8,64 | 6,60 | 4,95 | 1,66 | 0,0524 | 10,26 |
| 3 | BO1 | 0,306 | 7,27 | 4,49 | 5,77 | 0,77 | 0,0336 | 10,99 |
| 3 | BO1 | 0,272 | 7,97 | 4,50 | 5,52 | 0,95 | 0,0431 | 15,82 |
| 3 | BO1 | 0,314 | 9,95 | 4,08 | 5,87 | 1,29 | 0,0435 | 13,88 |
| 3 | BO1 | 0,520 | 11,63 | 5,16 | 5,00 | 0,80 | 0,0331 | 6,37 |
| 3 | BO1 | 0,500 | 9,52 | 6,47 | 4,75 | 1,35 | 0,0425 | 8,49 |
| 3 | BO1 | 0,351 | 8,30 | 4,78 | 5,29 | 1,37 | 0,0353 | 10,05 |
| 3 | BO1 | 0,322 | 8,20 | 4,48 | 6,83 | 1,35 | 0,0484 | 15,05 |
| 3 | BO1 | 0,471 | 9,80 | 4,29 | 6,01 | 1,31 | 0,0584 | 12,40 |
| 3 | BO1 | 0,547 | 11,90 | 7,13 | 5,99 | 1,52 | 0,0452 | 8,26 |
| 3 | BO1 | 0,439 | 10,37 | 5,44 | 5,87 | 0,72 | 0,0333 | 7,59 |
| 3 | BO1 | 0,238 | 9,30 | 2,48 | 6,48 | 1,15 | 0,0469 | 19,71 |
| 3 | BO1 | 0,450 | 9,86 | 4,93 | 5,39 | 1,63 | 0,0384 | 8,53 |
| 3 | BO1 | 0,224 | 8,63 | 3,14 | 6,38 | 1,47 | 0,0358 | 15,96 |
| 3 | BO1 | 0,416 | 9,36 | 4,13 | 5,01 | 1,60 | 0,0435 | 10,45 |
| 3 | BO1 | 0,452 | 8,75 | 5,51 | 5,06 | 0,67 | 0,0403 | 8,92 |
| 3 | BO1 | 0,363 | 8,95 | 3,85 | 4,98 | 0,75 | 0,0476 | 13,12 |
| 3 | BO1 | 0,472 | 10,67 | 5,47 | 5,04 | 0,90 | 0,0459 | 9,73 |
| 3 | BO1 | 0,426 | 10,10 | 4,30 | 6,50 | 1,02 | 0,0315 | 7,40 |
| 3 | ADO2 | 0,619 | 15,46 | 7,36 | 9,90 | 1,50 | 0,0392 | 6,33 |
| 3 | ADO2 | 0,661 | 13,26 | 6,04 | 7,91 | 1,98 | 0,0527 | 7,97 |
| 3 | ADO2 | 0,650 | 14,39 | 8,27 | 6,72 | 3,00 | 0,0520 | 8,00 |
| 3 | ADO2 | 0,637 | 14,24 | 6,62 | 9,00 | 1,90 | 0,0454 | 7,13 |
| 3 | ADO2 | 0,591 | 15,84 | 7,27 | 9,99 | 1,63 | 0,0467 | 7,90 |
| 3 | ADO2 | 0,645 | 15,58 | 8,76 | 7,32 | 2,18 | 0,0281 | 4,36 |
| 3 | ADO2 | 0,763 | 15,04 | 8,24 | 7,28 | 2,15 | 0,0396 | 5,19 |
| 3 | ADO2 | 0,677 | 13,81 | 7,67 | 6,98 | 1,51 | 0,0399 | 5,90 |
| 3 | ADO2 | 0,443 | 14,39 | 6,68 | 7,85 | 1,67 | 0,0268 | 6,05 |
| 3 | ADO2 | 0,606 | 13,95 | 7,65 | 7,47 | 1,62 | 0,0366 | 6,04 |
| 3 | ADO2 | 0,707 | 13,49 | 8,52 | 7,34 | 1,82 | 0,0323 | 4,57 |
| 3 | ADO2 | 0,537 | 12,72 | 6,59 | 6,71 | 1,47 | 0,0292 | 5,44 |
| 3 | ADO2 | 0,591 | 12,64 | 7,66 | 6,28 | 1,51 | 0,0280 | 4,73 |
| 3 | ADO2 | 0,572 | 14,67 | 7,64 | 6,85 | 2,29 | 0,0340 | 5,95 |
| 3 | ADO2 | 0,634 | 13,19 | 8,13 | 7,16 | 1,99 | 0,0573 | 9,04 |
| 3 | ADO2 | 0,616 | 12,89 | 6,07 | 7,10 | 2,03 | 0,0357 | 5,79 |
| 3 | ADO2 | 0,741 | 14,39 | 8,10 | 7,17 | 1,82 | 0,0302 | 4,08 |
| 3 | ADO2 | 0,746 | 14,54 | 7,47 | 6,91 | 2,55 | 0,0373 | 5,00 |
| 3 | ADO2 | 0,563 | 13,31 | 7,81 | 6,62 | 2,12 | 0,0406 | 7,21 |
| 3 | ADO2 | 0,621 | 11,85 | 8,65 | 7,51 | 2,36 | 0,0439 | 7,07 |
| 3 | ADO2 | 0,441 | 9,41 | 6,95 | 6,82 | 2,14 | 0,0319 | 7,23 |
| 3 | ADO2 | 0,715 | 14,14 | 7,17 | 7,56 | 1,73 | 0,0423 | 5,92 |
| 3 | ADO2 | 0,652 | 12,81 | 8,50 | 5,43 | 2,22 | 0,0215 | 3,30 |
| 3 | ADO2 | 0,551 | 14,61 | 6,84 | 9,07 | 1,47 | 0,0402 | 7,30 |
| 3 | ADO2 | 0,501 | 11,15 | 6,83 | 7,09 | 1,09 | 0,0370 | 7,38 |
| 3 | PG | 0,533 | 10,98 | 5,02 | 5,92 | 0,94 | 0,0449 | 8,43 |
| 3 | PG | 0,418 | 8,71 | 5,89 | 4,90 | 0,89 | 0,0343 | 8,20 |
| 3 | PG | 0,539 | 10,31 | 7,12 | 4,17 | 0,90 | 0,0372 | 6,90 |
| 3 | PG | 0,482 | 9,86 | 7,56 | 5,94 | 0,99 | 0,0536 | 11,12 |
| 3 | PG | 0,546 | 10,95 | 4,96 | 6,05 | 1,16 | 0,0403 | 7,38 |
| 3 | PG | 0,383 | 8,36 | 4,37 | 5,05 | 1,85 | 0,0467 | 12,19 |
| 3 | PG | 0,268 | 7,91 | 2,95 | 4,92 | 0,90 | 0,0462 | 17,26 |
| 3 | PG | 0,389 | 9,36 | 6,47 | 6,47 | 1,74 | 0,0759 | 19,49 |
| 3 | PG | 0,429 | 11,36 | 4,76 | 6,59 | 3,15 | 0,0660 | 15,40 |
| 3 | PG | 0,558 | 11,00 | 4,90 | 5,50 | 2,00 | 0,0459 | 8,23 |
| 3 | PG | 0,491 | 9,66 | 6,15 | 6,17 | 1,00 | 0,0590 | 12,01 |
| 3 | PG | 0,489 | 10,00 | 6,41 | 6,08 | 1,98 | 0,0701 | 14,34 |
| 3 | PG | 0,511 | 10,77 | 4,58 | 6,83 | 0,71 | 0,0421 | 8,24 |
| 3 | PG | 0,360 | 8,60 | 6,03 | 6,14 | 1,37 | 0,0613 | 17,05 |
| 3 | PG | 0,484 | 9,68 | 5,20 | 4,99 | 0,93 | 0,0543 | 11,21 |
| 3 | PG | 0,608 | 11,39 | 6,05 | 6,74 | 1,38 | 0,0735 | 12,09 |
| 3 | PG | 0,419 | 9,10 | 5,39 | 7,05 | 2,01 | 0,0874 | 20,85 |
| 3 | PG | 0,443 | 9,78 | 5,06 | 7,66 | 0,71 | 0,0319 | 7,20 |
| 3 | PG | 0,630 | 9,51 | 5,55 | 5,43 | 0,87 | 0,0385 | 6,11 |
| 3 | PG | 0,326 | 9,20 | 4,44 | 3,92 | 1,16 | 0,0444 | 13,62 |
| 3 | PG | 0,360 | 8,32 | 5,50 | 3,77 | 1,54 | 0,0498 | 13,85 |
| 3 | PG | 0,575 | 10,53 | 4,25 | 5,68 | 0,64 | 0,0382 | 6,64 |
| 3 | PG | 0,421 | 10,46 | 5,63 | 5,90 | 0,72 | 0,0346 | 8,22 |
| 3 | PG | 0,293 | 8,53 | 4,83 | 6,46 | 1,25 | 0,0450 | 15,38 |
| 3 | PG | 0,365 | 7,35 | 4,65 | 4,32 | 1,56 | 0,0472 | 12,94 |

**Raw Data**

**Data from juice characteristics of pomegranate accessions**

| Period | Variety | JV | pH | TSS | A | MI |
| --- | --- | --- | --- | --- | --- | --- |
| 1 | ME1 | 63,00 | 4,25 | 13,40 | 0,18 | 74,78 |
| 1 | ME2 | 55,00 | 4,27 | 13,80 | 0,19 | 71,88 |
| 1 | ME3 | 54,00 | 4,19 | 14,00 | 0,19 | 72,92 |
| 1 | ME31 | 62,00 | 4,23 | 14,70 | 0,20 | 71,78 |
| 1 | ME4 | 65,00 | 4,09 | 14,00 | 0,21 | 66,29 |
| 1 | ME5 | 58,00 | 4,15 | 16,40 | 0,24 | 69,26 |
| 1 | ME6 | 58,00 | 4,04 | 15,00 | 0,92 | 16,28 |
| 1 | ME7 | 58,00 | 4,01 | 16,40 | 0,46 | 35,59 |
| 1 | ME8 | 65,00 | 4,36 | 12,40 | 0,17 | 71,76 |
| 1 | ME9 | 42,00 | 3,41 | 16,10 | 0,22 | 73,99 |
| 1 | ME10 | 61,50 | 4,11 | 14,10 | 0,19 | 73,44 |
| 1 | ME11 | 61,00 | 4,09 | 15,20 | 0,20 | 76,61 |
| 1 | ME12 | 40,00 | 3,90 | 14,80 | 0,19 | 77,08 |
| 1 | ME13 | 55,00 | 3,95 | 14,20 | 0,19 | 73,96 |
| 1 | ME14 | 60,00 | 4,31 | 12,40 | 0,27 | 46,13 |
| 1 | ME15 | 58,00 | 4,31 | 14,90 | 0,26 | 58,20 |
| 1 | ME16 | 51,00 | 4,08 | 14,60 | 0,24 | 60,03 |
| 1 | ME17 | 58,00 | 4,17 | 13,80 | 0,19 | 71,88 |
| 1 | ME18 | 61,00 | 4,22 | 15,80 | 0,22 | 70,54 |
| 1 | ME19 | 50,00 | 3,97 | 15,00 | 0,25 | 60,10 |
| 1 | ME20 | 64,00 | 4,09 | 13,00 | 0,22 | 59,74 |
| 1 | ME21 | 53,00 | 4,04 | 15,30 | 0,24 | 64,61 |
| 1 | MA1 | 59,50 | 4,04 | 17,00 | 0,29 | 59,03 |
| 1 | MA2 | 45,00 | 4,08 | 16,00 | 0,21 | 75,76 |
| 1 | MA3 | 60,00 | 4,01 | 15,60 | 0,31 | 50,78 |
| 1 | MA4 | 63,00 | 4,04 | 15,40 | 0,22 | 68,75 |
| 1 | MA5 | 52,00 | 4,23 | 13,80 | 0,21 | 65,34 |
| 1 | BA1 | 40,50 | 2,85 | 15,60 | 1,99 | 7,84 |
| 1 | VA1 | 55,00 | 3,93 | 13,80 | 0,22 | 61,61 |
| 1 | MC1 | 50,00 | 3,92 | 13,80 | 0,23 | 59,90 |
| 1 | MO2 | 50,00 | 3,94 | 15,00 | 0,20 | 75,60 |
| 1 | MO3 | 60,00 | 4,02 | 13,60 | 0,16 | 85,00 |
| 1 | MO4 | 50,00 | 4,02 | 12,40 | 0,22 | 56,99 |
| 1 | MO5 | 48,00 | 4,02 | 13,40 | 0,17 | 77,55 |
| 1 | MO6 | 50,00 | 4,03 | 14,60 | 0,19 | 76,04 |
| 1 | AB1 | 44,00 | 4,17 | 14,00 | 0,18 | 78,13 |
| 1 | PB1 | 50,00 | 3,44 | 15,00 | 0,33 | 45,96 |
| 1 | PTB1 | 50,00 | 3,96 | 15,20 | 0,33 | 46,57 |
| 1 | BB1 | 59,00 | 2,75 | 13,70 | 2,34 | 5,85 |
| 1 | SFB1 | 55,00 | 3,80 | 13,20 | 0,29 | 45,83 |
| 1 | PTO1 | 62,00 | 4,10 | 13,70 | 0,29 | 48,07 |
| 1 | PTO2 | 65,00 | 4,03 | 13,80 | 0,29 | 47,92 |
| 1 | PTO3 | 49,00 | 3,85 | 13,80 | 0,34 | 40,68 |
| 1 | PTO4 | 56,00 | 3,94 | 13,50 | 0,26 | 51,45 |
| 1 | PTO5 | 65,00 | 3,61 | 14,60 | 0,58 | 25,35 |
| 1 | PTO6 | 60,00 | 3,94 | 13,50 | 0,27 | 50,22 |
| 1 | PTO7 | 50,00 | 3,35 | 16,30 | 1,00 | 16,22 |
| 1 | PTO8 | 52,00 | 3,85 | 13,60 | 0,30 | 45,21 |
| 1 | PTO9 | 51,00 | 3,24 | 13,00 | 0,90 | 14,51 |
| 1 | PDO1 | 54,00 | 4,12 | 14,50 | 0,31 | 46,77 |
| 1 | PDO2 | 61,00 | 4,03 | 13,90 | 0,24 | 58,70 |
| 1 | CRO1 | 64,00 | 4,01 | 13,00 | 0,28 | 47,24 |
| 1 | CRO2 | 63,00 | 3,93 | 12,80 | 0,35 | 37,04 |
| 1 | ADO4 | 42,00 | 3,45 | 15,20 | 0,90 | 16,96 |
| 1 | BO1 | 61,00 | 2,71 | 13,60 | 2,32 | 5,85 |
| 1 | ADO2 | 51,00 | 4,06 | 12,00 | 0,26 | 45,73 |
| 1 | ADO3 | 58,00 | 4,10 | 13,80 | 0,28 | 49,64 |
| 1 | PG | 58,00 | 3,42 | 13,00 | 0,72 | 18,14 |
| 2 | ME1 | 59,00 | 4,29 | 15,50 | 0,23 | 67,27 |
[truncated: 569,233 more chars]
