# Supplementary material for: Genetic diversity of pomegranate germplasm collection from Spain determined by fruit, seed, leaf and flower characteristics
Source: PeerJ. 2016 Jul 19;4:e2214. doi: 10.7717/peerj.2214 (PMC4957998; doi:10.7717/peerj.2214)
Supplement: Table S1 [file peerj-04-2214-s002.docx]

Table S1. Mean values of fruit characters of pomegranate accessions

| Variety | FW | FD1 | FD2 | FL1 | FL2 | FL3 | Nc | PcMc | Ec | Rs |
| --- | --- | --- | --- | --- | --- | --- | --- | --- | --- | --- |
| AB1 | 361.4 | 89.6 | 18.5 | 77.2 | 94.0 | 16.9 | 7.0 | 165.4 | 3.5 | 53.7 |
| ADO2 | 361.1 | 90.1 | 19.6 | 76.0 | 95.3 | 19.3 | 6.4 | 137.2 | 3.2 | 61.4 |
| ADO3 | 458.1 | 96.3 | 18.9 | 81.3 | 98.6 | 17.2 | 6.6 | 150.6 | 3.1 | 66.1 |
| BA1 | 400.1 | 92.1 | 22.4 | 78.4 | 96.2 | 17.8 | 6.4 | 186.6 | 3.9 | 52.8 |
| BO1 | 395.7 | 92.4 | 23.0 | 80.1 | 98.4 | 18.3 | 6.4 | 192.1 | 4.4 | 51.4 |
| CRO1 | 456.4 | 98.4 | 19.5 | 82.4 | 99.6 | 17.1 | 6.5 | 163.0 | 3.2 | 63.6 |
| CRO2 | 371.1 | 92.8 | 20.4 | 78.3 | 96.8 | 18.5 | 6.7 | 146.6 | 3.0 | 60.2 |
| MA1 | 362.9 | 90.4 | 19.8 | 77.1 | 91.3 | 14.3 | 6.5 | 165.3 | 4.5 | 53.7 |
| MA2 | 362.0 | 89.7 | 21.2 | 78.1 | 94.3 | 16.1 | 6.7 | 161.2 | 3.6 | 55.0 |
| MA3 | 354.3 | 88.6 | 22.0 | 75.6 | 90.8 | 15.1 | 6.7 | 154.9 | 4.1 | 55.7 |
| MA4 | 367.0 | 89.5 | 18.8 | 77.6 | 93.3 | 15.8 | 6.9 | 153.5 | 4.0 | 57.8 |
| MA5 | 343.5 | 87.7 | 21.7 | 75.8 | 90.1 | 14.3 | 6.5 | 137.5 | 4.1 | 59.4 |
| MC1 | 344.8 | 87.9 | 21.9 | 78.3 | 94.4 | 16.1 | 6.1 | 167.4 | 4.3 | 51.4 |
| ME1 | 321.4 | 85.8 | 19.2 | 73.3 | 91.6 | 18.3 | 6.7 | 134.4 | 3.0 | 58.2 |
| ME10 | 322.1 | 86.3 | 21.2 | 75.7 | 91.2 | 15.5 | 6.5 | 142.2 | 3.8 | 55.1 |
| ME11 | 275.9 | 82.3 | 21.7 | 71.1 | 86.7 | 15.7 | 6.9 | 130.3 | 3.9 | 52.1 |
| ME12 | 340.9 | 87.2 | 20.4 | 73.4 | 89.5 | 16.1 | 7.3 | 140.3 | 2.9 | 57.7 |
| ME13 | 332.7 | 85.0 | 19.6 | 71.9 | 88.7 | 16.7 | 6.3 | 141.5 | 4.4 | 57.9 |
| ME14 | 333.5 | 88.3 | 23.8 | 74.0 | 90.7 | 16.7 | 6.5 | 156.8 | 4.6 | 52.7 |
| ME16 | 350.0 | 87.9 | 20.6 | 77.8 | 92.9 | 15.1 | 6.3 | 149.3 | 4.1 | 57.0 |
| ME17 | 350.7 | 89.8 | 24.5 | 75.8 | 91.6 | 15.8 | 6.6 | 166.1 | 4.6 | 52.1 |
| ME18 | 335.1 | 85.3 | 21.4 | 77.8 | 93.1 | 15.2 | 6.4 | 148.1 | 4.2 | 55.5 |
| ME19 | 347.2 | 89.4 | 21.3 | 77.2 | 92.0 | 14.7 | 6.7 | 142.0 | 4.0 | 58.7 |
| ME2 | 301.3 | 84.3 | 20.8 | 72.9 | 90.7 | 17.8 | 6.6 | 134.0 | 3.7 | 55.7 |
| ME20 | 346.6 | 88.1 | 20.8 | 74.9 | 90.6 | 15.8 | 7.1 | 139.8 | 3.3 | 58.9 |
| ME21 | 309.5 | 82.4 | 19.1 | 71.6 | 88.0 | 16.4 | 6.7 | 142.8 | 3.5 | 53.5 |
| ME3 | 356.1 | 88.7 | 20.2 | 76.5 | 92.9 | 16.4 | 6.2 | 137.3 | 3.6 | 61.2 |
| ME31 | 329.0 | 87.6 | 19.4 | 75.0 | 92.9 | 17.8 | 6.8 | 138.3 | 3.1 | 57.8 |
| ME4 | 278.4 | 83.6 | 21.1 | 71.8 | 88.4 | 16.6 | 6.8 | 124.5 | 4.0 | 54.7 |
| ME5 | 312.3 | 85.9 | 21.4 | 73.5 | 90.5 | 17.0 | 6.8 | 139.4 | 4.6 | 54.9 |
| ME6 | 348.3 | 88.1 | 21.6 | 77.4 | 93.3 | 15.9 | 6.7 | 148.1 | 4.4 | 57.1 |
| ME7 | 317.0 | 83.6 | 20.5 | 73.4 | 88.6 | 15.2 | 6.5 | 138.3 | 4.0 | 55.6 |
| ME8 | 300.6 | 82.9 | 20.2 | 72.3 | 88.0 | 15.6 | 6.5 | 141.9 | 4.1 | 52.7 |
| ME9 | 328.5 | 87.3 | 22.5 | 75.7 | 92.6 | 16.9 | 6.5 | 151.9 | 4.4 | 52.8 |
| MO2 | 350.7 | 87.0 | 19.4 | 75.6 | 90.7 | 15.1 | 6.6 | 145.7 | 3.4 | 57.1 |
| MO3 | 369.7 | 90.9 | 20.5 | 78.9 | 95.4 | 16.5 | 6.6 | 154.9 | 3.4 | 57.5 |
| MO4 | 352.1 | 88.1 | 21.2 | 75.7 | 91.8 | 16.2 | 6.6 | 145.8 | 3.8 | 57.7 |
| MO5 | 375.9 | 90.4 | 21.1 | 77.0 | 91.8 | 14.8 | 6.9 | 150.5 | 3.1 | 59.9 |
| MO6 | 379.6 | 91.8 | 20.6 | 76.6 | 93.0 | 16.4 | 6.6 | 155.0 | 3.5 | 58.7 |
| PB1 | 366.6 | 90.8 | 19.7 | 75.5 | 92.8 | 17.3 | 7.0 | 168.4 | 3.6 | 53.9 |
| PDO2 | 436.5 | 95.8 | 18.0 | 82.8 | 96.8 | 14.0 | 6.8 | 179.5 | 3.2 | 58.8 |
| PG | 331.5 | 87.8 | 17.9 | 75.2 | 90.3 | 15.1 | 6.3 | 146.3 | 3.7 | 55.3 |
| PTB1 | 436.0 | 97.0 | 17.3 | 84.7 | 101.7 | 17.1 | 6.6 | 192.2 | 3.0 | 55.8 |
| PTO2 | 428.5 | 97.8 | 19.5 | 83.8 | 102.3 | 18.5 | 6.4 | 166.7 | 3.6 | 60.5 |
| PTO3 | 428.4 | 96.1 | 19.3 | 82.0 | 98.6 | 16.6 | 6.4 | 163.5 | 3.7 | 61.3 |
| PTO4 | 425.4 | 95.8 | 19.5 | 80.7 | 95.8 | 15.1 | 6.2 | 144.3 | 3.3 | 65.8 |
| PTO5 | 339.4 | 86.3 | 18.2 | 76.8 | 90.0 | 13.2 | 6.1 | 140.4 | 4.1 | 58.3 |
| PTO6 | 412.7 | 94.7 | 19.7 | 80.0 | 96.3 | 16.3 | 6.4 | 139.9 | 3.4 | 65.9 |
| PTO7 | 464.2 | 95.9 | 19.1 | 82.4 | 98.9 | 16.5 | 6.4 | 153.9 | 3.2 | 65.9 |
| PTO8 | 369.5 | 86.7 | 17.8 | 75.3 | 93.9 | 18.6 | 6.3 | 129.3 | 2.6 | 64.1 |
| SFB1 | 358.8 | 89.0 | 19.4 | 78.1 | 95.2 | 17.2 | 6.1 | 136.4 | 3.2 | 61.0 |
| VA1 | 341.7 | 87.2 | 22.0 | 76.6 | 91.7 | 15.1 | 6.3 | 164.6 | 3.8 | 51.7 |

For explanation of character symbols, see Material and methods
