# Supplementary material for: Genetic diversity of pomegranate germplasm collection from Spain determined by fruit, seed, leaf and flower characteristics
Source: PeerJ. 2016 Jul 19;4:e2214. doi: 10.7717/peerj.2214 (PMC4957998; doi:10.7717/peerj.2214)
Supplement: Table S3 [file peerj-04-2214-s004.docx]

Table S3. Mean values of leaf and flowers characteristics of pomegranate accessions

|  | Leafs | | | | | | Flowers | | | | | | | |
| --- | --- | --- | --- | --- | --- | --- | --- | --- | --- | --- | --- | --- | --- | --- |
| Variety | LW | Ll | Lt | Lp | Ll/LW | LS | FD | FL | Np | Lp | Wp | Ns | LS | NS |
| AB1 | 20.8 | 61.2 | 68.0 | 6.8 | 3.0 | 8.7 | 15.5 | 33.6 | 6.2 | 26.5 | 20.0 | 6.2 | 21.3 | 375.0 |
| ADO2 | 19.2 | 51.5 | 57.0 | 5.5 | 2.7 | 7.3 | 10.3 | 31.4 | 6.3 | 19.9 | 15.5 | 6.3 | 12.4 | 368.6 |
| ADO3 | 20.6 | 55.0 | 60.3 | 5.3 | 2.7 | 7.7 | 10.4 | 30.5 | 6.2 | 19.8 | 15.7 | 6.2 | 13.6 | 340.6 |
| BA1 | 22.8 | 60.3 | 65.6 | 5.2 | 2.7 | 8.3 | 16.3 | 34.7 | 7.7 | 24.9 | 17.0 | 7.7 | 20.7 | 312.6 |
| BO1 | 21.2 | 62.2 | 68.7 | 6.4 | 3.0 | 9.5 | 16.5 | 37.5 | 7.8 | 24.7 | 17.9 | 7.8 | 23.0 | 364.0 |
| CRO1 | 19.1 | 49.6 | 54.7 | 5.2 | 2.6 | 7.1 | 10.3 | 30.4 | 6.1 | 20.6 | 15.8 | 6.1 | 11.8 | 325.8 |
| CRO2 | 19.1 | 53.0 | 58.4 | 5.4 | 2.8 | 7.6 | 11.0 | 33.0 | 6.6 | 21.9 | 16.3 | 6.6 | 13.1 | 339.4 |
| MA1 | 21.3 | 57.3 | 62.3 | 5.0 | 2.7 | 8.2 | 10.5 | 28.0 | 6.8 | 23.1 | 16.4 | 6.8 | 9.4 | 349.3 |
| MA2 | 22.0 | 54.0 | 60.1 | 6.1 | 2.5 | 8.2 | 12.7 | 30.5 | 7.3 | 22.3 | 17.4 | 7.5 | 17.1 | 281.6 |
| MA3 | 23.1 | 53.9 | 59.9 | 6.0 | 2.4 | 8.0 | 13.8 | 32.3 | 6.5 | 22.8 | 16.9 | 6.5 | 20.9 | 335.6 |
| MA4 | 22.4 | 52.9 | 58.8 | 6.0 | 2.4 | 8.3 | 13.1 | 30.9 | 6.6 | 22.5 | 17.4 | 6.6 | 19.3 | 365.6 |
| MA5 | 22.0 | 55.9 | 61.9 | 6.0 | 2.6 | 8.5 | 12.9 | 30.7 | 6.3 | 21.0 | 16.4 | 6.3 | 18.8 | 339.0 |
| MC1 | 22.4 | 56.8 | 63.2 | 6.4 | 2.6 | 8.7 | 13.4 | 30.8 | 6.4 | 23.0 | 17.8 | 6.4 | 19.8 | 314.9 |
| ME1 | 21.2 | 50.3 | 55.6 | 5.3 | 2.4 | 7.5 | 16.0 | 33.9 | 7.0 | 23.4 | 18.1 | 7.0 | 16.0 | 232.1 |
| ME10 | 21.7 | 52.3 | 58.3 | 6.0 | 2.4 | 7.6 | 14.8 | 23.5 | 6.4 | 22.8 | 18.4 | 6.4 | 13.9 | 238.0 |
| ME11 | 21.4 | 52.5 | 58.6 | 6.0 | 2.5 | 6.9 | 15.4 | 26.7 | 6.2 | 21.6 | 17.0 | 6.2 | 11.5 | 231.7 |
| ME12 | 20.2 | 47.9 | 53.4 | 5.5 | 2.4 | 7.0 | 14.6 | 23.8 | 6.0 | 23.4 | 16.5 | 6.0 | 9.6 | 247.4 |
| ME13 | 20.2 | 56.6 | 62.7 | 6.1 | 2.8 | 7.5 | 13.3 | 22.9 | 6.1 | 19.9 | 15.1 | 6.1 | 10.3 | 245.4 |
| ME14 | 20.9 | 49.2 | 54.1 | 4.9 | 2.4 | 7.7 | 12.8 | 20.1 | 6.2 | 20.3 | 14.9 | 6.2 | 11.0 | 224.9 |
| ME16 | 18.3 | 52.4 | 57.9 | 5.5 | 3.0 | 6.7 | 13.5 | 22.6 | 6.0 | 20.5 | 14.3 | 6.0 | 9.1 | 251.7 |
| ME17 | 20.4 | 54.6 | 59.3 | 4.7 | 2.7 | 8.4 | 15.1 | 26.6 | 6.0 | 22.1 | 17.2 | 6.0 | 13.0 | 290.4 |
| ME18 | 20.4 | 48.9 | 54.6 | 5.6 | 2.4 | 8.1 | 15.2 | 23.4 | 6.1 | 22.8 | 18.2 | 6.1 | 9.8 | 250.9 |
| ME19 | 20.1 | 47.8 | 52.1 | 4.3 | 2.4 | 7.7 | 15.2 | 27.0 | 6.2 | 22.4 | 18.9 | 6.2 | 9.8 | 248.8 |
| ME2 | 22.2 | 50.6 | 56.2 | 5.6 | 2.3 | 8.9 | 16.1 | 35.1 | 7.0 | 23.1 | 17.7 | 7.0 | 15.3 | 209.7 |
| ME20 | 20.5 | 54.6 | 59.4 | 4.9 | 2.7 | 8.5 | 14.4 | 21.2 | 6.1 | 20.9 | 15.6 | 6.1 | 7.5 | 246.1 |
| ME21 | 21.2 | 47.2 | 52.4 | 5.2 | 2.3 | 7.5 | 15.1 | 25.6 | 6.4 | 20.6 | 17.1 | 6.3 | 10.6 | 244.6 |
| ME3 | 23.2 | 49.6 | 55.5 | 5.9 | 2.1 | 7.8 | 17.4 | 31.7 | 7.4 | 23.2 | 18.7 | 7.4 | 12.5 | 202.4 |
| ME31 | 22.6 | 53.1 | 58.8 | 5.7 | 2.4 | 8.3 | 17.1 | 31.6 | 6.6 | 21.2 | 17.8 | 6.6 | 10.9 | 208.3 |
| ME4 | 22.8 | 48.8 | 54.3 | 5.6 | 2.2 | 8.2 | 13.5 | 27.2 | 6.4 | 22.6 | 17.7 | 6.4 | 18.4 | 228.5 |
| ME5 | 22.9 | 54.0 | 60.4 | 6.3 | 2.4 | 8.4 | 16.7 | 28.6 | 6.3 | 22.1 | 18.7 | 6.2 | 15.2 | 280.9 |
| ME6 | 21.8 | 51.5 | 57.6 | 6.2 | 2.4 | 7.8 | 17.8 | 29.3 | 6.1 | 22.2 | 17.6 | 6.1 | 15.0 | 299.2 |
| ME7 | 21.9 | 52.4 | 58.4 | 6.0 | 2.4 | 7.8 | 16.7 | 29.3 | 6.2 | 23.8 | 18.6 | 6.2 | 13.5 | 250.1 |
| ME8 | 22.3 | 52.2 | 57.7 | 5.5 | 2.4 | 8.0 | 14.6 | 25.8 | 6.2 | 22.4 | 16.8 | 6.2 | 18.4 | 251.4 |
| ME9 | 20.8 | 50.0 | 55.6 | 5.6 | 2.4 | 7.5 | 15.8 | 29.3 | 6.2 | 22.8 | 18.7 | 6.2 | 14.8 | 250.1 |
| MO2 | 21.7 | 48.8 | 54.1 | 5.4 | 2.3 | 9.0 | 14.6 | 22.2 | 6.4 | 20.7 | 17.1 | 6.3 | 8.0 | 255.8 |
| MO3 | 22.5 | 56.1 | 62.2 | 6.1 | 2.5 | 8.5 | 16.8 | 30.9 | 7.8 | 22.5 | 17.1 | 7.8 | 17.7 | 364.4 |
| MO4 | 23.3 | 54.9 | 60.8 | 5.9 | 2.4 | 8.5 | 14.2 | 31.4 | 7.5 | 24.6 | 18.3 | 7.5 | 19.2 | 356.0 |
| MO5 | 23.0 | 56.2 | 62.6 | 6.4 | 2.5 | 8.8 | 15.2 | 34.4 | 7.5 | 24.3 | 18.8 | 7.5 | 21.5 | 383.0 |
| MO6 | 21.7 | 54.3 | 60.1 | 5.7 | 2.5 | 8.1 | 11.8 | 30.6 | 6.7 | 20.6 | 15.8 | 6.7 | 15.5 | 328.3 |
| PB1 | 21.7 | 61.0 | 66.1 | 5.1 | 2.9 | 8.6 | 10.6 | 33.9 | 6.7 | 22.5 | 18.5 | 6.7 | 14.3 | 212.8 |
| PDO2 | 19.9 | 52.6 | 58.2 | 5.6 | 2.7 | 8.0 | 11.8 | 34.1 | 6.8 | 25.7 | 20.5 | 6.8 | 14.1 | 206.2 |
| PG | 17.5 | 51.3 | 57.5 | 6.1 | 3.0 | 7.0 | 11.9 | 34.7 | 6.6 | 19.6 | 17.5 | 6.6 | 15.9 | 240.4 |
| PTB1 | 20.8 | 60.2 | 66.1 | 5.8 | 2.9 | 9.1 | 13.1 | 35.3 | 7.6 | 19.9 | 15.7 | 7.6 | 16.4 | 324.8 |
| PTO2 | 19.2 | 52.5 | 57.6 | 5.2 | 2.7 | 7.4 | 12.0 | 31.8 | 7.1 | 21.5 | 15.9 | 7.1 | 11.9 | 365.5 |
| PTO3 | 20.1 | 49.3 | 54.2 | 4.9 | 2.5 | 7.6 | 11.6 | 29.6 | 6.8 | 21.2 | 16.2 | 6.8 | 10.0 | 380.2 |
| PTO4 | 18.5 | 49.1 | 54.6 | 5.5 | 2.7 | 7.0 | 12.0 | 35.6 | 7.8 | 20.9 | 16.5 | 7.8 | 13.4 | 312.7 |
| PTO5 | 18.8 | 47.2 | 52.4 | 5.2 | 2.6 | 6.8 | 11.8 | 35.1 | 7.1 | 25.6 | 18.3 | 7.1 | 11.8 | 422.2 |
| PTO6 | 21.2 | 54.2 | 58.5 | 4.3 | 2.6 | 10.0 | 12.9 | 35.6 | 8.4 | 18.7 | 13.6 | 8.4 | 14.4 | 327.4 |
| PTO7 | 20.0 | 47.1 | 52.2 | 5.1 | 2.4 | 6.9 | 10.4 | 31.4 | 7.7 | 19.7 | 15.0 | 7.7 | 8.9 | 364.0 |
| PTO8 | 19.5 | 52.5 | 57.9 | 5.3 | 2.7 | 7.6 | 12.5 | 34.2 | 7.6 | 19.1 | 14.3 | 7.6 | 15.4 | 297.8 |
| SFB1 | 20.7 | 55.3 | 60.7 | 5.4 | 2.7 | 8.0 | 14.4 | 36.2 | 7.0 | 24.0 | 18.8 | 7.0 | 12.4 | 289.6 |
| VA1 | 20.5 | 57.9 | 63.9 | 5.9 | 2.8 | 7.9 | 11.9 | 28.4 | 6.2 | 20.3 | 15.7 | 6.2 | 14.5 | 356.6 |

For explanation of character symbols, see Material and methods
